# Dynamics of Solid Proteins by Means of Nuclear Magnetic Resonance Relaxometry

**DOI:** 10.3390/biom9110652

**Published:** 2019-10-25

**Authors:** Danuta Kruk, Elzbieta Masiewicz, Anna M. Borkowska, Pawel Rochowski, Pascal H. Fries, Lionel M. Broche, David J. Lurie

**Affiliations:** 1 Faculty of Mathematics and Computer Science, University of Warmia and Mazury in Olsztyn, Słoneczna 54, 10-710 Olsztyn, Poland; elzbieta.masiewicz@matman.uwm.edu.pl (E.M.); a.borkowska@uwm.edu.pl (A.M.B.); pawel.rochowski@gmail.com (P.R.); 2 Laboratoire de Reconnaissance Ionique et Chimie de Coordination, Service de Chimie Inorganique et Biologique (UMR E-3 CEA/UJF), CEA-Grenoble, INAC, 17 rue des Martyrs, CEDEX 09, 38054 Grenoble, France; pascal-h.fries@cea.fr; 3 Bio-Medical Physics, School of Medicine, Medical Sciences & Nutrition, University of Aberdeen, Foresterhill, Aberdeen, Scotland AB25 2ZD, UK; l.broche@abdn.ac.uk (L.M.B.); d.lurie@abdn.ac.uk (D.J.L.)

**Keywords:** proteins, relaxation, dynamics, NMR relaxometry, quadrupole relaxation enhancement, solids

## Abstract

^1^H Nuclear magnetic resonance (NMR) relaxometry was exploited to investigate the dynamics of solid proteins. The relaxation experiments were performed at 37 °C over a broad frequency range, from approximately 10 kHz to 40 MHz. Two relaxation contributions to the overall ^1^H spin–lattice relaxation were revealed; they were associated with ^1^H–^1^H and ^1^H–^14^N magnetic dipole–dipole interactions, respectively. The ^1^H–^1^H relaxation contribution was interpreted in terms of three dynamical processes occurring on timescales of 10^−6^ s, 10^−7^ s, and 10^−8^ s, respectively. The ^1^H–^14^N relaxation contribution shows quadrupole relaxation enhancement effects. A thorough analysis of the data was performed revealing similarities in the protein dynamics, despite their different structures. Among several parameters characterizing the protein dynamics and structure (e.g., electric field gradient tensor at the position of ^14^N nuclei), the orientation of the ^1^H–^14^N dipole–dipole axis, with respect to the principal axis system of the electric field gradient, was determined, showing that, for lysozyme, it was considerably different than for the other proteins. Moreover, the validity range of a closed form expression describing the ^1^H–^14^N relaxation contribution was determined by a comparison with a general approach based on the stochastic Liouville equation.

## 1. Introduction

The combined effect of the structure and dynamics of biological macromolecules is essential for their biological function. This explains why the determination of protein structures and their dynamical properties is one of the most pertinent questions in science. As far as protein structures are concerned, high-resolution nuclear magnetic resonance (NMR) spectroscopy is a leading method providing access to multi-dimensional protein conformation [1,2,3]. Much less is known about protein dynamics, especially on a long-term scale; this is due to the lack of appropriate experimental methods providing information on dynamic processes over a broad time scale with molecular (atomistic) resolution—NMR relaxometry offers such an opportunity.

Nuclear magnetic resonance relaxometry is one of the main methods providing information about molecular dynamics and structure [4,5,6,7,8]. Standard NMR relaxation experiments are performed only at a single relatively high magnetic field (resonance frequency), while fast field-cycling (FFC) NMR relaxometry [9,10] allows one to carry out relaxation studies in a remarkably broad frequency range encompassing five orders of magnitude, from approximately 1 kHz to 120 MHz (referring to ^1^H resonance frequency). As a consequence, NMR relaxometry can detect motional processes across a broad range of time scales (from ms to ps) in a single experiment [4,5,6]. Moreover, frequency-dependent relaxation studies possess the exceptional potential to reveal the underlying mechanisms of molecular motion (not only its time scale) [4,5,8,11,12,13,14,15,16,17,18,19]. The dominant mechanism of ^1^H relaxation is provided by magnetic dipole–dipole interactions. The interactions stochastically fluctuate in time due to the molecular dynamics. According to spin relaxation theory, relaxation rates are given as linear combinations of spectral density functions (Fourier transform of time correlation function) of the motion modulating the interactions. Mathematical forms of spectral densities characterizing different kinds of dynamics are essentially different, thus relaxation dispersion experiments reveal the mechanism of molecular motion.

As spin relaxation is a complex, quantum-mechanical phenomenon, the unique advantages of NMR relaxometry are even more far reaching. An example of quantum-mechanical interplay among spin interactions is quadrupole relaxation enhancement (QRE) [20,21,22,23,24,25,26,27,28]. This effect involves at least one nucleus of a spin-quantum number I=1/2 (typically ^1^H) and one nucleus of spin quantum number S≥1. In this work, we focused on ^14^N (S=1), as nitrogen is one of the fundamental components of organic matter, from simple molecules via proteins to tissues. The two nuclei (^1^H and ^14^N) have to be mutually coupled by ^1^H–^14^N dipole–dipole interactions, providing a relaxation pathway for ^1^H. The energy level structure of ^1^H is fully determined by its Zeeman interaction and, hence, its magnetic spin quantum number, $m_{I}=\pm 1/2$. The ^14^N nucleus experiences two kinds of interactions: Zeeman interaction and quadrupole coupling, i.e., a coupling with an electric field gradient tensor at its position. For slow dynamics, the energy level structure of ^14^N is determined by a superposition of its Zeeman and quadrupole interactions. When the ^1^H resonance frequency (the transition frequency among the ^1^H energy levels) matches one of the ^14^N transition frequencies, the ^1^H polarization can be taken over by ^14^N, leading to a frequency-specific enhancement of the ^1^H spin–lattice relaxation rate referred to as QRE; the ^1^H spin–lattice relaxation maxima are called quadrupole peaks. It is clear that the positions of the quadrupole peaks depend on the quadrupole parameters which are determined by the electric field gradient tensor at the ^14^N position. Consequently, even subtle changes in the electronic structure around ^14^N are reflected by changes in the position and shape of the quadrupole peaks. Moreover, the intensity of the effect provides information about the fraction of molecules undergoing slow dynamics. Thus, the QRE is a very sensitive fingerprint of molecular arrangement which can be exploited in material science [25,26], biology [27,28,29,30], and medicine [31,32,33].

As NMR relaxometry studies for solids are rare, one can provide only a few examples of such results for solid proteins. The results are, however, of high importance because of the proposed theory. It has been assumed that the shape of the frequency dependence of ^1^H spin–lattice relaxation in solid proteins can be attributed to structural fluctuations along the backbone. Consequently, a power-law model has been developed, with an amplitude reflecting the highest vibrational frequency of the fluctuations and a slope related to their fractal dimensionality [34]. This concept has also been exploited for hydrated immobilized proteins [35] and confined proteins [36]. The theory has been revisited [37] by including strong dipole–dipole interactions between the side-chain protons and the protein backbone. The extended model has been applied to lyophilized globular proteins. In the present work, we did not use the power-law theory, but a concept referred to as a “model free approach”. This concept is based on a decomposition of the overall spin–lattice relaxation rates originating from ^1^H–^1^H dipole–dipole interactions into contributions associated with dynamical processes occurring on considerably different time scales. The concept was proposed in Reference [38] and then used for the analysis of ^1^H relaxation data for proteins in solution [39,40], in sediments [41], as well as for polymers [42].

In this work NMR relaxometry has been applied to extract information about the dynamics of solid proteins; bovine serum albumin (BSA), albumin from human plasma (AHP), elastin from bovine neck ligament, and lysozyme from hen egg whites have been used as samples.

The paper is organized as follows. In Section 2, the theoretical foundation of ^1^H relaxation processes in solids is presented, and special attention was paid to the description of the QRE effects. Section 2 also includes the experimental details. While in Section 3, the results obtained are presented and discussed. Section 4 contains the concluding remarks.

## 2. Materials and Methods

### 2.1. Theory: Decomposition of ^1^H Spin-Lattice Relaxation Profiles

The overall relaxation of the ^1^H spin–lattice relaxation rate, $R_{1}^{}\left(\omega_{H}\right)$ ($\omega_{H}$) denotes the ^1^H resonance frequency in angular frequency units) for proteins resulting from ^1^H–^1^H and ^1^H–^14^N magnetic dipole–dipole interactions, leading to the corresponding relaxation rates $R_{1}^{HH}\left(\omega_{H}\right)$ and $R_{1}^{HN}\left(\omega_{H}\right)$, respectively:

(1) $$R_{1}^{}\left(\omega_{H}\right)=R_{1}^{HH}\left(\omega_{H}\right)+R_{1}^{HN}\left(\omega_{H}\right)$$

In the case of a single dynamical process involved in the relaxation, the $R_{1}^{HH}\left(\omega_{H}\right)$ term is given as [43,44,45]:

(2) $$R_{1}^{HH}\left(\omega_{H}\right)=C^{HH}\left(\frac{\tau_{c}}{1+\omega_{H}^{2}\tau_{c}^{2}}+\frac{4\tau_{c}}{1+4\omega_{H}^{2}\tau_{c}^{2}}\right)$$

where $\tau_{c}$ denotes the correlation time of this dynamical process, while $C^{HH}$ is referred to as a dipolar relaxation constant; it yields: $C^{HH}=\frac{3}{10}{\left(\frac{\mu_{0}}{4\pi }\frac{\gamma_{H}^{2}\hbar }{r_{HH}^{3}}\right)}^{2}$, where $\gamma_{H}$ denotes the ^1^H gyromagnetic factor, while $r_{HH}$ should be treated as an “effective” inter-spin distance accounting for dipole–dipole interactions among several pairs of protons. As pointed out in the Introduction, NMR relaxation studies performed as a function of frequency provides access to dynamical processes occurring on different time scales. Slow dynamics dominate the relaxation at low frequencies, then, with increasing frequency, progressively faster dynamics come into play, while the relaxation contributions associated with a slower motion decrease. Anticipating the results, the $R_{1}^{HH}\left(\omega_{H}\right)$ relaxation rates for solid proteins can be expressed as a sum of the following terms:

(3) $$\begin{array}{ll} R_{1}^{HH}\left(\omega_{H}\right) & =C_{s}^{HH}\left(\frac{\tau_{s}}{1+\omega_{H}^{2}\tau_{s}^{2}}+\frac{4\tau_{s}}{1+4\omega_{H}^{2}\tau_{s}^{2}}\right)+C_{i}^{HH}\left(\frac{\tau_{s}}{1+\omega_{H}^{2}\tau_{i}^{2}}+\frac{4\tau_{i}}{1+4\omega_{H}^{2}\tau_{i}^{2}}\right)\\ & +C_{f}^{HH}\left(\frac{\tau_{f}}{1+\omega_{H}^{2}\tau_{f}^{2}}+\frac{4\tau_{f}}{1+4\omega_{H}^{2}\tau_{f}^{2}}\right)+A \end{array}$$

where the pairs of parameters, $\left(C_{s}^{HH},\tau_{s}\right),\;\left(C_{i}^{HH},\tau_{i}\right),\;\left(C_{f}^{HH},\tau_{f}\right)$ refer to slow, intermediate, and fast dynamical processes, while the frequency independent term, A, describes a relaxation contribution associated with the dynamics of a timescale being shorter than 10^−9^ s. For such a short correlation time, $\omega_{H}\tau_{c}\ll 1$ applies and, in consequence, the corresponding relaxation rate does not show a dependence on $\omega_{H}$. For convenience we shall refer to the first, second, and third terms of Equation (3) as: $R_{1,s}^{HH},\;R_{1,i}^{HH}$ and $R_{1,f}^{HH}$, respectively. At this stage we wish to clearly state that we do not contradict the power-law theory proposed in References [34,35,36,37]. Nevertheless, we exploited an alternative that is (in our opinion) worthy of consideration [38,39,40,41,42].

In response to the growing interest in QRE effects as a source of information about molecular dynamics and structure, recently a closed form description of the QRE effect has been provided [46]. According to this model, the ^1^H spin–lattice relaxation rate originating from the ^1^H-^14^N dipole–dipole interaction, $R_{1}^{HN}\left(\omega_{H}\right)$, is given as:

(4) $$R_{1}^{HN}\left(\omega_{H}\right)=C_{}^{HN}\times \left[\begin{array}{l} \left(\frac{1}{3}+\sin^{2}\Theta \cos^{2}\Phi \right)\left(\frac{\tau_{Q}}{1+{\left(\omega_{H}-\omega_{-}\right)}^{2}\tau_{Q}^{2}}+\frac{\tau_{Q}}{1+{\left(\omega_{H}+\omega_{-}\right)}^{2}\tau_{Q}^{2}}\right)+\\ \left(\frac{1}{3}+\sin^{2}\Theta \sin^{2}\Phi \right)\left(\frac{\tau_{Q}}{1+{\left(\omega_{H}-\omega_{+}\right)}^{2}\tau_{Q}^{2}}+\frac{\tau_{Q}}{1+{\left(\omega_{H}+\omega_{+}\right)}^{2}\tau_{Q}^{2}}\right)+\\ \left(\frac{1}{3}+\cos^{2}\Theta \right)\left(\frac{\tau_{Q}}{1+{\left(\omega_{H}-\omega_{0}\right)}^{2}\tau_{Q}^{2}}+\frac{\tau_{Q}}{1+{\left(\omega_{H}+\omega_{0}\right)}^{2}\tau_{Q}^{2}}\right) \end{array}\right]$$

where $C_{}^{HN}$ denotes a ^1^H–^14^N dipolar relaxation constant. For a single ^1^H–^14^N spin pair, it is defined as $C^{HN}=\frac{2}{3}{\left(\frac{\mu_{0}}{4\pi }\frac{\gamma_{H}\gamma_{N}\hbar }{r_{HN}^{3}}\right)}^{2}$, where $r_{HN}$ is the inter-spin distance. When the quadrupole coupling of ^14^N dominates its Zeeman coupling (this happens in most cases as the gyromagnetic factor, $\gamma_{N}$, of ^14^N is small), the energy levels of ^14^N are fully determined by the quadrupole coupling and given as $E_{1}=\frac{1}{4}a_{Q}(1-\eta )$, $E_{2}=-\frac{1}{2}a_{Q}$, $E_{3}=\frac{1}{4}a_{Q}(1+\eta )$, where $a_{Q}$ and η determine the amplitude and the asymmetry parameter of the quadrupole coupling, respectively. The amplitude is defined as: $a_{Q}=e^{2}qQ/h$, where Q denotes the quadrupolar moment of the nucleus, while q is the zz component of the electric field gradient tensor. In consequence, the three transition frequencies yield:$\nu_{-}=\frac{\omega_{-}}{2\pi }=\frac{3}{4}a_{Q}\left(1-\frac{\eta }{3}\right)$, $\nu_{+}=\frac{\omega_{+}}{2\pi }=\frac{3}{4}a_{Q}\left(1+\frac{\eta }{3}\right)$, and $\nu_{0}=\nu_{+}-\nu_{-}=\frac{\omega_{0}}{2\pi }=\frac{1}{2}\eta a_{Q}$ [20,21,25,26,29,30,46]. The angles Θ and Φ describe the orientation of the ^1^H–^14^N dipole–dipole axis with respect to the principal axis system of the electric field gradient at the position of ^14^N, $\tau_{Q}$ denotes the correlation time characterizing fluctuations of the ^1^H–^14^N dipole–dipole coupling, $r_{HN}$ denotes the ^1^H–^14^N inter-spin distance, while $\gamma_{N}$ is the ^14^N gyromagnetic factor. Equation (4) is valid in the two limiting cases: when $\omega_{Q}\tau_{Q}\gg 1$ or when $\omega_{Q}\tau_{Q}\ll 1$ ($\omega_{Q}$ denotes $a_{Q}$ in angular frequency units). When the last condition holds, Equation (1) converges to $R_{1}^{HN}\left(\omega_{H}\right)=\frac{8}{3}{\left(\frac{\mu_{0}}{4\pi }\frac{\gamma_{H}\gamma_{N}\hbar }{r_{HN}^{3}}\right)}^{2}\frac{\tau_{Q}}{1+\omega_{H}^{2}\tau_{Q}^{2}}$. In this case, there are obviously no quadrupole peaks as the quadrupole coupling does not contribute to the ^14^N energy level structure.

### 2.2. Theory: Validity Range of the Description of QRE effects

The great advantage of Equation (4) is its very simple mathematical form. However, when one departs from the limit of very slow dynamics, $\omega_{Q}\tau_{rot}\gg 1$, it may not be used for a theoretical interpretation of the QRE effects. To describe the ^1^H–^14^N spin–lattice relaxation contribution for an arbitrary time scale of the molecular dynamics one has to use a much more cumbersome description, based on the Stochastic Liouville Equation (SLE) [22,43,47,48]. As the spin and spatial variables cannot be separated in the stochastic Liouville approach, one constructs a basis being an outer product of the spin and rotational variables: $|O_{\alpha })=|\Sigma ,\sigma )⊗|L,K,M)$, where Σ ranges from 1 to (2S+1), i.e., 3 for ^14^N, σ=−Σ,…,Σ while the quantum numbers *L*, *K*, *M* correspond to the indices of Wigner rotation matrices; for practical calculations it is sufficient to set *L* = 1, …, 8, *K* = −*L*, …, *L*, *M* = −*L*, …, *L*. Then the relaxation rate, $R_{1}^{HN}$, is given as [22,49,50,51,52]:

(5) R1HN=Re{[T11]+[M]−1[T11]}

where the matrix [*M*] is defined in the $\left\{|O_{\alpha })(O_{\beta }|\right\}$ basis constructed from pairs of the $|O_{\alpha })$ vectors. The matrix elements are given as [21,22,25,51,52]:

(6) $$\begin{array}{l} {\left[M\right]}_{\alpha ,\beta }=\frac{\sqrt{30}}{4}a_{Q}{\left(-1\right)}^{\sigma^{\prime }}F_{\left|K-K^{\prime }\right|}^{2}\left[{\left(-1\right)}^{\Sigma^{\prime }+\Sigma }-1\right]\times \sqrt{\left(2L^{\prime }+1\right)\left(2L+1\right)\left(2\Sigma^{\prime }+1\right)\left(2\Sigma +1\right)}\times \\ \left(\begin{array}{ccc} L^{\prime } & 2 & L\\ -K^{\prime } & K^{\prime }-K & K \end{array}\right)\left(\begin{array}{ccc} L^{\prime } & 2 & L\\ -M^{\prime } & M^{\prime }-M & M \end{array}\right)\left(\begin{array}{ccc} \Sigma^{\prime } & 2 & \Sigma \\ -\sigma^{\prime } & B^{\prime }-B & \sigma \end{array}\right)\left\{\begin{array}{ccc} \Sigma^{\prime } & 2 & \Sigma \\ 1 & 1 & 1 \end{array}\right\}\times \\ \delta_{LL^{\prime }}\delta_{KK^{\prime }}\delta_{MM^{\prime }}\delta_{\Sigma \Sigma^{\prime }}\delta_{\sigma \sigma^{\prime }}\left(\omega_{N}\sigma +\frac{iL\left(L+1\right)}{6\tau_{Q}}\right) \end{array}$$

where $F_{0}^{2}=1$, $F_{\pm 1}^{2}=0$, $F_{\pm 2}^{2}==\frac{\eta }{\sqrt{6}}$, $\omega_{N}$ denotes the ^14^N resonance frequency. The matrix $\left[T_{1}^{1}\right]$ is a representation of the $T_{1}^{1}$ operators ($T_{0}^{1}=S_{z}$, $T_{\pm 1}^{1}=\mp S_{\pm }/\sqrt{2}$) in the basis $\left\{|O_{\alpha })\right\}$ and it contains only three non-zero elements associated with the functions: |1,−1)⊗|2,0,2), suppl, and |1,1)⊗|2,0,0) equal to 1/5, 1/10, and 1/30, respectively.

**Figure 1:**
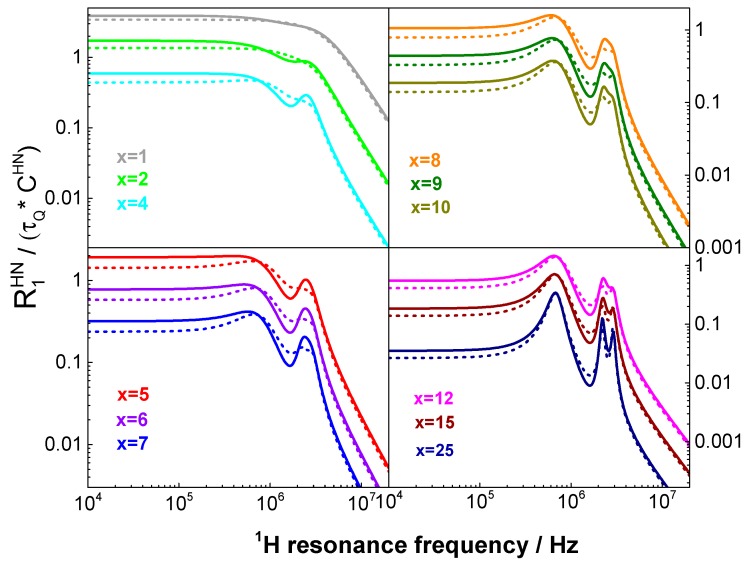
Simulated ^1^H–^14^N spin–lattice relaxation contribution: comparison between the predictions of the SLE approach (solid lines) and of Equation (4) (dashed lines) depending on the value of the product: $x=\omega_{Q}\tau_{Q}$. For clarity of the figure, the simulation results have been divided by a factor of 2 for *x* = 2,6,9,15 and by a factor of 4 for *x* = 4,7,10,25. $C_{}^{HN}=\frac{2}{3}{\left(\frac{\mu_{0}}{4\pi }\frac{\gamma_{H}\gamma_{N}\hbar }{r_{HN}^{3}}\right)}^{2}$, Θ=Φ=0. The values of $\tau_{Q}$ are 4.68 × 10^−8^ s^−1^ (*x* = 1); 9.36 × 10^−8^s^−1^ (*x* = 2), 1.17 × 10^−6^ s^−1^ (*x* = 25).

To avoid misinterpretations of QRE effects and, in consequence, of the underlying dynamical processes, it is very important to thoroughly compare the two descriptions in order to explicitly reveal the validity range of Equation (4). In Figure 1, a comparison is shown between the predictions of the SLE approach and the predictions of Equation (4), depending on the value of the product $x=\omega_{Q}\tau_{Q}$. The selected parameters: $a_{Q}=$ 3.4 MHz and η=0.4 are relatively close to the values characteristic of ^14^N of amide groups in protein side chains [30]. One can conclude that the expression of Equation (4) is in a good agreement with the SLE approach for x≤1, then the discrepancies increase and, in the range of 1<x<25, Equation (4) breaks down. Eventually, for x≥25, it can again be used, as for such slow dynamics, the quadrupole coupling can be treated as time independent, hence, constituting the energy level structure of ^14^N.

### 2.3. Experimental Details

Bovine serum albumin (BSA), albumin from human plasma (AHP), elastin from bovine neck ligament, and lysozyme from hen egg whites were purchased from Sigma–Aldrich in the form of lyophilized powder. The first three proteins are globular, while elastin is a fibrillar protein [53,54]. Molecular weights of BSA and AHP are similar: 66.4 kDa [55] and 66.5 kDa [56], respectively. They also contain similar numbers of amino acids: 583 [57] and 585 [56], respectively. Despite those similarities, the secondary structure of BSA and AHP differs. The helical content of BSA reaches 53%, 14% of BSA structure forms β-sheets, 4% forms β-turns, and 16% is random [58]. On the other hand, about 60% of AHP’s structure is α-helix, 30% of AHP forms β-strands, and the remaining 10% forms turns [59]. The molecular weight of lysozyme is 13.9 kDa; the α-helical content ranges from 26%–31% and the β structure content varies between 11–16% [60,61]. The elastin monomer (tropoelastin) weighs about 70 kDa, the helical content of elastin is about 10%, while about 35% of the structure forms β-strands [62,63].

The ^1^H spin–lattice relaxation experiments for these proteins have been performed at 308 K (controlled with an accuracy of 0.5 K) in a frequency range from approximately 10 kHz (the limit depends on the specific protein) up to 40 MHz (referring to ^1^H resonance frequency) using an FFC relaxometer (“Spinmaster 2000”, Stelar S.r.l., Mede, Italy). The duration of the radio-frequency pulse was 8 µs, the detection frequency was 15.8 MHz, the slew rate of the magnetic field was 12 MHz/ms, and the repetition delay time was five times larger than the spin–lattice relaxation time at the highest magnetic field. The relaxation process was observed to be single exponential for all proteins over the whole frequency range.

## 3. Results and Discussion

**Figure 2:**
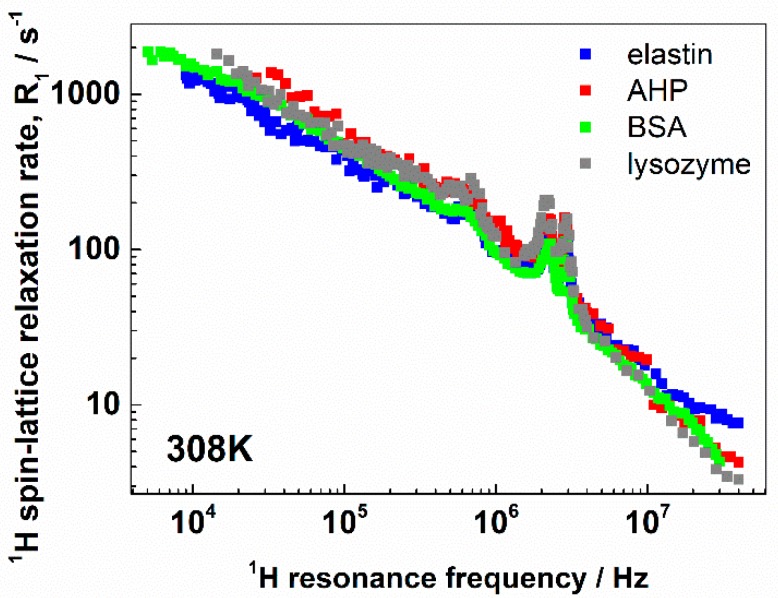
^1^H spin–lattice relaxation profiles for elastin, albumin from human plasma (AHP), bovine serum albumin (BSA), and lysozyme at 308 K.

Figure 2 shows a comparison of the ^1^H spin–lattice relaxation profiles for elastin, AHP, BSA, and lysozyme collected at 308 K (the data are included into Supplementary Materials).

Two observations can immediately be made. The first one is that in all cases, the QRE effects were well pronounced and the frequency positions of the quadrupole peaks were similar. The second observation is that the overall shapes of the relaxation profiles were similar, although the protein structures were significantly different. The data were analyzed in terms of Equation (1), where the $R_{1}^{HH}\left(\omega_{H}\right)$ and $R_{1}^{HN}\left(\omega_{H}\right)$ relaxation terms are given by Equation (3) and Equation (4), respectively. The quadrupole parameters, $a_{Q}$ and η, were determined from the positions of the quadrupole peaks. They are given in Table 1.

**Figure 3:**
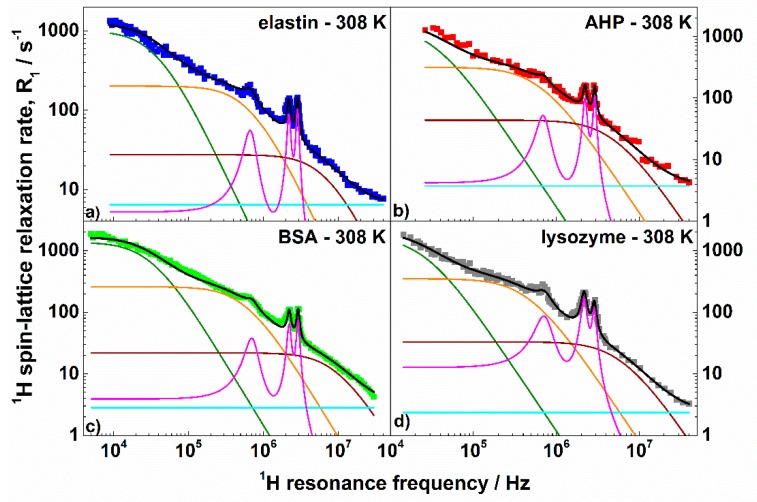
^1^H spin–lattice relaxation rates, $R_{1}^{HH}$, for: (**a**) elastin, (**b**) AHP, (**c**) BSA, and (**d**) lysozyme in the solid state at 308 K. Black lines are theoretical fits decomposed into the individual relaxation contributions: $R_{1,s}^{HH}$ (green lines), $R_{1,i}^{HH}$ (orange lines), $R_{1,f}^{HH}$ (brown lines), A (light blue lines), and $R_{1}^{HN}$ (magenta lines).

Nevertheless, the model includes ten adjustable parameters—the ^1^H–^1^H dipolar relaxation constants and the corresponding correlation times $C_{s}^{HH},\tau_{s},C_{i}^{HH},\tau_{i},C_{f}^{HH},\tau_{f}$, A, the ^1^H–^14^N dipolar relaxation constant, and the corresponding correlation time $C_{}^{HN}$, $\tau_{Q}$, and, finally, the angles Θ and Φ. The number of the adjustable parameters can, of course, raise doubts concerning the ambiguity of the analysis. The parameters are, however, responsible for different features of the relaxation profiles. Before addressing this subject in more detail, let us discuss the obtained values. They are collected in Table 1, while the results of the analysis are graphically represented in Figure 3.

**Figure 4:**
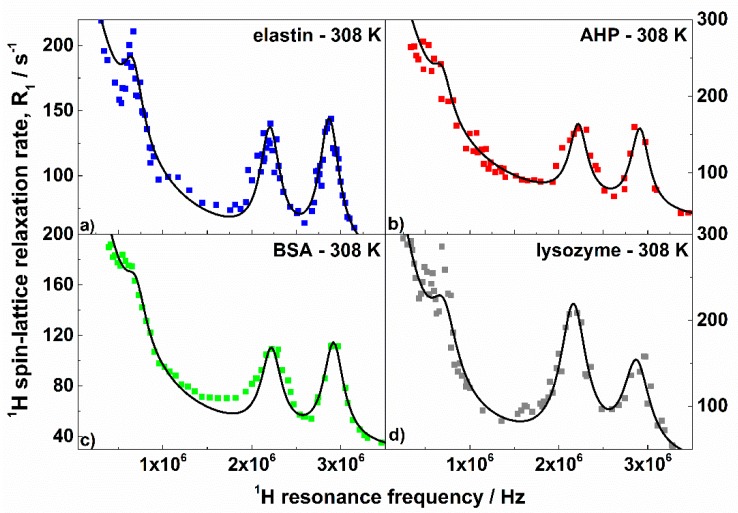
^1^H spin–lattice relaxation rates, $R_{1}^{HH}$, for: (**a**) elastin, (**b**) AHP, (**c**) BSA, and (**d**) lysozyme in the solid state at 308 K in the frequency ranges in which the quadrupole peaks are present. The black lines are theoretical fits.

As already anticipated, $a_{Q}$ and η were directly obtained from the positions of the quadrupole peaks and then fixed for the fitting. The parameters were very close as expected from the similar positions of the quadrupole peaks; $a_{Q}$ varied between 3.36 MHz and 3.43 MHz, while η was in the range 0.39–0.42. The values show that, independently of the structure of the proteins, the electric field gradient tensor at ^14^N nuclei is similar. The correlation time, $\tau_{Q}$, characterizing the fluctuations of the ^1^H–^14^N dipole–dipole coupling determines the widths of the quadrupole peaks and, therefore, it also can be (almost) independently obtained. The values calculated from the widths of the quadrupole peaks were used as initial parameters and only slightly (not more than 10%) adjusted in the course of the fitting. Figure 4 illustrates the high accuracy with which the shapes and the quadrupole peaks were reproduced. The $\tau_{Q}$ values varied between 8.9 × 10^−7^ s and 1.27 × 10^−6^ s. As far as the dynamics are concerned, the correlation times $\tau_{s},\tau_{i}$, and $\tau_{f}$ were of the order of 10^−6^ s, 10^−7^ s, and 10^−8^ s, respectively; $\tau_{s}$ was about (3–4) × 10^−6^ s, while $\tau_{i}$ was approximately (1–2) × 10^−7^ s. The corresponding dipolar relaxation constants varied between (5–9) × 10^7^ Hz^2^ for the slow process and (3–4) × 10^8^ Hz^2^ for the intermediate one. The correlation times might correspond to global motions of whole protein domains leading to conformational changes [64]. One should, however, clearly stress that this statement is hypothetical. It is worth noting that processes on similar time scales have been observed by means of dielectric spectroscopy for dry lysozymes [65]. The dynamics were attributed to structural relaxation of an “unclear” mechanism. One can also interpret the obtained values of the $\tau_{s},\tau_{i},C_{s}^{HH},C_{i}^{HH}$ parameters as a fingerprint of a distribution of the correlation times characterizing the dynamics of protein domains. One can conclude that the time scale of the dynamics ranged between (3–4) × 10^−6^ s and (1–2) × 10^−7^ s and the center of the distribution was shifted towards the shorter correlation times (because $C_{i}^{HH}$ was larger than $C_{s}^{HH}$). As far as the fast dynamics are concerned, the correlation time $\tau_{f}$ ranges between (1.0–2.5) × 10^−8^ s while the corresponding dipolar relaxation constant yielded about (4–5) × 10^8^ Hz^2^. This motional process can be associated with the dynamics of structural elements, like α-helices [66]. The frequency independent relaxation contribution, A, likely stemmed from dynamics of molecular fragments occurring on a timescale shorter than 10^−9^ s. For elastin, the parameter was larger than for the other proteins. This can directly be seen from the high-frequency relaxation data (Figure 2). The products $x=\omega_{Q}\tau_{Q}$ are of the order of 80–100, which implies that Equation (4) can be applied to describe the ^1^H–^14^N relaxation contribution. There are two conceivable reasons why $\tau_{Q}<\tau_{s}$ (the ratio $\tau_{s}/\tau_{Q}$ yields about 3–4). The first one is the possible distribution of the correlation times including also somewhat faster dynamics—then $\tau_{Q}$ can be treated as an “effective” parameter. The second one is the ^14^N relaxation caused by local fluctuations of the electric field gradient. The relaxation contributes to the fluctuations of the ^1^H–^14^N dipole–dipole coupling, shortening the “effective” correlation time. For the intermediate dynamics one gets $\omega_{Q}\tau_{i}\approx 2$. The simulations shown in Figure 1 indicate that for this value, the QRE effects are only weakly pronounced, moreover, the ^1^H–^14^N relaxation contribution associated with the intermediate dynamics was anyway masked by the corresponding ^1^H–^1^H contribution, as the ^14^N gyromagnetic factor was relatively low. The last statement also applies to the ^1^H–^14^N relaxation contribution associated with the fast dynamics. The value of the amplitude of the quadrupole interaction describes a residual quadrupolar coupling which remains after a partial averaging of the electric field gradient tensor due to the fast-dynamical processes. The dipolar relaxation constant $C_{}^{HN}$ leads to effective ^1^H–^14^N inter-spin distances, $r_{HN}$, of 1.64 Å (elastin, AHP), 1.71 Å (BSA), and 1.47 Å (lysozyme), while the ^1^H–^14^N bound length in the amide groups was determined as being approximately 1 Å [67]. One should, however, take into account that the relaxation model treats the QRE effects as originating from a system including one ^1^H and one ^14^N nuclei, because one can hardly consider a many spin model characterized by numerous (unknown) parameters. However, there were longer-range dipole–dipole couplings between non-bonded ^1^H and ^14^N nuclei contributing to the effect. Moreover, the proportion between the number of involved ^1^H and ^14^N nuclei was not 1:1. Moreover, the ^1^H–^14^N dipole–dipole interactions can also be partially reduced due to the local fast dynamics.

Finally, we should turn our attention to the Θ,Φ angles describing the orientation of the ^1^H–^14^N dipole–dipole axis with respect to the principal axis system of the electric field gradient tensor at the ^14^N position. The parameters are responsible for the relative intensities of the quadrupole peaks, as can be easily determined from Equation (4). A closer inspection of Figure 4 shows, however, that the relative intensities of the quadrupole peaks for different proteins indeed differ considerably. For instance, for elastin and BSA the amplitude of the peak at $\nu_{+}$ was higher than at $\nu_{-}$; for AHP, the amplitudes were similar; while for lysozyme, the peak at $\nu_{+}$ was smaller than at $\nu_{-}$. This effect was reflected by the angle Φ being smaller for lysozyme than for the other proteins.

Finishing this discussion, we wish to stress that since the parameters involved in the analysis determined different features of the ^1^H spin–lattice relaxation profiles, therefore we have been able to determine them unambiguously, despite their relatively high number. Subtle differences in the parameters might be, to some extent, caused by slight “wetness” of the solid proteins [68].

## 4. Conclusions

The ^1^H spin–lattice relaxation studies were performed for solid bovine serum albumin, albumin from human plasma, elastin from bovine neck ligament, and lysozyme from hen egg whites at 308 K in the frequency range between 10 kHz (5 kHz for BSA) and 40 MHz. A thorough analysis of the relaxation results provided a rich set of information. Three relaxation processes were revealed to be associated with dynamics occurring on the time scales 10^−6^ s, 10^−7^ s, and 10^−8^ s, referred to, respectively, as slow, intermediate, and fast dynamics. The motion of the time scale 10^−6^ s–10^−7^ s was interpreted as dynamics of whole protein domains leading to conformational changes, while the motion on the timescale 10^−8^ s could be attributed to the dynamics of structural elements such as, for instance, α-helices. As far as the dynamics of large protein domains is concerned, one can interpret the findings as a distribution of correlation times spanning the range of 10^−6^ s–10^−7^ s and shifted towards the lower limit. This shift was reflected by the relationship among the corresponding dipolar relaxation constants; the relaxation constant for the intermediate dynamics (10^−7^ s) was larger than for the slow dynamics (10^−6^ s). In addition, a frequency-independent relaxation contribution associated with motion faster than that of 10^−9^s timescale has been revealed. This contribution is more pronounced for elastin than for the other proteins. The relaxation data for all proteins showed distinct QRE effects. From the position of the quadrupole peaks, the amplitude and the asymmetry parameter of the ^14^N quadrupole coupling was determined. The parameters were very close for all proteins: the quadrupole coupling constant ranged between 3.36 MHz and 3.43 MHz, while η was within the range 0.39–0.42. The correlation time characterizing the fluctuations of the ^1^H–^14^N dipole–dipole was by a factor 3–4 shorter than the correlation time associated with the slow dynamics. The shortening may reflect the possible distribution of the correlation times including also faster dynamics as well as a contribution of the ^14^N relaxation to the effective fluctuations of the ^1^H–^14^N dipole–dipole interactions. The ^1^H–^14^N inter-spin distance, $r_{HN}$, turned out to be longer (1.47–1.71 Å) compared to the ^1^H–^14^N bound length in the amide groups (1 Å) when assuming a single pair, ^1^H–^14^N QRE model. However, there were longer-range dipole–dipole couplings between non-bonded ^1^H and ^14^N nuclei contributing to the effect, and the proportion between the number of involved ^1^H and ^14^N nuclei was not 1:1. Moreover, fast local dynamics led to a partial averaging of the dipolar coupling that manifested itself as an increase of the effective inter-spin distance. The analysis also allowed one to determine the orientation of the principal axis system of the electric field gradient tensor at the ^14^N position with respect to the ^1^H–^14^N dipole–dipole axis, being a fingerprint of the local configurations of the molecules.

As the QRE effects are a source of unique information on the molecular (atomistic) arrangement and dynamics in the vicinity of ^14^N nuclei, their proper theoretical modelling is of high importance. It has been shown that for the intermediate timescale of molecular motion (one can estimate it as 5 × 10^−8^ s–1 × 10^−6^ s), one should not rely on the perturbation description of QRE, but the general approach based on SLE should be applied.

## Figures and Tables

**Table 1:** Parameters obtained from the analysis of the ^1^H spin–lattice relaxation data; * non-adjustable parameters. The uncertainty level of the correlation times is provided in the parentheses. For the relaxation constants, the level is in the range of 5–8%.

Parameter	Elastin	AHP	BSA	Lysozyme
$C_{s}^{HH}$/Hz^2^	7.85 × 10^7^	9.91 × 10^7^	8.95 × 10^7^	8.89 × 10^7^
$\tau_{s}$/s	2.55 × 10^−6^ (2%)	2.93 × 10^−6^ (8%)	3.06 × 10^−6^ (2%)	3.79 × 10^−6^ (7%)
$C_{i}^{HH}$/Hz^2^	2.84 × 10^8^	4.16 × 10^8^	3.01 × 10^8^	3.36 × 10^8^
$\tau_{i}$/s	1.43 × 10^−7^ (5%)	1.52 × 10^−7^ (9%)	1.72 × 10^−7^ (3%)	2.09 × 10^−7^ (5%)
$C_{f}^{HH}$/Hz^2^	4.21 × 10^8^	4.52 × 10^8^	4.37 × 10^8^	4.24 × 10^8^
$\tau_{f}$/s	1.31 × 10^−8^ (12%)	1.94 × 10^−8^ (11%)	1.10 × 10^−8^ (5%)	1.56 × 10^−8^ (7%)
A/s^−1^	6.47	3.73	3.04	2.36
* $a_{Q}$/MHz	3.38	3.42	3.43	3.36
* η	0.39	0.40	0.41	0.42
$\tau_{Q}$/s	1.19 × 10^−6^ (8%)	1.27 × 10^−6^ (12%)	1.16 × 10^−6^ (7%)	8.91 × 10^−7^ (8%)
$C_{}^{HN}$/Hz^2^	1.01 × 10^8^	1.01 × 10^8^	7.81 × 10^7^	1.93 × 10^8^
Θ/^o^	69	75	72	68
Φ/^o^	50	47	49	33
relative error (%)	6.9	10.1	6.1	7.4

## Supplementary Materials

The following are available online at https://www.mdpi.com/2218-273X/9/11/652/s1. Table S1: ^1^H magnetization of elastin, Table S2: ^1^H magnetization of AHP, Table S3: ^1^H magnetization of BSA, Table S4: ^1^H magnetization of lysozyme.
