# Supplementary material for: Dynamics of Solid Proteins by Means of Nuclear Magnetic Resonance Relaxometry
Source: Biomolecules. 2019 Oct 25;9(11):652. doi: 10.3390/biom9110652 (PMC6920843; doi:10.3390/biom9110652)
Supplement: Supplementary file 1 [file biomolecules-09-00652-s001.pdf]

SUPPLEMENTARY MATERIALS

Dynamics of solid proteins by means of Nuclear Magnetic Resonance relaxometry

Danuta Kruk[1,*], Anna Borkowska[1], Elzbieta Masiewicz[1], Pawel Rochowski[1,#], Pascal H. Fries[2], Lionel M. Broche[3] and David J. Lurie[3]

[1] Faculty of Mathematics and Computer Science, University of Warmia & Mazury in Olsztyn, Słoneczna 54, 10-710 Olsztyn, Poland; danuta.kruk@matman.uwm.edu.pl, elzbieta.masiewicz@matman.uwm.edu.pl, a.borkowska@uwm.edu.pl, proch@matman.uwm.edu.pl.

[2] Laboratoire de Reconnaissance Ionique et Chimie de Coordination, Service de Chimie Inorganique et Biologique (UMR E-3 CEA/UJF), CEA-Grenoble, INAC, 17 rue des Martyrs, 38054 Grenoble cedex 9, France; pascal-h.fries@cea.fr

[3] Bio-Medical Physics, School of Medicine, Medical Sciences & Nutrition, University of Aberdeen, Foresterhill, Aberdeen AB25 2ZD, Scotland, United Kingdom ; l.broche@abdn.ac.uk, d.lurie@abdn.ac.uk.

# Current affiliation of Pawel Rochowski:
Faculty of Mathematics, Physics and Informatics, Gdansk University, Wita Stwosza 57, 80-308 Gdansk, Poland

* Correspondence: danuta.kruk@matman.uwm.edu.pl; Tel.: +48 89 524 60 11

1. ^{1}H magnetization of elastin:

FREQUENCY [Hz]: 9025.7		9176.2		9484.7		9800.8	
TAU [s]	MAGNITUDES [a.u]	TAU [s]	MAGNITUDES [a.u]	TAU [s]	MAGNITUDES [a.u]	TAU [s]	MAGNITUDES [a.u]
3.3986e-03	3.8059e+02	3.9505e-03	3.9646e+02	3.1708e-03	3.5411e+02	3.2623e-03	3.1001e+02
3.1726e-03	3.7220e+02	2.6496e-03	2.8921e+02	2.9600e-03	3.4804e+02	3.0453e-03	3.0136e+02
2.9466e-03	3.5156e+02	1.7771e-03	4.1858e+02	2.7491e-03	3.2054e+02	2.8284e-03	4.6144e+02
2.7206e-03	4.4054e+02	1.1919e-03	6.8785e+02	2.5382e-03	3.3897e+02	2.6115e-03	3.3416e+02
2.4945e-03	3.6390e+02	7.9942e-04	6.8628e+02	2.3274e-03	4.2488e+02	2.3945e-03	3.6499e+02
2.2685e-03	3.6885e+02	5.3617e-04	8.9243e+02	2.1165e-03	4.7050e+02	2.1776e-03	4.1298e+02
2.0425e-03	4.3517e+02	3.5961e-04	1.0962e+03	1.9057e-03	3.6466e+02	1.9606e-03	4.7086e+02
1.8165e-03	4.3455e+02	2.4119e-04	1.1510e+03	1.6948e-03	4.2526e+02	1.7437e-03	4.5370e+02
1.5905e-03	4.8098e+02	1.6177e-04	1.2801e+03	1.4839e-03	4.7733e+02	1.5267e-03	5.0235e+02
1.3645e-03	5.1865e+02	1.0850e-04	1.3071e+03	1.2731e-03	6.1063e+02	1.3098e-03	5.5303e+02
1.1385e-03	6.0729e+02	7.2770e-05	1.4474e+03	1.0622e-03	6.0150e+02	1.0929e-03	5.5036e+02
9.1251e-04	7.3623e+02	4.8807e-05	1.4115e+03	8.5136e-04	8.2837e+02	8.7592e-04	7.6168e+02
6.8651e-04	8.2997e+02	3.2735e-05	1.4539e+03	6.4050e-04	8.1643e+02	6.5898e-04	8.6307e+02
4.6051e-04	1.0094e+03	2.1955e-05	1.4527e+03	4.2964e-04	1.0644e+03	4.4204e-04	9.8381e+02
2.3450e-04	1.2272e+03	1.4725e-05	1.4219e+03	2.1879e-04	1.1830e+03	2.2510e-04	1.2039e+03
8.4964e-06	1.5317e+03	9.8764e-06	1.4286e+03	7.9270e-06	1.4906e+03	8.1557e-06	1.5077e+03

FREQUENCY [Hz]: 10280		10772		11616		12136	
TAU [s]	MAGNITUDES [a.u]	TAU [s]	MAGNITUDES [a.u]	TAU [s]	MAGNITUDES [a.u]	TAU [s]	MAGNITUDES [a.u]
2.8656e-03	4.3937e+02	4.2793e-03	3.2444e+02	3.1416e-03	3.8096e+02	3.3430e-03	3.4136e+02
2.6750e-03	3.8013e+02	2.8702e-03	4.7396e+02	2.9327e-03	4.0870e+02	3.1207e-03	3.7949e+02
2.4844e-03	3.8257e+02	1.9250e-03	5.3401e+02	2.7238e-03	3.8144e+02	2.8984e-03	5.1106e+02
2.2939e-03	4.2564e+02	1.2911e-03	5.4644e+02	2.5149e-03	4.2613e+02	2.6761e-03	4.6845e+02
2.1033e-03	4.4081e+02	8.6595e-04	7.0171e+02	2.3060e-03	3.4985e+02	2.4538e-03	4.5607e+02
1.9128e-03	3.9784e+02	5.8080e-04	8.5837e+02	2.0970e-03	3.6025e+02	2.2314e-03	4.6075e+02
1.7222e-03	5.3249e+02	3.8954e-04	1.0828e+03	1.8881e-03	5.0896e+02	2.0091e-03	3.6483e+02
1.5316e-03	5.4244e+02	2.6127e-04	1.2851e+03	1.6792e-03	4.0520e+02	1.7868e-03	4.9826e+02
1.3411e-03	5.5240e+02	1.7523e-04	1.3554e+03	1.4703e-03	5.4194e+02	1.5645e-03	4.8795e+02
1.1505e-03	6.3926e+02	1.1753e-04	1.3772e+03	1.2614e-03	5.7140e+02	1.3422e-03	6.0823e+02
9.5996e-04	7.4816e+02	7.8826e-05	1.4472e+03	1.0525e-03	6.9279e+02	1.1199e-03	6.1838e+02
7.6940e-04	7.9994e+02	5.2869e-05	1.3957e+03	8.4353e-04	8.3033e+02	8.9759e-04	7.3032e+02
5.7884e-04	9.7760e+02	3.5459e-05	1.5515e+03	6.3461e-04	8.2673e+02	6.7528e-04	9.4636e+02
3.8828e-04	1.0713e+03	2.3783e-05	1.5206e+03	4.2569e-04	1.1310e+03	4.5297e-04	1.1586e+03
1.9772e-04	1.3396e+03	1.5951e-05	1.4802e+03	2.1677e-04	1.2389e+03	2.3067e-04	1.2598e+03
7.1639e-06	1.5325e+03	1.0698e-05	1.4717e+03	7.8541e-06	1.5583e+03	8.3575e-06	1.5637e+03

FREQUENCY [Hz]: 12672		13215		13959		14339	
TAU [s]	MAGNITUDES [a.u]	TAU [s]	MAGNITUDES [a.u]	TAU [s]	MAGNITUDES [a.u]	TAU [s]	MAGNITUDES [a.u]
3.4260e-03	3.1906e+02	4.3388e-03	3.3504e+02	4.2464e-03	4.0852e+02	3.9665e-03	3.4495e+02
3.1981e-03	4.4160e+02	4.0503e-03	3.4734e+02	2.8480e-03	4.1814e+02	3.7027e-03	4.2974e+02
2.9703e-03	3.8827e+02	3.7618e-03	3.8950e+02	1.9102e-03	4.4583e+02	3.4389e-03	4.2347e+02
2.7425e-03	5.0217e+02	3.4732e-03	3.6960e+02	1.2812e-03	6.5199e+02	3.1752e-03	3.9548e+02
2.5147e-03	3.7949e+02	3.1847e-03	4.1460e+02	8.5928e-04	8.0021e+02	2.9114e-03	3.8172e+02
2.2868e-03	4.3222e+02	2.8962e-03	3.6453e+02	5.7632e-04	1.0923e+03	2.6476e-03	3.9306e+02
2.0590e-03	4.2344e+02	2.6076e-03	4.6095e+02	3.8654e-04	1.2459e+03	2.3839e-03	4.6291e+02
1.8312e-03	5.5679e+02	2.3191e-03	3.6419e+02	2.5925e-04	1.2675e+03	2.1201e-03	4.0522e+02
1.6034e-03	5.0638e+02	2.0306e-03	4.1945e+02	1.7388e-04	1.2499e+03	1.8563e-03	5.0897e+02
1.3755e-03	6.7994e+02	1.7420e-03	4.3594e+02	1.1662e-04	1.4477e+03	1.5925e-03	5.2556e+02
1.1477e-03	6.6405e+02	1.4535e-03	4.1508e+02	7.8219e-05	1.4756e+03	1.3288e-03	5.5323e+02
9.1987e-04	7.7588e+02	1.1650e-03	7.0423e+02	5.2462e-05	1.5776e+03	1.0650e-03	7.4593e+02
6.9205e-04	8.5249e+02	8.7644e-04	8.8843e+02	3.5186e-05	1.4684e+03	8.0123e-04	8.9338e+02
4.6422e-04	1.1501e+03	5.8791e-04	1.0524e+03	2.3599e-05	1.5268e+03	5.3746e-04	1.1072e+03
2.3639e-04	1.3113e+03	2.9938e-04	1.2616e+03	1.5828e-05	1.5061e+03	2.7369e-04	1.1933e+03
8.5649e-06	1.6102e+03	1.0847e-05	1.5817e+03	1.0616e-05	1.5294e+03	9.9162e-06	1.5859e+03

FREQUENCY [Hz]: 14921		15117		15509		16935	
TAU [s]	MAGNITUDES [a.u]	TAU [s]	MAGNITUDES [a.u]	TAU [s]	MAGNITUDES [a.u]	TAU [s]	MAGNITUDES [a.u]
3.6976e-03	4.1383e+02	5.5739e-03	3.6692e+02	3.2976e-03	5.7092e+02	4.3148e-03	3.8525e+02
3.4517e-03	3.6992e+02	3.7384e-03	2.9510e+02	3.0783e-03	3.6165e+02	4.0279e-03	3.2887e+02
3.2058e-03	4.5331e+02	2.5074e-03	3.7250e+02	2.8590e-03	4.0928e+02	3.7409e-03	3.5056e+02
2.9599e-03	3.7490e+02	1.6817e-03	4.6787e+02	2.6397e-03	3.8188e+02	3.4540e-03	3.9933e+02
2.7140e-03	3.6043e+02	1.1279e-03	6.9056e+02	2.4204e-03	3.7467e+02	3.1671e-03	3.7882e+02
2.4682e-03	4.2759e+02	7.5650e-04	1.0166e+03	2.2011e-03	5.1227e+02	2.8801e-03	4.2044e+02
2.2223e-03	3.9484e+02	5.0738e-04	1.1465e+03	1.9818e-03	4.6669e+02	2.5932e-03	4.0990e+02
1.9764e-03	5.3794e+02	3.4030e-04	1.2165e+03	1.7626e-03	5.9569e+02	2.3063e-03	4.4913e+02
1.7305e-03	5.6601e+02	2.2824e-04	1.4550e+03	1.5433e-03	6.6533e+02	2.0193e-03	4.7329e+02
1.4846e-03	6.3568e+02	1.5308e-04	1.3966e+03	1.3240e-03	6.7548e+02	1.7324e-03	5.8469e+02
1.2387e-03	7.1909e+02	1.0267e-04	1.4293e+03	1.1047e-03	7.5635e+02	1.4455e-03	6.0763e+02
9.9281e-04	8.3068e+02	6.8863e-05	1.5439e+03	8.8540e-04	9.2068e+02	1.1585e-03	6.4402e+02
7.4692e-04	9.3433e+02	4.6186e-05	1.4713e+03	6.6611e-04	9.6184e+02	8.7159e-04	9.2736e+02
5.0103e-04	1.1802e+03	3.0977e-05	1.5667e+03	4.4682e-04	1.2443e+03	5.8466e-04	1.0820e+03
2.5514e-04	1.3382e+03	2.0776e-05	1.4681e+03	2.2753e-04	1.2666e+03	2.9772e-04	1.4211e+03
9.2440e-06	1.6306e+03	1.3935e-05	1.6696e+03	8.2439e-06	1.6991e+03	1.0787e-05	1.7232e+03

FREQUENCY [Hz]: 17562		19744		20652		21578	
TAU [s]	MAGNITUDES [a.u]	TAU [s]	MAGNITUDES [a.u]	TAU [s]	MAGNITUDES [a.u]	TAU [s]	MAGNITUDES [a.u]
5.4518e-03	3.8727e+02	4.1874e-03	4.7573e+02	4.2594e-03	3.3443e+02	5.3928e-03	4.0933e+02
5.0893e-03	3.9245e+02	3.9089e-03	4.2221e+02	3.9762e-03	2.8842e+02	5.0342e-03	3.0287e+02
4.7267e-03	3.2388e+02	3.6304e-03	3.9814e+02	3.6929e-03	4.5644e+02	4.6756e-03	3.4780e+02
4.3642e-03	3.4133e+02	3.3520e-03	4.2740e+02	3.4097e-03	4.1025e+02	4.3170e-03	3.2634e+02
4.0016e-03	4.4099e+02	3.0735e-03	4.1168e+02	3.1264e-03	4.0465e+02	3.9584e-03	3.7697e+02
3.6391e-03	4.2910e+02	2.7951e-03	4.7007e+02	2.8432e-03	3.7003e+02	3.5997e-03	4.3906e+02
3.2765e-03	4.4071e+02	2.5166e-03	4.5151e+02	2.5599e-03	5.4246e+02	3.2411e-03	4.1354e+02
2.9140e-03	2.9828e+02	2.2381e-03	6.3053e+02	2.2767e-03	4.9696e+02	2.8825e-03	4.4207e+02
2.5515e-03	3.8481e+02	1.9597e-03	5.1554e+02	1.9934e-03	5.1465e+02	2.5239e-03	5.9866e+02
2.1889e-03	4.8502e+02	1.6812e-03	6.2433e+02	1.7102e-03	5.3167e+02	2.1652e-03	3.9482e+02
1.8264e-03	6.3758e+02	1.4028e-03	6.7718e+02	1.4269e-03	7.1592e+02	1.8066e-03	5.8811e+02
1.4638e-03	6.6323e+02	1.1243e-03	7.6922e+02	1.1437e-03	7.6239e+02	1.4480e-03	6.8324e+02
1.1013e-03	8.6330e+02	8.4585e-04	9.3664e+02	8.6041e-04	9.3991e+02	1.0894e-03	7.9535e+02
7.3872e-04	1.0070e+03	5.6739e-04	1.1133e+03	5.7715e-04	1.1296e+03	7.3073e-04	1.1310e+03
3.7618e-04	1.2330e+03	2.8893e-04	1.3838e+03	2.9390e-04	1.4418e+03	3.7211e-04	1.3418e+03
1.3630e-05	1.6062e+03	1.0468e-05	1.6192e+03	1.0649e-05	1.6656e+03	1.3482e-05	1.7003e+03

FREQUENCY [Hz]: 23011		23495		24484		24989	
TAU [s]	MAGNITUDES [a.u]	TAU [s]	MAGNITUDES [a.u]	TAU [s]	MAGNITUDES [a.u]	TAU [s]	MAGNITUDES [a.u]
5.0833e-03	4.1538e+02	5.1005e-03	4.0181e+02	4.3425e-03	4.3994e+02	5.5165e-03	3.7304e+02
3.4094e-03	4.6714e+02	4.7613e-03	3.6309e+02	4.0537e-03	4.1321e+02	3.6999e-03	3.9497e+02
2.2867e-03	4.6682e+02	4.4221e-03	3.9867e+02	3.7650e-03	4.1983e+02	2.4815e-03	4.7709e+02
1.5337e-03	7.1812e+02	4.0829e-03	4.2980e+02	3.4762e-03	4.9409e+02	1.6644e-03	6.7904e+02
1.0286e-03	8.9382e+02	3.7438e-03	3.8316e+02	3.1874e-03	3.6805e+02	1.1163e-03	9.0803e+02
6.8991e-04	1.1316e+03	3.4046e-03	3.9199e+02	2.8986e-03	4.7489e+02	7.4870e-04	1.1377e+03
4.6273e-04	1.2614e+03	3.0654e-03	4.9646e+02	2.6099e-03	4.0301e+02	5.0216e-04	1.3866e+03
3.1035e-04	1.4071e+03	2.7262e-03	5.8016e+02	2.3211e-03	6.4596e+02	3.3680e-04	1.2835e+03
2.0815e-04	1.5089e+03	2.3870e-03	4.7576e+02	2.0323e-03	6.3912e+02	2.2589e-04	1.5410e+03
1.3961e-04	1.6430e+03	2.0478e-03	5.7248e+02	1.7435e-03	6.8536e+02	1.5150e-04	1.6506e+03
9.3636e-05	1.6875e+03	1.7087e-03	6.7290e+02	1.4547e-03	8.5812e+02	1.0161e-04	1.5909e+03
6.2802e-05	1.6707e+03	1.3695e-03	7.9533e+02	1.1660e-03	9.0074e+02	6.8153e-05	1.6771e+03
4.2121e-05	1.7042e+03	1.0303e-03	9.4702e+02	8.7719e-04	1.0988e+03	4.5710e-05	1.6442e+03
2.8251e-05	1.6741e+03	6.9112e-04	9.0999e+02	5.8841e-04	1.2628e+03	3.0658e-05	1.6824e+03
1.8948e-05	1.5918e+03	3.5193e-04	1.3338e+03	2.9963e-04	1.5000e+03	2.0562e-05	1.8330e+03
1.2708e-05	1.7317e+03	1.2751e-05	1.6434e+03	1.0856e-05	1.7670e+03	1.3791e-05	1.6440e+03

FREQUENCY [Hz]: 25494		26524		27312		27577	
TAU [s]	MAGNITUDES [a.u]	TAU [s]	MAGNITUDES [a.u]	TAU [s]	MAGNITUDES [a.u]	TAU [s]	MAGNITUDES [a.u]
5.1356e-03	3.8683e+02	5.4795e-03	3.6776e+02	6.0240e-03	3.2122e+02	5.0758e-03	4.1770e+02
4.7941e-03	3.6894e+02	5.1151e-03	3.6867e+02	4.0403e-03	3.8542e+02	4.7382e-03	3.5841e+02
4.4526e-03	4.5112e+02	4.7507e-03	4.3889e+02	2.7098e-03	5.0614e+02	4.4007e-03	3.3961e+02
4.1111e-03	3.7002e+02	4.3863e-03	3.3911e+02	1.8175e-03	6.4468e+02	4.0632e-03	3.8754e+02
3.7696e-03	4.3035e+02	4.0219e-03	4.1051e+02	1.2190e-03	9.4386e+02	3.7256e-03	4.4448e+02
3.4280e-03	4.3299e+02	3.6575e-03	4.0548e+02	8.1758e-04	1.1476e+03	3.3881e-03	4.2875e+02
3.0865e-03	4.8093e+02	3.2932e-03	4.0290e+02	5.4835e-04	1.3608e+03	3.0505e-03	4.5152e+02
2.7450e-03	5.0536e+02	2.9288e-03	4.5711e+02	3.6778e-04	1.4893e+03	2.7130e-03	5.2123e+02
2.4035e-03	5.3099e+02	2.5644e-03	5.5183e+02	2.4667e-04	1.5680e+03	2.3755e-03	6.6757e+02
2.0620e-03	5.8132e+02	2.2000e-03	6.4515e+02	1.6544e-04	1.6033e+03	2.0379e-03	5.3428e+02
1.7204e-03	6.9836e+02	1.8356e-03	6.0189e+02	1.1096e-04	1.6716e+03	1.7004e-03	6.8631e+02
1.3789e-03	8.7460e+02	1.4712e-03	8.7190e+02	7.4423e-05	1.7622e+03	1.3628e-03	9.1824e+02
1.0374e-03	9.0461e+02	1.1069e-03	9.5895e+02	4.9916e-05	1.7655e+03	1.0253e-03	9.2631e+02
6.9588e-04	1.1110e+03	7.4247e-04	1.0445e+03	3.3479e-05	1.7710e+03	6.8777e-04	1.2811e+03
3.5436e-04	1.4495e+03	3.7808e-04	1.4197e+03	2.2454e-05	1.8263e+03	3.5023e-04	1.4789e+03
1.2839e-05	1.8512e+03	1.3699e-05	1.7295e+03	1.5060e-05	1.8011e+03	1.2689e-05	1.6975e+03

FREQUENCY [Hz]: 28918		29736		30014		32268	
TAU [s]	MAGNITUDES [a.u]	TAU [s]	MAGNITUDES [a.u]	TAU [s]	MAGNITUDES [a.u]	TAU [s]	MAGNITUDES [a.u]
5.1119e-03	3.3098e+02	6.8378e-03	3.8689e+02	8.0000e-03	3.8648e+02	6.0099e-03	3.1821e+02
4.7719e-03	3.9882e+02	4.5861e-03	4.3009e+02	7.4680e-03	4.0604e+02	4.0308e-03	3.9828e+02
4.4320e-03	3.4476e+02	3.0759e-03	5.1322e+02	6.9360e-03	4.5865e+02	2.7035e-03	6.0518e+02
4.0920e-03	5.2622e+02	2.0630e-03	6.6990e+02	6.4040e-03	4.0018e+02	1.8132e-03	8.5420e+02
3.7521e-03	4.3412e+02	1.3837e-03	9.4649e+02	5.8720e-03	3.4804e+02	1.2161e-03	9.3328e+02
3.4122e-03	5.2576e+02	9.2804e-04	1.0965e+03	5.3400e-03	3.5601e+02	8.1567e-04	1.2629e+03
3.0722e-03	3.8907e+02	6.2243e-04	1.2814e+03	4.8080e-03	3.0335e+02	5.4707e-04	1.3283e+03
2.7323e-03	4.3835e+02	4.1747e-04	1.3679e+03	4.2760e-03	4.6362e+02	3.6692e-04	1.5464e+03
2.3923e-03	6.1074e+02	2.8000e-04	1.6198e+03	3.7440e-03	4.4770e+02	2.4609e-04	1.5885e+03
2.0524e-03	6.0062e+02	1.8779e-04	1.6845e+03	3.2120e-03	4.9982e+02	1.6506e-04	1.7165e+03
1.7125e-03	6.2173e+02	1.2595e-04	1.6575e+03	2.6800e-03	5.1156e+02	1.1070e-04	1.6058e+03
1.3725e-03	8.7195e+02	8.4477e-05	1.7252e+03	2.1480e-03	6.4072e+02	7.4249e-05	1.8059e+03
1.0326e-03	1.0796e+03	5.6659e-05	1.6202e+03	1.6160e-03	7.7613e+02	4.9799e-05	1.7221e+03
6.9266e-04	1.3029e+03	3.8001e-05	1.7264e+03	1.0840e-03	1.0194e+03	3.3400e-05	1.7496e+03
3.5272e-04	1.4456e+03	2.5488e-05	1.7402e+03	5.5200e-04	1.3888e+03	2.2402e-05	1.8352e+03
1.2780e-05	1.8141e+03	1.7095e-05	1.6662e+03	2.0000e-05	1.7997e+03	1.5025e-05	1.7483e+03

FREQUENCY [Hz]: 35200		38260		41447		45101	
TAU [s]	MAGNITUDES [a.u]	TAU [s]	MAGNITUDES [a.u]	TAU [s]	MAGNITUDES [a.u]	TAU [s]	MAGNITUDES [a.u]
7.2544e-03	3.4912e+02	6.8202e-03	4.3494e+02	6.6493e-03	3.8851e+02	6.5069e-03	4.3458e+02
4.8656e-03	4.7602e+02	4.5743e-03	4.1680e+02	4.4597e-03	4.2087e+02	4.3642e-03	4.3722e+02
3.2633e-03	5.6850e+02	3.0680e-03	5.2778e+02	2.9911e-03	6.1546e+02	2.9271e-03	6.2319e+02
2.1887e-03	7.2400e+02	2.0577e-03	8.2825e+02	2.0061e-03	9.1205e+02	1.9632e-03	8.0415e+02
1.4680e-03	9.2708e+02	1.3801e-03	9.7819e+02	1.3455e-03	1.0898e+03	1.3167e-03	1.0402e+03
9.8458e-04	1.1178e+03	9.2564e-04	1.2896e+03	9.0245e-04	1.0923e+03	8.8312e-04	1.3049e+03
6.6036e-04	1.3592e+03	6.2083e-04	1.3418e+03	6.0527e-04	1.4047e+03	5.9231e-04	1.5304e+03
4.4290e-04	1.4603e+03	4.1639e-04	1.5961e+03	4.0596e-04	1.5431e+03	3.9726e-04	1.5352e+03
2.9706e-04	1.5575e+03	2.7927e-04	1.7241e+03	2.7228e-04	1.6250e+03	2.6645e-04	1.6002e+03
1.9924e-04	1.6017e+03	1.8731e-04	1.6664e+03	1.8262e-04	1.7838e+03	1.7871e-04	1.7225e+03
1.3363e-04	1.7411e+03	1.2563e-04	1.5990e+03	1.2248e-04	1.8134e+03	1.1986e-04	1.7632e+03
8.9624e-05	1.8076e+03	8.4259e-05	1.7915e+03	8.2148e-05	1.7521e+03	8.0389e-05	1.7372e+03
6.0111e-05	1.7646e+03	5.6513e-05	1.7243e+03	5.5097e-05	1.7927e+03	5.3917e-05	1.8812e+03
4.0317e-05	1.8633e+03	3.7903e-05	1.7603e+03	3.6954e-05	1.8147e+03	3.6162e-05	1.8729e+03
2.7040e-05	1.7602e+03	2.5422e-05	1.8272e+03	2.4785e-05	1.8556e+03	2.4254e-05	1.9266e+03
1.8136e-05	1.8532e+03	1.7050e-05	1.7387e+03	1.6623e-05	1.8746e+03	1.6267e-05	1.8547e+03

FREQUENCY [Hz]: 47156		48553		49259		49973	
TAU [s]	MAGNITUDES [a.u]	TAU [s]	MAGNITUDES [a.u]	TAU [s]	MAGNITUDES [a.u]	TAU [s]	MAGNITUDES [a.u]
7.1248e-03	3.2296e+02	6.3601e-03	4.4602e+02	7.9727e-03	4.7869e+02	8.0000e-03	5.9455e+02
4.7786e-03	4.9833e+02	4.2657e-03	4.3447e+02	5.3473e-03	4.5284e+02	5.3656e-03	5.4180e+02
3.2050e-03	7.0751e+02	2.8610e-03	7.2634e+02	3.5864e-03	6.0479e+02	3.5987e-03	5.6130e+02
2.1496e-03	8.3363e+02	1.9189e-03	8.7132e+02	2.4054e-03	7.5521e+02	2.4137e-03	7.5484e+02
1.4417e-03	1.0185e+03	1.2870e-03	1.2156e+03	1.6133e-03	1.0670e+03	1.6189e-03	1.0573e+03
9.6698e-04	1.3340e+03	8.6319e-04	1.4349e+03	1.0821e-03	1.1226e+03	1.0858e-03	1.3186e+03
6.4856e-04	1.5308e+03	5.7895e-04	1.4821e+03	7.2574e-04	1.4783e+03	7.2823e-04	1.4868e+03
4.3499e-04	1.5179e+03	3.8830e-04	1.6377e+03	4.8675e-04	1.6446e+03	4.8842e-04	1.5462e+03
2.9175e-04	1.7721e+03	2.6043e-04	1.5956e+03	3.2647e-04	1.7311e+03	3.2759e-04	1.8455e+03
1.9568e-04	1.8478e+03	1.7467e-04	1.8325e+03	2.1896e-04	1.7520e+03	2.1971e-04	1.8338e+03
1.3124e-04	1.8302e+03	1.1715e-04	2.0445e+03	1.4686e-04	1.8312e+03	1.4736e-04	1.8886e+03
8.8023e-05	1.7829e+03	7.8575e-05	1.8291e+03	9.8498e-05	1.8427e+03	9.8835e-05	1.8563e+03
5.9037e-05	1.7985e+03	5.2700e-05	1.8992e+03	6.6063e-05	1.9808e+03	6.6289e-05	1.9600e+03
3.9596e-05	1.9416e+03	3.5346e-05	1.9841e+03	4.4308e-05	2.0157e+03	4.4460e-05	1.9975e+03
2.6557e-05	1.9393e+03	2.3707e-05	2.0305e+03	2.9718e-05	1.8742e+03	2.9820e-05	1.8333e+03
1.7812e-05	1.8852e+03	1.5900e-05	1.9546e+03	1.9932e-05	1.9570e+03	2.0000e-05	2.0044e+03

FREQUENCY [Hz]: 53604		57364		58134		60075	
TAU [s]	MAGNITUDES [a.u]	TAU [s]	MAGNITUDES [a.u]	TAU [s]	MAGNITUDES [a.u]	TAU [s]	MAGNITUDES [a.u]
7.9898e-03	4.1398e+02	8.0635e-03	4.3831e+02	8.0182e-03	4.5266e+02	8.0000e-03	3.9894e+02
5.3588e-03	5.7289e+02	5.4082e-03	4.2291e+02	5.3778e-03	5.2165e+02	5.3656e-03	4.7219e+02
3.5941e-03	5.8496e+02	3.6273e-03	5.3434e+02	3.6069e-03	6.0938e+02	3.5987e-03	7.0920e+02
2.4106e-03	9.0706e+02	2.4328e-03	7.7679e+02	2.4191e-03	7.3978e+02	2.4137e-03	8.7552e+02
1.6168e-03	1.0317e+03	1.6317e-03	1.2363e+03	1.6225e-03	1.1953e+03	1.6189e-03	1.0850e+03
1.0844e-03	1.3172e+03	1.0944e-03	1.2259e+03	1.0882e-03	1.3543e+03	1.0858e-03	1.3486e+03
7.2730e-04	1.5258e+03	7.3401e-04	1.4417e+03	7.2988e-04	1.5463e+03	7.2823e-04	1.4763e+03
4.8780e-04	1.6700e+03	4.9230e-04	1.6279e+03	4.8953e-04	1.6215e+03	4.8842e-04	1.5919e+03
3.2717e-04	1.6749e+03	3.3019e-04	1.7813e+03	3.2833e-04	1.6927e+03	3.2759e-04	1.6837e+03
2.1943e-04	1.8444e+03	2.2146e-04	1.8906e+03	2.2021e-04	1.8746e+03	2.1971e-04	1.6955e+03
1.4717e-04	1.7491e+03	1.4853e-04	1.9287e+03	1.4770e-04	1.9770e+03	1.4736e-04	1.9028e+03
9.8709e-05	1.8242e+03	9.9620e-05	1.9691e+03	9.9060e-05	1.9917e+03	9.8835e-05	1.9752e+03
6.6205e-05	1.9413e+03	6.6815e-05	1.9512e+03	6.6439e-05	1.8865e+03	6.6289e-05	2.0091e+03
4.4403e-05	1.9630e+03	4.4813e-05	1.9119e+03	4.4561e-05	1.8948e+03	4.4460e-05	1.9214e+03
2.9781e-05	1.9577e+03	3.0056e-05	1.9563e+03	2.9887e-05	1.8510e+03	2.9820e-05	1.9342e+03
1.9974e-05	1.9056e+03	2.0159e-05	1.8607e+03	2.0045e-05	2.0173e+03	2.0000e-05	1.9681e+03

FREQUENCY [Hz]: 63246		68993		74990		81691	
TAU [s]	MAGNITUDES [a.u]	TAU [s]	MAGNITUDES [a.u]	TAU [s]	MAGNITUDES [a.u]	TAU [s]	MAGNITUDES [a.u]
8.5840e-03	4.0246e+02	8.7706e-03	4.0114e+02	8.7235e-03	3.9598e+02	1.0556e-02	3.6817e+02
5.7573e-03	3.9409e+02	5.8824e-03	4.6239e+02	5.8508e-03	4.3171e+02	7.0800e-03	5.8081e+02
3.8614e-03	5.2098e+02	3.9454e-03	6.1469e+02	3.9242e-03	6.8737e+02	4.7486e-03	7.8697e+02
2.5899e-03	7.5613e+02	2.6462e-03	8.7716e+02	2.6319e-03	8.8627e+02	3.1849e-03	8.6508e+02
1.7370e-03	1.0674e+03	1.7748e-03	1.0497e+03	1.7652e-03	1.1101e+03	2.1361e-03	1.0230e+03
1.1650e-03	1.3433e+03	1.1904e-03	1.4369e+03	1.1840e-03	1.4323e+03	1.4327e-03	1.2737e+03
7.8139e-04	1.5048e+03	7.9837e-04	1.3927e+03	7.9408e-04	1.5978e+03	9.6091e-04	1.5546e+03
5.2408e-04	1.6024e+03	5.3547e-04	1.7141e+03	5.3259e-04	1.6682e+03	6.4448e-04	1.6359e+03
3.5150e-04	1.7633e+03	3.5914e-04	1.7049e+03	3.5721e-04	1.7718e+03	4.3226e-04	1.7219e+03
2.3575e-04	1.8627e+03	2.4088e-04	1.8938e+03	2.3958e-04	1.8884e+03	2.8991e-04	1.9622e+03
1.5812e-04	1.9036e+03	1.6156e-04	1.8504e+03	1.6069e-04	1.9964e+03	1.9445e-04	1.9918e+03
1.0605e-04	2.0164e+03	1.0836e-04	1.9401e+03	1.0777e-04	2.0969e+03	1.3042e-04	1.9369e+03
7.1128e-05	1.9932e+03	7.2674e-05	1.9102e+03	7.2284e-05	2.0252e+03	8.7470e-05	2.1069e+03
4.7706e-05	1.9504e+03	4.8743e-05	2.0780e+03	4.8481e-05	2.0468e+03	5.8666e-05	2.1173e+03
3.1996e-05	1.9834e+03	3.2692e-05	2.0475e+03	3.2516e-05	2.0356e+03	3.9347e-05	2.0183e+03
2.1460e-05	1.9983e+03	2.1926e-05	1.9804e+03	2.1809e-05	2.1916e+03	2.6390e-05	2.0844e+03

FREQUENCY [Hz]: 88682		96453		105070		108200	
TAU [s]	MAGNITUDES [a.u]	TAU [s]	MAGNITUDES [a.u]	TAU [s]	MAGNITUDES [a.u]	TAU [s]	MAGNITUDES [a.u]
9.2298e-03	3.5052e+02	9.8999e-03	4.2210e+02	1.1078e-02	4.3884e+02	1.0705e-02	4.6009e+02
6.1904e-03	5.5783e+02	6.6399e-03	5.8157e+02	7.4298e-03	4.6360e+02	7.1801e-03	5.6031e+02
4.1519e-03	6.7750e+02	4.4534e-03	6.9517e+02	4.9832e-03	6.8084e+02	4.8157e-03	7.4619e+02
2.7847e-03	9.4777e+02	2.9869e-03	9.0234e+02	3.3422e-03	8.9975e+02	3.2299e-03	9.7068e+02
1.8677e-03	1.1230e+03	2.0033e-03	1.1742e+03	2.2416e-03	1.0659e+03	2.1663e-03	1.2771e+03
1.2527e-03	1.4501e+03	1.3436e-03	1.4358e+03	1.5035e-03	1.3775e+03	1.4529e-03	1.4422e+03
8.4017e-04	1.6695e+03	9.0117e-04	1.5376e+03	1.0084e-03	1.5038e+03	9.7449e-04	1.5674e+03
5.6350e-04	1.8314e+03	6.0442e-04	1.6794e+03	6.7632e-04	1.8847e+03	6.5359e-04	1.7291e+03
3.7794e-04	1.9201e+03	4.0538e-04	1.8815e+03	4.5361e-04	1.9312e+03	4.3836e-04	1.8707e+03
2.5349e-04	2.0230e+03	2.7189e-04	1.9990e+03	3.0424e-04	1.9926e+03	2.9401e-04	2.0964e+03
1.7001e-04	1.9753e+03	1.8236e-04	1.9863e+03	2.0405e-04	2.0614e+03	1.9719e-04	2.0166e+03
1.1403e-04	2.0472e+03	1.2231e-04	2.1259e+03	1.3686e-04	2.1809e+03	1.3226e-04	1.9018e+03
7.6479e-05	2.1249e+03	8.2032e-05	2.0817e+03	9.1791e-05	2.0882e+03	8.8706e-05	2.0125e+03
5.1295e-05	1.9909e+03	5.5019e-05	2.0669e+03	6.1564e-05	2.0632e+03	5.9495e-05	2.0626e+03
3.4404e-05	2.1009e+03	3.6901e-05	2.1881e+03	4.1291e-05	2.0912e+03	3.9903e-05	1.8810e+03
2.3074e-05	2.0748e+03	2.4750e-05	2.0920e+03	2.7694e-05	2.2148e+03	2.6763e-05	2.1397e+03

FREQUENCY [Hz]: 114050		124520		133120		135460	
TAU [s]	MAGNITUDES [a.u]	TAU [s]	MAGNITUDES [a.u]	TAU [s]	MAGNITUDES [a.u]	TAU [s]	MAGNITUDES [a.u]
1.2309e-02	4.0701e+02	1.3541e-02	4.3073e+02	1.2414e-02	4.2440e+02	1.1776e-02	3.2238e+02
8.2558e-03	4.9426e+02	9.0818e-03	4.2709e+02	8.3263e-03	4.6624e+02	7.8985e-03	6.0134e+02
5.5372e-03	6.9528e+02	6.0912e-03	5.0040e+02	5.5844e-03	6.5209e+02	5.2975e-03	7.0213e+02
3.7138e-03	8.1762e+02	4.0854e-03	8.1178e+02	3.7455e-03	1.0194e+03	3.5531e-03	8.8606e+02
2.4908e-03	1.1357e+03	2.7401e-03	1.1885e+03	2.5121e-03	1.2174e+03	2.3830e-03	1.3634e+03
1.6706e-03	1.4292e+03	1.8378e-03	1.3698e+03	1.6849e-03	1.5223e+03	1.5983e-03	1.5217e+03
1.1205e-03	1.6230e+03	1.2326e-03	1.6414e+03	1.1300e-03	1.6186e+03	1.0720e-03	1.7065e+03
7.5151e-04	1.8123e+03	8.2670e-04	1.8535e+03	7.5793e-04	1.7349e+03	7.1898e-04	1.8847e+03
5.0404e-04	2.0012e+03	5.5447e-04	1.8891e+03	5.0834e-04	1.8629e+03	4.8222e-04	2.0169e+03
3.3806e-04	2.0076e+03	3.7188e-04	2.0913e+03	3.4095e-04	2.0405e+03	3.2343e-04	2.0687e+03
2.2674e-04	2.1320e+03	2.4942e-04	2.1055e+03	2.2867e-04	2.1441e+03	2.1692e-04	2.1349e+03
1.5207e-04	2.1298e+03	1.6729e-04	2.1323e+03	1.5337e-04	2.1455e+03	1.4549e-04	2.1157e+03
1.0200e-04	2.1906e+03	1.1220e-04	2.1560e+03	1.0287e-04	2.1711e+03	9.7581e-05	2.2140e+03
6.8408e-05	2.2150e+03	7.5253e-05	2.1994e+03	6.8993e-05	2.1258e+03	6.5448e-05	2.2545e+03
4.5882e-05	2.1576e+03	5.0472e-05	2.2100e+03	4.6273e-05	2.2247e+03	4.3896e-05	2.2119e+03
3.0773e-05	2.2328e+03	3.3852e-05	2.1702e+03	3.1036e-05	2.1767e+03	2.9441e-05	2.2462e+03

FREQUENCY [Hz]: 146860		147470		159990		163850	
TAU [s]	MAGNITUDES [a.u]	TAU [s]	MAGNITUDES [a.u]	TAU [s]	MAGNITUDES [a.u]	TAU [s]	MAGNITUDES [a.u]
1.3427e-02	4.9390e+02	1.5999e-02	3.8205e+02	1.4000e-02	4.5128e+02	1.4680e-02	4.5336e+02
9.0054e-03	6.4159e+02	1.0731e-02	5.3346e+02	9.3898e-03	5.0221e+02	9.8457e-03	4.5712e+02
6.0399e-03	6.8630e+02	7.1971e-03	4.3759e+02	6.2978e-03	8.2125e+02	6.6035e-03	7.5790e+02
4.0510e-03	9.2089e+02	4.8271e-03	8.7202e+02	4.2239e-03	1.0244e+03	4.4290e-03	9.9632e+02
2.7170e-03	1.2140e+03	3.2376e-03	1.0866e+03	2.8330e-03	1.1352e+03	2.9705e-03	1.2991e+03
1.8223e-03	1.4955e+03	2.1714e-03	1.2201e+03	1.9001e-03	1.4737e+03	1.9923e-03	1.4268e+03
1.2222e-03	1.5884e+03	1.4564e-03	1.5864e+03	1.2744e-03	1.7565e+03	1.3363e-03	1.7371e+03
8.1974e-04	1.8485e+03	9.7680e-04	1.7204e+03	8.5474e-04	1.8709e+03	8.9623e-04	1.8320e+03
5.4980e-04	2.0574e+03	6.5514e-04	1.8254e+03	5.7327e-04	2.0593e+03	6.0111e-04	1.7630e+03
3.6875e-04	2.0433e+03	4.3941e-04	1.9819e+03	3.8450e-04	2.1734e+03	4.0316e-04	1.9041e+03
2.4732e-04	2.1515e+03	2.9471e-04	2.0617e+03	2.5788e-04	2.2675e+03	2.7040e-04	2.1666e+03
1.6588e-04	2.1295e+03	1.9766e-04	2.1826e+03	1.7296e-04	2.1476e+03	1.8136e-04	2.1625e+03
1.1126e-04	2.2535e+03	1.3257e-04	2.1987e+03	1.1601e-04	2.1425e+03	1.2164e-04	2.0413e+03
7.4620e-05	2.2061e+03	8.8916e-05	2.0474e+03	7.7805e-05	2.3033e+03	8.1583e-05	2.1281e+03
5.0048e-05	2.2224e+03	5.9636e-05	2.3375e+03	5.2184e-05	2.3044e+03	5.4718e-05	2.3370e+03
3.3567e-05	2.2276e+03	3.9998e-05	2.1162e+03	3.5000e-05	2.3343e+03	3.6699e-05	2.1748e+03

FREQUENCY [Hz]: 181760		223390		248190		275140	
TAU [s]	MAGNITUDES [a.u]	TAU [s]	MAGNITUDES [a.u]	TAU [s]	MAGNITUDES [a.u]	TAU [s]	MAGNITUDES [a.u]
1.3511e-02	3.6613e+02	1.7645e-02	6.1683e+02	1.7358e-02	5.2662e+02	1.8251e-02	4.7094e+02
9.0619e-03	6.6072e+02	1.1835e-02	4.9813e+02	1.1642e-02	4.7740e+02	1.2241e-02	5.8529e+02
6.0778e-03	8.7044e+02	7.9376e-03	7.6251e+02	7.8085e-03	8.8170e+02	8.2099e-03	6.9023e+02
4.0764e-03	9.5145e+02	5.3238e-03	1.0014e+03	5.2372e-03	1.0609e+03	5.5064e-03	1.0358e+03
2.7340e-03	1.2841e+03	3.5707e-03	1.1924e+03	3.5126e-03	1.2765e+03	3.6932e-03	1.3736e+03
1.8337e-03	1.5643e+03	2.3948e-03	1.4625e+03	2.3559e-03	1.7407e+03	2.4770e-03	1.4257e+03
1.2299e-03	1.7878e+03	1.6062e-03	1.6994e+03	1.5801e-03	1.8862e+03	1.6613e-03	1.8729e+03
8.2489e-04	1.8198e+03	1.0773e-03	1.8620e+03	1.0598e-03	1.7840e+03	1.1143e-03	2.0646e+03
5.5325e-04	2.0122e+03	7.2255e-04	2.2949e+03	7.1079e-04	2.0113e+03	7.4734e-04	2.1682e+03
3.7107e-04	2.2157e+03	4.8461e-04	2.2796e+03	4.7673e-04	2.1674e+03	5.0124e-04	2.1730e+03
2.4888e-04	2.0893e+03	3.2503e-04	2.1906e+03	3.1974e-04	2.2833e+03	3.3618e-04	2.2569e+03
1.6692e-04	2.2086e+03	2.1800e-04	2.2815e+03	2.1445e-04	2.3358e+03	2.2548e-04	2.3423e+03
1.1195e-04	2.3272e+03	1.4621e-04	2.3450e+03	1.4383e-04	2.4425e+03	1.5123e-04	2.5210e+03
7.5088e-05	2.2370e+03	9.8064e-05	2.1674e+03	9.6470e-05	2.3794e+03	1.0143e-04	2.4343e+03
5.0362e-05	2.3098e+03	6.5772e-05	2.2203e+03	6.4702e-05	2.4972e+03	6.8029e-05	2.2812e+03
3.3778e-05	2.2374e+03	4.4113e-05	2.3011e+03	4.3396e-05	2.3279e+03	4.5627e-05	2.4746e+03

FREQUENCY [Hz]: 305230		338750		374980	
TAU [s]	MAGNITUDES [a.u]	TAU [s]	MAGNITUDES [a.u]	TAU [s]	MAGNITUDES [a.u]
2.0416e-02	4.4525e+02	2.1160e-02	4.1638e+02	1.9388e-02	4.3965e+02
1.3693e-02	4.8700e+02	1.4192e-02	5.3661e+02	1.3003e-02	7.2186e+02
9.1838e-03	7.5908e+02	9.5185e-03	7.7794e+02	8.7214e-03	8.7477e+02
6.1596e-03	9.4514e+02	6.3841e-03	1.1764e+03	5.8494e-03	1.2047e+03
4.1312e-03	1.2087e+03	4.2818e-03	1.3708e+03	3.9232e-03	1.4860e+03
2.7708e-03	1.5103e+03	2.8718e-03	1.5250e+03	2.6313e-03	1.6977e+03
1.8584e-03	1.7933e+03	1.9261e-03	1.7669e+03	1.7648e-03	1.8907e+03
1.2464e-03	2.0211e+03	1.2919e-03	1.8877e+03	1.1837e-03	2.0581e+03
8.3598e-04	2.1524e+03	8.6645e-04	2.1597e+03	7.9389e-04	2.3098e+03
5.6069e-04	2.1958e+03	5.8113e-04	2.2447e+03	5.3246e-04	2.3793e+03
3.7606e-04	2.2013e+03	3.8977e-04	2.4816e+03	3.5712e-04	2.3194e+03
2.5222e-04	2.3922e+03	2.6142e-04	2.4380e+03	2.3952e-04	2.5134e+03
1.6917e-04	2.4050e+03	1.7533e-04	2.4342e+03	1.6065e-04	2.5496e+03
1.1346e-04	2.4503e+03	1.1760e-04	2.3623e+03	1.0775e-04	2.5768e+03
7.6098e-05	2.4151e+03	7.8872e-05	2.4984e+03	7.2266e-05	2.4361e+03
5.1039e-05	2.4290e+03	5.2899e-05	2.5647e+03	4.8469e-05	2.6093e+03

FREQUENCY [Hz]: 461610		511720		534820		548930	
TAU [s]	MAGNITUDES [a.u]	TAU [s]	MAGNITUDES [a.u]	TAU [s]	MAGNITUDES [a.u]	TAU [s]	MAGNITUDES [a.u]
2.5249e-02	4.4020e+02	2.1298e-02	5.8058e+02	2.3908e-02	4.0644e+02	1.9590e-02	4.2824e+02
1.6934e-02	7.2055e+02	1.4284e-02	5.6428e+02	1.6035e-02	5.9386e+02	1.3139e-02	7.0608e+02
1.1358e-02	7.1429e+02	9.5806e-03	8.9698e+02	1.0755e-02	8.8129e+02	8.8125e-03	8.7205e+02
7.6178e-03	1.0073e+03	6.4257e-03	1.0154e+03	7.2131e-03	1.1516e+03	5.9105e-03	1.3097e+03
5.1093e-03	1.4364e+03	4.3097e-03	1.6077e+03	4.8379e-03	1.3789e+03	3.9642e-03	1.4583e+03
3.4268e-03	1.6295e+03	2.8905e-03	1.8089e+03	3.2448e-03	1.6699e+03	2.6588e-03	1.7725e+03
2.2983e-03	1.9512e+03	1.9387e-03	2.0778e+03	2.1763e-03	1.8756e+03	1.7833e-03	1.9976e+03
1.5415e-03	1.9883e+03	1.3003e-03	2.1386e+03	1.4596e-03	2.1994e+03	1.1960e-03	2.1638e+03
1.0339e-03	2.2838e+03	8.7210e-04	2.2313e+03	9.7897e-04	2.3816e+03	8.0218e-04	2.1897e+03
6.9343e-04	2.2630e+03	5.8492e-04	2.1611e+03	6.5660e-04	2.5686e+03	5.3803e-04	2.4531e+03
4.6509e-04	2.3366e+03	3.9231e-04	2.3835e+03	4.4038e-04	2.4532e+03	3.6086e-04	2.4088e+03
3.1193e-04	2.5633e+03	2.6312e-04	2.4097e+03	2.9537e-04	2.3148e+03	2.4203e-04	2.5140e+03
2.0921e-04	2.5282e+03	1.7648e-04	2.4808e+03	1.9810e-04	2.5238e+03	1.6233e-04	2.4879e+03
1.4032e-04	2.5075e+03	1.1836e-04	2.4305e+03	1.3287e-04	2.5075e+03	1.0887e-04	2.4890e+03
9.4113e-05	2.5047e+03	7.9386e-05	2.5842e+03	8.9114e-05	2.6592e+03	7.3021e-05	2.5484e+03
6.3122e-05	2.6504e+03	5.3244e-05	2.5237e+03	5.9769e-05	2.5916e+03	4.8976e-05	2.6628e+03

FREQUENCY [Hz]: 568020		580110		600980		623460	
TAU [s]	MAGNITUDES [a.u]	TAU [s]	MAGNITUDES [a.u]	TAU [s]	MAGNITUDES [a.u]	TAU [s]	MAGNITUDES [a.u]
2.0742e-02	4.9443e+02	2.2000e-02	4.4508e+02	1.9947e-02	4.8164e+02	2.1767e-02	6.0466e+02
1.3911e-02	5.6996e+02	1.4755e-02	6.3020e+02	1.3379e-02	5.9241e+02	1.4599e-02	5.9677e+02
9.3305e-03	7.7803e+02	9.8965e-03	6.8471e+02	8.9732e-03	8.5021e+02	9.7915e-03	7.7259e+02
6.2580e-03	1.1231e+03	6.6376e-03	1.1074e+03	6.0183e-03	1.1399e+03	6.5672e-03	1.0962e+03
4.1972e-03	1.4054e+03	4.4518e-03	1.5870e+03	4.0365e-03	1.4965e+03	4.4046e-03	1.4180e+03
2.8151e-03	1.6536e+03	2.9859e-03	1.6788e+03	2.7073e-03	1.5862e+03	2.9542e-03	1.5789e+03
1.8881e-03	1.9572e+03	2.0026e-03	2.0185e+03	1.8158e-03	1.9091e+03	1.9814e-03	1.9649e+03
1.2663e-03	2.1033e+03	1.3432e-03	2.1899e+03	1.2178e-03	2.1679e+03	1.3289e-03	2.1836e+03
8.4933e-04	2.1462e+03	9.0086e-04	2.3164e+03	8.1681e-04	2.2518e+03	8.9130e-04	2.1205e+03
5.6965e-04	2.2707e+03	6.0421e-04	2.3566e+03	5.4784e-04	2.3577e+03	5.9780e-04	2.2512e+03
3.8207e-04	2.4815e+03	4.0524e-04	2.3357e+03	3.6744e-04	2.4880e+03	4.0094e-04	2.4364e+03
2.5625e-04	2.4781e+03	2.7180e-04	2.5227e+03	2.4644e-04	2.4267e+03	2.6891e-04	2.4360e+03
1.7187e-04	2.4981e+03	1.8229e-04	2.6159e+03	1.6529e-04	2.4305e+03	1.8036e-04	2.6367e+03
1.1527e-04	2.5781e+03	1.2227e-04	2.5612e+03	1.1086e-04	2.4132e+03	1.2097e-04	2.5110e+03
7.7313e-05	2.4046e+03	8.2004e-05	2.5987e+03	7.4353e-05	2.5255e+03	8.1134e-05	2.5488e+03
5.1854e-05	2.5378e+03	5.5000e-05	2.6601e+03	4.9869e-05	2.6145e+03	5.4416e-05	2.4631e+03

FREQUENCY [Hz]: 629780		645080		669680		693410	
TAU [s]	MAGNITUDES [a.u]	TAU [s]	MAGNITUDES [a.u]	TAU [s]	MAGNITUDES [a.u]	TAU [s]	MAGNITUDES [a.u]
2.4708e-02	5.4355e+02	1.8955e-02	4.0662e+02	2.2902e-02	5.8077e+02	2.4864e-02	5.0489e+02
1.6571e-02	5.3536e+02	1.2713e-02	5.9567e+02	1.5360e-02	5.9273e+02	1.6677e-02	6.7928e+02
1.1115e-02	7.0279e+02	8.5267e-03	9.7548e+02	1.0302e-02	8.2991e+02	1.1185e-02	8.6041e+02
7.4545e-03	9.5712e+02	5.7189e-03	1.1152e+03	6.9097e-03	1.0895e+03	7.5018e-03	1.0725e+03
4.9998e-03	1.3023e+03	3.8357e-03	1.5231e+03	4.6344e-03	1.2092e+03	5.0315e-03	1.3332e+03
3.3533e-03	1.6383e+03	2.5726e-03	1.7168e+03	3.1083e-03	1.7483e+03	3.3746e-03	1.7774e+03
2.2491e-03	1.8960e+03	1.7254e-03	1.9529e+03	2.0847e-03	1.7949e+03	2.2634e-03	1.9748e+03
1.5085e-03	1.9816e+03	1.1573e-03	2.2120e+03	1.3982e-03	2.0914e+03	1.5180e-03	2.2166e+03
1.0117e-03	2.2060e+03	7.7617e-04	2.2537e+03	9.3779e-04	2.3685e+03	1.0181e-03	2.3411e+03
6.7857e-04	2.3140e+03	5.2058e-04	2.4372e+03	6.2898e-04	2.3618e+03	6.8287e-04	2.4254e+03
4.5512e-04	2.3196e+03	3.4915e-04	2.4644e+03	4.2186e-04	2.4765e+03	4.5800e-04	2.5369e+03
3.0525e-04	2.5968e+03	2.3418e-04	2.6149e+03	2.8294e-04	2.5405e+03	3.0718e-04	2.6899e+03
2.0473e-04	2.6707e+03	1.5706e-04	2.5772e+03	1.8977e-04	2.4956e+03	2.0603e-04	2.6056e+03
1.3731e-04	2.4435e+03	1.0534e-04	2.5891e+03	1.2728e-04	2.6032e+03	1.3818e-04	2.7036e+03
9.2096e-05	2.6331e+03	7.0653e-05	2.5175e+03	8.5366e-05	2.6245e+03	9.2680e-05	2.6007e+03
6.1769e-05	2.4319e+03	4.7387e-05	2.6232e+03	5.7255e-05	2.5509e+03	6.2161e-05	2.5590e+03

FREQUENCY [Hz]: 698730		718900		744850		771270	
TAU [s]	MAGNITUDES [a.u]	TAU [s]	MAGNITUDES [a.u]	TAU [s]	MAGNITUDES [a.u]	TAU [s]	MAGNITUDES [a.u]
2.6969e-02	4.5776e+02	2.3270e-02	4.8686e+02	2.6737e-02	5.7673e+02	2.6746e-02	4.9920e+02
1.8088e-02	5.8444e+02	1.5607e-02	5.0656e+02	1.7933e-02	6.1037e+02	1.7938e-02	6.2439e+02
1.2132e-02	7.7285e+02	1.0468e-02	8.5099e+02	1.2028e-02	8.6769e+02	1.2031e-02	9.0096e+02
8.1367e-03	1.0253e+03	7.0208e-03	1.3328e+03	8.0669e-03	1.1244e+03	8.0694e-03	1.1849e+03
5.4573e-03	1.2597e+03	4.7088e-03	1.4771e+03	5.4105e-03	1.4731e+03	5.4122e-03	1.3544e+03
3.6602e-03	1.6975e+03	3.1582e-03	1.7118e+03	3.6288e-03	1.6388e+03	3.6300e-03	1.8151e+03
2.4549e-03	2.0375e+03	2.1182e-03	1.7823e+03	2.4338e-03	1.8674e+03	2.4346e-03	2.0504e+03
1.6465e-03	2.1327e+03	1.4207e-03	2.0808e+03	1.6324e-03	2.2667e+03	1.6329e-03	2.1478e+03
1.1043e-03	2.2219e+03	9.5286e-04	2.3483e+03	1.0948e-03	2.4074e+03	1.0952e-03	2.4156e+03
7.4067e-04	2.3983e+03	6.3909e-04	2.5368e+03	7.3431e-04	2.4328e+03	7.3455e-04	2.5415e+03
4.9677e-04	2.4185e+03	4.2864e-04	2.4830e+03	4.9251e-04	2.5814e+03	4.9266e-04	2.5113e+03
3.3318e-04	2.5476e+03	2.8749e-04	2.6584e+03	3.3032e-04	2.7970e+03	3.3043e-04	2.6996e+03
2.2347e-04	2.6443e+03	1.9282e-04	2.6457e+03	2.2155e-04	2.6974e+03	2.2162e-04	2.5926e+03
1.4988e-04	2.5865e+03	1.2932e-04	2.4435e+03	1.4859e-04	2.6401e+03	1.4864e-04	2.6213e+03
1.0052e-04	2.6035e+03	8.6737e-05	2.6725e+03	9.9662e-05	2.6717e+03	9.9693e-05	2.6687e+03
6.7422e-05	2.6050e+03	5.8175e-05	2.5706e+03	6.6843e-05	2.5831e+03	6.6864e-05	2.6828e+03

FREQUENCY [Hz]: 775480		799570		824020		848850	
TAU [s]	MAGNITUDES [a.u]	TAU [s]	MAGNITUDES [a.u]	TAU [s]	MAGNITUDES [a.u]	TAU [s]	MAGNITUDES [a.u]
3.6351e-02	4.6771e+02	2.9424e-02	5.1238e+02	3.2820e-02	4.8525e+02	3.3031e-02	4.5351e+02
2.4381e-02	6.6061e+02	1.9735e-02	6.9918e+02	2.2012e-02	5.5667e+02	2.2154e-02	6.6207e+02
1.6352e-02	6.7461e+02	1.3236e-02	8.3061e+02	1.4764e-02	7.6571e+02	1.4858e-02	9.5459e+02
1.0968e-02	8.9016e+02	8.8775e-03	1.1493e+03	9.9021e-03	1.0076e+03	9.9656e-03	9.9254e+02
7.3559e-03	1.3208e+03	5.9541e-03	1.5110e+03	6.6413e-03	1.3592e+03	6.6840e-03	1.5055e+03
4.9336e-03	1.6016e+03	3.9934e-03	1.8114e+03	4.4544e-03	1.7720e+03	4.4829e-03	1.8994e+03
3.3090e-03	1.8907e+03	2.6784e-03	2.0898e+03	2.9875e-03	2.0228e+03	3.0067e-03	2.1560e+03
2.2194e-03	1.9935e+03	1.7964e-03	2.2817e+03	2.0037e-03	2.3278e+03	2.0166e-03	2.3811e+03
1.4885e-03	2.5230e+03	1.2049e-03	2.5690e+03	1.3439e-03	2.5336e+03	1.3525e-03	2.4389e+03
9.9835e-04	2.3568e+03	8.0810e-04	2.7078e+03	9.0137e-04	2.6204e+03	9.0715e-04	2.6135e+03
6.6960e-04	2.4769e+03	5.4199e-04	2.6550e+03	6.0455e-04	2.6488e+03	6.0843e-04	2.6961e+03
4.4910e-04	2.5931e+03	3.6352e-04	2.7587e+03	4.0547e-04	2.7958e+03	4.0807e-04	2.6517e+03
3.0121e-04	2.6707e+03	2.4381e-04	2.7261e+03	2.7195e-04	2.7048e+03	2.7370e-04	2.8023e+03
2.0202e-04	2.7578e+03	1.6352e-04	2.8393e+03	1.8240e-04	2.6644e+03	1.8357e-04	2.8806e+03
1.3550e-04	2.6974e+03	1.0968e-04	2.8603e+03	1.2233e-04	2.8264e+03	1.2312e-04	2.6935e+03
9.0878e-05	2.7222e+03	7.3560e-05	2.8176e+03	8.2050e-05	2.7468e+03	8.2576e-05	2.8857e+03

FREQUENCY [Hz]: 859180		874040		899610		953390	
TAU [s]	MAGNITUDES [a.u]	TAU [s]	MAGNITUDES [a.u]	TAU [s]	MAGNITUDES [a.u]	TAU [s]	MAGNITUDES [a.u]
4.1117e-02	5.3906e+02	3.4815e-02	4.9048e+02	3.6000e-02	4.8902e+02	4.0270e-02	6.0703e+02
2.7577e-02	5.5925e+02	2.3350e-02	5.9482e+02	2.4145e-02	7.1240e+02	2.7009e-02	5.4071e+02
1.8496e-02	8.5218e+02	1.5661e-02	8.4274e+02	1.6194e-02	9.9635e+02	1.8115e-02	9.2640e+02
1.2405e-02	1.1118e+03	1.0504e-02	1.1255e+03	1.0862e-02	1.3284e+03	1.2150e-02	1.2004e+03
8.3203e-03	1.3718e+03	7.0450e-03	1.4277e+03	7.2848e-03	1.5622e+03	8.1489e-03	1.5830e+03
5.5805e-03	1.7510e+03	4.7251e-03	1.9014e+03	4.8860e-03	1.9316e+03	5.4655e-03	1.8841e+03
3.7428e-03	1.9205e+03	3.1691e-03	2.0702e+03	3.2770e-03	2.0282e+03	3.6657e-03	2.1346e+03
2.5103e-03	2.2863e+03	2.1255e-03	2.4857e+03	2.1979e-03	2.2941e+03	2.4586e-03	2.2125e+03
1.6837e-03	2.3149e+03	1.4256e-03	2.4836e+03	1.4741e-03	2.6647e+03	1.6490e-03	2.3721e+03
1.1292e-03	2.4522e+03	9.5615e-04	2.6788e+03	9.8870e-04	2.5963e+03	1.1060e-03	2.5651e+03
7.5739e-04	2.4968e+03	6.4129e-04	2.5879e+03	6.6313e-04	2.8505e+03	7.4178e-04	2.5365e+03
5.0798e-04	2.7692e+03	4.3011e-04	2.7123e+03	4.4476e-04	2.8259e+03	4.9751e-04	2.5539e+03
3.4070e-04	2.7420e+03	2.8848e-04	2.9315e+03	2.9830e-04	2.8927e+03	3.3368e-04	2.7944e+03
2.2851e-04	2.8308e+03	1.9348e-04	2.8762e+03	2.0007e-04	2.9414e+03	2.2380e-04	2.7251e+03
1.5326e-04	2.6097e+03	1.2977e-04	2.9146e+03	1.3419e-04	2.9150e+03	1.5010e-04	2.8521e+03
1.0279e-04	2.6442e+03	8.7036e-05	2.8081e+03	9.0000e-05	2.9465e+03	1.0067e-04	2.8578e+03

FREQUENCY [Hz]: 1.0574E6		1.172E6		1.2998E6		1.4418E6	
TAU [s]	MAGNITUDES [a.u]	TAU [s]	MAGNITUDES [a.u]	TAU [s]	MAGNITUDES [a.u]	TAU [s]	MAGNITUDES [a.u]
4.2755e-02	5.0801e+02	3.2995e-02	6.9582e+02	4.9601e-02	6.1986e+02	4.2530e-02	6.5616e+02
2.8676e-02	6.6626e+02	2.2130e-02	8.6784e+02	3.3267e-02	6.7515e+02	2.8525e-02	7.7504e+02
1.9233e-02	8.2292e+02	1.4843e-02	1.3657e+03	2.2312e-02	8.7392e+02	1.9132e-02	1.0016e+03
1.2900e-02	1.1592e+03	9.9549e-03	1.5157e+03	1.4965e-02	1.1524e+03	1.2832e-02	1.3403e+03
8.6517e-03	1.3945e+03	6.6768e-03	1.7858e+03	1.0037e-02	1.6424e+03	8.6062e-03	1.9452e+03
5.8027e-03	1.8841e+03	4.4781e-03	2.0400e+03	6.7318e-03	1.8907e+03	5.7722e-03	2.1337e+03
3.8919e-03	2.2357e+03	3.0035e-03	2.1724e+03	4.5150e-03	2.1915e+03	3.8714e-03	2.0619e+03
2.6103e-03	2.2863e+03	2.0144e-03	2.6052e+03	3.0282e-03	2.4310e+03	2.5966e-03	2.4659e+03
1.7507e-03	2.4893e+03	1.3511e-03	2.6866e+03	2.0311e-03	2.4888e+03	1.7415e-03	2.6435e+03
1.1742e-03	2.7115e+03	9.0618e-04	2.6131e+03	1.3622e-03	2.5541e+03	1.1680e-03	2.6626e+03
7.8755e-04	2.7644e+03	6.0777e-04	2.8217e+03	9.1365e-04	2.8179e+03	7.8341e-04	2.7419e+03
5.2821e-04	2.7114e+03	4.0764e-04	2.5078e+03	6.1279e-04	2.9919e+03	5.2543e-04	2.9547e+03
3.5427e-04	2.7844e+03	2.7340e-04	2.9279e+03	4.1100e-04	2.9789e+03	3.5241e-04	2.7099e+03
2.3761e-04	2.9026e+03	1.8337e-04	2.7727e+03	2.7566e-04	2.7306e+03	2.3636e-04	2.9856e+03
1.5937e-04	2.7586e+03	1.2299e-04	3.0315e+03	1.8488e-04	2.9634e+03	1.5853e-04	2.8815e+03
1.0689e-04	2.8769e+03	8.2488e-05	3.0728e+03	1.2400e-04	2.9904e+03	1.0632e-04	2.8936e+03

FREQUENCY [Hz]: 1.5993E6		1.7564E6		1.814E6		1.8748E6	
TAU [s]	MAGNITUDES [a.u]	TAU [s]	MAGNITUDES [a.u]	TAU [s]	MAGNITUDES [a.u]	TAU [s]	MAGNITUDES [a.u]
6.2168e-02	6.2314e+02	5.1312e-02	5.9046e+02	5.3644e-02	6.4815e+02	5.0143e-02	5.4749e+02
4.1696e-02	7.0185e+02	3.4415e-02	6.9051e+02	3.5979e-02	7.0881e+02	3.3631e-02	7.9920e+02
2.7966e-02	8.0338e+02	2.3082e-02	9.8136e+02	2.4131e-02	9.9576e+02	2.2556e-02	9.9683e+02
1.8757e-02	1.2097e+03	1.5481e-02	1.3108e+03	1.6185e-02	1.3192e+03	1.5129e-02	1.3580e+03
1.2580e-02	1.6236e+03	1.0383e-02	1.7006e+03	1.0855e-02	1.6148e+03	1.0147e-02	1.6517e+03
8.4375e-03	1.6344e+03	6.9641e-03	2.0110e+03	7.2806e-03	1.9090e+03	6.8054e-03	2.0201e+03
5.6590e-03	2.0606e+03	4.6708e-03	2.3205e+03	4.8831e-03	2.1797e+03	4.5644e-03	2.2421e+03
3.7955e-03	2.5275e+03	3.1327e-03	2.3873e+03	3.2751e-03	2.6109e+03	3.0614e-03	2.5691e+03
2.5457e-03	2.6138e+03	2.1011e-03	2.4740e+03	2.1966e-03	2.6098e+03	2.0533e-03	2.5594e+03
1.7074e-03	2.7276e+03	1.4092e-03	2.7702e+03	1.4733e-03	2.7815e+03	1.3771e-03	2.7013e+03
1.1451e-03	2.7216e+03	9.4517e-04	2.8589e+03	9.8813e-04	2.8454e+03	9.2364e-04	2.8513e+03
7.6805e-04	2.7659e+03	6.3393e-04	2.8633e+03	6.6274e-04	2.8449e+03	6.1949e-04	2.7972e+03
5.1513e-04	2.9803e+03	4.2518e-04	2.9065e+03	4.4450e-04	2.8572e+03	4.1549e-04	2.8929e+03
3.4550e-04	2.9927e+03	2.8517e-04	3.0784e+03	2.9813e-04	2.8419e+03	2.7867e-04	2.8955e+03
2.3173e-04	2.7313e+03	1.9126e-04	2.8916e+03	1.9995e-04	3.0795e+03	1.8690e-04	3.0189e+03
1.5542e-04	3.0529e+03	1.2828e-04	2.9379e+03	1.3411e-04	3.0144e+03	1.2536e-04	3.0481e+03

FREQUENCY [Hz]: 1.9365E6		1.9588E6		1.9992E6		2.0652E6	
TAU [s]	MAGNITUDES [a.u]	TAU [s]	MAGNITUDES [a.u]	TAU [s]	MAGNITUDES [a.u]	TAU [s]	MAGNITUDES [a.u]
4.1336e-02	5.7498e+02	3.0139e-02	8.0086e+02	3.4670e-02	7.2104e+02	3.5962e-02	6.3444e+02
2.7724e-02	7.9614e+02	2.0214e-02	9.7060e+02	2.3253e-02	7.5391e+02	2.4120e-02	8.2557e+02
1.8595e-02	1.0192e+03	1.3558e-02	1.2363e+03	1.5596e-02	1.2154e+03	1.6177e-02	1.0049e+03
1.2472e-02	1.4405e+03	9.0933e-03	1.6953e+03	1.0460e-02	1.3104e+03	1.0850e-02	1.3128e+03
8.3647e-03	1.8117e+03	6.0989e-03	1.8769e+03	7.0156e-03	1.8250e+03	7.2772e-03	1.5377e+03
5.6102e-03	1.9517e+03	4.0905e-03	2.2787e+03	4.7054e-03	2.0684e+03	4.8808e-03	1.9312e+03
3.7628e-03	2.3181e+03	2.7435e-03	2.1782e+03	3.1559e-03	2.2889e+03	3.2736e-03	2.2736e+03
2.5237e-03	2.4886e+03	1.8401e-03	2.6370e+03	2.1167e-03	2.6881e+03	2.1956e-03	2.4373e+03
1.6926e-03	2.6257e+03	1.2341e-03	2.8771e+03	1.4197e-03	2.7456e+03	1.4726e-03	2.3358e+03
1.1353e-03	2.6902e+03	8.2775e-04	2.8224e+03	9.5217e-04	2.7697e+03	9.8766e-04	2.6239e+03
7.6142e-04	2.8246e+03	5.5517e-04	2.8779e+03	6.3862e-04	2.7866e+03	6.6243e-04	2.8785e+03
5.1069e-04	2.8240e+03	3.7235e-04	2.8528e+03	4.2832e-04	2.6879e+03	4.4429e-04	2.7580e+03
3.4252e-04	2.7986e+03	2.4974e-04	3.0091e+03	2.8728e-04	2.9406e+03	2.9799e-04	3.0049e+03
2.2973e-04	2.9770e+03	1.6750e-04	2.8513e+03	1.9268e-04	2.8945e+03	1.9986e-04	2.7699e+03
1.5408e-04	2.8871e+03	1.1234e-04	3.0219e+03	1.2923e-04	2.8782e+03	1.3405e-04	2.9111e+03
1.0334e-04	2.9384e+03	7.5348e-05	3.0048e+03	8.6674e-05	2.9514e+03	8.9905e-05	2.7625e+03

FREQUENCY [Hz]: 2.0998E6		2.1206E6		2.13E6		2.1346E6	
TAU [s]	MAGNITUDES [a.u]	TAU [s]	MAGNITUDES [a.u]	TAU [s]	MAGNITUDES [a.u]	TAU [s]	MAGNITUDES [a.u]
3.4301e-02	5.6739e+02	3.5255e-02	6.8164e+02	4.0686e-02	8.2790e+02	3.2048e-02	6.3169e+02
2.3006e-02	7.3021e+02	2.3645e-02	8.2972e+02	2.7288e-02	6.7683e+02	2.1495e-02	8.1047e+02
1.5430e-02	8.7027e+02	1.5859e-02	1.0694e+03	1.8302e-02	7.8251e+02	1.4417e-02	1.0288e+03
1.0349e-02	1.3287e+03	1.0637e-02	1.3086e+03	1.2275e-02	1.1102e+03	9.6692e-03	1.2985e+03
6.9410e-03	1.7370e+03	7.1340e-03	1.7281e+03	8.2331e-03	1.5734e+03	6.4851e-03	1.6963e+03
4.6554e-03	2.0186e+03	4.7848e-03	1.9659e+03	5.5220e-03	1.7267e+03	4.3496e-03	1.9475e+03
3.1224e-03	2.2070e+03	3.2092e-03	2.1658e+03	3.7036e-03	2.0878e+03	2.9173e-03	2.2062e+03
2.0942e-03	2.5048e+03	2.1524e-03	2.4353e+03	2.4840e-03	2.2096e+03	1.9566e-03	2.3780e+03
1.4046e-03	2.6679e+03	1.4436e-03	2.6105e+03	1.6660e-03	2.4113e+03	1.3123e-03	2.6459e+03
9.4204e-04	2.5942e+03	9.6823e-04	2.6870e+03	1.1174e-03	2.3617e+03	8.8017e-04	2.6319e+03
6.3183e-04	2.7234e+03	6.4939e-04	2.9071e+03	7.4945e-04	2.7373e+03	5.9033e-04	2.5788e+03
4.2377e-04	2.6752e+03	4.3555e-04	2.8698e+03	5.0266e-04	2.7193e+03	3.9594e-04	2.7207e+03
2.8422e-04	2.9158e+03	2.9212e-04	2.9083e+03	3.3713e-04	2.7947e+03	2.6555e-04	2.8154e+03
1.9063e-04	3.0038e+03	1.9593e-04	2.9456e+03	2.2611e-04	2.9536e+03	1.7811e-04	2.7264e+03
1.2785e-04	2.9842e+03	1.3141e-04	2.9098e+03	1.5166e-04	2.8149e+03	1.1946e-04	2.8330e+03
8.5752e-05	2.8235e+03	8.8136e-05	2.8522e+03	1.0172e-04	2.7990e+03	8.0120e-05	2.7914e+03

FREQUENCY [Hz]: 2.144E6		2.1651E6		2.1863E6		2.2052E6	
TAU [s]	MAGNITUDES [a.u]	TAU [s]	MAGNITUDES [a.u]	TAU [s]	MAGNITUDES [a.u]	TAU [s]	MAGNITUDES [a.u]
3.2936e-02	5.8813e+02	3.1515e-02	6.4267e+02	2.8534e-02	7.0497e+02	3.1218e-02	7.1950e+02
2.2090e-02	7.0820e+02	2.1137e-02	7.3362e+02	1.9138e-02	7.6861e+02	2.0938e-02	7.1692e+02
1.4816e-02	1.0638e+03	1.4177e-02	9.2170e+02	1.2836e-02	1.0059e+03	1.4043e-02	9.7204e+02
9.9369e-03	1.2945e+03	9.5083e-03	1.4568e+03	8.6090e-03	1.2974e+03	9.4187e-03	1.4095e+03
6.6647e-03	1.7533e+03	6.3772e-03	1.6129e+03	5.7741e-03	1.8010e+03	6.3171e-03	1.5447e+03
4.4700e-03	2.0055e+03	4.2772e-03	1.9604e+03	3.8727e-03	2.0661e+03	4.2369e-03	2.0333e+03
2.9981e-03	2.1947e+03	2.8687e-03	2.2002e+03	2.5974e-03	2.3927e+03	2.8417e-03	2.2249e+03
2.0108e-03	2.2743e+03	1.9241e-03	2.5262e+03	1.7421e-03	2.5276e+03	1.9059e-03	2.4650e+03
1.3487e-03	2.5857e+03	1.2905e-03	2.6744e+03	1.1684e-03	2.6033e+03	1.2783e-03	2.5383e+03
9.0454e-04	2.7794e+03	8.6552e-04	2.7881e+03	7.8366e-04	2.7211e+03	8.5737e-04	2.7436e+03
6.0668e-04	2.8644e+03	5.8051e-04	2.8544e+03	5.2560e-04	2.7594e+03	5.7504e-04	2.7047e+03
4.0690e-04	2.8231e+03	3.8935e-04	2.7040e+03	3.5252e-04	2.8897e+03	3.8568e-04	2.7858e+03
2.7291e-04	2.8989e+03	2.6114e-04	2.8822e+03	2.3644e-04	2.9285e+03	2.5868e-04	2.9073e+03
1.8304e-04	2.8293e+03	1.7514e-04	2.9615e+03	1.5858e-04	2.9116e+03	1.7349e-04	2.8579e+03
1.2277e-04	2.9374e+03	1.1747e-04	2.9131e+03	1.0636e-04	3.0007e+03	1.1636e-04	2.7765e+03
8.2339e-05	2.9593e+03	7.8787e-05	2.9316e+03	7.1335e-05	2.8805e+03	7.8045e-05	2.9020e+03

FREQUENCY [Hz]: 2.2099E6		2.2313E6		2.2529E6		2.2769E6	
TAU [s]	MAGNITUDES [a.u]	TAU [s]	MAGNITUDES [a.u]	TAU [s]	MAGNITUDES [a.u]	TAU [s]	MAGNITUDES [a.u]
3.3504e-02	6.7080e+02	3.8978e-02	5.1474e+02	3.6374e-02	5.6430e+02	4.5646e-02	6.4340e+02
2.2471e-02	7.2011e+02	2.6143e-02	6.2774e+02	2.4396e-02	7.3790e+02	3.0615e-02	7.8195e+02
1.5071e-02	1.0106e+03	1.7534e-02	8.3360e+02	1.6362e-02	1.0197e+03	2.0533e-02	9.3989e+02
1.0108e-02	1.2475e+03	1.1760e-02	1.1167e+03	1.0974e-02	1.3857e+03	1.3772e-02	1.0281e+03
6.7797e-03	1.6175e+03	7.8875e-03	1.5568e+03	7.3604e-03	1.5889e+03	9.2367e-03	1.3832e+03
4.5472e-03	1.8147e+03	5.2902e-03	1.7903e+03	4.9367e-03	2.0239e+03	6.1951e-03	1.6618e+03
3.0498e-03	2.2949e+03	3.5481e-03	2.1580e+03	3.3110e-03	2.2908e+03	4.1550e-03	2.0728e+03
2.0455e-03	2.3833e+03	2.3797e-03	2.3728e+03	2.2207e-03	2.4166e+03	2.7868e-03	2.2410e+03
1.3719e-03	2.5944e+03	1.5961e-03	2.6765e+03	1.4894e-03	2.5732e+03	1.8691e-03	2.4065e+03
9.2015e-04	2.6082e+03	1.0705e-03	2.7141e+03	9.9896e-04	2.7601e+03	1.2536e-03	2.7112e+03
6.1714e-04	2.7091e+03	7.1799e-04	2.6991e+03	6.7001e-04	2.8244e+03	8.4080e-04	2.6498e+03
4.1392e-04	2.7751e+03	4.8156e-04	2.9659e+03	4.4937e-04	2.9108e+03	5.6393e-04	2.8501e+03
2.7762e-04	2.9499e+03	3.2298e-04	2.8483e+03	3.0140e-04	2.8235e+03	3.7823e-04	2.9173e+03
1.8620e-04	3.0169e+03	2.1662e-04	2.9742e+03	2.0215e-04	2.8450e+03	2.5368e-04	2.8226e+03
1.2488e-04	2.9769e+03	1.4529e-04	3.0002e+03	1.3558e-04	3.0173e+03	1.7014e-04	2.9090e+03
8.3759e-05	3.1540e+03	9.7446e-05	3.0832e+03	9.0934e-05	2.9827e+03	1.1411e-04	2.7922e+03

FREQUENCY [Hz]: 2.301E6		2.318E6		2.3522E6		2.4287E6	
TAU [s]	MAGNITUDES [a.u]	TAU [s]	MAGNITUDES [a.u]	TAU [s]	MAGNITUDES [a.u]	TAU [s]	MAGNITUDES [a.u]
3.8000e-02	6.3041e+02	5.4759e-02	5.5077e+02	5.2431e-02	6.0361e+02	5.6182e-02	6.4519e+02
2.5487e-02	7.6873e+02	3.6727e-02	7.4732e+02	3.5166e-02	6.4128e+02	3.7681e-02	7.8422e+02
1.7094e-02	9.5607e+02	2.4633e-02	7.0440e+02	2.3586e-02	8.4227e+02	2.5273e-02	9.0348e+02
1.1465e-02	1.2639e+03	1.6521e-02	9.9884e+02	1.5819e-02	1.2101e+03	1.6950e-02	1.3933e+03
7.6896e-03	1.6399e+03	1.1081e-02	1.3860e+03	1.0610e-02	1.6397e+03	1.1369e-02	1.6297e+03
5.1574e-03	2.0317e+03	7.4320e-03	1.6937e+03	7.1160e-03	1.8321e+03	7.6250e-03	1.8241e+03
3.4591e-03	2.3587e+03	4.9846e-03	1.9371e+03	4.7727e-03	2.0097e+03	5.1141e-03	2.3132e+03
2.3200e-03	2.5234e+03	3.3432e-03	2.2944e+03	3.2011e-03	2.4943e+03	3.4300e-03	2.4664e+03
1.5560e-03	2.7435e+03	2.2423e-03	2.5878e+03	2.1470e-03	2.5696e+03	2.3005e-03	2.6385e+03
1.0436e-03	2.7079e+03	1.5039e-03	2.5295e+03	1.4400e-03	2.6585e+03	1.5430e-03	2.7773e+03
6.9997e-04	2.9456e+03	1.0087e-03	2.7616e+03	9.6579e-04	2.8075e+03	1.0349e-03	2.8761e+03
4.6947e-04	2.8612e+03	6.7652e-04	2.7748e+03	6.4776e-04	2.8977e+03	6.9409e-04	2.9764e+03
3.1487e-04	2.9963e+03	4.5374e-04	2.8956e+03	4.3445e-04	2.8192e+03	4.6553e-04	2.9740e+03
2.1119e-04	2.9515e+03	3.0433e-04	2.8351e+03	2.9139e-04	2.8770e+03	3.1223e-04	2.9641e+03
1.4164e-04	3.0339e+03	2.0411e-04	2.8881e+03	1.9543e-04	2.9800e+03	2.0941e-04	2.8480e+03
9.5000e-05	3.0825e+03	1.3690e-04	2.6924e+03	1.3108e-04	3.1155e+03	1.4045e-04	3.0114e+03

FREQUENCY [Hz]: 2.509E6		2.5191E6		2.5932E6		2.6787E6	
TAU [s]	MAGNITUDES [a.u]	TAU [s]	MAGNITUDES [a.u]	TAU [s]	MAGNITUDES [a.u]	TAU [s]	MAGNITUDES [a.u]
6.2735e-02	6.5470e+02	4.1466e-02	9.2934e+02	5.4980e-02	7.6079e+02	3.7104e-02	8.0214e+02
4.2077e-02	8.0588e+02	2.7811e-02	9.4120e+02	3.6875e-02	8.7139e+02	2.4886e-02	1.0200e+03
2.8221e-02	1.0454e+03	1.8653e-02	1.2005e+03	2.4732e-02	1.1649e+03	1.6691e-02	1.3779e+03
1.8928e-02	1.3035e+03	1.2511e-02	1.7226e+03	1.6588e-02	1.5707e+03	1.1195e-02	1.7807e+03
1.2695e-02	1.6178e+03	8.3908e-03	2.0269e+03	1.1126e-02	1.7782e+03	7.5083e-03	1.8884e+03
8.5145e-03	1.9129e+03	5.6278e-03	2.2812e+03	7.4619e-03	2.2379e+03	5.0358e-03	2.2672e+03
5.7107e-03	2.3325e+03	3.7745e-03	2.4882e+03	5.0047e-03	2.3361e+03	3.3775e-03	2.5070e+03
3.8302e-03	2.4950e+03	2.5316e-03	2.7845e+03	3.3567e-03	2.6295e+03	2.2653e-03	2.5284e+03
2.5689e-03	2.7226e+03	1.6979e-03	2.7210e+03	2.2513e-03	2.7683e+03	1.5193e-03	2.7701e+03
1.7230e-03	2.8580e+03	1.1388e-03	2.9143e+03	1.5100e-03	2.8006e+03	1.0190e-03	2.7895e+03
1.1556e-03	2.9236e+03	7.6380e-04	2.8583e+03	1.0127e-03	2.9082e+03	6.8346e-04	2.7360e+03
7.7506e-04	2.9101e+03	5.1228e-04	2.9436e+03	6.7924e-04	2.9696e+03	4.5840e-04	2.9579e+03
5.1983e-04	3.0009e+03	3.4359e-04	3.1270e+03	4.5557e-04	3.0247e+03	3.0745e-04	2.9592e+03
3.4865e-04	3.0233e+03	2.3045e-04	3.1201e+03	3.0555e-04	3.0354e+03	2.0621e-04	3.1028e+03
2.3384e-04	3.0368e+03	1.5456e-04	2.9758e+03	2.0493e-04	2.9612e+03	1.3830e-04	2.8347e+03
1.5684e-04	3.0767e+03	1.0366e-04	2.8826e+03	1.3745e-04	3.1088e+03	9.2760e-05	2.9815e+03

FREQUENCY [Hz]: 2.6997E6		2.7207E6		2.7392E6		2.7418E6	
TAU [s]	MAGNITUDES [a.u]	TAU [s]	MAGNITUDES [a.u]	TAU [s]	MAGNITUDES [a.u]	TAU [s]	MAGNITUDES [a.u]
3.8605e-02	7.3075e+02	3.8399e-02	8.5180e+02	3.5373e-02	7.1485e+02	3.9492e-02	7.3327e+02
2.5892e-02	8.5347e+02	2.5754e-02	9.6108e+02	2.3725e-02	8.9552e+02	2.6487e-02	8.2186e+02
1.7366e-02	1.2620e+03	1.7273e-02	1.1637e+03	1.5912e-02	1.1785e+03	1.7765e-02	1.1371e+03
1.1647e-02	1.5398e+03	1.1585e-02	1.5058e+03	1.0672e-02	1.4414e+03	1.1915e-02	1.3819e+03
7.8120e-03	1.9625e+03	7.7703e-03	1.7808e+03	7.1579e-03	1.7487e+03	7.9914e-03	1.6654e+03
5.2395e-03	2.1469e+03	5.2115e-03	2.1377e+03	4.8008e-03	2.1083e+03	5.3598e-03	1.9140e+03
3.5141e-03	2.4583e+03	3.4954e-03	2.4800e+03	3.2199e-03	2.3142e+03	3.5949e-03	2.3434e+03
2.3569e-03	2.7412e+03	2.3444e-03	2.5687e+03	2.1596e-03	2.5250e+03	2.4111e-03	2.5265e+03
1.5808e-03	2.7554e+03	1.5724e-03	2.7251e+03	1.4484e-03	2.6582e+03	1.6171e-03	2.6667e+03
1.0602e-03	2.6882e+03	1.0546e-03	2.8878e+03	9.7148e-04	2.6913e+03	1.0846e-03	2.7107e+03
7.1111e-04	3.0395e+03	7.0732e-04	3.0736e+03	6.5157e-04	2.7593e+03	7.2744e-04	2.8611e+03
4.7694e-04	2.9899e+03	4.7440e-04	2.8744e+03	4.3701e-04	2.8426e+03	4.8790e-04	2.9282e+03
3.1989e-04	3.0111e+03	3.1818e-04	3.1358e+03	2.9310e-04	2.8363e+03	3.2723e-04	2.9022e+03
2.1455e-04	2.9465e+03	2.1340e-04	2.9659e+03	1.9658e-04	2.8842e+03	2.1948e-04	3.0297e+03
1.4390e-04	3.0269e+03	1.4313e-04	3.0513e+03	1.3185e-04	2.8011e+03	1.4720e-04	2.8969e+03
9.6513e-05	3.0550e+03	9.5997e-05	3.0457e+03	8.8432e-05	3.0110e+03	9.8729e-05	2.9148e+03

FREQUENCY [Hz]: 2.7603E6		2.7657E6		2.7816E6		2.803E6	
TAU [s]	MAGNITUDES [a.u]	TAU [s]	MAGNITUDES [a.u]	TAU [s]	MAGNITUDES [a.u]	TAU [s]	MAGNITUDES [a.u]
4.3324e-02	7.0965e+02	2.8304e-02	7.6827e+02	3.5549e-02	7.3570e+02	2.9862e-02	8.4051e+02
2.9058e-02	7.4572e+02	1.8983e-02	1.1443e+03	2.3843e-02	7.5900e+02	2.0028e-02	8.7429e+02
1.9489e-02	1.0363e+03	1.2732e-02	1.3414e+03	1.5991e-02	1.1863e+03	1.3433e-02	1.2483e+03
1.3071e-02	1.2122e+03	8.5394e-03	1.5783e+03	1.0725e-02	1.3846e+03	9.0096e-03	1.5447e+03
8.7669e-03	1.7623e+03	5.7274e-03	1.8901e+03	7.1936e-03	1.8647e+03	6.0428e-03	1.8428e+03
5.8800e-03	1.9739e+03	3.8414e-03	2.1192e+03	4.8248e-03	2.2090e+03	4.0529e-03	2.2103e+03
3.9437e-03	2.1414e+03	2.5764e-03	2.4522e+03	3.2360e-03	2.3566e+03	2.7183e-03	2.3624e+03
2.6451e-03	2.5472e+03	1.7280e-03	2.5419e+03	2.1704e-03	2.3785e+03	1.8232e-03	2.5033e+03
1.7740e-03	2.5466e+03	1.1590e-03	2.7020e+03	1.4557e-03	2.6286e+03	1.2228e-03	2.6292e+03
1.1899e-03	2.6873e+03	7.7733e-04	2.8333e+03	9.7632e-04	2.7227e+03	8.2013e-04	2.6642e+03
7.9804e-04	2.7551e+03	5.2136e-04	2.8696e+03	6.5482e-04	2.8656e+03	5.5006e-04	2.8587e+03
5.3524e-04	2.8404e+03	3.4967e-04	2.7539e+03	4.3919e-04	2.8465e+03	3.6893e-04	2.8956e+03
3.5899e-04	2.9126e+03	2.3453e-04	2.8105e+03	2.9456e-04	2.8101e+03	2.4744e-04	2.8986e+03
2.4077e-04	2.9150e+03	1.5730e-04	2.9678e+03	1.9756e-04	3.0023e+03	1.6596e-04	2.9298e+03
1.6149e-04	2.9480e+03	1.0550e-04	2.8869e+03	1.3251e-04	2.9232e+03	1.1131e-04	2.9046e+03
1.0831e-04	3.2313e+03	7.0759e-05	2.9198e+03	8.8873e-05	2.9361e+03	7.4655e-05	3.0007e+03

FREQUENCY [Hz]: 2.8244E6		2.8459E6		2.8567E6		2.8675E6	
TAU [s]	MAGNITUDES [a.u]	TAU [s]	MAGNITUDES [a.u]	TAU [s]	MAGNITUDES [a.u]	TAU [s]	MAGNITUDES [a.u]
2.9346e-02	8.3313e+02	2.8338e-02	7.2831e+02	3.4221e-02	7.0793e+02	2.7850e-02	7.4882e+02
1.9682e-02	9.4023e+02	1.9006e-02	8.1843e+02	2.2952e-02	7.3244e+02	1.8679e-02	1.0240e+03
1.3201e-02	1.0031e+03	1.2748e-02	1.1330e+03	1.5394e-02	9.1898e+02	1.2528e-02	1.1481e+03
8.8539e-03	1.5223e+03	8.5499e-03	1.4203e+03	1.0325e-02	1.1663e+03	8.4025e-03	1.3681e+03
5.9383e-03	1.7853e+03	5.7344e-03	1.7430e+03	6.9248e-03	1.4944e+03	5.6356e-03	1.7499e+03
3.9828e-03	1.9741e+03	3.8461e-03	2.1420e+03	4.6445e-03	1.8555e+03	3.7798e-03	2.1524e+03
2.6713e-03	2.4137e+03	2.5796e-03	2.2470e+03	3.1150e-03	2.0105e+03	2.5351e-03	2.2634e+03
1.7916e-03	2.5195e+03	1.7301e-03	2.4712e+03	2.0893e-03	2.4095e+03	1.7003e-03	2.6641e+03
1.2017e-03	2.7657e+03	1.1604e-03	2.6752e+03	1.4013e-03	2.4587e+03	1.1404e-03	2.5875e+03
8.0595e-04	2.8864e+03	7.7828e-04	2.7219e+03	9.3984e-04	2.5857e+03	7.6487e-04	2.6628e+03
5.4055e-04	2.7337e+03	5.2199e-04	2.8631e+03	6.3035e-04	2.7138e+03	5.1300e-04	2.9042e+03
3.6255e-04	2.8431e+03	3.5010e-04	2.9812e+03	4.2278e-04	2.6673e+03	3.4407e-04	2.8290e+03
2.4316e-04	3.0306e+03	2.3481e-04	2.9643e+03	2.8356e-04	2.8651e+03	2.3077e-04	2.8290e+03
1.6309e-04	2.9804e+03	1.5749e-04	3.0162e+03	1.9018e-04	2.7995e+03	1.5478e-04	3.0714e+03
1.0938e-04	2.9564e+03	1.0563e-04	2.9604e+03	1.2756e-04	2.9000e+03	1.0381e-04	2.9069e+03
7.3364e-05	2.8910e+03	7.0845e-05	2.9938e+03	8.5552e-05	2.7806e+03	6.9624e-05	3.0407e+03

FREQUENCY [Hz]: 2.8892E6		2.9109E6		2.9327E6		2.9519E6	
TAU [s]	MAGNITUDES [a.u]	TAU [s]	MAGNITUDES [a.u]	TAU [s]	MAGNITUDES [a.u]	TAU [s]	MAGNITUDES [a.u]
3.3014e-02	7.8714e+02	3.4115e-02	7.1620e+02	3.3266e-02	6.6359e+02	5.0192e-02	7.1357e+02
2.2142e-02	7.8311e+02	2.2881e-02	7.5765e+02	2.2312e-02	8.2414e+02	3.3664e-02	7.0385e+02
1.4851e-02	1.0026e+03	1.5346e-02	1.0359e+03	1.4965e-02	9.7688e+02	2.2578e-02	7.4499e+02
9.9605e-03	1.2909e+03	1.0293e-02	1.4034e+03	1.0037e-02	1.3175e+03	1.5143e-02	1.0699e+03
6.6805e-03	1.5893e+03	6.9033e-03	1.5696e+03	6.7316e-03	1.6516e+03	1.0157e-02	1.3942e+03
4.4806e-03	1.9047e+03	4.6301e-03	2.0938e+03	4.5149e-03	2.1896e+03	6.8121e-03	1.7121e+03
3.0052e-03	2.2471e+03	3.1054e-03	2.1579e+03	3.0282e-03	2.3605e+03	4.5689e-03	2.0883e+03
2.0156e-03	2.4376e+03	2.0828e-03	2.5544e+03	2.0310e-03	2.5723e+03	3.0643e-03	2.2081e+03
1.3518e-03	2.5472e+03	1.3969e-03	2.6526e+03	1.3622e-03	2.7552e+03	2.0553e-03	2.4874e+03
9.0669e-04	2.7262e+03	9.3692e-04	2.7692e+03	9.1362e-04	2.7498e+03	1.3785e-03	2.5410e+03
6.0812e-04	2.8262e+03	6.2840e-04	2.6993e+03	6.1277e-04	2.7930e+03	9.2454e-04	2.7824e+03
4.0786e-04	2.8397e+03	4.2147e-04	2.8720e+03	4.1099e-04	2.8767e+03	6.2009e-04	2.8276e+03
2.7356e-04	2.8561e+03	2.8268e-04	2.9569e+03	2.7565e-04	2.9916e+03	4.1590e-04	2.7428e+03
1.8347e-04	2.8785e+03	1.8959e-04	2.9649e+03	1.8488e-04	2.9662e+03	2.7894e-04	2.8833e+03
1.2306e-04	2.9763e+03	1.2716e-04	2.9236e+03	1.2400e-04	3.0222e+03	1.8709e-04	3.0330e+03
8.2534e-05	3.0480e+03	8.5286e-05	3.0478e+03	8.3166e-05	3.1804e+03	1.2548e-04	2.9584e+03

FREQUENCY [Hz]: 2.9547E6		2.9767E6		2.9794E6		2.9987E6	
TAU [s]	MAGNITUDES [a.u]	TAU [s]	MAGNITUDES [a.u]	TAU [s]	MAGNITUDES [a.u]	TAU [s]	MAGNITUDES [a.u]
3.7834e-02	6.7815e+02	3.9170e-02	6.6076e+02	7.6751e-02	6.0847e+02	4.7144e-02	6.6427e+02
2.5375e-02	8.5146e+02	2.6271e-02	8.0657e+02	5.1477e-02	5.9106e+02	3.1620e-02	8.6568e+02
1.7019e-02	1.0236e+03	1.7620e-02	1.0703e+03	3.4526e-02	8.1218e+02	2.1207e-02	9.8922e+02
1.1415e-02	1.3470e+03	1.1818e-02	1.2813e+03	2.3157e-02	9.1316e+02	1.4224e-02	1.2582e+03
7.6559e-03	1.6427e+03	7.9263e-03	1.7219e+03	1.5531e-02	1.1179e+03	9.5400e-03	1.6749e+03
5.1348e-03	1.9385e+03	5.3162e-03	2.0794e+03	1.0417e-02	1.2969e+03	6.3985e-03	1.9971e+03
3.4439e-03	2.2376e+03	3.5656e-03	2.3589e+03	6.9865e-03	1.8357e+03	4.2915e-03	2.3004e+03
2.3098e-03	2.6029e+03	2.3914e-03	2.5452e+03	4.6859e-03	2.0157e+03	2.8783e-03	2.4450e+03
1.5492e-03	2.6040e+03	1.6039e-03	2.8630e+03	3.1428e-03	2.2906e+03	1.9305e-03	2.7813e+03
1.0391e-03	2.9134e+03	1.0758e-03	2.9032e+03	2.1079e-03	2.4729e+03	1.2948e-03	2.8207e+03
6.9690e-04	2.8488e+03	7.2152e-04	2.9882e+03	1.4138e-03	2.5941e+03	8.6841e-04	3.0040e+03
4.6741e-04	3.0157e+03	4.8392e-04	2.8578e+03	9.4822e-04	2.8614e+03	5.8244e-04	2.9296e+03
3.1349e-04	3.0549e+03	3.2457e-04	2.9763e+03	6.3597e-04	3.0161e+03	3.9064e-04	3.0828e+03
2.1026e-04	3.0205e+03	2.1769e-04	3.1450e+03	4.2655e-04	2.9065e+03	2.6201e-04	3.0151e+03
1.4102e-04	2.9672e+03	1.4600e-04	3.1346e+03	2.8609e-04	2.9412e+03	1.7573e-04	3.0142e+03
9.4584e-05	3.1033e+03	9.7925e-05	3.1452e+03	1.9188e-04	2.9600e+03	1.1786e-04	3.0693e+03

FREQUENCY [Hz]: 3.0237E6		3.0487E6		3.0739E6		3.0991E6	
TAU [s]	MAGNITUDES [a.u]	TAU [s]	MAGNITUDES [a.u]	TAU [s]	MAGNITUDES [a.u]	TAU [s]	MAGNITUDES [a.u]
5.0395e-02	7.1849e+02	5.8420e-02	7.6415e+02	5.9508e-02	6.7392e+02	6.0000e-02	7.5273e+02
3.3800e-02	7.7119e+02	3.9182e-02	7.9510e+02	3.9912e-02	8.3871e+02	4.0242e-02	9.4591e+02
2.2670e-02	1.1046e+03	2.6280e-02	1.0254e+03	2.6769e-02	1.0267e+03	2.6990e-02	1.2120e+03
1.5204e-02	1.4322e+03	1.7626e-02	1.3518e+03	1.7954e-02	1.4948e+03	1.8103e-02	1.5781e+03
1.0198e-02	1.7288e+03	1.1822e-02	1.7151e+03	1.2042e-02	1.7817e+03	1.2141e-02	1.8965e+03
6.8396e-03	2.0558e+03	7.9288e-03	2.0653e+03	8.0764e-03	2.0782e+03	8.1433e-03	2.1706e+03
4.5873e-03	2.3989e+03	5.3179e-03	2.3801e+03	5.4169e-03	2.5208e+03	5.4617e-03	2.5580e+03
3.0767e-03	2.5290e+03	3.5667e-03	2.5756e+03	3.6331e-03	2.6602e+03	3.6632e-03	2.6200e+03
2.0636e-03	2.6554e+03	2.3922e-03	2.8159e+03	2.4367e-03	2.9598e+03	2.4569e-03	2.9312e+03
1.3840e-03	2.9025e+03	1.6044e-03	2.8910e+03	1.6343e-03	2.8279e+03	1.6478e-03	3.0380e+03
9.2828e-04	3.0011e+03	1.0761e-03	2.9757e+03	1.0961e-03	3.1070e+03	1.1052e-03	3.0150e+03
6.2260e-04	3.1329e+03	7.2175e-04	2.9913e+03	7.3518e-04	3.1750e+03	7.4127e-04	3.1571e+03
4.1758e-04	3.1056e+03	4.8408e-04	3.1027e+03	4.9309e-04	3.2012e+03	4.9717e-04	3.0591e+03
2.8007e-04	3.1387e+03	3.2467e-04	3.2826e+03	3.3072e-04	3.2139e+03	3.3345e-04	3.4276e+03
1.8784e-04	3.2050e+03	2.1776e-04	3.2717e+03	2.2181e-04	3.1914e+03	2.2365e-04	3.3231e+03
1.2599e-04	3.0680e+03	1.4605e-04	3.1741e+03	1.4877e-04	3.2999e+03	1.5000e-04	3.3599e+03

FREQUENCY [Hz]: 3.1499E6		3.2412E6		3.5231E6		3.8323E6	
TAU [s]	MAGNITUDES [a.u]	TAU [s]	MAGNITUDES [a.u]	TAU [s]	MAGNITUDES [a.u]	TAU [s]	MAGNITUDES [a.u]
1.2000e-01	7.5664e+02	8.7722e-02	8.4531e+02	1.0170e-01	8.9736e+02	1.1662e-01	7.3404e+02
8.0484e-02	7.7142e+02	5.8836e-02	9.5261e+02	6.8211e-02	9.0501e+02	7.8220e-02	9.7410e+02
5.3981e-02	8.7991e+02	3.9461e-02	1.1628e+03	4.5749e-02	1.1225e+03	5.2463e-02	1.0367e+03
3.6205e-02	1.1966e+03	2.6467e-02	1.4956e+03	3.0684e-02	1.4327e+03	3.5187e-02	1.4454e+03
2.4283e-02	1.2284e+03	1.7751e-02	1.7713e+03	2.0580e-02	1.8419e+03	2.3600e-02	1.6966e+03
1.6287e-02	1.7242e+03	1.1906e-02	2.1387e+03	1.3803e-02	2.1617e+03	1.5828e-02	2.2318e+03
1.0923e-02	2.0903e+03	7.9852e-03	2.4282e+03	9.2577e-03	2.3872e+03	1.0616e-02	2.4386e+03
7.3263e-03	2.2686e+03	5.3557e-03	2.4903e+03	6.2091e-03	2.5753e+03	7.1203e-03	2.6555e+03
4.9138e-03	2.5558e+03	3.5921e-03	2.7770e+03	4.1645e-03	2.6686e+03	4.7756e-03	2.7738e+03
3.2957e-03	2.6193e+03	2.4092e-03	3.0313e+03	2.7931e-03	3.1235e+03	3.2030e-03	3.0008e+03
2.2104e-03	3.1133e+03	1.6159e-03	3.0088e+03	1.8734e-03	3.0820e+03	2.1482e-03	3.0780e+03
1.4825e-03	2.8746e+03	1.0838e-03	3.1417e+03	1.2565e-03	3.0404e+03	1.4408e-03	3.0955e+03
9.9434e-04	3.0814e+03	7.2688e-04	3.0376e+03	8.4271e-04	3.1933e+03	9.6637e-04	3.1557e+03
6.6690e-04	3.0388e+03	4.8752e-04	3.2522e+03	5.6521e-04	3.1792e+03	6.4814e-04	3.3740e+03
4.4729e-04	3.1882e+03	3.2698e-04	3.0988e+03	3.7909e-04	3.1843e+03	4.3471e-04	3.3167e+03
3.0000e-04	3.1821e+03	2.1931e-04	3.2560e+03	2.5425e-04	3.2222e+03	2.9156e-04	3.2609e+03

FREQUENCY [Hz]: 4.1643E6		4.5304E6		4.9262E6		5.0797E6	
TAU [s]	MAGNITUDES [a.u]	TAU [s]	MAGNITUDES [a.u]	TAU [s]	MAGNITUDES [a.u]	TAU [s]	MAGNITUDES [a.u]
1.2763e-01	8.2036e+02	1.2084e-01	1.0063e+03	1.2840e-01	1.0057e+03	1.4875e-01	9.0829e+02
8.5601e-02	9.6524e+02	8.1048e-02	1.0392e+03	8.6118e-02	1.2267e+03	9.9765e-02	1.0917e+03
5.7412e-02	1.0700e+03	5.4359e-02	1.3536e+03	5.7759e-02	1.1843e+03	6.6913e-02	1.2595e+03
3.8507e-02	1.4051e+03	3.6459e-02	1.5902e+03	3.8739e-02	1.6337e+03	4.4879e-02	1.6222e+03
2.5826e-02	1.7659e+03	2.4453e-02	2.0668e+03	2.5983e-02	2.0065e+03	3.0100e-02	2.0684e+03
1.7322e-02	2.3016e+03	1.6401e-02	2.3471e+03	1.7427e-02	2.3355e+03	2.0188e-02	2.2609e+03
1.1618e-02	2.4448e+03	1.1000e-02	2.6670e+03	1.1688e-02	2.6138e+03	1.3540e-02	2.5959e+03
7.7921e-03	2.6881e+03	7.3776e-03	2.6833e+03	7.8392e-03	2.7400e+03	9.0815e-03	2.8729e+03
5.2262e-03	2.8750e+03	4.9482e-03	2.8368e+03	5.2577e-03	3.0574e+03	6.0910e-03	2.9795e+03
3.5052e-03	2.7827e+03	3.3188e-03	2.9895e+03	3.5264e-03	3.1095e+03	4.0852e-03	3.0024e+03
2.3509e-03	3.1063e+03	2.2259e-03	3.1532e+03	2.3651e-03	3.0934e+03	2.7400e-03	3.2150e+03
1.5768e-03	3.1461e+03	1.4929e-03	3.1102e+03	1.5863e-03	3.1079e+03	1.8377e-03	3.3253e+03
1.0575e-03	3.1914e+03	1.0013e-03	3.2360e+03	1.0639e-03	3.2157e+03	1.2325e-03	3.3013e+03
7.0930e-04	3.2597e+03	6.7157e-04	3.1402e+03	7.1358e-04	3.4010e+03	8.2667e-04	3.2717e+03
4.7573e-04	3.2775e+03	4.5043e-04	3.2169e+03	4.7860e-04	3.3489e+03	5.5445e-04	3.2891e+03
3.1907e-04	3.1572e+03	3.0210e-04	3.3342e+03	3.2100e-04	3.2523e+03	3.7187e-04	3.4241e+03

FREQUENCY [Hz]: 5.3569E6		5.3828E6		5.825E6		6.3327E6	
TAU [s]	MAGNITUDES [a.u]	TAU [s]	MAGNITUDES [a.u]	TAU [s]	MAGNITUDES [a.u]	TAU [s]	MAGNITUDES [a.u]
1.7042e-01	9.6933e+02	1.2615e-01	9.7324e+02	1.6598e-01	1.0403e+03	1.8163e-01	1.0674e+03
1.1430e-01	1.0631e+03	8.4607e-02	1.2875e+03	1.1132e-01	1.1158e+03	1.2182e-01	1.2313e+03
7.6662e-02	1.3807e+03	5.6746e-02	1.5965e+03	7.4663e-02	1.3363e+03	8.1705e-02	1.3644e+03
5.1418e-02	1.5638e+03	3.8060e-02	1.7911e+03	5.0077e-02	1.6711e+03	5.4800e-02	1.6734e+03
3.4486e-02	1.8422e+03	2.5527e-02	2.1851e+03	3.3587e-02	2.0785e+03	3.6754e-02	2.0360e+03
2.3130e-02	2.1396e+03	1.7121e-02	2.4213e+03	2.2527e-02	2.3265e+03	2.4651e-02	2.2237e+03
1.5513e-02	2.2651e+03	1.1483e-02	2.7432e+03	1.5109e-02	2.5878e+03	1.6534e-02	2.5762e+03
1.0405e-02	2.7630e+03	7.7016e-03	3.0080e+03	1.0133e-02	2.8351e+03	1.1089e-02	2.6765e+03
6.9784e-03	2.8444e+03	5.1655e-03	3.0999e+03	6.7965e-03	2.8005e+03	7.4375e-03	3.0449e+03
4.6805e-03	3.0679e+03	3.4645e-03	3.2882e+03	4.5584e-03	3.0882e+03	4.9883e-03	3.0999e+03
3.1392e-03	3.0569e+03	2.3236e-03	3.2498e+03	3.0573e-03	3.2223e+03	3.3457e-03	3.1046e+03
2.1055e-03	3.1266e+03	1.5585e-03	3.1882e+03	2.0506e-03	3.2808e+03	2.2440e-03	3.0922e+03
1.4121e-03	3.2932e+03	1.0453e-03	3.4335e+03	1.3753e-03	3.1485e+03	1.5050e-03	3.2640e+03
9.4712e-04	3.3029e+03	7.0106e-04	3.3715e+03	9.2242e-04	3.1828e+03	1.0094e-03	3.3120e+03
6.3524e-04	3.3725e+03	4.7020e-04	3.5144e+03	6.1867e-04	3.2506e+03	6.7702e-04	3.2886e+03
4.2605e-04	3.2762e+03	3.1537e-04	3.2255e+03	4.1494e-04	3.2551e+03	4.5408e-04	3.1646e+03

FREQUENCY [Hz]: 6.8868E6		7.486E6		8.1419E6		8.854E6	
TAU [s]	MAGNITUDES [a.u]	TAU [s]	MAGNITUDES [a.u]	TAU [s]	MAGNITUDES [a.u]	TAU [s]	MAGNITUDES [a.u]
1.7610e-01	1.1482e+03	1.7999e-01	1.2164e+03	2.0504e-01	1.4418e+03	2.2341e-01	1.4496e+03
1.1811e-01	1.1944e+03	1.2072e-01	1.4209e+03	1.3752e-01	1.4623e+03	1.4984e-01	1.5485e+03
7.9218e-02	1.4266e+03	8.0967e-02	1.5330e+03	9.2236e-02	1.6434e+03	1.0050e-01	1.6849e+03
5.3132e-02	1.7441e+03	5.4304e-02	1.9117e+03	6.1863e-02	1.9850e+03	6.7405e-02	2.0006e+03
3.5636e-02	2.1541e+03	3.6422e-02	2.1259e+03	4.1491e-02	2.0765e+03	4.5209e-02	2.2202e+03
2.3901e-02	2.3251e+03	2.4428e-02	2.3937e+03	2.7828e-02	2.5144e+03	3.0321e-02	2.4769e+03
1.6030e-02	2.6468e+03	1.6384e-02	2.7177e+03	1.8665e-02	2.8273e+03	2.0337e-02	2.7071e+03
1.0752e-02	2.9601e+03	1.0989e-02	2.8736e+03	1.2518e-02	2.8859e+03	1.3640e-02	2.9592e+03
7.2111e-03	3.0367e+03	7.3702e-03	3.0510e+03	8.3961e-03	3.0187e+03	9.1483e-03	2.9622e+03
4.8365e-03	2.9975e+03	4.9432e-03	3.1889e+03	5.6313e-03	3.2552e+03	6.1358e-03	3.1000e+03
3.2438e-03	3.2658e+03	3.3154e-03	3.0592e+03	3.7769e-03	3.2166e+03	4.1153e-03	3.2879e+03
2.1756e-03	3.1247e+03	2.2237e-03	3.1805e+03	2.5332e-03	3.2840e+03	2.7601e-03	3.2576e+03
1.4592e-03	3.3087e+03	1.4914e-03	3.2995e+03	1.6990e-03	3.2753e+03	1.8512e-03	3.2469e+03
9.7869e-04	3.1692e+03	1.0003e-03	3.3152e+03	1.1395e-03	3.5115e+03	1.2416e-03	3.3704e+03
6.5641e-04	3.3079e+03	6.7090e-04	3.2494e+03	7.6428e-04	3.3481e+03	8.3275e-04	3.2210e+03
4.4026e-04	3.3295e+03	4.4997e-04	3.4885e+03	5.1260e-04	3.3547e+03	5.5853e-04	3.3942e+03

FREQUENCY [Hz]: 9.6255E6		1.1384E7		1.238E7		1.3458E7	
TAU [s]	MAGNITUDES [a.u]	TAU [s]	MAGNITUDES [a.u]	TAU [s]	MAGNITUDES [a.u]	TAU [s]	MAGNITUDES [a.u]
4.6773e-01	1.5258e+03	2.9252e-01	1.7274e+03	3.4698e-01	1.8608e+03	3.4066e-01	2.0475e+03
3.1371e-01	1.4926e+03	1.9619e-01	1.6278e+03	2.3272e-01	1.8950e+03	2.2848e-01	1.9093e+03
2.1041e-01	1.5063e+03	1.3159e-01	1.4466e+03	1.5608e-01	1.7087e+03	1.5324e-01	1.7915e+03
1.4112e-01	1.7286e+03	8.8256e-02	1.3389e+03	1.0469e-01	1.6287e+03	1.0278e-01	1.4344e+03
9.4649e-02	1.7270e+03	5.9193e-02	1.1785e+03	7.0213e-02	1.3103e+03	6.8935e-02	1.3810e+03
6.3481e-02	2.1743e+03	3.9701e-02	1.1248e+03	4.7092e-02	1.0145e+03	4.6235e-02	1.0647e+03
4.2577e-02	2.4834e+03	2.6628e-02	8.2546e+02	3.1585e-02	9.6767e+02	3.1010e-02	9.7451e+02
2.8556e-02	2.7061e+03	1.7859e-02	7.0928e+02	2.1184e-02	8.7582e+02	2.0798e-02	8.9889e+02
1.9153e-02	2.8305e+03	1.1978e-02	7.3540e+02	1.4208e-02	6.3591e+02	1.3950e-02	6.3472e+02
1.2846e-02	2.8889e+03	8.0338e-03	5.8569e+02	9.5294e-03	6.9498e+02	9.3560e-03	4.8524e+02
8.6157e-03	3.0906e+03	5.3883e-03	4.9299e+02	6.3914e-03	5.2773e+02	6.2751e-03	4.4034e+02
5.7786e-03	3.2430e+03	3.6139e-03	4.9034e+02	4.2867e-03	4.9388e+02	4.2087e-03	4.4899e+02
3.8757e-03	3.3099e+03	2.4239e-03	3.9561e+02	2.8751e-03	4.9582e+02	2.8228e-03	5.6305e+02
2.5994e-03	3.3684e+03	1.6257e-03	4.9024e+02	1.9283e-03	4.3570e+02	1.8932e-03	5.3258e+02
1.7435e-03	3.5152e+03	1.0903e-03	4.7252e+02	1.2933e-03	4.2346e+02	1.2698e-03	4.3325e+02
1.1693e-03	3.3275e+03	7.3130e-04	4.1292e+02	8.6744e-04	4.2213e+02	8.5166e-04	4.1171e+02

FREQUENCY [Hz]: 1.4637E7		1.5916E7		1.7309E7		1.8822E7	
TAU [s]	MAGNITUDES [a.u]	TAU [s]	MAGNITUDES [a.u]	TAU [s]	MAGNITUDES [a.u]	TAU [s]	MAGNITUDES [a.u]
3.4950e-01	2.0777e+03	3.5816e-01	2.2589e+03	3.8997e-01	2.4465e+03	4.2746e-01	2.7000e+03
2.3441e-01	2.0197e+03	2.4022e-01	2.2041e+03	2.6155e-01	2.3523e+03	2.8670e-01	2.6205e+03
1.5722e-01	1.7711e+03	1.6111e-01	1.9697e+03	1.7542e-01	2.2214e+03	1.9229e-01	2.4129e+03
1.0545e-01	1.6321e+03	1.0806e-01	1.7222e+03	1.1766e-01	1.8917e+03	1.2897e-01	2.1604e+03
7.0724e-02	1.4986e+03	7.2475e-02	1.5252e+03	7.8913e-02	1.6364e+03	8.6499e-02	1.8196e+03
4.7434e-02	1.0577e+03	4.8609e-02	1.2090e+03	5.2927e-02	1.3035e+03	5.8015e-02	1.3558e+03
3.1814e-02	8.5842e+02	3.2602e-02	9.0552e+02	3.5498e-02	1.1335e+03	3.8911e-02	1.1995e+03
2.1338e-02	6.7628e+02	2.1866e-02	7.8051e+02	2.3809e-02	8.0967e+02	2.6098e-02	9.2040e+02
1.4311e-02	6.1838e+02	1.4666e-02	7.1360e+02	1.5969e-02	6.6898e+02	1.7504e-02	7.1490e+02
9.5987e-03	5.6721e+02	9.8364e-03	6.4601e+02	1.0710e-02	6.4084e+02	1.1740e-02	5.7849e+02
6.4379e-03	4.3945e+02	6.5973e-03	5.1797e+02	7.1833e-03	5.2486e+02	7.8739e-03	6.1601e+02
4.3179e-03	4.5531e+02	4.4248e-03	4.9510e+02	4.8179e-03	4.2825e+02	5.2810e-03	4.3645e+02
2.8960e-03	5.2005e+02	2.9677e-03	4.7539e+02	3.2313e-03	4.3170e+02	3.5420e-03	4.5828e+02
1.9424e-03	4.5971e+02	1.9905e-03	4.1056e+02	2.1673e-03	4.7241e+02	2.3756e-03	4.6003e+02
1.3027e-03	3.7931e+02	1.3350e-03	3.5451e+02	1.4536e-03	4.3817e+02	1.5933e-03	4.5483e+02
8.7375e-04	3.6242e+02	8.9539e-04	4.0781e+02	9.7493e-04	4.6438e+02	1.0687e-03	4.5458e+02

FREQUENCY [Hz]: 2.0463E7		2.2248E7		2.6313E7		2.8613E7	
TAU [s]	MAGNITUDES [a.u]	TAU [s]	MAGNITUDES [a.u]	TAU [s]	MAGNITUDES [a.u]	TAU [s]	MAGNITUDES [a.u]
4.1698e-01	2.9587e+03	4.9079e-01	3.0029e+03	4.8768e-01	3.4133e+03	4.5674e-01	3.8332e+03
2.7967e-01	2.5860e+03	3.2917e-01	2.9872e+03	3.2709e-01	3.3460e+03	3.0634e-01	3.5752e+03
1.8757e-01	2.4969e+03	2.2078e-01	2.7835e+03	2.1938e-01	3.0781e+03	2.0546e-01	3.2155e+03
1.2581e-01	2.0030e+03	1.4808e-01	2.3896e+03	1.4714e-01	2.7289e+03	1.3780e-01	2.8192e+03
8.4378e-02	1.7542e+03	9.9314e-02	2.1131e+03	9.8686e-02	2.2588e+03	9.2425e-02	2.2313e+03
5.6593e-02	1.4958e+03	6.6610e-02	1.6382e+03	6.6189e-02	1.8437e+03	6.1989e-02	1.7718e+03
3.7957e-02	1.2624e+03	4.4676e-02	1.3029e+03	4.4393e-02	1.4267e+03	4.1576e-02	1.4053e+03
2.5458e-02	1.0999e+03	2.9964e-02	9.8855e+02	2.9774e-02	1.0580e+03	2.7885e-02	1.0829e+03
1.7075e-02	8.3471e+02	2.0097e-02	9.6878e+02	1.9970e-02	9.5238e+02	1.8703e-02	8.7358e+02
1.1452e-02	6.7579e+02	1.3479e-02	7.2107e+02	1.3394e-02	7.5483e+02	1.2544e-02	7.3093e+02
7.6808e-03	5.5414e+02	9.0404e-03	5.9468e+02	8.9832e-03	6.0934e+02	8.4133e-03	6.2572e+02
5.1515e-03	4.8579e+02	6.0634e-03	5.7765e+02	6.0250e-03	5.8808e+02	5.6428e-03	4.8686e+02
3.4551e-03	5.1023e+02	4.0667e-03	4.8980e+02	4.0410e-03	5.5102e+02	3.7846e-03	4.7362e+02
2.3174e-03	5.4398e+02	2.7276e-03	4.6602e+02	2.7103e-03	4.8379e+02	2.5384e-03	4.7282e+02
1.5543e-03	4.1188e+02	1.8294e-03	4.9407e+02	1.8178e-03	4.4312e+02	1.7025e-03	4.7176e+02
1.0424e-03	4.3815e+02	1.2270e-03	4.4477e+02	1.2192e-03	4.7131e+02	1.1419e-03	4.0639e+02

FREQUENCY [Hz]: 3.1108E7		3.3828E7		3.6788E7		4.0003E7	
TAU [s]	MAGNITUDES [a.u]	TAU [s]	MAGNITUDES [a.u]	TAU [s]	MAGNITUDES [a.u]	TAU [s]	MAGNITUDES [a.u]
4.9669e-01	4.0908e+03	5.2123e-01	4.4940e+03	5.2035e-01	4.7401e+03	5.2000e-01	4.9745e+03
3.3313e-01	3.9859e+03	3.4959e-01	4.2231e+03	3.4900e-01	4.5527e+03	3.4876e-01	4.8020e+03
2.2343e-01	3.4980e+03	2.3447e-01	3.8547e+03	2.3407e-01	4.1255e+03	2.3392e-01	4.3542e+03
1.4986e-01	3.1496e+03	1.5726e-01	3.3529e+03	1.5699e-01	3.5775e+03	1.5689e-01	3.6541e+03
1.0051e-01	2.6995e+03	1.0547e-01	2.7678e+03	1.0530e-01	2.8555e+03	1.0523e-01	2.9285e+03
6.7411e-02	2.0213e+03	7.0741e-02	2.2218e+03	7.0622e-02	2.2253e+03	7.0575e-02	2.3173e+03
4.5213e-02	1.5159e+03	4.7446e-02	1.6904e+03	4.7366e-02	1.6563e+03	4.7335e-02	1.8327e+03
3.0324e-02	1.1458e+03	3.1822e-02	1.3457e+03	3.1769e-02	1.3572e+03	3.1747e-02	1.3940e+03
2.0338e-02	9.3993e+02	2.1343e-02	9.3710e+02	2.1307e-02	1.0898e+03	2.1293e-02	1.1222e+03
1.3641e-02	7.1471e+02	1.4315e-02	7.2349e+02	1.4291e-02	7.5518e+02	1.4281e-02	7.8270e+02
9.1491e-03	5.2012e+02	9.6011e-03	5.8441e+02	9.5849e-03	6.0856e+02	9.5785e-03	6.2522e+02
6.1363e-03	5.9371e+02	6.4395e-03	5.9883e+02	6.4286e-03	5.7023e+02	6.4243e-03	5.0572e+02
4.1156e-03	3.6181e+02	4.3190e-03	5.4863e+02	4.3117e-03	5.6765e+02	4.3088e-03	4.9971e+02
2.7604e-03	4.8691e+02	2.8967e-03	5.3082e+02	2.8918e-03	4.8349e+02	2.8899e-03	4.3112e+02
1.8514e-03	4.1660e+02	1.9428e-03	4.4645e+02	1.9396e-03	3.8189e+02	1.9383e-03	4.7665e+02
1.2417e-03	4.3710e+02	1.3031e-03	4.4719e+02	1.3009e-03	5.4313e+02	1.3000e-03	4.1375e+02

2. ^{1}H **magnetization of AHP**:

FREQUENCY [Hz]: 26266		32844		36409		40799	
TAU [s]	MAGNITUDES [a.u]	TAU [s]	MAGNITUDES [a.u]	TAU [s]	MAGNITUDES [a.u]	TAU [s]	MAGNITUDES [a.u]
3.0655e-03	3.1423e+02	2.6584e-03	3.1698e+02	3.1302e-03	2.8810e+02	4.3579e-03	2.9731e+02
2.9668e-03	3.1448e+02	2.5728e-03	3.1057e+02	3.0294e-03	4.0079e+02	4.2177e-03	3.9428e+02
2.8682e-03	3.3839e+02	2.4873e-03	3.3262e+02	2.9287e-03	3.6624e+02	4.0775e-03	3.0004e+02
2.7696e-03	3.3329e+02	2.4017e-03	3.7906e+02	2.8280e-03	4.3701e+02	3.9372e-03	3.5015e+02
2.6709e-03	2.7892e+02	2.3162e-03	3.3830e+02	2.7273e-03	4.6289e+02	3.7970e-03	2.8137e+02
2.5723e-03	4.4143e+02	2.2307e-03	3.6175e+02	2.6266e-03	3.4916e+02	3.6568e-03	3.4527e+02
2.4736e-03	2.7272e+02	2.1451e-03	3.7219e+02	2.5258e-03	3.4782e+02	3.5166e-03	3.4339e+02
2.3750e-03	3.0425e+02	2.0596e-03	3.4579e+02	2.4251e-03	3.3851e+02	3.3763e-03	3.1428e+02
2.2764e-03	2.9346e+02	1.9741e-03	2.8455e+02	2.3244e-03	3.6134e+02	3.2361e-03	3.1041e+02
2.1777e-03	3.9178e+02	1.8885e-03	3.2215e+02	2.2237e-03	3.5089e+02	3.0959e-03	3.6815e+02
2.0791e-03	3.8882e+02	1.8030e-03	4.5739e+02	2.1230e-03	3.9690e+02	2.9556e-03	3.2876e+02
1.9804e-03	3.5468e+02	1.7174e-03	3.3235e+02	2.0222e-03	3.9929e+02	2.8154e-03	3.9057e+02
1.8818e-03	3.8141e+02	1.6319e-03	3.9562e+02	1.9215e-03	3.8988e+02	2.6752e-03	3.2965e+02
1.7832e-03	3.6639e+02	1.5464e-03	3.6026e+02	1.8208e-03	3.4757e+02	2.5350e-03	4.3774e+02
1.6845e-03	3.7475e+02	1.4608e-03	3.6001e+02	1.7201e-03	3.9143e+02	2.3947e-03	3.1480e+02
1.5859e-03	4.1272e+02	1.3753e-03	3.4440e+02	1.6193e-03	3.6498e+02	2.2545e-03	3.3163e+02
1.4872e-03	3.7812e+02	1.2897e-03	4.6839e+02	1.5186e-03	3.8983e+02	2.1143e-03	3.3522e+02
1.3886e-03	4.0446e+02	1.2042e-03	4.2598e+02	1.4179e-03	4.0789e+02	1.9741e-03	3.8868e+02
1.2900e-03	4.0674e+02	1.1187e-03	4.7367e+02	1.3172e-03	4.4491e+02	1.8338e-03	3.9062e+02
1.1913e-03	3.8276e+02	1.0331e-03	3.8415e+02	1.2165e-03	4.1301e+02	1.6936e-03	4.3016e+02
1.0927e-03	3.9916e+02	9.4758e-04	4.1146e+02	1.1157e-03	4.0461e+02	1.5534e-03	3.6728e+02
9.9405e-04	3.7682e+02	8.6204e-04	4.7379e+02	1.0150e-03	5.7478e+02	1.4132e-03	3.4418e+02
8.9541e-04	4.4163e+02	7.7650e-04	4.3307e+02	9.1431e-04	5.1767e+02	1.2729e-03	3.9203e+02
7.9677e-04	4.8850e+02	6.9096e-04	4.5011e+02	8.1359e-04	5.0071e+02	1.1327e-03	4.4847e+02
6.9814e-04	4.2552e+02	6.0542e-04	5.2493e+02	7.1287e-04	4.9475e+02	9.9248e-04	4.1631e+02
5.9950e-04	5.5244e+02	5.1988e-04	5.8560e+02	6.1215e-04	5.6751e+02	8.5225e-04	5.0057e+02
5.0086e-04	5.7700e+02	4.3434e-04	5.1643e+02	5.1143e-04	5.3693e+02	7.1203e-04	5.5178e+02
4.0222e-04	5.9296e+02	3.4880e-04	5.7135e+02	4.1071e-04	6.1712e+02	5.7180e-04	5.2488e+02
3.0358e-04	6.4090e+02	2.6326e-04	5.8145e+02	3.0999e-04	5.6058e+02	4.3157e-04	6.1768e+02
2.0494e-04	5.9932e+02	1.7772e-04	6.6160e+02	2.0927e-04	7.2195e+02	2.9135e-04	6.6454e+02
1.0630e-04	6.0342e+02	9.2185e-05	7.0013e+02	1.0855e-04	6.5835e+02	1.5112e-04	6.8481e+02
7.6637e-06	7.7523e+02	6.6459e-06	7.3660e+02	7.8254e-06	7.9457e+02	1.0895e-05	7.1613e+02

FREQUENCY [Hz]: 45440		51049		56984		63647	
TAU [s]	MAGNITUDES [a.u]	TAU [s]	MAGNITUDES [a.u]	TAU [s]	MAGNITUDES [a.u]	TAU [s]	MAGNITUDES [a.u]
3.8370e-03	3.3301e+02	4.4163e-03	3.4337e+02	5.1551e-03	3.6301e+02	5.8256e-03	3.7677e+02
3.7135e-03	3.4541e+02	4.2742e-03	3.1895e+02	4.9892e-03	3.2697e+02	5.6382e-03	2.7327e+02
3.5900e-03	3.7433e+02	4.1321e-03	3.7237e+02	4.8233e-03	3.6025e+02	5.4507e-03	3.1387e+02
3.4666e-03	3.0750e+02	3.9900e-03	3.4829e+02	4.6574e-03	3.6468e+02	5.2633e-03	3.8462e+02
3.3431e-03	3.7021e+02	3.8479e-03	3.5818e+02	4.4916e-03	3.6329e+02	5.0758e-03	3.4379e+02
3.2196e-03	3.4762e+02	3.7058e-03	3.4911e+02	4.3257e-03	3.8054e+02	4.8884e-03	2.8081e+02
3.0962e-03	3.2906e+02	3.5637e-03	3.5808e+02	4.1598e-03	3.1181e+02	4.7009e-03	3.2252e+02
2.9727e-03	3.5092e+02	3.4215e-03	3.3424e+02	3.9939e-03	2.8845e+02	4.5135e-03	2.7068e+02
2.8493e-03	3.4920e+02	3.2794e-03	3.0265e+02	3.8280e-03	3.6145e+02	4.3260e-03	3.4859e+02
2.7258e-03	3.2067e+02	3.1373e-03	3.4278e+02	3.6622e-03	3.5793e+02	4.1386e-03	3.4779e+02
2.6023e-03	3.4817e+02	2.9952e-03	3.2397e+02	3.4963e-03	3.5395e+02	3.9511e-03	3.3061e+02
2.4789e-03	3.5664e+02	2.8531e-03	3.8771e+02	3.3304e-03	3.5732e+02	3.7636e-03	3.0368e+02
2.3554e-03	3.9361e+02	2.7110e-03	3.4144e+02	3.1645e-03	3.6345e+02	3.5762e-03	3.3132e+02
2.2319e-03	3.8678e+02	2.5689e-03	4.2420e+02	2.9987e-03	3.1066e+02	3.3887e-03	3.5111e+02
2.1085e-03	4.4623e+02	2.4268e-03	3.5432e+02	2.8328e-03	3.5560e+02	3.2013e-03	3.2351e+02
1.9850e-03	3.8868e+02	2.2847e-03	3.8413e+02	2.6669e-03	3.8688e+02	3.0138e-03	4.4225e+02
1.8615e-03	4.4980e+02	2.1426e-03	3.2729e+02	2.5010e-03	4.1583e+02	2.8264e-03	3.4927e+02
1.7381e-03	4.0205e+02	2.0005e-03	4.2410e+02	2.3352e-03	4.2406e+02	2.6389e-03	4.1566e+02
1.6146e-03	3.9410e+02	1.8584e-03	4.5659e+02	2.1693e-03	3.6941e+02	2.4515e-03	3.7592e+02
1.4912e-03	4.3563e+02	1.7163e-03	3.8967e+02	2.0034e-03	4.1733e+02	2.2640e-03	4.3132e+02
1.3677e-03	3.9580e+02	1.5742e-03	4.3810e+02	1.8375e-03	4.5158e+02	2.0766e-03	3.8875e+02
1.2442e-03	4.3452e+02	1.4321e-03	4.2273e+02	1.6717e-03	4.3700e+02	1.8891e-03	4.3842e+02
1.1208e-03	4.7809e+02	1.2900e-03	4.4828e+02	1.5058e-03	4.3711e+02	1.7016e-03	4.9750e+02
9.9730e-04	4.5015e+02	1.1479e-03	4.5491e+02	1.3399e-03	4.3800e+02	1.5142e-03	4.8454e+02
8.7384e-04	4.5768e+02	1.0058e-03	4.8767e+02	1.1740e-03	4.2125e+02	1.3267e-03	4.2718e+02
7.5037e-04	5.2509e+02	8.6367e-04	5.1876e+02	1.0081e-03	5.7533e+02	1.1393e-03	5.6469e+02
6.2691e-04	5.7379e+02	7.2156e-04	5.8155e+02	8.4227e-04	5.5495e+02	9.5183e-04	5.3107e+02
5.0345e-04	5.4607e+02	5.7946e-04	5.6501e+02	6.7639e-04	5.6578e+02	7.6438e-04	5.7544e+02
3.7998e-04	6.5437e+02	4.3735e-04	5.4384e+02	5.1052e-04	6.1552e+02	5.7693e-04	6.3221e+02
2.5652e-04	5.9718e+02	2.9525e-04	6.8346e+02	3.4464e-04	6.5900e+02	3.8947e-04	6.0073e+02
1.3306e-04	6.4691e+02	1.5315e-04	6.6023e+02	1.7876e-04	7.2501e+02	2.0202e-04	7.0792e+02
9.5924e-06	7.2005e+02	1.1041e-05	7.5668e+02	1.2888e-05	7.9779e+02	1.4564e-05	8.4907e+02

FREQUENCY [Hz]: 71104		79426		88682		98946	
TAU [s]	MAGNITUDES [a.u]	TAU [s]	MAGNITUDES [a.u]	TAU [s]	MAGNITUDES [a.u]	TAU [s]	MAGNITUDES [a.u]
5.5515e-03	3.2769e+02	5.7331e-03	3.2452e+02	6.3722e-03	3.0701e+02	5.7168e-03	3.3997e+02
5.3728e-03	3.7009e+02	5.5486e-03	3.2697e+02	6.1672e-03	3.0264e+02	5.5329e-03	2.9909e+02
5.1942e-03	3.4301e+02	5.3641e-03	3.4497e+02	5.9621e-03	3.1235e+02	5.3489e-03	3.4721e+02
5.0156e-03	3.7419e+02	5.1797e-03	3.4848e+02	5.7571e-03	3.2309e+02	5.1650e-03	3.0593e+02
4.8369e-03	3.5902e+02	4.9952e-03	3.9349e+02	5.5520e-03	3.4801e+02	4.9810e-03	3.5821e+02
4.6583e-03	3.4081e+02	4.8107e-03	4.1675e+02	5.3470e-03	2.9231e+02	4.7971e-03	3.5474e+02
4.4797e-03	3.5585e+02	4.6262e-03	3.6113e+02	5.1420e-03	3.4792e+02	4.6131e-03	3.6272e+02
4.3010e-03	3.4037e+02	4.4417e-03	4.0762e+02	4.9369e-03	3.4756e+02	4.4292e-03	3.2999e+02
4.1224e-03	3.1048e+02	4.2573e-03	3.5470e+02	4.7319e-03	3.6619e+02	4.2452e-03	3.6575e+02
3.9438e-03	3.6112e+02	4.0728e-03	4.2727e+02	4.5268e-03	3.6423e+02	4.0613e-03	3.6463e+02
3.7651e-03	4.1884e+02	3.8883e-03	4.1730e+02	4.3218e-03	3.5282e+02	3.8773e-03	4.2574e+02
3.5865e-03	4.2930e+02	3.7038e-03	4.1970e+02	4.1168e-03	3.4463e+02	3.6934e-03	3.2720e+02
3.4079e-03	3.7560e+02	3.5194e-03	3.1818e+02	3.9117e-03	3.4777e+02	3.5094e-03	4.0444e+02
3.2292e-03	3.3591e+02	3.3349e-03	3.9819e+02	3.7067e-03	3.3167e+02	3.3254e-03	4.2692e+02
3.0506e-03	3.3300e+02	3.1504e-03	4.6135e+02	3.5016e-03	3.7810e+02	3.1415e-03	4.6507e+02
2.8720e-03	3.7195e+02	2.9659e-03	3.6946e+02	3.2966e-03	3.6184e+02	2.9575e-03	4.0063e+02
2.6934e-03	4.3442e+02	2.7815e-03	4.0807e+02	3.0915e-03	4.1384e+02	2.7736e-03	4.0751e+02
2.5147e-03	4.5297e+02	2.5970e-03	4.6674e+02	2.8865e-03	4.1582e+02	2.5896e-03	3.9226e+02
2.3361e-03	4.0180e+02	2.4125e-03	4.3747e+02	2.6815e-03	3.4014e+02	2.4057e-03	3.9371e+02
2.1575e-03	4.8766e+02	2.2280e-03	4.7967e+02	2.4764e-03	4.4417e+02	2.2217e-03	4.7201e+02
1.9788e-03	3.8580e+02	2.0436e-03	4.8782e+02	2.2714e-03	3.8280e+02	2.0378e-03	5.2975e+02
1.8002e-03	4.4890e+02	1.8591e-03	4.1350e+02	2.0663e-03	4.3392e+02	1.8538e-03	5.2375e+02
1.6216e-03	4.9481e+02	1.6746e-03	5.0492e+02	1.8613e-03	4.7825e+02	1.6699e-03	4.9849e+02
1.4429e-03	4.4314e+02	1.4901e-03	5.8135e+02	1.6563e-03	4.7981e+02	1.4859e-03	5.2663e+02
1.2643e-03	5.1959e+02	1.3057e-03	5.2605e+02	1.4512e-03	4.3205e+02	1.3020e-03	5.7415e+02
1.0857e-03	5.1226e+02	1.1212e-03	4.7792e+02	1.2462e-03	4.7955e+02	1.1180e-03	5.4016e+02
9.0704e-04	5.5458e+02	9.3671e-04	6.2570e+02	1.0411e-03	5.0904e+02	9.3406e-04	6.2952e+02
7.2840e-04	5.8795e+02	7.5224e-04	5.8824e+02	8.3609e-04	6.1646e+02	7.5010e-04	6.1012e+02
5.4977e-04	6.1155e+02	5.6776e-04	6.6992e+02	6.3105e-04	5.9888e+02	5.6615e-04	7.3485e+02
3.7114e-04	6.1405e+02	3.8328e-04	6.8489e+02	4.2601e-04	6.8908e+02	3.8220e-04	7.0036e+02
1.9251e-04	7.3797e+02	1.9881e-04	8.2950e+02	2.2097e-04	6.7785e+02	1.9825e-04	7.6215e+02
1.3879e-05	7.3391e+02	1.4333e-05	7.9695e+02	1.5931e-05	8.2241e+02	1.4292e-05	7.6280e+02

FREQUENCY [Hz]: 110310		123400		137820		171690	
TAU [s]	MAGNITUDES [a.u]	TAU [s]	MAGNITUDES [a.u]	TAU [s]	MAGNITUDES [a.u]	TAU [s]	MAGNITUDES [a.u]
7.8943e-03	3.8534e+02	7.1349e-03	3.6876e+02	7.6634e-03	3.4441e+02	1.0679e-02	3.2167e+02
7.6403e-03	3.7781e+02	6.9053e-03	3.8447e+02	7.4168e-03	3.2046e+02	1.0336e-02	3.4395e+02
7.3863e-03	3.4655e+02	6.6757e-03	4.1319e+02	7.1702e-03	3.5403e+02	9.9920e-03	3.3085e+02
7.1323e-03	3.9488e+02	6.4462e-03	3.4427e+02	6.9236e-03	3.5657e+02	9.6484e-03	2.4491e+02
6.8782e-03	3.3356e+02	6.2166e-03	3.5613e+02	6.6771e-03	3.6377e+02	9.3047e-03	3.5846e+02
6.6242e-03	3.5214e+02	5.9870e-03	3.7600e+02	6.4305e-03	2.8301e+02	8.9611e-03	3.1516e+02
6.3702e-03	2.9843e+02	5.7574e-03	3.5429e+02	6.1839e-03	3.7740e+02	8.6175e-03	4.2333e+02
6.1162e-03	4.0339e+02	5.5278e-03	3.9741e+02	5.9373e-03	3.5594e+02	8.2738e-03	3.5709e+02
5.8622e-03	3.1056e+02	5.2982e-03	3.6018e+02	5.6907e-03	4.1924e+02	7.9302e-03	3.7072e+02
5.6082e-03	3.3867e+02	5.0687e-03	3.8097e+02	5.4441e-03	3.7099e+02	7.5866e-03	4.0515e+02
5.3541e-03	3.0550e+02	4.8391e-03	3.7526e+02	5.1975e-03	3.7876e+02	7.2429e-03	3.3229e+02
5.1001e-03	3.1462e+02	4.6095e-03	3.3885e+02	4.9509e-03	3.8379e+02	6.8993e-03	2.8124e+02
4.8461e-03	3.4160e+02	4.3799e-03	4.2010e+02	4.7043e-03	3.2306e+02	6.5557e-03	3.3597e+02
4.5921e-03	3.8966e+02	4.1503e-03	3.6098e+02	4.4578e-03	3.5965e+02	6.2120e-03	3.5234e+02
4.3381e-03	3.9777e+02	3.9207e-03	4.3174e+02	4.2112e-03	3.8464e+02	5.8684e-03	3.4616e+02
4.0840e-03	4.0137e+02	3.6912e-03	3.8597e+02	3.9646e-03	3.4908e+02	5.5248e-03	3.4667e+02
3.8300e-03	4.4878e+02	3.4616e-03	4.4840e+02	3.7180e-03	4.5251e+02	5.1812e-03	3.7895e+02
3.5760e-03	3.6891e+02	3.2320e-03	4.3774e+02	3.4714e-03	4.3294e+02	4.8375e-03	3.8027e+02
3.3220e-03	4.0513e+02	3.0024e-03	4.6425e+02	3.2248e-03	4.8522e+02	4.4939e-03	4.1503e+02
3.0680e-03	4.0850e+02	2.7728e-03	4.8245e+02	2.9782e-03	5.2942e+02	4.1503e-03	3.8797e+02
2.8139e-03	4.0165e+02	2.5432e-03	5.1765e+02	2.7316e-03	4.9423e+02	3.8066e-03	4.3342e+02
2.5599e-03	5.1071e+02	2.3137e-03	4.8566e+02	2.4850e-03	4.7751e+02	3.4630e-03	4.2261e+02
2.3059e-03	4.5350e+02	2.0841e-03	4.7353e+02	2.2385e-03	4.6172e+02	3.1194e-03	4.6992e+02
2.0519e-03	4.6126e+02	1.8545e-03	6.0107e+02	1.9919e-03	5.0329e+02	2.7757e-03	5.1902e+02
1.7979e-03	6.2263e+02	1.6249e-03	6.0602e+02	1.7453e-03	5.9722e+02	2.4321e-03	5.2125e+02
1.5438e-03	5.7560e+02	1.3953e-03	5.8931e+02	1.4987e-03	5.8582e+02	2.0885e-03	5.6167e+02
1.2898e-03	5.7457e+02	1.1658e-03	7.1459e+02	1.2521e-03	6.0699e+02	1.7449e-03	6.5230e+02
1.0358e-03	5.8915e+02	9.3617e-04	6.4562e+02	1.0055e-03	7.1203e+02	1.4012e-03	6.7963e+02
7.8179e-04	5.9214e+02	7.0659e-04	7.4277e+02	7.5892e-04	7.8497e+02	1.0576e-03	6.8615e+02
5.2777e-04	7.1913e+02	4.7700e-04	7.9482e+02	5.1234e-04	7.6360e+02	7.1396e-04	7.2804e+02
2.7375e-04	7.0371e+02	2.4742e-04	8.2577e+02	2.6575e-04	8.3494e+02	3.7033e-04	7.6608e+02
1.9736e-05	8.5817e+02	1.7837e-05	8.2476e+02	1.9159e-05	8.8198e+02	2.6698e-05	8.7568e+02

FREQUENCY [Hz]: 192120		214420		267650		299090	
TAU [s]	MAGNITUDES [a.u]	TAU [s]	MAGNITUDES [a.u]	TAU [s]	MAGNITUDES [a.u]	TAU [s]	MAGNITUDES [a.u]
9.3509e-03	3.8144e+02	1.0441e-02	3.2075e+02	1.0403e-02	3.4997e+02	1.4907e-02	3.1569e+02
9.0500e-03	3.7499e+02	1.0105e-02	3.5907e+02	1.0068e-02	3.6348e+02	1.4427e-02	3.4465e+02
8.7491e-03	3.6390e+02	9.7692e-03	3.3710e+02	9.7335e-03	3.9627e+02	1.3948e-02	2.8318e+02
8.4483e-03	3.2803e+02	9.4332e-03	3.5801e+02	9.3987e-03	3.9813e+02	1.3468e-02	4.0361e+02
8.1474e-03	3.6428e+02	9.0972e-03	3.4010e+02	9.0640e-03	3.3328e+02	1.2988e-02	3.5077e+02
7.8465e-03	3.5070e+02	8.7613e-03	3.6160e+02	8.7293e-03	3.3170e+02	1.2509e-02	3.7380e+02
7.5456e-03	4.0712e+02	8.4253e-03	3.6385e+02	8.3945e-03	3.1896e+02	1.2029e-02	3.8605e+02
7.2447e-03	3.7641e+02	8.0893e-03	3.0636e+02	8.0598e-03	4.1065e+02	1.1549e-02	3.1846e+02
6.9438e-03	3.5940e+02	7.7534e-03	3.8677e+02	7.7250e-03	4.0912e+02	1.1070e-02	3.8585e+02
6.6429e-03	3.7631e+02	7.4174e-03	4.0611e+02	7.3903e-03	4.0131e+02	1.0590e-02	3.2740e+02
6.3420e-03	4.5318e+02	7.0814e-03	4.1243e+02	7.0556e-03	3.4972e+02	1.0110e-02	3.5991e+02
6.0411e-03	4.0766e+02	6.7455e-03	4.0798e+02	6.7208e-03	3.4575e+02	9.6305e-03	3.1507e+02
5.7403e-03	3.5689e+02	6.4095e-03	2.7174e+02	6.3861e-03	3.9768e+02	9.1509e-03	3.0477e+02
5.4394e-03	3.8343e+02	6.0735e-03	4.2034e+02	6.0513e-03	4.1254e+02	8.6712e-03	4.0709e+02
5.1385e-03	4.0572e+02	5.7376e-03	3.4553e+02	5.7166e-03	4.3982e+02	8.1915e-03	4.0805e+02
4.8376e-03	4.4575e+02	5.4016e-03	3.9151e+02	5.3819e-03	4.0030e+02	7.7119e-03	3.5188e+02
4.5367e-03	4.2000e+02	5.0656e-03	4.0653e+02	5.0471e-03	4.2475e+02	7.2322e-03	3.7371e+02
4.2358e-03	3.8357e+02	4.7297e-03	3.7716e+02	4.7124e-03	4.2736e+02	6.7526e-03	4.3955e+02
3.9349e-03	4.6174e+02	4.3937e-03	4.1077e+02	4.3776e-03	4.5271e+02	6.2729e-03	4.1706e+02
3.6340e-03	5.4149e+02	4.0577e-03	4.3471e+02	4.0429e-03	5.0791e+02	5.7932e-03	4.4175e+02
3.3331e-03	5.3373e+02	3.7218e-03	4.8049e+02	3.7082e-03	4.5957e+02	5.3136e-03	4.0237e+02
3.0323e-03	5.4021e+02	3.3858e-03	5.4191e+02	3.3734e-03	4.6918e+02	4.8339e-03	4.5670e+02
2.7314e-03	6.2342e+02	3.0498e-03	6.1042e+02	3.0387e-03	5.2368e+02	4.3542e-03	4.9536e+02
2.4305e-03	5.5704e+02	2.7138e-03	5.3158e+02	2.7039e-03	6.1186e+02	3.8746e-03	4.1421e+02
2.1296e-03	5.3850e+02	2.3779e-03	6.3682e+02	2.3692e-03	6.2694e+02	3.3949e-03	6.3899e+02
1.8287e-03	6.3702e+02	2.0419e-03	5.7987e+02	2.0344e-03	6.5854e+02	2.9152e-03	5.9461e+02
1.5278e-03	6.0134e+02	1.7059e-03	6.3054e+02	1.6997e-03	5.8455e+02	2.4356e-03	6.2204e+02
1.2269e-03	6.5684e+02	1.3700e-03	6.8297e+02	1.3650e-03	7.5197e+02	1.9559e-03	6.8321e+02
9.2604e-04	7.8208e+02	1.0340e-03	7.1747e+02	1.0302e-03	8.4381e+02	1.4763e-03	6.5792e+02
6.2515e-04	8.3137e+02	6.9804e-04	8.1767e+02	6.9549e-04	9.0403e+02	9.9659e-04	7.2706e+02
3.2427e-04	8.7091e+02	3.6207e-04	9.3395e+02	3.6075e-04	8.5451e+02	5.1693e-04	8.6381e+02
2.3377e-05	9.0861e+02	2.6103e-05	8.9832e+02	2.6007e-05	9.5347e+02	3.7267e-05	9.2456e+02

FREQUENCY [Hz]: 335040		335960		376940		399820	
TAU [s]	MAGNITUDES [a.u]	TAU [s]	MAGNITUDES [a.u]	TAU [s]	MAGNITUDES [a.u]	TAU [s]	MAGNITUDES [a.u]
1.1379e-02	2.8281e+02	1.5552e-02	3.0645e+02	1.5974e-02	3.4440e+02	1.6956e-02	2.8144e+02
1.1013e-02	2.9383e+02	1.2819e-02	3.7582e+02	1.3167e-02	4.0503e+02	1.6411e-02	2.5711e+02
1.0647e-02	2.7548e+02	1.0566e-02	4.1258e+02	1.0853e-02	3.8294e+02	1.5865e-02	2.7180e+02
1.0281e-02	2.5757e+02	8.7091e-03	3.6166e+02	8.9456e-03	3.7112e+02	1.5319e-02	3.1490e+02
9.9147e-03	3.4518e+02	7.1786e-03	4.0657e+02	7.3735e-03	4.8300e+02	1.4774e-02	3.2113e+02
9.5486e-03	3.0227e+02	5.9170e-03	4.7452e+02	6.0776e-03	3.9327e+02	1.4228e-02	2.7279e+02
9.1824e-03	2.7922e+02	4.8771e-03	5.1164e+02	5.0095e-03	5.2031e+02	1.3682e-02	2.9227e+02
8.8163e-03	2.7821e+02	4.0200e-03	6.1566e+02	4.1291e-03	5.8197e+02	1.3137e-02	2.8934e+02
8.4501e-03	2.7124e+02	3.3135e-03	6.0604e+02	3.4035e-03	5.9077e+02	1.2591e-02	3.0790e+02
8.0839e-03	3.2102e+02	2.7312e-03	6.3727e+02	2.8053e-03	6.2917e+02	1.2046e-02	3.1565e+02
7.7178e-03	2.9931e+02	2.2512e-03	6.3676e+02	2.3123e-03	6.7594e+02	1.1500e-02	3.0180e+02
7.3516e-03	3.5277e+02	1.8556e-03	6.5490e+02	1.9059e-03	7.2226e+02	1.0954e-02	3.4191e+02
6.9855e-03	2.6898e+02	1.5295e-03	7.7012e+02	1.5710e-03	7.7361e+02	1.0409e-02	3.3062e+02
6.6193e-03	2.6824e+02	1.2607e-03	7.1735e+02	1.2949e-03	6.9641e+02	9.8633e-03	3.1959e+02
6.2532e-03	3.2958e+02	1.0391e-03	8.1469e+02	1.0673e-03	7.1258e+02	9.3177e-03	3.2816e+02
5.8870e-03	3.2877e+02	8.5650e-04	8.0229e+02	8.7975e-04	7.9460e+02	8.7721e-03	2.9515e+02
5.5208e-03	3.2461e+02	7.0597e-04	8.0201e+02	7.2514e-04	8.3957e+02	8.2264e-03	2.8281e+02
5.1547e-03	2.7960e+02	5.8190e-04	8.5980e+02	5.9770e-04	8.6074e+02	7.6808e-03	3.1324e+02
4.7885e-03	3.0901e+02	4.7964e-04	8.8221e+02	4.9266e-04	7.6399e+02	7.1352e-03	4.1624e+02
4.4224e-03	3.2495e+02	3.9534e-04	9.2205e+02	4.0608e-04	8.3049e+02	6.5896e-03	4.1139e+02
4.0562e-03	3.0583e+02	3.2587e-04	8.4156e+02	3.3471e-04	9.2251e+02	6.0440e-03	3.8652e+02
3.6900e-03	2.9952e+02	2.6860e-04	9.6781e+02	2.7589e-04	8.9088e+02	5.4984e-03	4.2389e+02
3.3239e-03	3.2920e+02	2.2139e-04	9.3349e+02	2.2740e-04	9.3482e+02	4.9528e-03	4.4311e+02
2.9577e-03	3.2357e+02	1.8248e-04	9.2676e+02	1.8744e-04	9.0514e+02	4.4072e-03	4.6238e+02
2.5916e-03	3.0775e+02	1.5041e-04	9.1831e+02	1.5450e-04	8.2411e+02	3.8616e-03	5.2615e+02
2.2254e-03	3.6923e+02	1.2398e-04	9.0690e+02	1.2735e-04	9.1981e+02	3.3160e-03	6.2703e+02
1.8592e-03	3.4799e+02	1.0219e-04	9.2341e+02	1.0497e-04	8.7759e+02	2.7704e-03	6.8521e+02
1.4931e-03	4.1022e+02	8.4232e-05	9.1593e+02	8.6519e-05	9.3688e+02	2.2248e-03	6.6181e+02
1.1269e-03	3.4825e+02	6.9429e-05	9.0250e+02	7.1314e-05	8.5299e+02	1.6792e-03	7.0746e+02
7.6077e-04	3.7179e+02	5.7227e-05	9.0874e+02	5.8781e-05	9.1562e+02	1.1336e-03	7.4616e+02
3.9461e-04	4.5167e+02	4.7170e-05	9.4325e+02	4.8451e-05	9.2071e+02	5.8799e-04	7.3281e+02
2.8448e-05	3.6884e+02	3.8880e-05	1.0062e+03	3.9936e-05	1.0155e+03	4.2390e-05	9.9420e+02

FREQUENCY [Hz]: 424420		476930		478030		534820	
TAU [s]	MAGNITUDES [a.u]	TAU [s]	MAGNITUDES [a.u]	TAU [s]	MAGNITUDES [a.u]	TAU [s]	MAGNITUDES [a.u]
1.4677e-02	3.5465e+02	1.4925e-02	4.2462e+02	1.7259e-02	3.0908e+02	1.6797e-02	3.8525e+02
1.2097e-02	4.1821e+02	1.2302e-02	3.2713e+02	1.6704e-02	2.8611e+02	1.3845e-02	3.5177e+02
9.9714e-03	3.6694e+02	1.0140e-02	4.0549e+02	1.6148e-02	2.8708e+02	1.1412e-02	3.7967e+02
8.2190e-03	4.2103e+02	8.3580e-03	3.8161e+02	1.5593e-02	2.9986e+02	9.4061e-03	4.8569e+02
6.7746e-03	4.2408e+02	6.8892e-03	4.7065e+02	1.5038e-02	2.5497e+02	7.7530e-03	5.0393e+02
5.5840e-03	4.8010e+02	5.6784e-03	4.6341e+02	1.4482e-02	2.5229e+02	6.3905e-03	4.5179e+02
4.6026e-03	4.9441e+02	4.6805e-03	5.3060e+02	1.3927e-02	3.5949e+02	5.2674e-03	6.0212e+02
3.7938e-03	6.3032e+02	3.8579e-03	6.2425e+02	1.3372e-02	2.9335e+02	4.3417e-03	5.7253e+02
3.1270e-03	6.2663e+02	3.1799e-03	5.9593e+02	1.2816e-02	2.5141e+02	3.5787e-03	6.0135e+02
2.5775e-03	6.4568e+02	2.6211e-03	6.7567e+02	1.2261e-02	3.3260e+02	2.9497e-03	6.4757e+02
2.1245e-03	7.3007e+02	2.1604e-03	6.6221e+02	1.1706e-02	2.9992e+02	2.4314e-03	6.9173e+02
1.7511e-03	7.7392e+02	1.7808e-03	7.9936e+02	1.1150e-02	3.6684e+02	2.0041e-03	6.4361e+02
1.4434e-03	7.6553e+02	1.4678e-03	7.0863e+02	1.0595e-02	3.4680e+02	1.6519e-03	7.8603e+02
1.1897e-03	8.2299e+02	1.2098e-03	7.3484e+02	1.0040e-02	3.2015e+02	1.3616e-03	8.3042e+02
9.8064e-04	8.1756e+02	9.9722e-04	8.0887e+02	9.4842e-03	3.3771e+02	1.1223e-03	8.9417e+02
8.0830e-04	8.3508e+02	8.2197e-04	8.6973e+02	8.9288e-03	3.4767e+02	9.2504e-04	8.9241e+02
6.6624e-04	8.6529e+02	6.7751e-04	8.3964e+02	8.3735e-03	3.6169e+02	7.6247e-04	8.0786e+02
5.4916e-04	8.7515e+02	5.5844e-04	8.8186e+02	7.8181e-03	3.6109e+02	6.2847e-04	8.7760e+02
4.5265e-04	9.2382e+02	4.6030e-04	9.5783e+02	7.2628e-03	3.9853e+02	5.1802e-04	8.5378e+02
3.7310e-04	8.3990e+02	3.7941e-04	8.8561e+02	6.7074e-03	3.9779e+02	4.2698e-04	1.0161e+03
3.0753e-04	8.8279e+02	3.1273e-04	8.3811e+02	6.1521e-03	4.4535e+02	3.5194e-04	9.6091e+02
2.5348e-04	9.3675e+02	2.5777e-04	9.0776e+02	5.5967e-03	4.8250e+02	2.9009e-04	8.8441e+02
2.0893e-04	9.2335e+02	2.1247e-04	9.6846e+02	5.0413e-03	5.0041e+02	2.3911e-04	9.0127e+02
1.7221e-04	9.2448e+02	1.7513e-04	1.0091e+03	4.4860e-03	4.6462e+02	1.9709e-04	9.1232e+02
1.4195e-04	8.4548e+02	1.4435e-04	9.3616e+02	3.9306e-03	5.4323e+02	1.6245e-04	9.3836e+02
1.1700e-04	8.9967e+02	1.1898e-04	8.6380e+02	3.3753e-03	6.5593e+02	1.3390e-04	8.9778e+02
9.6440e-05	8.8527e+02	9.8072e-05	1.0023e+03	2.8199e-03	6.0384e+02	1.1037e-04	1.0100e+03
7.9492e-05	9.5728e+02	8.0836e-05	9.2163e+02	2.2646e-03	7.0604e+02	9.0973e-05	1.0126e+03
6.5521e-05	9.6940e+02	6.6630e-05	9.6895e+02	1.7092e-03	7.0124e+02	7.4985e-05	9.7901e+02
5.4007e-05	1.0223e+03	5.4920e-05	9.5130e+02	1.1539e-03	8.0509e+02	6.1807e-05	1.0116e+03
4.4515e-05	8.9473e+02	4.5268e-05	8.9150e+02	5.9850e-04	8.7238e+02	5.0945e-05	1.0082e+03
3.6692e-05	1.0699e+03	3.7313e-05	9.6849e+02	4.3148e-05	9.6841e+02	4.1992e-05	1.0470e+03

FREQUENCY [Hz]: 570430		600980		676230		680170	
TAU [s]	MAGNITUDES [a.u]	TAU [s]	MAGNITUDES [a.u]	TAU [s]	MAGNITUDES [a.u]	TAU [s]	MAGNITUDES [a.u]
1.9481e-02	3.6066e+02	1.6402e-02	3.2009e+02	1.6374e-02	3.4922e+02	2.2855e-02	2.5438e+02
1.8854e-02	2.6169e+02	1.3519e-02	3.8212e+02	1.3496e-02	3.8601e+02	2.2120e-02	2.6967e+02
1.8227e-02	2.7445e+02	1.1143e-02	3.9059e+02	1.1124e-02	3.9987e+02	2.1384e-02	2.4535e+02
1.7601e-02	2.6903e+02	9.1851e-03	3.8598e+02	9.1694e-03	3.5838e+02	2.0649e-02	2.6130e+02
1.6974e-02	2.8946e+02	7.5709e-03	4.8013e+02	7.5579e-03	3.5155e+02	1.9913e-02	2.8392e+02
1.6347e-02	2.9854e+02	6.2403e-03	4.1997e+02	6.2297e-03	5.1053e+02	1.9178e-02	2.9411e+02
1.5720e-02	2.7167e+02	5.1436e-03	5.5897e+02	5.1348e-03	5.2385e+02	1.8442e-02	2.6301e+02
1.5093e-02	2.9408e+02	4.2397e-03	5.6855e+02	4.2324e-03	5.1314e+02	1.7707e-02	2.9111e+02
1.4466e-02	2.9616e+02	3.4946e-03	5.8895e+02	3.4886e-03	5.6182e+02	1.6972e-02	3.3234e+02
1.3839e-02	2.6904e+02	2.8804e-03	6.7311e+02	2.8755e-03	6.1663e+02	1.6236e-02	3.0135e+02
1.3213e-02	3.2348e+02	2.3742e-03	7.4493e+02	2.3702e-03	7.0393e+02	1.5501e-02	3.0208e+02
1.2586e-02	3.2815e+02	1.9570e-03	7.4888e+02	1.9536e-03	7.4945e+02	1.4765e-02	2.7498e+02
1.1959e-02	3.0361e+02	1.6130e-03	7.1921e+02	1.6103e-03	8.5338e+02	1.4030e-02	2.7462e+02
1.1332e-02	3.7352e+02	1.3296e-03	7.8168e+02	1.3273e-03	8.6424e+02	1.3295e-02	2.9084e+02
1.0705e-02	3.4753e+02	1.0959e-03	8.2774e+02	1.0940e-03	8.5208e+02	1.2559e-02	2.8378e+02
1.0078e-02	3.6357e+02	9.0330e-04	7.7530e+02	9.0176e-04	8.6304e+02	1.1824e-02	2.9463e+02
9.4515e-03	3.6178e+02	7.4455e-04	8.7398e+02	7.4328e-04	8.8039e+02	1.1088e-02	3.2537e+02
8.8246e-03	3.9746e+02	6.1370e-04	8.7055e+02	6.1266e-04	9.5546e+02	1.0353e-02	3.0399e+02
8.1978e-03	3.6845e+02	5.0585e-04	8.4985e+02	5.0499e-04	8.5217e+02	9.6175e-03	3.6013e+02
7.5709e-03	4.0206e+02	4.1695e-04	9.8815e+02	4.1624e-04	9.4538e+02	8.8821e-03	4.0278e+02
6.9441e-03	4.3109e+02	3.4367e-04	8.6253e+02	3.4309e-04	9.0285e+02	8.1467e-03	3.6489e+02
6.3172e-03	4.1591e+02	2.8328e-04	9.2475e+02	2.8279e-04	8.9668e+02	7.4113e-03	3.9242e+02
5.6904e-03	4.6177e+02	2.3349e-04	9.7019e+02	2.3309e-04	1.0252e+03	6.6758e-03	4.6562e+02
5.0635e-03	4.9027e+02	1.9246e-04	1.0515e+03	1.9213e-04	9.7863e+02	5.9404e-03	5.4322e+02
4.4367e-03	5.4718e+02	1.5863e-04	9.0932e+02	1.5836e-04	9.5423e+02	5.2050e-03	4.9037e+02
3.8098e-03	5.6409e+02	1.3076e-04	9.0604e+02	1.3053e-04	9.0565e+02	4.4696e-03	5.9703e+02
3.1830e-03	6.4208e+02	1.0778e-04	9.6075e+02	1.0759e-04	9.3584e+02	3.7342e-03	6.1941e+02
2.5561e-03	6.8074e+02	8.8835e-05	9.5171e+02	8.8683e-05	1.0429e+03	2.9988e-03	6.3760e+02
1.9293e-03	7.0875e+02	7.3223e-05	1.0277e+03	7.3098e-05	1.0013e+03	2.2634e-03	7.5431e+02
1.3024e-03	7.7236e+02	6.0355e-05	1.0026e+03	6.0251e-05	9.5892e+02	1.5280e-03	7.9968e+02
6.7555e-04	9.1135e+02	4.9748e-05	9.3012e+02	4.9663e-05	9.1404e+02	7.9255e-04	8.8755e+02
4.8703e-05	9.8822e+02	4.1005e-05	1.0027e+03	4.0935e-05	8.9865e+02	5.7137e-05	9.5829e+02

FREQUENCY [Hz]: 760090		812470		853270		959640	
TAU [s]	MAGNITUDES [a.u]	TAU [s]	MAGNITUDES [a.u]	TAU [s]	MAGNITUDES [a.u]	TAU [s]	MAGNITUDES [a.u]
2.4162e-02	3.1793e+02	2.2999e-02	3.0082e+02	2.3893e-02	3.1246e+02	2.5632e-02	3.2604e+02
1.9916e-02	3.6939e+02	2.2259e-02	2.6636e+02	1.9694e-02	3.3525e+02	2.1127e-02	3.5048e+02
1.6416e-02	3.7591e+02	2.1519e-02	3.0807e+02	1.6233e-02	4.5982e+02	1.7414e-02	4.6556e+02
1.3531e-02	4.1430e+02	2.0779e-02	2.9891e+02	1.3380e-02	3.9398e+02	1.4354e-02	4.1482e+02
1.1153e-02	4.3735e+02	2.0039e-02	3.2335e+02	1.1029e-02	4.5779e+02	1.1831e-02	5.1819e+02
9.1927e-03	4.5446e+02	1.9299e-02	3.0306e+02	9.0904e-03	4.5198e+02	9.7521e-03	5.4313e+02
7.5771e-03	5.1350e+02	1.8559e-02	2.9293e+02	7.4928e-03	5.0705e+02	8.0382e-03	5.2628e+02
6.2455e-03	5.0794e+02	1.7819e-02	3.1570e+02	6.1760e-03	5.9031e+02	6.6255e-03	6.0131e+02
5.1479e-03	6.0708e+02	1.7079e-02	2.6512e+02	5.0906e-03	7.6809e+02	5.4611e-03	7.1178e+02
4.2432e-03	6.4954e+02	1.6338e-02	3.4732e+02	4.1960e-03	6.1753e+02	4.5014e-03	6.7970e+02
3.4975e-03	6.7914e+02	1.5598e-02	3.4520e+02	3.4586e-03	7.3683e+02	3.7103e-03	7.9667e+02
2.8828e-03	6.8453e+02	1.4858e-02	3.3491e+02	2.8507e-03	8.2086e+02	3.0582e-03	8.0977e+02
2.3762e-03	7.8517e+02	1.4118e-02	3.6419e+02	2.3497e-03	8.1211e+02	2.5208e-03	8.1871e+02
1.9586e-03	7.8276e+02	1.3378e-02	3.2427e+02	1.9368e-03	8.5519e+02	2.0778e-03	8.9925e+02
1.6144e-03	8.0455e+02	1.2638e-02	3.3292e+02	1.5964e-03	8.7685e+02	1.7126e-03	8.1424e+02
1.3307e-03	8.6417e+02	1.1898e-02	3.8157e+02	1.3159e-03	8.9606e+02	1.4116e-03	9.7657e+02
1.0968e-03	9.5610e+02	1.1158e-02	4.0354e+02	1.0846e-03	9.6242e+02	1.1635e-03	1.0730e+03
9.0405e-04	9.1160e+02	1.0418e-02	3.3441e+02	8.9399e-04	1.0018e+03	9.5906e-04	8.8614e+02
7.4517e-04	8.2662e+02	9.6781e-03	3.8769e+02	7.3688e-04	9.8092e+02	7.9051e-04	9.7541e+02
6.1421e-04	9.4681e+02	8.9380e-03	4.4712e+02	6.0738e-04	1.0068e+03	6.5159e-04	1.0709e+03
5.0627e-04	9.7593e+02	8.1980e-03	4.3949e+02	5.0063e-04	9.6274e+02	5.3707e-04	9.4376e+02
4.1730e-04	9.1477e+02	7.4579e-03	4.2973e+02	4.1265e-04	1.0162e+03	4.4269e-04	1.0334e+03
3.4396e-04	1.0286e+03	6.7179e-03	4.9244e+02	3.4013e-04	1.0086e+03	3.6489e-04	1.0734e+03
2.8351e-04	9.3204e+02	5.9779e-03	5.2045e+02	2.8036e-04	1.0002e+03	3.0076e-04	9.6546e+02
2.3369e-04	9.9527e+02	5.2378e-03	5.9818e+02	2.3108e-04	1.0686e+03	2.4790e-04	1.0296e+03
1.9262e-04	9.8467e+02	4.4978e-03	6.3947e+02	1.9047e-04	1.0151e+03	2.0434e-04	1.0477e+03
1.5877e-04	9.4125e+02	3.7577e-03	6.2719e+02	1.5700e-04	1.0788e+03	1.6843e-04	1.0394e+03
1.3086e-04	1.0054e+03	3.0177e-03	7.8851e+02	1.2941e-04	1.0207e+03	1.3883e-04	9.8726e+02
1.0787e-04	9.6116e+02	2.2776e-03	7.6742e+02	1.0666e-04	9.8276e+02	1.1443e-04	1.0312e+03
8.8909e-05	9.9437e+02	1.5376e-03	8.6933e+02	8.7919e-05	1.0640e+03	9.4319e-05	1.0121e+03
7.3284e-05	1.0045e+03	7.9754e-04	1.0013e+03	7.2468e-05	1.1286e+03	7.7743e-05	1.0412e+03
6.0404e-05	1.0517e+03	5.7497e-05	1.0387e+03	5.9732e-05	1.0327e+03	6.4080e-05	1.0517e+03

FREQUENCY [Hz]: 1.0007E6		1.0427E6		1.0772E6		1.0872E6	
TAU [s]	MAGNITUDES [a.u]	TAU [s]	MAGNITUDES [a.u]	TAU [s]	MAGNITUDES [a.u]	TAU [s]	MAGNITUDES [a.u]
3.0618e-02	3.1003e+02	3.1164e-02	3.1620e+02	2.8987e-02	4.2215e+02	3.0477e-02	3.3027e+02
2.9633e-02	3.6277e+02	3.0162e-02	3.2028e+02	2.3893e-02	3.7282e+02	2.9497e-02	3.8298e+02
2.8648e-02	3.0859e+02	2.9159e-02	3.4179e+02	1.9694e-02	3.6234e+02	2.8516e-02	2.8912e+02
2.7663e-02	3.7368e+02	2.8156e-02	3.1205e+02	1.6233e-02	4.7649e+02	2.7535e-02	3.9913e+02
2.6677e-02	3.2506e+02	2.7153e-02	3.3933e+02	1.3380e-02	4.4265e+02	2.6555e-02	3.4745e+02
2.5692e-02	3.5000e+02	2.6150e-02	3.2872e+02	1.1028e-02	5.0023e+02	2.5574e-02	3.5870e+02
2.4707e-02	3.8234e+02	2.5148e-02	3.2168e+02	9.0903e-03	5.2829e+02	2.4593e-02	2.9786e+02
2.3722e-02	4.0547e+02	2.4145e-02	3.1415e+02	7.4927e-03	5.3496e+02	2.3613e-02	3.2509e+02
2.2737e-02	3.7904e+02	2.3142e-02	3.8037e+02	6.1759e-03	7.5397e+02	2.2632e-02	4.0889e+02
2.1751e-02	3.8041e+02	2.2139e-02	3.4266e+02	5.0905e-03	6.6476e+02	2.1651e-02	3.9562e+02
2.0766e-02	3.4793e+02	2.1137e-02	3.5348e+02	4.1959e-03	7.1752e+02	2.0671e-02	4.3071e+02
1.9781e-02	4.0740e+02	2.0134e-02	4.2571e+02	3.4585e-03	8.2508e+02	1.9690e-02	4.6926e+02
1.8796e-02	4.0697e+02	1.9131e-02	3.7199e+02	2.8507e-03	8.2524e+02	1.8709e-02	3.8522e+02
1.7810e-02	4.0244e+02	1.8128e-02	3.6505e+02	2.3497e-03	7.9934e+02	1.7728e-02	3.8663e+02
1.6825e-02	3.7945e+02	1.7125e-02	3.3323e+02	1.9368e-03	9.1969e+02	1.6748e-02	3.8984e+02
1.5840e-02	3.7422e+02	1.6123e-02	4.1300e+02	1.5964e-03	9.2040e+02	1.5767e-02	3.9435e+02
1.4855e-02	4.3188e+02	1.5120e-02	3.6469e+02	1.3158e-03	9.8607e+02	1.4786e-02	4.3018e+02
1.3870e-02	3.9293e+02	1.4117e-02	4.2834e+02	1.0846e-03	1.0377e+03	1.3806e-02	4.3064e+02
1.2884e-02	4.4301e+02	1.3114e-02	4.5670e+02	8.9398e-04	9.3897e+02	1.2825e-02	4.7541e+02
1.1899e-02	5.3672e+02	1.2111e-02	4.4843e+02	7.3687e-04	1.0397e+03	1.1844e-02	4.5003e+02
1.0914e-02	5.4233e+02	1.1109e-02	4.9226e+02	6.0737e-04	9.3915e+02	1.0864e-02	4.9157e+02
9.9287e-03	6.0537e+02	1.0106e-02	5.6566e+02	5.0063e-04	9.1873e+02	9.8830e-03	5.2310e+02
8.9435e-03	5.0520e+02	9.1030e-03	5.5768e+02	4.1265e-04	1.0756e+03	8.9023e-03	6.0842e+02
7.9583e-03	5.1633e+02	8.1002e-03	5.4244e+02	3.4013e-04	1.0548e+03	7.9216e-03	6.0594e+02
6.9731e-03	5.8804e+02	7.0974e-03	6.8683e+02	2.8035e-04	1.1074e+03	6.9410e-03	5.8228e+02
5.9878e-03	6.8730e+02	6.0947e-03	6.3529e+02	2.3108e-04	1.0572e+03	5.9603e-03	6.8716e+02
5.0026e-03	6.2945e+02	5.0919e-03	7.0484e+02	1.9047e-04	1.0768e+03	4.9796e-03	7.5867e+02
4.0174e-03	7.1034e+02	4.0891e-03	6.8482e+02	1.5700e-04	1.0187e+03	3.9989e-03	7.2005e+02
3.0322e-03	8.3296e+02	3.0863e-03	8.1591e+02	1.2941e-04	1.0371e+03	3.0182e-03	8.9739e+02
2.0470e-03	8.6436e+02	2.0835e-03	8.4787e+02	1.0666e-04	1.0055e+03	2.0376e-03	9.5175e+02
1.0618e-03	1.0274e+03	1.0807e-03	9.9632e+02	8.7918e-05	1.1017e+03	1.0569e-03	9.3380e+02
7.6546e-05	1.0896e+03	7.7911e-05	1.0614e+03	7.2467e-05	1.0617e+03	7.6193e-05	1.0258e+03

FREQUENCY [Hz]: 1.1343E6		1.1582E6		1.1824E6		1.2104E6	
TAU [s]	MAGNITUDES [a.u]	TAU [s]	MAGNITUDES [a.u]	TAU [s]	MAGNITUDES [a.u]	TAU [s]	MAGNITUDES [a.u]
2.9462e-02	3.6584e+02	4.5119e-02	2.8735e+02	2.7589e-02	4.2137e+02	3.8814e-02	3.7489e+02
2.8514e-02	4.5759e+02	4.3667e-02	2.9780e+02	2.6701e-02	3.2540e+02	3.1993e-02	3.2420e+02
2.7566e-02	3.3697e+02	4.2215e-02	2.5014e+02	2.5814e-02	4.5958e+02	2.6370e-02	3.4084e+02
2.6618e-02	3.7883e+02	4.0764e-02	2.9630e+02	2.4926e-02	3.4161e+02	2.1736e-02	3.2649e+02
2.5670e-02	4.0007e+02	3.9312e-02	2.8388e+02	2.4038e-02	3.9252e+02	1.7916e-02	4.5445e+02
2.4722e-02	4.0428e+02	3.7860e-02	2.6533e+02	2.3150e-02	3.9529e+02	1.4767e-02	5.4740e+02
2.3774e-02	3.0002e+02	3.6408e-02	2.8147e+02	2.2263e-02	4.2653e+02	1.2172e-02	5.2825e+02
2.2826e-02	4.0966e+02	3.4956e-02	3.1373e+02	2.1375e-02	3.6765e+02	1.0033e-02	5.5976e+02
2.1878e-02	3.8746e+02	3.3504e-02	2.9720e+02	2.0487e-02	4.7009e+02	8.2697e-03	6.1908e+02
2.0930e-02	4.1291e+02	3.2053e-02	2.9399e+02	1.9599e-02	3.9534e+02	6.8164e-03	6.6423e+02
1.9982e-02	4.3247e+02	3.0601e-02	2.8891e+02	1.8712e-02	4.6063e+02	5.6184e-03	7.2883e+02
1.9034e-02	4.5447e+02	2.9149e-02	2.8441e+02	1.7824e-02	4.4206e+02	4.6310e-03	8.4704e+02
1.8086e-02	3.7618e+02	2.7697e-02	3.1513e+02	1.6936e-02	4.4134e+02	3.8172e-03	8.1818e+02
1.7138e-02	4.6915e+02	2.6245e-02	3.0704e+02	1.6048e-02	4.0060e+02	3.1463e-03	8.9290e+02
1.6190e-02	4.1504e+02	2.4794e-02	3.2515e+02	1.5161e-02	4.3660e+02	2.5934e-03	8.7584e+02
1.5242e-02	4.3499e+02	2.3342e-02	3.1813e+02	1.4273e-02	4.7607e+02	2.1376e-03	9.0098e+02
1.4294e-02	4.7735e+02	2.1890e-02	3.5602e+02	1.3385e-02	4.5342e+02	1.7619e-03	9.5779e+02
1.3346e-02	4.1517e+02	2.0438e-02	3.4223e+02	1.2497e-02	5.2961e+02	1.4523e-03	9.4040e+02
1.2398e-02	4.7846e+02	1.8986e-02	3.6515e+02	1.1610e-02	5.0138e+02	1.1971e-03	1.0055e+03
1.1450e-02	5.2751e+02	1.7535e-02	4.1555e+02	1.0722e-02	5.5621e+02	9.8669e-04	1.0146e+03
1.0502e-02	5.7795e+02	1.6083e-02	4.1412e+02	9.8341e-03	5.4525e+02	8.1328e-04	1.0583e+03
9.5538e-03	5.5987e+02	1.4631e-02	4.5946e+02	8.9464e-03	6.0075e+02	6.7035e-04	1.0795e+03
8.6058e-03	5.9406e+02	1.3179e-02	4.8949e+02	8.0587e-03	5.7019e+02	5.5254e-04	1.0654e+03
7.6578e-03	6.2071e+02	1.1727e-02	4.9227e+02	7.1709e-03	7.1828e+02	4.5544e-04	1.0398e+03
6.7098e-03	6.5312e+02	1.0275e-02	5.3544e+02	6.2832e-03	7.4900e+02	3.7540e-04	1.0314e+03
5.7617e-03	7.3396e+02	8.8237e-03	5.6770e+02	5.3954e-03	7.5602e+02	3.0942e-04	1.0771e+03
4.8137e-03	7.3894e+02	7.3719e-03	6.6001e+02	4.5077e-03	8.4332e+02	2.5505e-04	1.1351e+03
3.8657e-03	8.2961e+02	5.9200e-03	6.9401e+02	3.6199e-03	8.3810e+02	2.1022e-04	1.1058e+03
2.9177e-03	8.6560e+02	4.4682e-03	7.2488e+02	2.7322e-03	9.1469e+02	1.7328e-04	1.1300e+03
1.9697e-03	9.9691e+02	3.0164e-03	8.5106e+02	1.8445e-03	9.7471e+02	1.4283e-04	9.8276e+02
1.0217e-03	9.9600e+02	1.5646e-03	9.4959e+02	9.5672e-04	1.0084e+03	1.1772e-04	1.0330e+03
7.3655e-05	1.0458e+03	1.1280e-04	1.1211e+03	6.8973e-05	1.1429e+03	9.7035e-05	9.8881e+02

FREQUENCY [Hz]: 1.2333E6		1.2853E6		1.3402E6		1.3606E6	
TAU [s]	MAGNITUDES [a.u]	TAU [s]	MAGNITUDES [a.u]	TAU [s]	MAGNITUDES [a.u]	TAU [s]	MAGNITUDES [a.u]
3.2365e-02	3.8430e+02	3.6110e-02	3.0099e+02	4.1760e-02	3.3116e+02	3.2319e-02	3.7539e+02
3.1323e-02	3.6267e+02	3.4948e-02	3.3977e+02	4.0417e-02	3.9137e+02	2.6639e-02	4.1735e+02
3.0282e-02	4.1526e+02	3.3786e-02	3.4593e+02	3.9073e-02	3.6874e+02	2.1957e-02	3.7793e+02
2.9241e-02	3.9229e+02	3.2624e-02	3.6786e+02	3.7729e-02	3.4267e+02	1.8099e-02	5.2831e+02
2.8199e-02	4.1687e+02	3.1462e-02	3.5856e+02	3.6386e-02	3.4981e+02	1.4918e-02	5.8694e+02
2.7158e-02	3.8874e+02	3.0300e-02	4.2729e+02	3.5042e-02	4.1554e+02	1.2296e-02	4.7872e+02
2.6116e-02	4.1500e+02	2.9138e-02	3.6645e+02	3.3698e-02	3.6411e+02	1.0135e-02	6.4038e+02
2.5075e-02	3.7756e+02	2.7977e-02	3.4767e+02	3.2354e-02	3.9747e+02	8.3540e-03	6.2190e+02
2.4033e-02	3.9326e+02	2.6815e-02	4.1914e+02	3.1011e-02	3.8663e+02	6.8859e-03	7.8393e+02
2.2992e-02	4.3934e+02	2.5653e-02	4.2186e+02	2.9667e-02	4.2335e+02	5.6757e-03	7.8691e+02
2.1951e-02	3.9383e+02	2.4491e-02	4.2825e+02	2.8323e-02	3.6504e+02	4.6782e-03	7.7735e+02
2.0909e-02	4.7812e+02	2.3329e-02	4.0591e+02	2.6979e-02	3.7682e+02	3.8561e-03	8.3466e+02
1.9868e-02	4.4661e+02	2.2167e-02	3.1531e+02	2.5636e-02	4.3917e+02	3.1784e-03	8.5809e+02
1.8826e-02	4.6678e+02	2.1005e-02	3.1200e+02	2.4292e-02	4.8215e+02	2.6198e-03	8.5494e+02
1.7785e-02	4.4995e+02	1.9843e-02	3.1667e+02	2.2948e-02	4.0687e+02	2.1594e-03	8.9910e+02
1.6744e-02	4.8659e+02	1.8681e-02	4.5858e+02	2.1604e-02	4.2464e+02	1.7799e-03	1.0215e+03
1.5702e-02	4.7990e+02	1.7519e-02	4.1618e+02	2.0261e-02	4.1188e+02	1.4671e-03	9.9393e+02
1.4661e-02	5.2106e+02	1.6357e-02	4.9744e+02	1.8917e-02	4.5662e+02	1.2093e-03	1.0102e+03
1.3619e-02	5.2958e+02	1.5195e-02	4.3248e+02	1.7573e-02	4.6283e+02	9.9674e-04	1.0216e+03
1.2578e-02	6.0835e+02	1.4033e-02	5.4374e+02	1.6229e-02	5.0438e+02	8.2157e-04	1.0803e+03
1.1536e-02	5.8772e+02	1.2871e-02	5.3752e+02	1.4886e-02	4.9011e+02	6.7719e-04	9.7160e+02
1.0495e-02	6.6261e+02	1.1710e-02	5.9662e+02	1.3542e-02	5.2077e+02	5.5818e-04	1.0956e+03
9.4536e-03	6.0037e+02	1.0548e-02	5.4826e+02	1.2198e-02	6.0996e+02	4.6008e-04	1.0751e+03
8.4122e-03	5.7862e+02	9.3857e-03	6.5473e+02	1.0854e-02	5.2138e+02	3.7922e-04	1.0636e+03
7.3708e-03	6.8613e+02	8.2238e-03	6.8752e+02	9.5106e-03	6.2719e+02	3.1258e-04	1.0707e+03
6.3294e-03	7.0353e+02	7.0618e-03	6.6438e+02	8.1669e-03	6.8078e+02	2.5764e-04	1.0243e+03
5.2880e-03	8.2089e+02	5.8999e-03	7.4927e+02	6.8231e-03	6.7576e+02	2.1237e-04	1.0872e+03
4.2466e-03	9.2408e+02	4.7380e-03	8.4321e+02	5.4794e-03	7.9385e+02	1.7504e-04	1.0701e+03
3.2052e-03	8.8784e+02	3.5761e-03	8.9881e+02	4.1356e-03	8.8416e+02	1.4428e-04	1.0376e+03
2.1637e-03	9.7709e+02	2.4141e-03	8.7500e+02	2.7919e-03	8.2101e+02	1.1892e-04	1.0834e+03
1.1223e-03	9.9319e+02	1.2522e-03	1.0600e+03	1.4481e-03	9.6995e+02	9.8024e-05	1.0515e+03
8.0912e-05	1.1774e+03	9.0275e-05	1.0396e+03	1.0440e-04	1.1020e+03	8.0797e-05	1.0543e+03

FREQUENCY [Hz]: 1.3811E6		1.3981E6		1.5195E6		1.5852E6	
TAU [s]	MAGNITUDES [a.u]	TAU [s]	MAGNITUDES [a.u]	TAU [s]	MAGNITUDES [a.u]	TAU [s]	MAGNITUDES [a.u]
4.0085e-02	3.5106e+02	3.5036e-02	3.9932e+02	4.4457e-02	3.5947e+02	3.6612e-02	3.9920e+02
3.8795e-02	3.2902e+02	3.3908e-02	3.4544e+02	4.3027e-02	3.5403e+02	3.5434e-02	3.7292e+02
3.7505e-02	3.3274e+02	3.2781e-02	3.7501e+02	4.1596e-02	3.2313e+02	3.4256e-02	3.6930e+02
3.6216e-02	2.7657e+02	3.1654e-02	3.8884e+02	4.0166e-02	3.8078e+02	3.3077e-02	3.6245e+02
3.4926e-02	2.9471e+02	3.0526e-02	3.7957e+02	3.8735e-02	3.6654e+02	3.1899e-02	3.5795e+02
3.3636e-02	3.1146e+02	2.9399e-02	3.9040e+02	3.7305e-02	3.5926e+02	3.0721e-02	3.7638e+02
3.2346e-02	3.1682e+02	2.8271e-02	3.4954e+02	3.5874e-02	4.4202e+02	2.9543e-02	4.3930e+02
3.1056e-02	3.4150e+02	2.7144e-02	3.5146e+02	3.4444e-02	2.6283e+02	2.8365e-02	4.6942e+02
2.9766e-02	2.9189e+02	2.6017e-02	3.7655e+02	3.3013e-02	3.6638e+02	2.7187e-02	3.9201e+02
2.8477e-02	3.7188e+02	2.4889e-02	4.3753e+02	3.1583e-02	4.2265e+02	2.6009e-02	4.0353e+02
2.7187e-02	3.7402e+02	2.3762e-02	4.4349e+02	3.0152e-02	3.8698e+02	2.4831e-02	4.1062e+02
2.5897e-02	2.5927e+02	2.2635e-02	3.7105e+02	2.8722e-02	3.7611e+02	2.3653e-02	4.3469e+02
2.4607e-02	3.4086e+02	2.1507e-02	4.3015e+02	2.7291e-02	3.8228e+02	2.2475e-02	4.7637e+02
2.3317e-02	3.5560e+02	2.0380e-02	4.7799e+02	2.5861e-02	4.2062e+02	2.1297e-02	4.5702e+02
2.2027e-02	3.4633e+02	1.9253e-02	4.6982e+02	2.4430e-02	4.1929e+02	2.0119e-02	4.6881e+02
2.0738e-02	3.7526e+02	1.8125e-02	4.9320e+02	2.2999e-02	4.4147e+02	1.8941e-02	4.5894e+02
1.9448e-02	3.9399e+02	1.6998e-02	4.9344e+02	2.1569e-02	5.6431e+02	1.7763e-02	5.1468e+02
1.8158e-02	3.7839e+02	1.5871e-02	5.0322e+02	2.0138e-02	4.5712e+02	1.6584e-02	5.2817e+02
1.6868e-02	4.2063e+02	1.4743e-02	4.8524e+02	1.8708e-02	4.5435e+02	1.5406e-02	4.9519e+02
1.5578e-02	4.2153e+02	1.3616e-02	5.1338e+02	1.7277e-02	4.9705e+02	1.4228e-02	5.7830e+02
1.4288e-02	4.9596e+02	1.2488e-02	5.3284e+02	1.5847e-02	4.8055e+02	1.3050e-02	5.0877e+02
1.2999e-02	5.2834e+02	1.1361e-02	6.2323e+02	1.4416e-02	5.5539e+02	1.1872e-02	6.5561e+02
1.1709e-02	5.6696e+02	1.0234e-02	6.2865e+02	1.2986e-02	5.4058e+02	1.0694e-02	6.2965e+02
1.0419e-02	5.6584e+02	9.1064e-03	6.8213e+02	1.1555e-02	5.4779e+02	9.5161e-03	7.0574e+02
9.1290e-03	6.4295e+02	7.9791e-03	6.3527e+02	1.0125e-02	6.7940e+02	8.3380e-03	7.2021e+02
7.8392e-03	6.8947e+02	6.8517e-03	7.8553e+02	8.6943e-03	7.3347e+02	7.1599e-03	7.9236e+02
6.5494e-03	7.8544e+02	5.7244e-03	7.9606e+02	7.2638e-03	6.4523e+02	5.9819e-03	8.1800e+02
5.2595e-03	7.8176e+02	4.5970e-03	9.2658e+02	5.8332e-03	7.8273e+02	4.8038e-03	9.3290e+02
3.9697e-03	8.7326e+02	3.4697e-03	8.7134e+02	4.4027e-03	7.9783e+02	3.6257e-03	1.0119e+03
2.6799e-03	9.1897e+02	2.3423e-03	9.5624e+02	2.9722e-03	9.0703e+02	2.4477e-03	1.0573e+03
1.3900e-03	1.0559e+03	1.2149e-03	1.0070e+03	1.5417e-03	9.8640e+02	1.2696e-03	1.0681e+03
1.0021e-04	1.1507e+03	8.7589e-05	1.0978e+03	1.1114e-04	1.1246e+03	9.1529e-05	1.1150e+03

FREQUENCY [Hz]: 1.6502E6		1.7248E6		1.7969E6		1.9298E6	
TAU [s]	MAGNITUDES [a.u]	TAU [s]	MAGNITUDES [a.u]	TAU [s]	MAGNITUDES [a.u]	TAU [s]	MAGNITUDES [a.u]
5.1837e-02	3.0185e+02	4.5173e-02	3.5760e+02	3.6385e-02	3.6774e+02	3.8393e-02	3.8208e+02
5.0169e-02	2.8144e+02	4.3719e-02	4.0580e+02	3.5214e-02	3.7751e+02	3.1646e-02	3.2186e+02
4.8501e-02	3.0436e+02	4.2266e-02	3.5770e+02	3.4044e-02	3.4273e+02	2.6084e-02	3.4816e+02
4.6833e-02	2.8480e+02	4.0812e-02	3.6232e+02	3.2873e-02	3.4687e+02	2.1500e-02	4.2279e+02
4.5165e-02	2.6744e+02	3.9359e-02	3.8408e+02	3.1702e-02	4.1242e+02	1.7722e-02	5.0392e+02
4.3497e-02	3.0156e+02	3.7905e-02	4.1728e+02	3.0531e-02	4.2154e+02	1.4607e-02	5.1509e+02
4.1829e-02	2.7385e+02	3.6452e-02	3.6108e+02	2.9360e-02	4.1865e+02	1.2040e-02	5.7741e+02
4.0161e-02	2.4453e+02	3.4998e-02	3.8274e+02	2.8190e-02	3.7450e+02	9.9242e-03	6.4628e+02
3.8493e-02	3.0406e+02	3.3544e-02	3.8030e+02	2.7019e-02	3.7918e+02	8.1801e-03	6.1469e+02
3.6825e-02	3.1909e+02	3.2091e-02	3.8916e+02	2.5848e-02	4.5319e+02	6.7425e-03	7.1738e+02
3.5157e-02	3.6889e+02	3.0637e-02	4.6566e+02	2.4677e-02	3.9394e+02	5.5575e-03	7.8650e+02
3.3489e-02	3.1259e+02	2.9184e-02	4.0826e+02	2.3507e-02	4.7150e+02	4.5808e-03	8.6602e+02
3.1821e-02	3.1807e+02	2.7730e-02	3.8248e+02	2.2336e-02	4.6706e+02	3.7758e-03	8.3153e+02
3.0153e-02	3.6741e+02	2.6277e-02	4.4877e+02	2.1165e-02	4.6705e+02	3.1122e-03	8.8234e+02
2.8485e-02	3.8019e+02	2.4823e-02	4.4299e+02	1.9994e-02	4.3131e+02	2.5653e-03	9.1688e+02
2.6817e-02	3.4408e+02	2.3370e-02	5.1454e+02	1.8823e-02	4.8552e+02	2.1144e-03	1.0559e+03
2.5149e-02	3.4123e+02	2.1916e-02	4.6430e+02	1.7653e-02	4.9082e+02	1.7428e-03	9.1570e+02
2.3481e-02	3.7907e+02	2.0463e-02	4.8407e+02	1.6482e-02	5.2131e+02	1.4365e-03	1.0010e+03
2.1813e-02	4.1878e+02	1.9009e-02	4.6920e+02	1.5311e-02	5.5068e+02	1.1841e-03	9.9147e+02
2.0145e-02	4.5315e+02	1.7555e-02	4.9170e+02	1.4140e-02	5.5663e+02	9.7599e-04	1.0142e+03
1.8477e-02	4.0266e+02	1.6102e-02	5.2182e+02	1.2970e-02	6.2015e+02	8.0447e-04	1.0066e+03
1.6809e-02	4.4997e+02	1.4648e-02	5.7584e+02	1.1799e-02	6.9350e+02	6.6309e-04	1.1065e+03
1.5141e-02	5.1421e+02	1.3195e-02	5.5317e+02	1.0628e-02	6.7371e+02	5.4655e-04	9.6902e+02
1.3473e-02	4.8212e+02	1.1741e-02	5.5136e+02	9.4572e-03	6.8328e+02	4.5050e-04	1.1268e+03
1.1806e-02	5.5917e+02	1.0288e-02	6.3770e+02	8.2864e-03	6.9941e+02	3.7133e-04	1.0644e+03
1.0138e-02	6.0650e+02	8.8342e-03	7.4316e+02	7.1156e-03	7.6590e+02	3.0607e-04	1.0661e+03
8.4695e-03	6.8560e+02	7.3806e-03	7.4658e+02	5.9449e-03	7.5822e+02	2.5228e-04	1.0227e+03
6.8015e-03	7.5224e+02	5.9271e-03	8.4863e+02	4.7741e-03	7.7410e+02	2.0794e-04	1.0678e+03
5.1336e-03	7.7860e+02	4.4736e-03	8.6029e+02	3.6033e-03	9.4008e+02	1.7140e-04	1.0796e+03
3.4656e-03	8.8749e+02	3.0200e-03	9.0878e+02	2.4325e-03	9.6845e+02	1.4128e-04	1.0324e+03
1.7976e-03	1.0172e+03	1.5665e-03	1.0659e+03	1.2617e-03	1.0801e+03	1.1645e-04	1.0509e+03
1.2959e-04	1.0904e+03	1.1293e-04	1.0578e+03	9.0963e-05	1.1459e+03	9.5983e-05	1.0143e+03

FREQUENCY [Hz]: 1.97E6		2.0378E6		2.1253E6		2.1698E6	
TAU [s]	MAGNITUDES [a.u]	TAU [s]	MAGNITUDES [a.u]	TAU [s]	MAGNITUDES [a.u]	TAU [s]	MAGNITUDES [a.u]
4.5539e-02	3.1757e+02	2.7897e-02	3.6982e+02	2.5755e-02	4.3133e+02	2.8660e-02	3.8844e+02
4.4073e-02	3.3832e+02	2.6999e-02	4.2285e+02	2.4926e-02	4.0098e+02	2.3623e-02	4.1073e+02
4.2608e-02	3.2198e+02	2.6101e-02	4.7176e+02	2.4098e-02	4.1895e+02	1.9471e-02	4.2955e+02
4.1143e-02	2.6836e+02	2.5204e-02	4.3994e+02	2.3269e-02	4.4690e+02	1.6049e-02	3.7999e+02
3.9677e-02	3.1122e+02	2.4306e-02	4.2397e+02	2.2440e-02	4.3113e+02	1.3229e-02	4.4541e+02
3.8212e-02	2.9613e+02	2.3408e-02	4.3117e+02	2.1611e-02	4.5294e+02	1.0904e-02	5.8072e+02
3.6747e-02	3.0629e+02	2.2511e-02	4.0678e+02	2.0783e-02	4.4171e+02	8.9876e-03	5.4934e+02
3.5281e-02	3.4898e+02	2.1613e-02	4.6895e+02	1.9954e-02	4.4465e+02	7.4081e-03	6.1869e+02
3.3816e-02	3.2913e+02	2.0715e-02	4.4070e+02	1.9125e-02	4.5795e+02	6.1062e-03	6.7450e+02
3.2351e-02	3.0268e+02	1.9818e-02	4.4621e+02	1.8296e-02	3.3988e+02	5.0331e-03	7.3406e+02
3.0886e-02	3.4905e+02	1.8920e-02	4.7615e+02	1.7468e-02	3.6018e+02	4.1485e-03	7.3338e+02
2.9420e-02	3.7132e+02	1.8023e-02	4.2756e+02	1.6639e-02	4.7926e+02	3.4195e-03	7.2843e+02
2.7955e-02	3.3186e+02	1.7125e-02	4.8072e+02	1.5810e-02	4.8082e+02	2.8185e-03	8.2271e+02
2.6490e-02	3.8150e+02	1.6227e-02	4.5356e+02	1.4982e-02	4.8371e+02	2.3232e-03	8.7294e+02
2.5024e-02	3.6851e+02	1.5330e-02	4.6844e+02	1.4153e-02	4.9754e+02	1.9149e-03	9.4263e+02
2.3559e-02	3.4957e+02	1.4432e-02	5.0694e+02	1.3324e-02	5.1468e+02	1.5784e-03	9.1215e+02
2.2094e-02	3.7180e+02	1.3534e-02	5.2963e+02	1.2495e-02	4.8002e+02	1.3010e-03	9.3087e+02
2.0628e-02	3.6257e+02	1.2637e-02	5.8316e+02	1.1667e-02	6.0517e+02	1.0723e-03	9.5126e+02
1.9163e-02	4.3035e+02	1.1739e-02	5.0145e+02	1.0838e-02	5.5222e+02	8.8389e-04	9.4207e+02
1.7698e-02	4.1977e+02	1.0841e-02	5.0122e+02	1.0009e-02	4.8970e+02	7.2855e-04	1.0709e+03
1.6232e-02	4.5688e+02	9.9438e-03	6.3390e+02	9.1804e-03	5.7965e+02	6.0051e-04	1.0189e+03
1.4767e-02	4.8606e+02	9.0461e-03	6.8045e+02	8.3517e-03	6.2735e+02	4.9498e-04	1.0287e+03
1.3302e-02	4.7621e+02	8.1485e-03	6.3179e+02	7.5230e-03	6.3821e+02	4.0799e-04	1.0390e+03
1.1836e-02	5.0151e+02	7.2509e-03	6.7527e+02	6.6942e-03	7.5463e+02	3.3629e-04	1.0028e+03
1.0371e-02	5.9114e+02	6.3532e-03	7.6376e+02	5.8655e-03	6.8306e+02	2.7719e-04	9.7574e+02
8.9058e-03	6.7162e+02	5.4556e-03	7.9462e+02	5.0368e-03	7.9661e+02	2.2847e-04	1.0678e+03
7.4404e-03	7.0413e+02	4.5579e-03	8.5791e+02	4.2080e-03	7.4450e+02	1.8832e-04	1.0729e+03
5.9751e-03	7.5099e+02	3.6603e-03	8.4351e+02	3.3793e-03	8.3333e+02	1.5522e-04	1.1090e+03
4.5098e-03	8.0984e+02	2.7627e-03	9.5454e+02	2.5506e-03	8.3768e+02	1.2794e-04	1.0732e+03
3.0445e-03	9.0609e+02	1.8650e-03	9.9177e+02	1.7219e-03	1.0108e+03	1.0546e-04	1.0861e+03
1.5792e-03	1.0245e+03	9.6738e-04	1.0372e+03	8.9312e-04	1.0266e+03	8.6926e-05	1.0504e+03
1.1385e-04	1.1574e+03	6.9741e-05	1.1881e+03	6.4388e-05	1.1104e+03	7.1649e-05	1.0498e+03

FREQUENCY [Hz]: 2.2171E6		2.3108E6		2.3522E6		2.4113E6	
TAU [s]	MAGNITUDES [a.u]	TAU [s]	MAGNITUDES [a.u]	TAU [s]	MAGNITUDES [a.u]	TAU [s]	MAGNITUDES [a.u]
2.4951e-02	3.9202e+02	2.7899e-02	4.4765e+02	3.4961e-02	3.6010e+02	4.1699e-02	4.1465e+02
2.4148e-02	3.7000e+02	2.7001e-02	3.6493e+02	3.3836e-02	3.0040e+02	4.0358e-02	3.9200e+02
2.3346e-02	4.5122e+02	2.6103e-02	4.4567e+02	3.2711e-02	3.4550e+02	3.9016e-02	4.3043e+02
2.2543e-02	4.1560e+02	2.5205e-02	4.3781e+02	3.1586e-02	3.3432e+02	3.7674e-02	3.6762e+02
2.1740e-02	4.1003e+02	2.4308e-02	3.7019e+02	3.0461e-02	3.5130e+02	3.6332e-02	3.9740e+02
2.0937e-02	3.8051e+02	2.3410e-02	4.1278e+02	2.9336e-02	3.7709e+02	3.4990e-02	3.9956e+02
2.0134e-02	4.6099e+02	2.2512e-02	3.9557e+02	2.8211e-02	3.6428e+02	3.3649e-02	3.8964e+02
1.9331e-02	4.9497e+02	2.1615e-02	4.4393e+02	2.7086e-02	3.9744e+02	3.2307e-02	4.9340e+02
1.8528e-02	3.9877e+02	2.0717e-02	3.6277e+02	2.5961e-02	3.4892e+02	3.0965e-02	3.9311e+02
1.7725e-02	4.6372e+02	1.9819e-02	4.2985e+02	2.4836e-02	3.7521e+02	2.9623e-02	4.6505e+02
1.6923e-02	3.6768e+02	1.8922e-02	5.1044e+02	2.3712e-02	3.7579e+02	2.8282e-02	4.5801e+02
1.6120e-02	4.1241e+02	1.8024e-02	4.7635e+02	2.2587e-02	3.6867e+02	2.6940e-02	4.2248e+02
1.5317e-02	4.8336e+02	1.7126e-02	4.9207e+02	2.1462e-02	4.7307e+02	2.5598e-02	4.1704e+02
1.4514e-02	4.2994e+02	1.6228e-02	4.8987e+02	2.0337e-02	4.3530e+02	2.4256e-02	4.2748e+02
1.3711e-02	4.3287e+02	1.5331e-02	4.2274e+02	1.9212e-02	3.6998e+02	2.2914e-02	4.4992e+02
1.2908e-02	4.8756e+02	1.4433e-02	4.5611e+02	1.8087e-02	3.9105e+02	2.1573e-02	5.4124e+02
1.2105e-02	3.8975e+02	1.3535e-02	4.5761e+02	1.6962e-02	3.9615e+02	2.0231e-02	4.9800e+02
1.1303e-02	5.6198e+02	1.2638e-02	4.8555e+02	1.5837e-02	4.3811e+02	1.8889e-02	5.0625e+02
1.0500e-02	5.4531e+02	1.1740e-02	4.5862e+02	1.4712e-02	4.4896e+02	1.7547e-02	5.8614e+02
9.6968e-03	5.6724e+02	1.0842e-02	4.8302e+02	1.3587e-02	4.8018e+02	1.6206e-02	5.4445e+02
8.8939e-03	4.8632e+02	9.9445e-03	5.4797e+02	1.2462e-02	5.1132e+02	1.4864e-02	5.3176e+02
8.0911e-03	6.5577e+02	9.0468e-03	5.3707e+02	1.1337e-02	5.3730e+02	1.3522e-02	5.1115e+02
7.2882e-03	6.0219e+02	8.1491e-03	6.4131e+02	1.0212e-02	5.1844e+02	1.2180e-02	5.7092e+02
6.4853e-03	6.6065e+02	7.2514e-03	7.2491e+02	9.0871e-03	5.8495e+02	1.0838e-02	6.5724e+02
5.6825e-03	7.3301e+02	6.3537e-03	6.4675e+02	7.9621e-03	7.0481e+02	9.4967e-03	6.6993e+02
4.8796e-03	7.8278e+02	5.4560e-03	7.6466e+02	6.8371e-03	7.6655e+02	8.1549e-03	7.1573e+02
4.0767e-03	7.5736e+02	4.5583e-03	7.4707e+02	5.7122e-03	7.2367e+02	6.8131e-03	7.3964e+02
3.2739e-03	8.3514e+02	3.6606e-03	8.3553e+02	4.5872e-03	7.6413e+02	5.4714e-03	8.1164e+02
2.4710e-03	8.6742e+02	2.7629e-03	7.9843e+02	3.4623e-03	8.7209e+02	4.1296e-03	8.5349e+02
1.6681e-03	9.7664e+02	1.8652e-03	9.8560e+02	2.3373e-03	9.7157e+02	2.7878e-03	1.0197e+03
8.6525e-04	1.0211e+03	9.6745e-04	1.0223e+03	1.2124e-03	1.0596e+03	1.4460e-03	1.1175e+03
6.2378e-05	1.1381e+03	6.9746e-05	1.1174e+03	8.7403e-05	1.1272e+03	1.0425e-04	1.1189e+03

FREQUENCY [Hz]: 2.4362E6		2.5141E6		2.6215E6		2.7339E6	
TAU [s]	MAGNITUDES [a.u]	TAU [s]	MAGNITUDES [a.u]	TAU [s]	MAGNITUDES [a.u]	TAU [s]	MAGNITUDES [a.u]
3.5704e-02	4.1694e+02	5.6858e-02	4.2008e+02	5.7930e-02	3.9276e+02	3.8766e-02	4.0189e+02
2.9429e-02	3.9552e+02	5.5029e-02	4.0365e+02	5.6066e-02	3.7155e+02	3.7518e-02	4.4312e+02
2.4257e-02	4.8617e+02	5.3199e-02	4.3785e+02	5.4202e-02	4.3557e+02	3.6271e-02	3.9985e+02
1.9994e-02	4.4086e+02	5.1370e-02	4.0969e+02	5.2338e-02	4.2114e+02	3.5024e-02	4.0433e+02
1.6480e-02	4.8266e+02	4.9540e-02	3.8883e+02	5.0474e-02	4.1367e+02	3.3776e-02	3.5939e+02
1.3584e-02	5.8350e+02	4.7710e-02	3.5593e+02	4.8610e-02	3.7909e+02	3.2529e-02	3.9594e+02
1.1197e-02	6.8235e+02	4.5881e-02	4.5521e+02	4.6746e-02	4.1753e+02	3.1282e-02	4.1778e+02
9.2289e-03	6.3144e+02	4.4051e-02	4.3998e+02	4.4882e-02	3.9259e+02	3.0034e-02	4.0795e+02
7.6070e-03	7.1461e+02	4.2222e-02	3.5018e+02	4.3018e-02	4.0157e+02	2.8787e-02	4.0368e+02
6.2701e-03	7.9725e+02	4.0392e-02	4.3908e+02	4.1154e-02	3.8760e+02	2.7539e-02	4.7891e+02
5.1682e-03	8.0351e+02	3.8563e-02	4.2032e+02	3.9290e-02	4.6657e+02	2.6292e-02	4.2969e+02
4.2599e-03	8.6455e+02	3.6733e-02	4.1817e+02	3.7426e-02	4.1653e+02	2.5045e-02	4.1430e+02
3.5113e-03	9.4398e+02	3.4904e-02	4.3781e+02	3.5562e-02	4.6440e+02	2.3797e-02	4.7269e+02
2.8942e-03	8.6540e+02	3.3074e-02	3.9434e+02	3.3697e-02	4.6327e+02	2.2550e-02	4.7111e+02
2.3856e-03	9.7774e+02	3.1245e-02	4.4334e+02	3.1833e-02	5.3626e+02	2.1302e-02	5.0408e+02
1.9663e-03	9.6711e+02	2.9415e-02	4.7704e+02	2.9969e-02	4.7013e+02	2.0055e-02	4.1779e+02
1.6207e-03	9.9661e+02	2.7585e-02	4.6223e+02	2.8105e-02	5.2255e+02	1.8808e-02	5.5654e+02
1.3359e-03	9.1983e+02	2.5756e-02	4.7550e+02	2.6241e-02	4.1799e+02	1.7560e-02	5.3158e+02
1.1011e-03	1.0787e+03	2.3926e-02	5.0689e+02	2.4377e-02	4.9948e+02	1.6313e-02	5.6224e+02
9.0762e-04	1.0672e+03	2.2097e-02	5.4618e+02	2.2513e-02	5.0040e+02	1.5066e-02	5.8564e+02
7.4811e-04	1.0971e+03	2.0267e-02	5.2888e+02	2.0649e-02	6.0916e+02	1.3818e-02	5.7909e+02
6.1664e-04	1.0551e+03	1.8438e-02	5.9443e+02	1.8785e-02	5.5328e+02	1.2571e-02	5.7350e+02
5.0827e-04	1.0577e+03	1.6608e-02	5.5332e+02	1.6921e-02	6.0342e+02	1.1323e-02	6.5669e+02
4.1894e-04	1.1310e+03	1.4779e-02	6.6386e+02	1.5057e-02	6.8596e+02	1.0076e-02	6.9250e+02
3.4532e-04	1.1337e+03	1.2949e-02	7.5883e+02	1.3193e-02	6.5459e+02	8.8286e-03	6.9975e+02
2.8463e-04	1.0674e+03	1.1119e-02	6.4125e+02	1.1329e-02	7.7657e+02	7.5812e-03	7.0119e+02
2.3461e-04	1.0782e+03	9.2899e-03	7.3606e+02	9.4650e-03	7.4440e+02	6.3338e-03	8.0024e+02
1.9338e-04	9.9884e+02	7.4604e-03	8.3236e+02	7.6010e-03	8.1033e+02	5.0865e-03	8.6967e+02
1.5939e-04	1.1975e+03	5.6308e-03	8.9727e+02	5.7369e-03	9.4944e+02	3.8391e-03	9.3129e+02
1.3138e-04	1.1176e+03	3.8012e-03	9.1958e+02	3.8729e-03	1.0748e+03	2.5917e-03	1.0288e+03
1.0829e-04	1.1294e+03	1.9717e-03	1.0525e+03	2.0089e-03	1.0465e+03	1.3443e-03	1.0596e+03
8.9259e-05	1.1488e+03	1.4215e-04	1.1307e+03	1.4482e-04	1.0958e+03	9.6915e-05	1.1105e+03

FREQUENCY [Hz]: 2.7392E6		2.8056E6		2.8513E6		2.9711E6	
TAU [s]	MAGNITUDES [a.u]	TAU [s]	MAGNITUDES [a.u]	TAU [s]	MAGNITUDES [a.u]	TAU [s]	MAGNITUDES [a.u]
4.9896e-02	3.6028e+02	3.7786e-02	3.0610e+02	2.9443e-02	3.9770e+02	3.0807e-02	3.8577e+02
4.1127e-02	4.5520e+02	3.6570e-02	3.2884e+02	2.8495e-02	4.6790e+02	2.9816e-02	3.6734e+02
3.3899e-02	4.5302e+02	3.5354e-02	3.2444e+02	2.7548e-02	4.6532e+02	2.8824e-02	3.9729e+02
2.7942e-02	4.2939e+02	3.4138e-02	3.5421e+02	2.6600e-02	3.7703e+02	2.7833e-02	4.2850e+02
2.3031e-02	5.0431e+02	3.2922e-02	3.5140e+02	2.5653e-02	4.1304e+02	2.6842e-02	4.3909e+02
1.8984e-02	4.4202e+02	3.1706e-02	3.7291e+02	2.4706e-02	3.8649e+02	2.5851e-02	4.7145e+02
1.5647e-02	5.4393e+02	3.0491e-02	3.7726e+02	2.3758e-02	4.3967e+02	2.4859e-02	4.8982e+02
1.2897e-02	6.4438e+02	2.9275e-02	3.8409e+02	2.2811e-02	4.3008e+02	2.3868e-02	4.5075e+02
1.0631e-02	6.0128e+02	2.8059e-02	3.5920e+02	2.1864e-02	3.9230e+02	2.2877e-02	3.6095e+02
8.7625e-03	7.2617e+02	2.6843e-02	3.9875e+02	2.0916e-02	3.6945e+02	2.1885e-02	4.0748e+02
7.2226e-03	8.3244e+02	2.5627e-02	3.6100e+02	1.9969e-02	4.5503e+02	2.0894e-02	4.4619e+02
5.9532e-03	7.4997e+02	2.4411e-02	3.9114e+02	1.9021e-02	4.4559e+02	1.9903e-02	4.3956e+02
4.9070e-03	8.4703e+02	2.3196e-02	3.6179e+02	1.8074e-02	3.7814e+02	1.8912e-02	4.8642e+02
4.0446e-03	9.2276e+02	2.1980e-02	3.3377e+02	1.7127e-02	4.3954e+02	1.7920e-02	4.0098e+02
3.3338e-03	9.3415e+02	2.0764e-02	3.8839e+02	1.6179e-02	4.3056e+02	1.6929e-02	4.8102e+02
2.7479e-03	9.1877e+02	1.9548e-02	4.5345e+02	1.5232e-02	5.5730e+02	1.5938e-02	5.2133e+02
2.2650e-03	9.6005e+02	1.8332e-02	4.2926e+02	1.4284e-02	4.0554e+02	1.4946e-02	4.1506e+02
1.8669e-03	1.0231e+03	1.7116e-02	4.9557e+02	1.3337e-02	4.6195e+02	1.3955e-02	5.2396e+02
1.5388e-03	1.0071e+03	1.5900e-02	4.6785e+02	1.2390e-02	4.5948e+02	1.2964e-02	5.2494e+02
1.2684e-03	1.0233e+03	1.4685e-02	4.5723e+02	1.1442e-02	5.2947e+02	1.1973e-02	5.7332e+02
1.0455e-03	9.7681e+02	1.3469e-02	4.8333e+02	1.0495e-02	5.5930e+02	1.0981e-02	4.8064e+02
8.6175e-04	1.0288e+03	1.2253e-02	5.3290e+02	9.5475e-03	5.8423e+02	9.9899e-03	6.2562e+02
7.1030e-04	1.0116e+03	1.1037e-02	5.1074e+02	8.6001e-03	5.9506e+02	8.9986e-03	5.5901e+02
5.8547e-04	1.0195e+03	9.8212e-03	5.5852e+02	7.6527e-03	5.6024e+02	8.0073e-03	6.3793e+02
4.8258e-04	1.0905e+03	8.6054e-03	5.8785e+02	6.7053e-03	6.2490e+02	7.0161e-03	6.5815e+02
3.9777e-04	9.6211e+02	7.3895e-03	6.2480e+02	5.7579e-03	6.8420e+02	6.0248e-03	6.8043e+02
3.2786e-04	9.9396e+02	6.1737e-03	7.3145e+02	4.8105e-03	7.4834e+02	5.0335e-03	8.8754e+02
2.7024e-04	1.1584e+03	4.9578e-03	7.0750e+02	3.8632e-03	7.7442e+02	4.0422e-03	8.3953e+02
2.2275e-04	1.0548e+03	3.7420e-03	8.4091e+02	2.9158e-03	9.0552e+02	3.0509e-03	8.9634e+02
1.8360e-04	9.5067e+02	2.5262e-03	9.3662e+02	1.9684e-03	9.6585e+02	2.0596e-03	9.7584e+02
1.5134e-04	1.0248e+03	1.3103e-03	1.0453e+03	1.0210e-03	9.8809e+02	1.0683e-03	9.9099e+02
1.2474e-04	1.0094e+03	9.4464e-05	1.0954e+03	7.3607e-05	1.0842e+03	7.7018e-05	1.1521e+03

FREQUENCY [Hz]: 3.0767E6		3.0991E6		3.369E6		3.4545E6	
TAU [s]	MAGNITUDES [a.u]	TAU [s]	MAGNITUDES [a.u]	TAU [s]	MAGNITUDES [a.u]	TAU [s]	MAGNITUDES [a.u]
4.5802e-02	4.0570e+02	5.3304e-02	4.1637e+02	9.4052e-02	3.8214e+02	1.0363e-01	3.7611e+02
3.7753e-02	3.8733e+02	5.1589e-02	3.4270e+02	9.1025e-02	3.5227e+02	1.0029e-01	3.7304e+02
3.1118e-02	4.1651e+02	4.9873e-02	4.0743e+02	8.7999e-02	4.5697e+02	9.6960e-02	3.6267e+02
2.5649e-02	5.2924e+02	4.8158e-02	3.9222e+02	8.4973e-02	3.5164e+02	9.3625e-02	3.7882e+02
2.1142e-02	5.1718e+02	4.6443e-02	4.5908e+02	8.1946e-02	4.1107e+02	9.0291e-02	3.3492e+02
1.7426e-02	5.4156e+02	4.4728e-02	4.0573e+02	7.8920e-02	4.2125e+02	8.6956e-02	4.2306e+02
1.4364e-02	6.4711e+02	4.3013e-02	4.3181e+02	7.5894e-02	4.5869e+02	8.3622e-02	3.2960e+02
1.1839e-02	6.4416e+02	4.1298e-02	4.0871e+02	7.2867e-02	4.2943e+02	8.0287e-02	3.6764e+02
9.7586e-03	7.6527e+02	3.9582e-02	4.9076e+02	6.9841e-02	4.4325e+02	7.6953e-02	3.3950e+02
8.0436e-03	8.1977e+02	3.7867e-02	3.9767e+02	6.6815e-02	4.9130e+02	7.3618e-02	3.1421e+02
6.6300e-03	7.8271e+02	3.6152e-02	4.7146e+02	6.3788e-02	4.4793e+02	7.0284e-02	4.2124e+02
5.4648e-03	9.0753e+02	3.4437e-02	4.6148e+02	6.0762e-02	4.3839e+02	6.6949e-02	3.2565e+02
4.5044e-03	9.4201e+02	3.2722e-02	5.0087e+02	5.7736e-02	4.7929e+02	6.3615e-02	4.1950e+02
3.7128e-03	9.9010e+02	3.1006e-02	4.7381e+02	5.4709e-02	4.7382e+02	6.0280e-02	3.7994e+02
3.0603e-03	9.6988e+02	2.9291e-02	5.1029e+02	5.1683e-02	4.5229e+02	5.6946e-02	3.6161e+02
2.5225e-03	9.6127e+02	2.7576e-02	4.5728e+02	4.8657e-02	4.4004e+02	5.3611e-02	4.0629e+02
2.0792e-03	1.0573e+03	2.5861e-02	4.9335e+02	4.5630e-02	5.3440e+02	5.0277e-02	4.6372e+02
1.7138e-03	1.0494e+03	2.4146e-02	5.1978e+02	4.2604e-02	4.9740e+02	4.6942e-02	5.0944e+02
1.4126e-03	1.0213e+03	2.2431e-02	5.0932e+02	3.9578e-02	4.4508e+02	4.3608e-02	4.9762e+02
1.1643e-03	1.1457e+03	2.0715e-02	6.5413e+02	3.6551e-02	5.7431e+02	4.0273e-02	4.8140e+02
9.5970e-04	1.1118e+03	1.9000e-02	5.4443e+02	3.3525e-02	5.6922e+02	3.6939e-02	4.4844e+02
7.9104e-04	1.0616e+03	1.7285e-02	5.8404e+02	3.0499e-02	6.0938e+02	3.3604e-02	5.0867e+02
6.5202e-04	1.0802e+03	1.5570e-02	6.2374e+02	2.7472e-02	6.1252e+02	3.0270e-02	5.8245e+02
5.3743e-04	1.1703e+03	1.3855e-02	6.8102e+02	2.4446e-02	6.5360e+02	2.6935e-02	6.3894e+02
4.4298e-04	1.0910e+03	1.2140e-02	7.2296e+02	2.1419e-02	7.1794e+02	2.3601e-02	5.9031e+02
3.6513e-04	1.1972e+03	1.0424e-02	6.8611e+02	1.8393e-02	6.7945e+02	2.0266e-02	7.0078e+02
3.0096e-04	1.1427e+03	8.7091e-03	8.9074e+02	1.5367e-02	7.5817e+02	1.6932e-02	7.3952e+02
2.4807e-04	1.1186e+03	6.9940e-03	9.1661e+02	1.2340e-02	8.2258e+02	1.3597e-02	8.3133e+02
2.0447e-04	1.1548e+03	5.2788e-03	9.3950e+02	9.3141e-03	9.5903e+02	1.0263e-02	8.6218e+02
1.6854e-04	1.1418e+03	3.5636e-03	1.0345e+03	6.2878e-03	1.0717e+03	6.9281e-03	9.9922e+02
1.3892e-04	1.1141e+03	1.8484e-03	1.0749e+03	3.2615e-03	9.9272e+02	3.5936e-03	1.1467e+03
1.1451e-04	1.2611e+03	1.3326e-04	1.2449e+03	2.3513e-04	1.2332e+03	2.5907e-04	1.2251e+03

FREQUENCY [Hz]: 3.5381E6		3.7114E6		3.8856E6		3.9998E6	
TAU [s]	MAGNITUDES [a.u]	TAU [s]	MAGNITUDES [a.u]	TAU [s]	MAGNITUDES [a.u]	TAU [s]	MAGNITUDES [a.u]
1.0669e-01	3.3873e+02	1.1382e-01	3.4503e+02	1.0311e-01	4.0460e+02	1.0000e-01	3.8664e+02
1.0325e-01	4.0179e+02	1.1016e-01	3.8500e+02	8.4986e-02	4.1866e+02	9.6782e-02	4.0725e+02
9.9821e-02	3.6008e+02	1.0650e-01	3.6209e+02	7.0051e-02	4.6701e+02	9.3565e-02	3.6602e+02
9.6388e-02	3.8643e+02	1.0284e-01	3.6899e+02	5.7740e-02	5.3709e+02	9.0347e-02	4.0340e+02
9.2955e-02	3.8746e+02	9.9174e-02	3.3760e+02	4.7592e-02	5.0182e+02	8.7129e-02	3.7912e+02
8.9522e-02	4.0172e+02	9.5512e-02	3.6184e+02	3.9228e-02	5.3550e+02	8.3911e-02	4.4186e+02
8.6089e-02	3.1822e+02	9.1849e-02	3.3027e+02	3.2334e-02	5.8654e+02	8.0694e-02	3.9393e+02
8.2656e-02	3.7665e+02	8.8186e-02	3.7203e+02	2.6652e-02	6.5146e+02	7.7476e-02	3.5720e+02
7.9223e-02	3.8837e+02	8.4524e-02	3.5746e+02	2.1968e-02	6.9781e+02	7.4258e-02	4.6094e+02
7.5791e-02	3.5578e+02	8.0861e-02	3.7480e+02	1.8107e-02	8.2147e+02	7.1040e-02	4.3988e+02
7.2358e-02	3.8826e+02	7.7199e-02	4.2825e+02	1.4925e-02	7.7050e+02	6.7823e-02	4.5623e+02
6.8925e-02	4.2589e+02	7.3536e-02	3.9500e+02	1.2302e-02	8.2966e+02	6.4605e-02	4.6357e+02
6.5492e-02	3.9103e+02	6.9874e-02	4.2536e+02	1.0140e-02	9.6777e+02	6.1387e-02	4.9176e+02
6.2059e-02	4.0615e+02	6.6211e-02	4.0593e+02	8.3580e-03	9.9757e+02	5.8169e-02	3.8957e+02
5.8626e-02	4.1473e+02	6.2548e-02	4.0183e+02	6.8891e-03	1.0223e+03	5.4952e-02	4.7816e+02
5.5193e-02	4.6059e+02	5.8886e-02	4.4899e+02	5.6784e-03	9.8730e+02	5.1734e-02	4.9289e+02
5.1760e-02	4.7788e+02	5.5223e-02	4.1794e+02	4.6805e-03	1.0407e+03	4.8516e-02	5.5753e+02
4.8327e-02	4.2141e+02	5.1561e-02	5.2935e+02	3.8579e-03	1.0837e+03	4.5298e-02	5.2065e+02
4.4894e-02	5.1680e+02	4.7898e-02	4.9904e+02	3.1799e-03	1.1545e+03	4.2081e-02	5.6991e+02
4.1462e-02	4.3330e+02	4.4236e-02	4.9333e+02	2.6211e-03	1.0721e+03	3.8863e-02	5.7286e+02
3.8029e-02	4.9429e+02	4.0573e-02	5.1891e+02	2.1604e-03	1.1626e+03	3.5645e-02	6.3861e+02
3.4596e-02	5.5058e+02	3.6910e-02	5.6303e+02	1.7807e-03	1.1650e+03	3.2427e-02	6.0733e+02
3.1163e-02	5.2997e+02	3.3248e-02	5.6905e+02	1.4678e-03	1.1349e+03	2.9210e-02	6.4891e+02
2.7730e-02	7.0109e+02	2.9585e-02	5.9168e+02	1.2098e-03	1.0870e+03	2.5992e-02	6.9348e+02
2.4297e-02	6.6799e+02	2.5923e-02	6.7621e+02	9.9722e-04	1.2809e+03	2.2774e-02	7.5416e+02
2.0864e-02	6.9115e+02	2.2260e-02	7.1257e+02	8.2196e-04	1.1682e+03	1.9556e-02	7.5436e+02
1.7431e-02	7.7193e+02	1.8597e-02	7.7794e+02	6.7751e-04	1.1812e+03	1.6339e-02	8.2984e+02
1.3998e-02	8.0365e+02	1.4935e-02	8.1635e+02	5.5844e-04	1.1537e+03	1.3121e-02	9.4735e+02
1.0565e-02	9.0616e+02	1.1272e-02	9.0014e+02	4.6030e-04	1.1606e+03	9.9032e-03	9.9477e+02
7.1325e-03	9.9404e+02	7.6097e-03	1.0121e+03	3.7940e-04	1.1710e+03	6.6855e-03	1.0859e+03
3.6996e-03	1.0802e+03	3.9471e-03	1.0836e+03	3.1273e-04	1.1337e+03	3.4677e-03	1.1573e+03
2.6672e-04	1.1846e+03	2.8456e-04	1.2665e+03	2.5777e-04	1.1012e+03	2.5000e-04	1.2815e+03

FREQUENCY [Hz]: 4.3654E6		4.9049E6		5.5133E6		6.1929E6	
TAU [s]	MAGNITUDES [a.u]	TAU [s]	MAGNITUDES [a.u]	TAU [s]	MAGNITUDES [a.u]	TAU [s]	MAGNITUDES [a.u]
1.2200e-01	4.4681e+02	1.3006e-01	4.5854e+02	1.2746e-01	4.8155e+02	1.7823e-01	4.5272e+02
1.0056e-01	4.2724e+02	1.0720e-01	4.3954e+02	1.0506e-01	5.7267e+02	1.4691e-01	4.7903e+02
8.2887e-02	5.1092e+02	8.8361e-02	5.1068e+02	8.6594e-02	5.0449e+02	1.2109e-01	5.7753e+02
6.8320e-02	4.6005e+02	7.2832e-02	4.2061e+02	7.1376e-02	5.6495e+02	9.9808e-02	5.1184e+02
5.6313e-02	5.2830e+02	6.0032e-02	5.3883e+02	5.8832e-02	6.9040e+02	8.2267e-02	5.1841e+02
4.6416e-02	5.5181e+02	4.9482e-02	6.5992e+02	4.8493e-02	6.4193e+02	6.7809e-02	6.5626e+02
3.8259e-02	5.7792e+02	4.0786e-02	6.4252e+02	3.9971e-02	6.8156e+02	5.5892e-02	6.7731e+02
3.1535e-02	6.5705e+02	3.3618e-02	7.0541e+02	3.2946e-02	7.1304e+02	4.6070e-02	6.8327e+02
2.5993e-02	7.0736e+02	2.7710e-02	7.5234e+02	2.7156e-02	7.4613e+02	3.7973e-02	7.1928e+02
2.1425e-02	7.5295e+02	2.2840e-02	8.2474e+02	2.2384e-02	8.0422e+02	3.1300e-02	8.1077e+02
1.7660e-02	8.1161e+02	1.8826e-02	7.7870e+02	1.8450e-02	9.2528e+02	2.5799e-02	8.7993e+02
1.4556e-02	8.6750e+02	1.5518e-02	9.1567e+02	1.5207e-02	9.1283e+02	2.1265e-02	9.3755e+02
1.1998e-02	9.1303e+02	1.2790e-02	9.2799e+02	1.2535e-02	1.0017e+03	1.7528e-02	9.6401e+02
9.8895e-03	9.3421e+02	1.0543e-02	9.3719e+02	1.0332e-02	9.3166e+02	1.4447e-02	9.5373e+02
8.1515e-03	1.0364e+03	8.6898e-03	1.0114e+03	8.5161e-03	1.0864e+03	1.1908e-02	1.0101e+03
6.7189e-03	1.0645e+03	7.1627e-03	1.0247e+03	7.0195e-03	1.0624e+03	9.8156e-03	1.0642e+03
5.5381e-03	1.0378e+03	5.9039e-03	1.0062e+03	5.7858e-03	1.0832e+03	8.0905e-03	9.8947e+02
4.5648e-03	1.0562e+03	4.8663e-03	1.0419e+03	4.7690e-03	1.0915e+03	6.6687e-03	1.1184e+03
3.7626e-03	1.1178e+03	4.0111e-03	1.0537e+03	3.9309e-03	1.0829e+03	5.4967e-03	1.0612e+03
3.1013e-03	1.0197e+03	3.3062e-03	1.0164e+03	3.2401e-03	1.1264e+03	4.5307e-03	1.1240e+03
2.5563e-03	1.0366e+03	2.7251e-03	1.0931e+03	2.6706e-03	1.1229e+03	3.7345e-03	1.1153e+03
2.1070e-03	1.1123e+03	2.2462e-03	1.0544e+03	2.2013e-03	1.1318e+03	3.0782e-03	1.0693e+03
1.7367e-03	1.1014e+03	1.8514e-03	1.1307e+03	1.8144e-03	1.1445e+03	2.5372e-03	1.1036e+03
1.4315e-03	1.1827e+03	1.5261e-03	1.1620e+03	1.4956e-03	1.1376e+03	2.0913e-03	1.1146e+03
1.1799e-03	1.1110e+03	1.2579e-03	1.1996e+03	1.2327e-03	1.1427e+03	1.7238e-03	1.2299e+03
9.7258e-04	1.0795e+03	1.0368e-03	1.1911e+03	1.0161e-03	1.1761e+03	1.4208e-03	1.1818e+03
8.0165e-04	1.1167e+03	8.5460e-04	1.1854e+03	8.3751e-04	1.2367e+03	1.1711e-03	1.1438e+03
6.6077e-04	1.1955e+03	7.0441e-04	1.2080e+03	6.9033e-04	1.1742e+03	9.6531e-04	1.1532e+03
5.4464e-04	1.1835e+03	5.8061e-04	1.1828e+03	5.6901e-04	1.1581e+03	7.9566e-04	1.1807e+03
4.4892e-04	1.2316e+03	4.7858e-04	1.1443e+03	4.6901e-04	1.1554e+03	6.5583e-04	1.1921e+03
3.7003e-04	1.1507e+03	3.9447e-04	1.2104e+03	3.8658e-04	1.0673e+03	5.4057e-04	1.1418e+03
3.0500e-04	1.2743e+03	3.2514e-04	1.1609e+03	3.1864e-04	1.1725e+03	4.4557e-04	1.1194e+03

FREQUENCY [Hz]: 6.9582E6		7.8217E6		8.7876E6		9.8748E6	
TAU [s]	MAGNITUDES [a.u]	TAU [s]	MAGNITUDES [a.u]	TAU [s]	MAGNITUDES [a.u]	TAU [s]	MAGNITUDES [a.u]
1.5503e-01	5.1409e+02	1.9457e-01	5.5963e+02	2.0523e-01	5.7736e+02	2.1828e-01	5.6406e+02
1.2778e-01	5.3487e+02	1.6037e-01	6.0577e+02	1.6916e-01	6.2350e+02	1.7992e-01	6.7666e+02
1.0533e-01	5.4663e+02	1.3219e-01	5.5465e+02	1.3943e-01	5.7682e+02	1.4830e-01	6.6123e+02
8.6815e-02	5.9980e+02	1.0896e-01	5.4056e+02	1.1493e-01	6.9720e+02	1.2224e-01	7.0662e+02
7.1558e-02	6.1406e+02	8.9809e-02	6.2759e+02	9.4729e-02	7.0509e+02	1.0075e-01	7.9547e+02
5.8982e-02	6.6276e+02	7.4025e-02	7.3638e+02	7.8081e-02	7.5901e+02	8.3048e-02	8.7340e+02
4.8616e-02	7.7367e+02	6.1016e-02	7.1033e+02	6.4359e-02	6.9774e+02	6.8453e-02	7.6321e+02
4.0072e-02	7.8444e+02	5.0293e-02	7.8149e+02	5.3048e-02	7.8413e+02	5.6423e-02	8.4533e+02
3.3030e-02	8.6164e+02	4.1454e-02	8.4522e+02	4.3725e-02	7.9752e+02	4.6507e-02	8.5983e+02
2.7225e-02	9.2405e+02	3.4169e-02	8.4181e+02	3.6041e-02	8.5961e+02	3.8334e-02	8.9727e+02
2.2440e-02	9.0304e+02	2.8164e-02	9.7169e+02	2.9707e-02	9.7705e+02	3.1597e-02	9.2593e+02
1.8497e-02	1.0272e+03	2.3214e-02	9.9239e+02	2.4486e-02	1.0426e+03	2.6044e-02	1.0925e+03
1.5246e-02	9.1621e+02	1.9135e-02	1.0218e+03	2.0183e-02	1.0223e+03	2.1467e-02	1.0018e+03
1.2567e-02	1.0027e+03	1.5772e-02	9.7502e+02	1.6636e-02	1.0545e+03	1.7694e-02	1.0554e+03
1.0358e-02	1.1446e+03	1.3000e-02	1.0153e+03	1.3712e-02	1.0616e+03	1.4585e-02	9.6533e+02
8.5378e-03	1.0574e+03	1.0715e-02	1.0358e+03	1.1302e-02	1.1530e+03	1.2021e-02	1.0425e+03
7.0373e-03	1.0982e+03	8.8322e-03	1.0811e+03	9.3161e-03	1.0770e+03	9.9087e-03	1.0904e+03
5.8006e-03	1.1157e+03	7.2800e-03	1.0398e+03	7.6789e-03	1.1141e+03	8.1673e-03	1.1585e+03
4.7812e-03	1.2290e+03	6.0006e-03	1.1498e+03	6.3294e-03	1.1622e+03	6.7320e-03	1.2144e+03
3.9409e-03	1.1132e+03	4.9460e-03	1.0876e+03	5.2170e-03	1.1057e+03	5.5489e-03	1.2123e+03
3.2483e-03	1.1192e+03	4.0768e-03	1.1050e+03	4.3002e-03	1.2110e+03	4.5737e-03	1.1320e+03
2.6774e-03	1.1826e+03	3.3603e-03	1.2037e+03	3.5444e-03	1.1619e+03	3.7699e-03	1.1633e+03
2.2069e-03	1.1600e+03	2.7698e-03	1.2487e+03	2.9215e-03	1.2550e+03	3.1074e-03	1.2069e+03
1.8191e-03	1.1832e+03	2.2830e-03	1.1804e+03	2.4081e-03	1.1562e+03	2.5613e-03	1.2349e+03
1.4994e-03	1.1528e+03	1.8818e-03	1.1830e+03	1.9849e-03	1.2148e+03	2.1111e-03	1.1984e+03
1.2359e-03	1.1869e+03	1.5511e-03	1.1989e+03	1.6361e-03	1.1562e+03	1.7401e-03	1.2496e+03
1.0187e-03	1.2600e+03	1.2785e-03	1.1764e+03	1.3485e-03	1.2123e+03	1.4343e-03	1.1273e+03
8.3965e-04	1.2345e+03	1.0538e-03	1.1809e+03	1.1115e-03	1.2248e+03	1.1822e-03	1.2341e+03
6.9208e-04	1.2475e+03	8.6860e-04	1.1880e+03	9.1619e-04	1.1750e+03	9.7447e-04	1.2577e+03
5.7046e-04	1.1966e+03	7.1595e-04	1.2040e+03	7.5518e-04	1.2215e+03	8.0321e-04	1.2349e+03
4.7020e-04	1.2889e+03	5.9013e-04	1.2251e+03	6.2246e-04	1.2286e+03	6.6205e-04	1.2804e+03
3.8757e-04	1.1705e+03	4.8642e-04	1.2062e+03	5.1307e-04	1.2900e+03	5.4570e-04	1.2400e+03

FREQUENCY [Hz]: 1.1094E7		1.2464E7		1.4009E7		1.5738E7	
TAU [s]	MAGNITUDES [a.u]	TAU [s]	MAGNITUDES [a.u]	TAU [s]	MAGNITUDES [a.u]	TAU [s]	MAGNITUDES [a.u]
4.2278e-01	7.3121e+02	3.9560e-01	7.8085e+02	3.8814e-01	7.9412e+02	4.1144e-01	9.6889e+02
3.4848e-01	6.6921e+02	3.2608e-01	7.7095e+02	3.1992e-01	8.2211e+02	3.3913e-01	8.5271e+02
2.8724e-01	7.8799e+02	2.6877e-01	8.0447e+02	2.6370e-01	8.0154e+02	2.7953e-01	8.7303e+02
2.3676e-01	7.7171e+02	2.2154e-01	7.6458e+02	2.1736e-01	7.8532e+02	2.3041e-01	8.8482e+02
1.9515e-01	7.5460e+02	1.8260e-01	7.4061e+02	1.7916e-01	6.9985e+02	1.8991e-01	8.5495e+02
1.6085e-01	6.8278e+02	1.5051e-01	7.7442e+02	1.4767e-01	7.6384e+02	1.5654e-01	7.9278e+02
1.3258e-01	7.3396e+02	1.2406e-01	7.1827e+02	1.2172e-01	6.9327e+02	1.2903e-01	7.9868e+02
1.0928e-01	6.2195e+02	1.0226e-01	6.3408e+02	1.0033e-01	6.6512e+02	1.0635e-01	7.2110e+02
9.0078e-02	5.6103e+02	8.4287e-02	6.5243e+02	8.2696e-02	6.4556e+02	8.7661e-02	6.3685e+02
7.4247e-02	6.0088e+02	6.9474e-02	5.3148e+02	6.8163e-02	5.3899e+02	7.2255e-02	5.4976e+02
6.1199e-02	4.6918e+02	5.7264e-02	4.7624e+02	5.6184e-02	5.8309e+02	5.9557e-02	5.9986e+02
5.0443e-02	5.4861e+02	4.7201e-02	4.7997e+02	4.6310e-02	4.8543e+02	4.9090e-02	4.8957e+02
4.1578e-02	4.9508e+02	3.8905e-02	4.6793e+02	3.8171e-02	4.5616e+02	4.0463e-02	4.8893e+02
3.4271e-02	4.1741e+02	3.2068e-02	4.6366e+02	3.1463e-02	4.7397e+02	3.3352e-02	5.0979e+02
2.8248e-02	3.5225e+02	2.6432e-02	3.8646e+02	2.5933e-02	3.9959e+02	2.7491e-02	4.6463e+02
2.3284e-02	4.2798e+02	2.1787e-02	3.9091e+02	2.1376e-02	3.9246e+02	2.2659e-02	4.2336e+02
1.9192e-02	3.3765e+02	1.7958e-02	4.2624e+02	1.7619e-02	4.5217e+02	1.8677e-02	3.5092e+02
1.5819e-02	3.4918e+02	1.4802e-02	4.1077e+02	1.4523e-02	4.2501e+02	1.5395e-02	3.5647e+02
1.3039e-02	4.2565e+02	1.2201e-02	4.0208e+02	1.1970e-02	3.7388e+02	1.2689e-02	3.8927e+02
1.0747e-02	3.2388e+02	1.0057e-02	3.3976e+02	9.8667e-03	4.2165e+02	1.0459e-02	3.8111e+02
8.8587e-03	3.7530e+02	8.2892e-03	3.8672e+02	8.1327e-03	3.8194e+02	8.6210e-03	3.4462e+02
7.3018e-03	3.3142e+02	6.8324e-03	3.1034e+02	6.7034e-03	3.4912e+02	7.1059e-03	3.0964e+02
6.0186e-03	3.6970e+02	5.6316e-03	3.1097e+02	5.5254e-03	3.2380e+02	5.8571e-03	2.7289e+02
4.9608e-03	3.3533e+02	4.6419e-03	3.0348e+02	4.5543e-03	4.0889e+02	4.8278e-03	3.5319e+02
4.0890e-03	3.1230e+02	3.8261e-03	3.6597e+02	3.7539e-03	3.1314e+02	3.9793e-03	3.5895e+02
3.3704e-03	3.9254e+02	3.1537e-03	4.0194e+02	3.0942e-03	3.4334e+02	3.2800e-03	3.6453e+02
2.7781e-03	4.3371e+02	2.5995e-03	3.5583e+02	2.5504e-03	3.3293e+02	2.7035e-03	3.3500e+02
2.2898e-03	3.7611e+02	2.1426e-03	3.2890e+02	2.1022e-03	3.9457e+02	2.2284e-03	3.7876e+02
1.8874e-03	3.3033e+02	1.7661e-03	3.5494e+02	1.7328e-03	3.2198e+02	1.8368e-03	3.4847e+02
1.5557e-03	4.3078e+02	1.4557e-03	3.8805e+02	1.4282e-03	3.7697e+02	1.5140e-03	3.3577e+02
1.2823e-03	3.8890e+02	1.1999e-03	3.3527e+02	1.1772e-03	3.7861e+02	1.2479e-03	3.1844e+02
1.0570e-03	2.5957e+02	9.8901e-04	3.6690e+02	9.7034e-04	3.0522e+02	1.0286e-03	3.3659e+02

FREQUENCY [Hz]: 1.7689E7		1.9875E7		2.2331E7		2.8196E7	
TAU [s]	MAGNITUDES [a.u]	TAU [s]	MAGNITUDES [a.u]	TAU [s]	MAGNITUDES [a.u]	TAU [s]	MAGNITUDES [a.u]
5.2404e-01	9.6779e+02	5.1292e-01	1.0146e+03	5.3570e-01	1.1915e+03	8.7663e-01	1.3745e+03
4.3195e-01	9.3566e+02	4.2277e-01	1.0340e+03	4.4156e-01	1.0998e+03	7.2257e-01	1.3746e+03
3.5603e-01	9.3934e+02	3.4847e-01	1.0068e+03	3.6396e-01	1.0999e+03	5.9558e-01	1.3776e+03
2.9346e-01	8.7200e+02	2.8723e-01	1.0660e+03	2.9999e-01	1.0823e+03	4.9091e-01	1.2907e+03
2.4189e-01	8.8604e+02	2.3675e-01	9.5683e+02	2.4727e-01	1.0557e+03	4.0464e-01	1.2514e+03
1.9938e-01	8.4958e+02	1.9515e-01	9.9407e+02	2.0382e-01	9.9508e+02	3.3353e-01	1.2375e+03
1.6434e-01	7.5310e+02	1.6085e-01	8.4183e+02	1.6800e-01	1.0125e+03	2.7491e-01	1.1386e+03
1.3546e-01	7.0781e+02	1.3258e-01	7.7176e+02	1.3847e-01	8.5078e+02	2.2660e-01	1.0654e+03
1.1165e-01	6.7975e+02	1.0928e-01	7.2966e+02	1.1414e-01	8.0903e+02	1.8677e-01	9.6927e+02
9.2030e-02	6.8255e+02	9.0076e-02	6.9603e+02	9.4078e-02	7.5894e+02	1.5395e-01	9.3445e+02
7.5857e-02	6.4010e+02	7.4246e-02	7.0635e+02	7.7544e-02	6.3339e+02	1.2689e-01	8.8007e+02
6.2525e-02	5.6775e+02	6.1198e-02	5.6309e+02	6.3917e-02	6.3954e+02	1.0459e-01	7.0696e+02
5.1537e-02	5.6962e+02	5.0443e-02	5.0489e+02	5.2684e-02	5.8961e+02	8.6212e-02	7.1258e+02
4.2480e-02	4.5940e+02	4.1578e-02	4.5881e+02	4.3425e-02	5.7297e+02	7.1061e-02	6.7818e+02
3.5014e-02	4.3914e+02	3.4271e-02	4.2848e+02	3.5793e-02	4.7989e+02	5.8572e-02	6.2773e+02
2.8861e-02	4.5476e+02	2.8248e-02	3.9450e+02	2.9503e-02	4.9878e+02	4.8279e-02	5.2195e+02
2.3789e-02	4.4305e+02	2.3283e-02	4.1890e+02	2.4318e-02	4.4629e+02	3.9794e-02	5.0049e+02
1.9608e-02	3.8592e+02	1.9192e-02	4.3987e+02	2.0044e-02	4.3613e+02	3.2801e-02	4.9838e+02
1.6162e-02	4.3835e+02	1.5819e-02	3.3736e+02	1.6522e-02	4.1244e+02	2.7036e-02	4.1000e+02
1.3322e-02	3.7099e+02	1.3039e-02	3.4904e+02	1.3618e-02	3.4307e+02	2.2285e-02	4.0926e+02
1.0980e-02	3.8887e+02	1.0747e-02	3.2434e+02	1.1225e-02	3.6337e+02	1.8368e-02	3.8648e+02
9.0507e-03	3.8198e+02	8.8585e-03	3.6240e+02	9.2521e-03	3.2600e+02	1.5140e-02	3.5550e+02
7.4601e-03	3.4934e+02	7.3017e-03	3.5618e+02	7.6261e-03	2.8063e+02	1.2479e-02	3.4463e+02
6.1490e-03	3.0439e+02	6.0185e-03	3.4333e+02	6.2859e-03	3.5648e+02	1.0286e-02	3.1837e+02
5.0684e-03	3.5112e+02	4.9608e-03	3.1768e+02	5.1812e-03	3.0070e+02	8.4785e-03	3.1248e+02
4.1776e-03	3.5357e+02	4.0889e-03	3.4755e+02	4.2706e-03	3.1061e+02	6.9885e-03	3.6325e+02
3.4435e-03	3.1623e+02	3.3703e-03	3.3853e+02	3.5201e-03	3.6565e+02	5.7603e-03	3.7602e+02
2.8383e-03	2.9378e+02	2.7780e-03	3.7331e+02	2.9014e-03	2.9376e+02	4.7480e-03	3.0187e+02
2.3395e-03	3.0995e+02	2.2898e-03	3.0568e+02	2.3915e-03	3.2531e+02	3.9135e-03	3.3290e+02
1.9283e-03	3.1008e+02	1.8874e-03	2.9886e+02	1.9712e-03	2.9438e+02	3.2258e-03	3.7955e+02
1.5894e-03	3.4406e+02	1.5557e-03	3.0266e+02	1.6248e-03	3.2181e+02	2.6589e-03	3.8856e+02
1.3101e-03	3.9973e+02	1.2823e-03	3.2062e+02	1.3393e-03	3.2069e+02	2.1916e-03	3.4604e+02

FREQUENCY [Hz]: 3.1681E7		3.5597E7		4.0003E7	
TAU [s]	MAGNITUDES [a.u]	TAU [s]	MAGNITUDES [a.u]	TAU [s]	MAGNITUDES [a.u]
8.6391e-01	1.5293e+03	9.3415e-01	1.6167e+03	9.6361e-01	1.8611e+03
7.1208e-01	1.5470e+03	7.6998e-01	1.5631e+03	7.9426e-01	1.7771e+03
5.8694e-01	1.3587e+03	6.3466e-01	1.5834e+03	6.5468e-01	1.7771e+03
4.8379e-01	1.4131e+03	5.2312e-01	1.5415e+03	5.3962e-01	1.6791e+03
3.9877e-01	1.3707e+03	4.3119e-01	1.5028e+03	4.4479e-01	1.6072e+03
3.2869e-01	1.2643e+03	3.5541e-01	1.3482e+03	3.6662e-01	1.5025e+03
2.7092e-01	1.3272e+03	2.9295e-01	1.3321e+03	3.0219e-01	1.4154e+03
2.2331e-01	1.1081e+03	2.4146e-01	1.2100e+03	2.4908e-01	1.3350e+03
1.8406e-01	9.8450e+02	1.9903e-01	1.1012e+03	2.0531e-01	1.1660e+03
1.5172e-01	8.9442e+02	1.6405e-01	1.0165e+03	1.6923e-01	1.1590e+03
1.2505e-01	7.8339e+02	1.3522e-01	9.3611e+02	1.3949e-01	1.0048e+03
1.0308e-01	8.4074e+02	1.1146e-01	7.7179e+02	1.1497e-01	8.8637e+02
8.4961e-02	6.2427e+02	9.1868e-02	7.4950e+02	9.4766e-02	8.0074e+02
7.0030e-02	6.2534e+02	7.5723e-02	7.0051e+02	7.8112e-02	6.8886e+02
5.7722e-02	6.4071e+02	6.2415e-02	5.8990e+02	6.4384e-02	6.5402e+02
4.7578e-02	5.4408e+02	5.1446e-02	5.0845e+02	5.3069e-02	6.1086e+02
3.9217e-02	4.4042e+02	4.2405e-02	5.1964e+02	4.3743e-02	5.6393e+02
3.2325e-02	5.5300e+02	3.4953e-02	5.0303e+02	3.6055e-02	5.0072e+02
2.6644e-02	4.4049e+02	2.8810e-02	4.3028e+02	2.9719e-02	5.3040e+02
2.1961e-02	4.2778e+02	2.3747e-02	4.4671e+02	2.4496e-02	4.2247e+02
1.8102e-02	3.9029e+02	1.9573e-02	3.5279e+02	2.0191e-02	4.1859e+02
1.4920e-02	3.8435e+02	1.6134e-02	3.5100e+02	1.6642e-02	3.8639e+02
1.2298e-02	3.2844e+02	1.3298e-02	3.5658e+02	1.3718e-02	3.7395e+02
1.0137e-02	3.2021e+02	1.0961e-02	3.3147e+02	1.1307e-02	3.2745e+02
8.3555e-03	3.7840e+02	9.0348e-03	3.6749e+02	9.3197e-03	2.9884e+02
6.8870e-03	3.1236e+02	7.4470e-03	3.0006e+02	7.6819e-03	3.6683e+02
5.6767e-03	3.0235e+02	6.1382e-03	3.0907e+02	6.3318e-03	3.0306e+02
4.6791e-03	3.0603e+02	5.0595e-03	3.1418e+02	5.2191e-03	3.6548e+02
3.8567e-03	3.6877e+02	4.1703e-03	3.7649e+02	4.3018e-03	2.9929e+02
3.1789e-03	3.5951e+02	3.4374e-03	2.7736e+02	3.5458e-03	2.7182e+02
2.6203e-03	3.2557e+02	2.8333e-03	3.0335e+02	2.9227e-03	3.1825e+02
2.1598e-03	3.1799e+02	2.3354e-03	3.2250e+02	2.4090e-03	3.1646e+02

3. [1]H magnetization of BSA:

FREQUENCY [Hz]: 5041.9		5385.7		6111.4		6493.4	
TAU [s]	MAGNITUDES [a.u]	TAU [s]	MAGNITUDES [a.u]	TAU [s]	MAGNITUDES [a.u]	TAU [s]	MAGNITUDES [a.u]
2.3795e-03	1.9719e+02	2.2250e-03	2.0341e+02	2.4298e-03	1.9586e+02	2.2677e-03	1.8904e+02
2.2213e-03	1.7968e+02	2.0771e-03	1.9240e+02	2.2683e-03	2.0475e+02	2.1169e-03	2.0007e+02
2.0631e-03	1.7596e+02	1.9291e-03	1.7708e+02	2.1067e-03	2.2828e+02	1.9661e-03	2.0622e+02
1.9048e-03	1.7873e+02	1.7811e-03	2.1229e+02	1.9451e-03	1.9408e+02	1.8153e-03	1.8632e+02
1.7466e-03	1.7730e+02	1.6332e-03	2.2297e+02	1.7835e-03	1.8774e+02	1.6645e-03	2.0578e+02
1.5883e-03	2.2064e+02	1.4852e-03	1.9632e+02	1.6219e-03	2.2125e+02	1.5137e-03	2.2428e+02
1.4301e-03	2.0645e+02	1.3372e-03	2.6339e+02	1.4603e-03	2.5137e+02	1.3629e-03	2.3243e+02
1.2719e-03	2.3629e+02	1.1893e-03	2.2309e+02	1.2988e-03	2.3462e+02	1.2121e-03	2.5113e+02
1.1136e-03	2.5545e+02	1.0413e-03	2.9371e+02	1.1372e-03	2.5802e+02	1.0613e-03	2.7699e+02
9.5538e-04	3.0025e+02	8.9335e-04	3.2285e+02	9.7558e-04	2.9716e+02	9.1048e-04	3.6035e+02
7.9714e-04	3.1085e+02	7.4539e-04	4.3608e+02	8.1400e-04	3.4307e+02	7.5968e-04	3.4058e+02
6.3890e-04	3.7896e+02	5.9742e-04	4.5416e+02	6.5241e-04	4.2901e+02	6.0887e-04	4.6546e+02
4.8067e-04	5.1675e+02	4.4946e-04	5.5699e+02	4.9083e-04	5.4321e+02	4.5807e-04	5.3755e+02
3.2243e-04	6.4387e+02	3.0149e-04	6.6022e+02	3.2924e-04	6.8194e+02	3.0727e-04	6.7460e+02
1.6419e-04	7.7352e+02	1.5353e-04	8.2954e+02	1.6766e-04	8.6280e+02	1.5647e-04	8.1715e+02
5.9488e-06	9.4739e+02	5.5626e-06	9.4920e+02	6.0746e-06	1.0114e+03	5.6692e-06	9.6991e+02

FREQUENCY [Hz]: 7017.9		7425.3		7983		8418.4	
TAU [s]	MAGNITUDES [a.u]	TAU [s]	MAGNITUDES [a.u]	TAU [s]	MAGNITUDES [a.u]	TAU [s]	MAGNITUDES [a.u]
2.3007e-03	1.9423e+02	2.5087e-03	1.8162e+02	2.4589e-03	1.7496e+02	2.5410e-03	2.0423e+02
2.1477e-03	2.0101e+02	2.3419e-03	1.8495e+02	2.2954e-03	1.9197e+02	2.3720e-03	1.8645e+02
1.9947e-03	2.1554e+02	2.1750e-03	2.1241e+02	2.1319e-03	1.7880e+02	2.2031e-03	2.2471e+02
1.8417e-03	2.0219e+02	2.0082e-03	1.9687e+02	1.9684e-03	1.7720e+02	2.0341e-03	1.6888e+02
1.6887e-03	2.1648e+02	1.8414e-03	2.0753e+02	1.8048e-03	2.1228e+02	1.8651e-03	2.0102e+02
1.5357e-03	2.2946e+02	1.6746e-03	2.2399e+02	1.6413e-03	2.2733e+02	1.6961e-03	2.0154e+02
1.3827e-03	2.2941e+02	1.5077e-03	2.3647e+02	1.4778e-03	2.1882e+02	1.5272e-03	2.0564e+02
1.2298e-03	2.6116e+02	1.3409e-03	2.5315e+02	1.3143e-03	2.5451e+02	1.3582e-03	2.3948e+02
1.0768e-03	3.2280e+02	1.1741e-03	2.8234e+02	1.1508e-03	3.0118e+02	1.1892e-03	2.9839e+02
9.2375e-04	3.5790e+02	1.0072e-03	2.7893e+02	9.8725e-04	3.2624e+02	1.0202e-03	2.9057e+02
7.7075e-04	3.6823e+02	8.4041e-04	3.7212e+02	8.2373e-04	3.7301e+02	8.5124e-04	3.2444e+02
6.1775e-04	4.8869e+02	6.7358e-04	4.4575e+02	6.6022e-04	4.3833e+02	6.8226e-04	4.5203e+02
4.6475e-04	5.6705e+02	5.0676e-04	5.5498e+02	4.9670e-04	5.2589e+02	5.1329e-04	5.0655e+02
3.1175e-04	7.1465e+02	3.3993e-04	6.7556e+02	3.3318e-04	6.6828e+02	3.4431e-04	6.6880e+02
1.5875e-04	8.7330e+02	1.7310e-04	8.3676e+02	1.6966e-04	8.7619e+02	1.7533e-04	8.0120e+02
5.7519e-06	1.0918e+03	6.2717e-06	1.0408e+03	6.1473e-06	9.9878e+02	6.3526e-06	9.5645e+02

FREQUENCY [Hz]: 9014.3		9630.5		10267		11775	
TAU [s]	MAGNITUDES [a.u]	TAU [s]	MAGNITUDES [a.u]	TAU [s]	MAGNITUDES [a.u]	TAU [s]	MAGNITUDES [a.u]
2.4588e-03	1.8321e+02	2.7779e-03	1.9814e+02	2.6076e-03	1.9557e+02	2.9398e-03	1.8333e+02
2.2953e-03	1.9833e+02	2.5932e-03	2.0264e+02	2.4342e-03	1.9036e+02	2.7443e-03	1.9682e+02
2.1318e-03	1.7288e+02	2.4085e-03	2.2218e+02	2.2608e-03	1.9818e+02	2.5488e-03	1.9572e+02
1.9683e-03	1.8533e+02	2.2237e-03	2.0724e+02	2.0874e-03	1.9930e+02	2.3533e-03	1.9708e+02
1.8048e-03	2.0008e+02	2.0390e-03	2.1936e+02	1.9140e-03	1.9356e+02	2.1578e-03	2.1098e+02
1.6413e-03	2.1900e+02	1.8543e-03	2.2773e+02	1.7406e-03	2.4733e+02	1.9623e-03	2.2127e+02
1.4778e-03	2.4316e+02	1.6695e-03	2.2535e+02	1.5672e-03	2.4709e+02	1.7668e-03	2.0525e+02
1.3143e-03	2.3768e+02	1.4848e-03	2.3847e+02	1.3938e-03	2.5224e+02	1.5713e-03	2.4850e+02
1.1507e-03	2.9806e+02	1.3001e-03	2.5996e+02	1.2204e-03	2.8249e+02	1.3758e-03	2.5849e+02
9.8723e-04	3.3597e+02	1.1153e-03	3.2993e+02	1.0470e-03	3.2635e+02	1.1803e-03	3.2754e+02
8.2371e-04	4.1137e+02	9.3061e-04	3.6696e+02	8.7354e-04	3.8460e+02	9.8484e-04	3.8015e+02
6.6020e-04	4.5176e+02	7.4588e-04	4.1820e+02	7.0014e-04	4.4579e+02	7.8934e-04	4.4738e+02
4.9669e-04	5.3226e+02	5.6114e-04	5.2357e+02	5.2673e-04	5.5439e+02	5.9384e-04	5.4660e+02
3.3317e-04	6.6091e+02	3.7641e-04	6.5647e+02	3.5333e-04	6.6310e+02	3.9835e-04	6.7643e+02
1.6966e-04	7.9059e+02	1.9168e-04	8.0796e+02	1.7992e-04	8.2497e+02	2.0285e-04	8.2756e+02
6.1471e-06	9.6908e+02	6.9448e-06	9.5775e+02	6.5190e-06	9.6117e+02	7.3496e-06	1.0136e+03

FREQUENCY [Hz]: 12656		13384		14324		15294	
TAU [s]	MAGNITUDES [a.u]	TAU [s]	MAGNITUDES [a.u]	TAU [s]	MAGNITUDES [a.u]	TAU [s]	MAGNITUDES [a.u]
2.9044e-03	1.9749e+02	2.8775e-03	1.8819e+02	3.2877e-03	2.2378e+02	3.0468e-03	2.0695e+02
2.7112e-03	1.9833e+02	2.6862e-03	2.1931e+02	3.0691e-03	1.8633e+02	2.8442e-03	1.9566e+02
2.5181e-03	1.6692e+02	2.4948e-03	1.9615e+02	2.8504e-03	1.6793e+02	2.6416e-03	2.1379e+02
2.3249e-03	1.9252e+02	2.3035e-03	2.1001e+02	2.6318e-03	1.7522e+02	2.4390e-03	1.8440e+02
2.1318e-03	1.8369e+02	2.1121e-03	2.0452e+02	2.4132e-03	1.8411e+02	2.2364e-03	1.8116e+02
1.9387e-03	1.8896e+02	1.9208e-03	2.2191e+02	2.1945e-03	2.0299e+02	2.0337e-03	2.1153e+02
1.7455e-03	2.2651e+02	1.7294e-03	2.6194e+02	1.9759e-03	2.1695e+02	1.8311e-03	2.5400e+02
1.5524e-03	2.2550e+02	1.5380e-03	2.4923e+02	1.7573e-03	2.3085e+02	1.6285e-03	2.5627e+02
1.3592e-03	2.5644e+02	1.3467e-03	2.8342e+02	1.5386e-03	2.4790e+02	1.4259e-03	2.8046e+02
1.1661e-03	3.5491e+02	1.1553e-03	2.9692e+02	1.3200e-03	2.8628e+02	1.2233e-03	3.7945e+02
9.7296e-04	3.9268e+02	9.6398e-04	3.9116e+02	1.1014e-03	3.2989e+02	1.0207e-03	4.0313e+02
7.7982e-04	4.9337e+02	7.7262e-04	4.7523e+02	8.8275e-04	4.3539e+02	8.1807e-04	4.6976e+02
5.8668e-04	5.9473e+02	5.8126e-04	6.0840e+02	6.6411e-04	5.3764e+02	6.1545e-04	6.0330e+02
3.9354e-04	7.2963e+02	3.8991e-04	6.9614e+02	4.4548e-04	6.8642e+02	4.1284e-04	7.2713e+02
2.0040e-04	9.2440e+02	1.9855e-04	8.5176e+02	2.2685e-04	8.2161e+02	2.1023e-04	8.9927e+02
7.2609e-06	1.0538e+03	7.1939e-06	1.0211e+03	8.2192e-06	1.0462e+03	7.6170e-06	1.0390e+03

FREQUENCY [Hz]: 16297		17540		18614		19944	
TAU [s]	MAGNITUDES [a.u]	TAU [s]	MAGNITUDES [a.u]	TAU [s]	MAGNITUDES [a.u]	TAU [s]	MAGNITUDES [a.u]
3.4460e-03	2.0712e+02	3.5835e-03	1.7262e+02	3.4204e-03	1.8037e+02	3.6978e-03	2.0245e+02
3.2169e-03	1.8831e+02	3.3452e-03	1.8520e+02	3.1929e-03	1.7502e+02	3.4519e-03	1.9358e+02
2.9877e-03	1.7235e+02	3.1069e-03	1.9090e+02	2.9655e-03	2.1718e+02	3.2060e-03	2.2545e+02
2.7585e-03	1.9138e+02	2.8686e-03	2.2169e+02	2.7380e-03	2.0029e+02	2.9601e-03	2.0470e+02
2.5294e-03	2.0670e+02	2.6303e-03	1.9985e+02	2.5106e-03	2.3021e+02	2.7142e-03	2.0327e+02
2.3002e-03	2.1572e+02	2.3920e-03	1.9343e+02	2.2831e-03	2.3447e+02	2.4683e-03	2.0749e+02
2.0711e-03	2.1796e+02	2.1537e-03	2.1080e+02	2.0557e-03	2.1933e+02	2.2224e-03	2.4667e+02
1.8419e-03	2.3885e+02	1.9154e-03	2.4750e+02	1.8282e-03	2.7397e+02	1.9765e-03	2.8439e+02
1.6127e-03	2.7296e+02	1.6771e-03	2.6843e+02	1.6007e-03	2.8655e+02	1.7306e-03	2.7462e+02
1.3836e-03	3.1020e+02	1.4388e-03	2.9000e+02	1.3733e-03	3.3952e+02	1.4847e-03	3.3092e+02
1.1544e-03	3.5223e+02	1.2005e-03	3.9674e+02	1.1458e-03	4.2249e+02	1.2388e-03	3.7931e+02
9.2525e-04	4.2065e+02	9.6218e-04	4.8131e+02	9.1838e-04	5.2443e+02	9.9286e-04	4.5044e+02
6.9609e-04	5.6239e+02	7.2387e-04	5.9350e+02	6.9092e-04	6.4662e+02	7.4695e-04	5.8567e+02
4.6693e-04	6.7118e+02	4.8557e-04	7.3860e+02	4.6346e-04	7.4850e+02	5.0105e-04	7.0042e+02
2.3777e-04	7.8092e+02	2.4726e-04	8.7679e+02	2.3601e-04	9.9201e+02	2.5515e-04	9.4227e+02
8.6150e-06	9.9823e+02	8.9588e-06	1.1294e+03	8.5510e-06	1.2007e+03	9.2445e-06	1.0959e+03

FREQUENCY [Hz]: 21319		22739		24453		25976	
TAU [s]	MAGNITUDES [a.u]	TAU [s]	MAGNITUDES [a.u]	TAU [s]	MAGNITUDES [a.u]	TAU [s]	MAGNITUDES [a.u]
3.8291e-03	1.5395e+02	3.8607e-03	1.9843e+02	4.1648e-03	1.9969e+02	4.2895e-03	2.0072e+02
3.5744e-03	1.7528e+02	3.6040e-03	1.8193e+02	3.8878e-03	1.5467e+02	4.0042e-03	1.8692e+02
3.3198e-03	1.8967e+02	3.3472e-03	1.9598e+02	3.6109e-03	2.2520e+02	3.7190e-03	2.1437e+02
3.0652e-03	2.2507e+02	3.0905e-03	1.9132e+02	3.3339e-03	2.1336e+02	3.4337e-03	2.2173e+02
2.8105e-03	1.9994e+02	2.8338e-03	2.0745e+02	3.0570e-03	1.6715e+02	3.1485e-03	2.0295e+02
2.5559e-03	1.9693e+02	2.5770e-03	1.7950e+02	2.7800e-03	2.2264e+02	2.8632e-03	2.0057e+02
2.3013e-03	2.2009e+02	2.3203e-03	2.4213e+02	2.5031e-03	2.1627e+02	2.5780e-03	2.2283e+02
2.0466e-03	2.3803e+02	2.0636e-03	2.4102e+02	2.2261e-03	2.4920e+02	2.2927e-03	2.3800e+02
1.7920e-03	2.8581e+02	1.8068e-03	2.8878e+02	1.9491e-03	2.6740e+02	2.0075e-03	2.9968e+02
1.5374e-03	3.4437e+02	1.5501e-03	3.5711e+02	1.6722e-03	3.0437e+02	1.7222e-03	3.4361e+02
1.2827e-03	3.7810e+02	1.2933e-03	4.2869e+02	1.3952e-03	3.9132e+02	1.4370e-03	4.1973e+02
1.0281e-03	4.9202e+02	1.0366e-03	4.7519e+02	1.1183e-03	4.5696e+02	1.1517e-03	4.9643e+02
7.7347e-04	6.3381e+02	7.7986e-04	6.5493e+02	8.4129e-04	5.6224e+02	8.6647e-04	6.1819e+02
5.1884e-04	7.8972e+02	5.2313e-04	7.6055e+02	5.6433e-04	6.9462e+02	5.8122e-04	7.9749e+02
2.6421e-04	9.8485e+02	2.6639e-04	9.4648e+02	2.8737e-04	8.9171e+02	2.9597e-04	9.6375e+02
9.5727e-06	1.1859e+03	9.6518e-06	1.1561e+03	1.0412e-05	1.0917e+03	1.0724e-05	1.1932e+03

FREQUENCY [Hz]: 27807		29699		31657		33969	
TAU [s]	MAGNITUDES [a.u]	TAU [s]	MAGNITUDES [a.u]	TAU [s]	MAGNITUDES [a.u]	TAU [s]	MAGNITUDES [a.u]
4.2540e-03	1.9732e+02	4.6290e-03	1.7885e+02	4.6385e-03	2.0723e+02	4.8985e-03	1.8778e+02
3.9712e-03	1.8383e+02	4.3212e-03	2.1639e+02	4.3300e-03	1.6981e+02	4.5728e-03	1.9856e+02
3.6883e-03	2.1588e+02	4.0133e-03	1.7055e+02	4.0216e-03	1.8849e+02	4.2470e-03	2.0333e+02
3.4054e-03	1.9769e+02	3.7055e-03	1.8854e+02	3.7131e-03	2.0616e+02	3.9213e-03	2.0700e+02
3.1225e-03	2.0722e+02	3.3977e-03	2.0479e+02	3.4047e-03	2.4162e+02	3.5955e-03	1.9874e+02
2.8396e-03	2.2592e+02	3.0899e-03	2.0969e+02	3.0962e-03	2.2128e+02	3.2698e-03	2.1429e+02
2.5567e-03	2.6744e+02	2.7820e-03	2.2366e+02	2.7877e-03	2.3235e+02	2.9440e-03	2.4309e+02
2.2738e-03	2.9004e+02	2.4742e-03	2.2823e+02	2.4793e-03	2.8657e+02	2.6183e-03	2.6205e+02
1.9909e-03	3.0971e+02	2.1664e-03	3.0040e+02	2.1708e-03	3.1636e+02	2.2925e-03	3.3517e+02
1.7080e-03	3.7251e+02	1.8585e-03	3.2828e+02	1.8624e-03	3.7099e+02	1.9668e-03	3.5556e+02
1.4251e-03	4.3252e+02	1.5507e-03	3.7243e+02	1.5539e-03	4.4179e+02	1.6410e-03	4.1848e+02
1.1422e-03	5.1616e+02	1.2429e-03	4.8433e+02	1.2454e-03	5.4672e+02	1.3153e-03	5.1005e+02
8.5932e-04	6.5612e+02	9.3506e-04	6.2695e+02	9.3698e-04	6.9457e+02	9.8950e-04	6.3391e+02
5.7642e-04	8.4410e+02	6.2723e-04	7.0847e+02	6.2852e-04	8.2151e+02	6.6375e-04	8.0991e+02
2.9353e-04	1.0567e+03	3.1940e-04	9.4567e+02	3.2006e-04	1.0563e+03	3.3800e-04	1.0319e+03
1.0635e-05	1.2680e+03	1.1572e-05	1.1810e+03	1.1596e-05	1.2988e+03	1.2246e-05	1.2899e+03

FREQUENCY [Hz]: 36060		38525		41395		44033	
TAU [s]	MAGNITUDES [a.u]	TAU [s]	MAGNITUDES [a.u]	TAU [s]	MAGNITUDES [a.u]	TAU [s]	MAGNITUDES [a.u]
4.8214e-03	2.2559e+02	5.4776e-03	1.5939e+02	5.5338e-03	2.0309e+02	5.8046e-03	2.0457e+02
4.5008e-03	2.0105e+02	5.1133e-03	1.9745e+02	5.1658e-03	1.8679e+02	5.4186e-03	2.1967e+02
4.1801e-03	2.0574e+02	4.7491e-03	1.9481e+02	4.7978e-03	2.1401e+02	5.0326e-03	2.2117e+02
3.8595e-03	2.0332e+02	4.3848e-03	2.2849e+02	4.4298e-03	2.2134e+02	4.6466e-03	2.1819e+02
3.5389e-03	2.1355e+02	4.0205e-03	2.0768e+02	4.0618e-03	2.1733e+02	4.2606e-03	2.1015e+02
3.2183e-03	2.1362e+02	3.6563e-03	2.2928e+02	3.6938e-03	2.4950e+02	3.8746e-03	2.1928e+02
2.8977e-03	2.4971e+02	3.2920e-03	2.5072e+02	3.3258e-03	2.4990e+02	3.4886e-03	2.5806e+02
2.5770e-03	2.7904e+02	2.9278e-03	2.6531e+02	2.9578e-03	2.5899e+02	3.1026e-03	2.9147e+02
2.2564e-03	3.1757e+02	2.5635e-03	2.8406e+02	2.5898e-03	3.1314e+02	2.7166e-03	3.2936e+02
1.9358e-03	3.8582e+02	2.1992e-03	3.3293e+02	2.2218e-03	3.7440e+02	2.3306e-03	3.4767e+02
1.6152e-03	4.0857e+02	1.8350e-03	4.0086e+02	1.8538e-03	4.3040e+02	1.9445e-03	4.3692e+02
1.2945e-03	5.3939e+02	1.4707e-03	5.0759e+02	1.4858e-03	5.4478e+02	1.5585e-03	5.8078e+02
9.7392e-04	6.4934e+02	1.1065e-03	6.4066e+02	1.1178e-03	6.5855e+02	1.1725e-03	7.1296e+02
6.5330e-04	7.9924e+02	7.4221e-04	8.0952e+02	7.4982e-04	8.6872e+02	7.8653e-04	8.7890e+02
3.3268e-04	1.0396e+03	3.7795e-04	1.0610e+03	3.8183e-04	1.0214e+03	4.0052e-04	1.1190e+03
1.2053e-05	1.2317e+03	1.3694e-05	1.3069e+03	1.3834e-05	1.3019e+03	1.4512e-05	1.3404e+03

FREQUENCY [Hz]: 47096		50266		53908		57294	
TAU [s]	MAGNITUDES [a.u]	TAU [s]	MAGNITUDES [a.u]	TAU [s]	MAGNITUDES [a.u]	TAU [s]	MAGNITUDES [a.u]
6.0794e-03	1.9839e+02	6.4941e-03	2.0537e+02	6.0224e-03	1.8920e+02	6.8188e-03	1.8565e+02
5.6751e-03	2.0197e+02	6.0622e-03	2.0939e+02	5.6219e-03	1.9002e+02	6.3654e-03	2.1811e+02
5.2708e-03	2.1446e+02	5.6304e-03	2.2962e+02	5.2214e-03	2.2282e+02	5.9119e-03	2.0002e+02
4.8666e-03	1.8863e+02	5.1985e-03	2.2603e+02	4.8209e-03	2.2038e+02	5.4585e-03	2.1485e+02
4.4623e-03	2.1079e+02	4.7667e-03	2.1226e+02	4.4204e-03	2.3971e+02	5.0050e-03	2.3928e+02
4.0580e-03	2.1432e+02	4.3348e-03	2.5838e+02	4.0199e-03	2.6949e+02	4.5516e-03	2.3650e+02
3.6537e-03	2.7834e+02	3.9029e-03	2.5636e+02	3.6195e-03	2.4915e+02	4.0981e-03	2.5621e+02
3.2494e-03	2.8056e+02	3.4711e-03	2.5841e+02	3.2190e-03	3.4011e+02	3.6447e-03	2.7430e+02
2.8452e-03	3.0554e+02	3.0392e-03	2.9195e+02	2.8185e-03	3.5584e+02	3.1912e-03	3.0913e+02
2.4409e-03	3.6607e+02	2.6074e-03	3.6113e+02	2.4180e-03	4.1148e+02	2.7378e-03	3.6659e+02
2.0366e-03	4.5028e+02	2.1755e-03	4.3993e+02	2.0175e-03	5.0789e+02	2.2843e-03	4.2060e+02
1.6323e-03	5.3595e+02	1.7437e-03	5.7212e+02	1.6170e-03	5.8635e+02	1.8309e-03	4.8571e+02
1.2280e-03	6.4809e+02	1.3118e-03	6.8056e+02	1.2165e-03	7.4075e+02	1.3774e-03	6.4435e+02
8.2376e-04	7.9303e+02	8.7995e-04	8.6335e+02	8.1603e-04	9.1886e+02	9.2395e-04	7.7470e+02
4.1948e-04	1.0043e+03	4.4809e-04	1.0948e+03	4.1554e-04	1.1214e+03	4.7050e-04	1.0675e+03
1.5198e-05	1.3153e+03	1.6235e-05	1.3565e+03	1.5056e-05	1.3907e+03	1.7047e-05	1.2932e+03

FREQUENCY [Hz]: 61178		65596		69746		74895	
TAU [s]	MAGNITUDES [a.u]	TAU [s]	MAGNITUDES [a.u]	TAU [s]	MAGNITUDES [a.u]	TAU [s]	MAGNITUDES [a.u]
6.9599e-03	2.0903e+02	7.3042e-03	1.9848e+02	7.6719e-03	2.0563e+02	7.9413e-03	2.1983e+02
6.4971e-03	2.0607e+02	6.8185e-03	2.0370e+02	7.1617e-03	2.2001e+02	7.4132e-03	2.0960e+02
6.0343e-03	2.3211e+02	6.3328e-03	2.0169e+02	6.6515e-03	1.8971e+02	6.8851e-03	2.3102e+02
5.5714e-03	2.1344e+02	5.8470e-03	2.0814e+02	6.1413e-03	2.2396e+02	6.3570e-03	2.2388e+02
5.1086e-03	2.2603e+02	5.3613e-03	2.5394e+02	5.6311e-03	1.9573e+02	5.8289e-03	2.5276e+02
4.6458e-03	2.4932e+02	4.8756e-03	2.6573e+02	5.1210e-03	2.5086e+02	5.3008e-03	2.6891e+02
4.1829e-03	2.9258e+02	4.3898e-03	2.6936e+02	4.6108e-03	2.9520e+02	4.7727e-03	3.0137e+02
3.7201e-03	3.1128e+02	3.9041e-03	2.7406e+02	4.1006e-03	2.7039e+02	4.2446e-03	3.2020e+02
3.2572e-03	3.3029e+02	3.4184e-03	3.4768e+02	3.5904e-03	3.3778e+02	3.7165e-03	3.5408e+02
2.7944e-03	4.2592e+02	2.9326e-03	3.7752e+02	3.0803e-03	3.9224e+02	3.1884e-03	3.9979e+02
2.3316e-03	5.2665e+02	2.4469e-03	4.7711e+02	2.5701e-03	4.5089e+02	2.6603e-03	4.7755e+02
1.8687e-03	5.6051e+02	1.9612e-03	5.6171e+02	2.0599e-03	6.3885e+02	2.1322e-03	6.0189e+02
1.4059e-03	7.8350e+02	1.4755e-03	7.3127e+02	1.5497e-03	7.4141e+02	1.6041e-03	7.7725e+02
9.4307e-04	9.6534e+02	9.8972e-04	9.1739e+02	1.0395e-03	9.0908e+02	1.0760e-03	9.8330e+02
4.8023e-04	1.2049e+03	5.0399e-04	1.1586e+03	5.2936e-04	1.1578e+03	5.4795e-04	1.1840e+03
1.7400e-05	1.4767e+03	1.8261e-05	1.4598e+03	1.9180e-05	1.5142e+03	1.9853e-05	1.4944e+03

FREQUENCY [Hz]: 79777		85274		90960		97324	
TAU [s]	MAGNITUDES [a.u]	TAU [s]	MAGNITUDES [a.u]	TAU [s]	MAGNITUDES [a.u]	TAU [s]	MAGNITUDES [a.u]
7.9779e-03	2.1203e+02	8.5247e-03	1.8395e+02	8.9861e-03	1.8835e+02	9.2485e-03	1.9546e+02
7.4474e-03	1.8480e+02	7.9578e-03	2.1264e+02	8.3885e-03	2.0329e+02	8.6335e-03	2.1931e+02
6.9169e-03	2.1174e+02	7.3909e-03	2.1117e+02	7.7909e-03	2.3222e+02	8.0185e-03	2.3257e+02
6.3863e-03	2.1637e+02	6.8241e-03	2.2392e+02	7.1933e-03	2.2150e+02	7.4034e-03	2.2345e+02
5.8558e-03	2.8378e+02	6.2572e-03	2.2796e+02	6.5958e-03	2.5683e+02	6.7884e-03	2.3273e+02
5.3253e-03	2.6200e+02	5.6903e-03	2.8145e+02	5.9982e-03	2.9427e+02	6.1734e-03	2.4903e+02
4.7947e-03	3.0681e+02	5.1234e-03	2.7653e+02	5.4006e-03	2.7151e+02	5.5584e-03	2.5898e+02
4.2642e-03	3.4422e+02	4.5565e-03	3.4028e+02	4.8031e-03	3.3545e+02	4.9433e-03	3.2140e+02
3.7337e-03	3.8181e+02	3.9896e-03	3.7122e+02	4.2055e-03	3.6728e+02	4.3283e-03	3.3157e+02
3.2031e-03	4.6131e+02	3.4227e-03	4.1742e+02	3.6079e-03	4.3688e+02	3.7133e-03	4.0362e+02
2.6726e-03	5.2999e+02	2.8558e-03	5.3609e+02	3.0103e-03	5.0331e+02	3.0983e-03	5.1924e+02
2.1421e-03	6.0146e+02	2.2889e-03	6.1810e+02	2.4128e-03	6.1119e+02	2.4832e-03	6.3536e+02
1.6115e-03	7.7439e+02	1.7220e-03	7.6430e+02	1.8152e-03	7.6749e+02	1.8682e-03	7.4194e+02
1.0810e-03	9.7590e+02	1.1551e-03	9.7829e+02	1.2176e-03	9.6943e+02	1.2532e-03	9.3122e+02
5.5048e-04	1.2223e+03	5.8821e-04	1.2492e+03	6.2004e-04	1.2375e+03	6.3815e-04	1.2187e+03
1.9945e-05	1.5287e+03	2.1312e-05	1.5761e+03	2.2465e-05	1.5757e+03	2.3121e-05	1.4764e+03

FREQUENCY [Hz]: 103900		111230		118800		126630	
TAU [s]	MAGNITUDES [a.u]	TAU [s]	MAGNITUDES [a.u]	TAU [s]	MAGNITUDES [a.u]	TAU [s]	MAGNITUDES [a.u]
9.5082e-03	2.1742e+02	9.6939e-03	2.2448e+02	1.0172e-02	1.6019e+02	1.0492e-02	2.3325e+02
8.8759e-03	2.2290e+02	9.0493e-03	2.3207e+02	9.4952e-03	2.2614e+02	9.7943e-03	1.9838e+02
8.2436e-03	2.2549e+02	8.4046e-03	2.4693e+02	8.8188e-03	2.2726e+02	9.0965e-03	2.2591e+02
7.6113e-03	2.5843e+02	7.7600e-03	2.2033e+02	8.1424e-03	2.4308e+02	8.3988e-03	2.5085e+02
6.9790e-03	2.5536e+02	7.1153e-03	2.5874e+02	7.4660e-03	2.5259e+02	7.7011e-03	2.5334e+02
6.3467e-03	2.6995e+02	6.4707e-03	2.5258e+02	6.7896e-03	2.1913e+02	7.0034e-03	3.0567e+02
5.7144e-03	2.7749e+02	5.8260e-03	2.8884e+02	6.1131e-03	2.5273e+02	6.3057e-03	3.0994e+02
5.0821e-03	2.9015e+02	5.1814e-03	3.2803e+02	5.4367e-03	3.3527e+02	5.6080e-03	3.2432e+02
4.4498e-03	3.4985e+02	4.5367e-03	3.7326e+02	4.7603e-03	3.8704e+02	4.9102e-03	4.0145e+02
3.8175e-03	4.1508e+02	3.8921e-03	4.5471e+02	4.0839e-03	4.2106e+02	4.2125e-03	4.6568e+02
3.1852e-03	5.1056e+02	3.2475e-03	5.4266e+02	3.4075e-03	5.4303e+02	3.5148e-03	5.5463e+02
2.5529e-03	6.0893e+02	2.6028e-03	6.5426e+02	2.7311e-03	6.4968e+02	2.8171e-03	6.4939e+02
1.9206e-03	7.6086e+02	1.9582e-03	7.8397e+02	2.0547e-03	7.9740e+02	2.1194e-03	8.1219e+02
1.2884e-03	9.5540e+02	1.3135e-03	1.0660e+03	1.3783e-03	9.7495e+02	1.4217e-03	1.0473e+03
6.5606e-04	1.1710e+03	6.6888e-04	1.3346e+03	7.0184e-04	1.2894e+03	7.2395e-04	1.3327e+03
2.3770e-05	1.4993e+03	2.4235e-05	1.6142e+03	2.5429e-05	1.6362e+03	2.6230e-05	1.6412e+03

FREQUENCY [Hz]: 134700		142430		150980		160430	
TAU [s]	MAGNITUDES [a.u]	TAU [s]	MAGNITUDES [a.u]	TAU [s]	MAGNITUDES [a.u]	TAU [s]	MAGNITUDES [a.u]
1.1076e-02	2.3199e+02	1.1407e-02	1.8425e+02	1.1856e-02	2.1801e+02	1.2642e-02	2.2777e+02
1.0719e-02	2.2593e+02	1.1040e-02	2.1477e+02	1.1475e-02	2.1512e+02	1.2235e-02	2.0710e+02
1.0363e-02	2.2947e+02	1.0673e-02	2.2691e+02	1.1093e-02	2.0597e+02	1.1828e-02	2.5059e+02
1.0007e-02	2.3396e+02	1.0306e-02	2.3368e+02	1.0712e-02	2.2895e+02	1.1422e-02	2.2909e+02
9.6502e-03	2.2991e+02	9.9387e-03	2.2362e+02	1.0330e-02	2.4189e+02	1.1015e-02	2.1636e+02
9.2938e-03	2.2427e+02	9.5717e-03	2.4327e+02	9.9488e-03	2.2133e+02	1.0608e-02	2.3500e+02
8.9374e-03	2.6587e+02	9.2046e-03	2.3052e+02	9.5673e-03	2.2089e+02	1.0201e-02	2.1863e+02
8.5810e-03	2.7735e+02	8.8376e-03	2.5968e+02	9.1858e-03	2.4108e+02	9.7945e-03	2.3267e+02
8.2246e-03	2.4929e+02	8.4705e-03	2.4321e+02	8.8042e-03	2.2979e+02	9.3878e-03	2.7456e+02
7.8682e-03	2.7322e+02	8.1035e-03	2.6107e+02	8.4227e-03	2.4989e+02	8.9810e-03	2.6780e+02
7.5119e-03	2.7360e+02	7.7365e-03	2.4986e+02	8.0412e-03	2.4224e+02	8.5742e-03	3.0187e+02
7.1555e-03	2.9060e+02	7.3694e-03	2.7994e+02	7.6597e-03	3.1974e+02	8.1674e-03	2.7690e+02
6.7991e-03	2.6554e+02	7.0024e-03	3.1797e+02	7.2782e-03	3.0719e+02	7.7606e-03	2.9929e+02
6.4427e-03	2.9893e+02	6.6353e-03	3.2929e+02	6.8967e-03	3.4800e+02	7.3538e-03	3.3063e+02
6.0863e-03	3.4488e+02	6.2683e-03	3.4988e+02	6.5152e-03	3.4185e+02	6.9470e-03	3.2423e+02
5.7299e-03	3.5793e+02	5.9012e-03	3.5699e+02	6.1337e-03	3.7475e+02	6.5402e-03	3.7040e+02
5.3735e-03	3.5724e+02	5.5342e-03	3.6143e+02	5.7522e-03	3.5643e+02	6.1334e-03	3.5751e+02
5.0171e-03	4.2971e+02	5.1671e-03	4.1306e+02	5.3707e-03	3.9626e+02	5.7267e-03	4.0508e+02
4.6607e-03	4.7608e+02	4.8001e-03	4.6659e+02	4.9892e-03	4.6261e+02	5.3199e-03	3.9708e+02
4.3044e-03	4.5553e+02	4.4331e-03	5.1596e+02	4.6077e-03	4.7816e+02	4.9131e-03	4.8139e+02
3.9480e-03	5.0206e+02	4.0660e-03	5.0873e+02	4.2262e-03	5.1342e+02	4.5063e-03	4.8651e+02
3.5916e-03	5.4827e+02	3.6990e-03	5.6719e+02	3.8447e-03	5.4648e+02	4.0995e-03	5.4565e+02
3.2352e-03	6.4444e+02	3.3319e-03	6.3133e+02	3.4632e-03	6.4701e+02	3.6927e-03	6.5122e+02
2.8788e-03	7.0146e+02	2.9649e-03	7.1698e+02	3.0817e-03	7.3798e+02	3.2859e-03	6.7974e+02
2.5224e-03	8.0524e+02	2.5978e-03	7.8797e+02	2.7002e-03	8.0622e+02	2.8791e-03	7.0714e+02
2.1660e-03	8.2668e+02	2.2308e-03	8.3944e+02	2.3187e-03	8.7430e+02	2.4723e-03	8.4328e+02
1.8096e-03	9.5518e+02	1.8637e-03	1.0017e+03	1.9372e-03	1.0253e+03	2.0656e-03	9.5806e+02
1.4532e-03	1.0692e+03	1.4967e-03	1.0383e+03	1.5557e-03	1.0741e+03	1.6588e-03	1.0733e+03
1.0969e-03	1.2136e+03	1.1297e-03	1.2267e+03	1.1742e-03	1.1859e+03	1.2520e-03	1.1398e+03
7.4047e-04	1.3132e+03	7.6261e-04	1.3534e+03	7.9265e-04	1.3664e+03	8.4518e-04	1.3240e+03
3.8408e-04	1.5308e+03	3.9556e-04	1.4924e+03	4.1115e-04	1.5811e+03	4.3839e-04	1.5252e+03
2.7689e-05	1.7331e+03	2.8517e-05	1.7009e+03	2.9641e-05	1.7593e+03	3.1605e-05	1.6804e+03

FREQUENCY [Hz]: 169500		179500		189780		201060	
TAU [s]	MAGNITUDES [a.u]	TAU [s]	MAGNITUDES [a.u]	TAU [s]	MAGNITUDES [a.u]	TAU [s]	MAGNITUDES [a.u]
1.2689e-02	2.3537e+02	1.3216e-02	2.0192e+02	1.3638e-02	2.3243e+02	1.4381e-02	2.4415e+02
1.2281e-02	2.1479e+02	1.2791e-02	2.2047e+02	1.3200e-02	2.0690e+02	1.3918e-02	2.2839e+02
1.1872e-02	2.2894e+02	1.2366e-02	2.4678e+02	1.2761e-02	2.3909e+02	1.3456e-02	2.4092e+02
1.1464e-02	2.1195e+02	1.1940e-02	2.1696e+02	1.2322e-02	2.2615e+02	1.2993e-02	2.1739e+02
1.1056e-02	2.2236e+02	1.1515e-02	2.3938e+02	1.1883e-02	2.4308e+02	1.2530e-02	2.1188e+02
1.0647e-02	2.1640e+02	1.1090e-02	2.6412e+02	1.1444e-02	2.2119e+02	1.2067e-02	2.2306e+02
1.0239e-02	2.3802e+02	1.0665e-02	2.5359e+02	1.1005e-02	2.3748e+02	1.1605e-02	2.5938e+02
9.8307e-03	2.2144e+02	1.0239e-02	2.6288e+02	1.0566e-02	2.5297e+02	1.1142e-02	2.7331e+02
9.4225e-03	2.7164e+02	9.8140e-03	2.2979e+02	1.0128e-02	2.7661e+02	1.0679e-02	2.4049e+02
9.0142e-03	2.7963e+02	9.3888e-03	2.7107e+02	9.6887e-03	2.6795e+02	1.0216e-02	2.5360e+02
8.6059e-03	2.8062e+02	8.9635e-03	2.9875e+02	9.2499e-03	2.8871e+02	9.7537e-03	2.5317e+02
8.1976e-03	2.9009e+02	8.5382e-03	2.6761e+02	8.8110e-03	3.1918e+02	9.2910e-03	2.8046e+02
7.7893e-03	2.6517e+02	8.1130e-03	2.9271e+02	8.3722e-03	3.1345e+02	8.8282e-03	2.8697e+02
7.3810e-03	3.3035e+02	7.6877e-03	3.4625e+02	7.9334e-03	3.2146e+02	8.3655e-03	3.1459e+02
6.9727e-03	3.3496e+02	7.2625e-03	3.6463e+02	7.4945e-03	3.7218e+02	7.9027e-03	3.3738e+02
6.5644e-03	3.6564e+02	6.8372e-03	3.6954e+02	7.0557e-03	4.0743e+02	7.4400e-03	3.4831e+02
6.1561e-03	3.7572e+02	6.4119e-03	4.0551e+02	6.6168e-03	3.9830e+02	6.9772e-03	3.6237e+02
5.7478e-03	4.3202e+02	5.9867e-03	4.0158e+02	6.1780e-03	4.4095e+02	6.5145e-03	4.4552e+02
5.3395e-03	4.8183e+02	5.5614e-03	4.9545e+02	5.7391e-03	4.9494e+02	6.0517e-03	4.1235e+02
4.9312e-03	5.1928e+02	5.1362e-03	5.1124e+02	5.3003e-03	4.9668e+02	5.5890e-03	4.7939e+02
4.5229e-03	5.4620e+02	4.7109e-03	5.2966e+02	4.8614e-03	5.6140e+02	5.1262e-03	5.0986e+02
4.1146e-03	5.8374e+02	4.2856e-03	5.9071e+02	4.4226e-03	5.9796e+02	4.6635e-03	5.7835e+02
3.7064e-03	6.6572e+02	3.8604e-03	6.6780e+02	3.9837e-03	6.6944e+02	4.2007e-03	6.3259e+02
3.2981e-03	7.2061e+02	3.4351e-03	7.1689e+02	3.5449e-03	7.7398e+02	3.7380e-03	6.8705e+02
2.8898e-03	8.1623e+02	3.0099e-03	8.3796e+02	3.1060e-03	8.4499e+02	3.2752e-03	8.3559e+02
2.4815e-03	9.3001e+02	2.5846e-03	8.8396e+02	2.6672e-03	9.3493e+02	2.8125e-03	8.9312e+02
2.0732e-03	1.0372e+03	2.1593e-03	9.9317e+02	2.2283e-03	1.0217e+03	2.3497e-03	9.4777e+02
1.6649e-03	1.1093e+03	1.7341e-03	1.1359e+03	1.7895e-03	1.1675e+03	1.8870e-03	1.1232e+03
1.2566e-03	1.2705e+03	1.3088e-03	1.3045e+03	1.3506e-03	1.2739e+03	1.4242e-03	1.2389e+03
8.4831e-04	1.4511e+03	8.8356e-04	1.4052e+03	9.1179e-04	1.4783e+03	9.6145e-04	1.3795e+03
4.4001e-04	1.6229e+03	4.5830e-04	1.5955e+03	4.7294e-04	1.6264e+03	4.9870e-04	1.5447e+03
3.1722e-05	1.7890e+03	3.3040e-05	1.7979e+03	3.4096e-05	1.8543e+03	3.5953e-05	1.7138e+03

FREQUENCY [Hz]: 212680		224610		237660		251070	
TAU [s]	MAGNITUDES [a.u]	TAU [s]	MAGNITUDES [a.u]	TAU [s]	MAGNITUDES [a.u]	TAU [s]	MAGNITUDES [a.u]
1.4812e-02	1.9625e+02	1.5645e-02	1.9205e+02	1.5595e-02	2.5413e+02	1.6576e-02	2.4090e+02
1.4335e-02	2.2843e+02	1.5142e-02	2.3080e+02	1.5094e-02	2.5859e+02	1.6042e-02	2.3568e+02
1.3859e-02	2.1455e+02	1.4639e-02	2.3025e+02	1.4592e-02	2.0509e+02	1.5509e-02	2.1033e+02
1.3382e-02	2.3004e+02	1.4135e-02	2.3322e+02	1.4090e-02	2.1380e+02	1.4976e-02	2.2571e+02
1.2906e-02	2.3058e+02	1.3632e-02	2.6203e+02	1.3588e-02	2.3777e+02	1.4442e-02	2.5230e+02
1.2429e-02	2.5635e+02	1.3128e-02	2.4933e+02	1.3086e-02	2.4641e+02	1.3909e-02	2.3866e+02
1.1952e-02	2.5294e+02	1.2625e-02	2.2167e+02	1.2584e-02	2.5797e+02	1.3376e-02	2.3959e+02
1.1476e-02	2.3600e+02	1.2121e-02	2.5303e+02	1.2083e-02	2.6642e+02	1.2842e-02	2.5718e+02
1.0999e-02	2.5498e+02	1.1618e-02	2.7248e+02	1.1581e-02	2.7536e+02	1.2309e-02	2.2518e+02
1.0523e-02	2.5875e+02	1.1115e-02	2.6678e+02	1.1079e-02	3.1513e+02	1.1776e-02	2.8181e+02
1.0046e-02	3.1627e+02	1.0611e-02	2.9613e+02	1.0577e-02	3.3103e+02	1.1242e-02	2.9491e+02
9.5693e-03	2.8892e+02	1.0108e-02	2.8322e+02	1.0075e-02	3.3549e+02	1.0709e-02	2.9636e+02
9.0927e-03	3.0494e+02	9.6043e-03	2.9704e+02	9.5735e-03	3.6079e+02	1.0175e-02	3.1742e+02
8.6161e-03	3.6185e+02	9.1008e-03	3.4896e+02	9.0717e-03	3.4813e+02	9.6420e-03	3.4779e+02
8.1395e-03	3.8054e+02	8.5974e-03	3.4502e+02	8.5699e-03	3.7261e+02	9.1087e-03	3.9987e+02
7.6629e-03	3.9402e+02	8.0940e-03	3.5456e+02	8.0681e-03	3.9769e+02	8.5753e-03	3.8230e+02
7.1862e-03	3.8318e+02	7.5905e-03	3.9498e+02	7.5663e-03	4.6279e+02	8.0419e-03	3.9622e+02
6.7096e-03	4.3245e+02	7.0871e-03	4.3901e+02	7.0644e-03	4.5736e+02	7.5086e-03	4.2222e+02
6.2330e-03	4.7399e+02	6.5837e-03	4.3876e+02	6.5626e-03	5.2116e+02	6.9752e-03	4.5489e+02
5.7564e-03	4.9930e+02	6.0803e-03	5.0405e+02	6.0608e-03	5.6295e+02	6.4418e-03	4.9453e+02
5.2798e-03	5.5268e+02	5.5768e-03	5.3219e+02	5.5590e-03	6.2213e+02	5.9085e-03	5.5487e+02
4.8032e-03	6.1365e+02	5.0734e-03	6.1267e+02	5.0572e-03	6.5136e+02	5.3751e-03	5.7829e+02
4.3266e-03	6.8904e+02	4.5700e-03	7.0227e+02	4.5554e-03	7.2372e+02	4.8417e-03	6.4658e+02
3.8499e-03	7.5436e+02	4.0665e-03	6.9715e+02	4.0535e-03	7.9234e+02	4.3084e-03	7.4365e+02
3.3733e-03	8.3614e+02	3.5631e-03	8.0831e+02	3.5517e-03	8.5613e+02	3.7750e-03	8.3659e+02
2.8967e-03	9.5964e+02	3.0597e-03	8.7583e+02	3.0499e-03	1.0062e+03	3.2416e-03	9.3302e+02
2.4201e-03	1.0397e+03	2.5563e-03	1.0332e+03	2.5481e-03	1.0931e+03	2.7083e-03	1.0401e+03
1.9435e-03	1.1981e+03	2.0528e-03	1.1952e+03	2.0463e-03	1.2356e+03	2.1749e-03	1.1714e+03
1.4669e-03	1.2829e+03	1.5494e-03	1.2617e+03	1.5444e-03	1.3325e+03	1.6415e-03	1.2628e+03
9.9026e-04	1.4651e+03	1.0460e-03	1.4840e+03	1.0426e-03	1.5074e+03	1.1082e-03	1.4587e+03
5.1364e-04	1.6562e+03	5.4254e-04	1.6393e+03	5.4081e-04	1.6936e+03	5.7481e-04	1.6431e+03
3.7030e-05	1.8481e+03	3.9113e-05	1.8373e+03	3.8988e-05	1.8923e+03	4.1440e-05	1.8696e+03

FREQUENCY [Hz]: 265660		280680		296970		313720	
TAU [s]	MAGNITUDES [a.u]	TAU [s]	MAGNITUDES [a.u]	TAU [s]	MAGNITUDES [a.u]	TAU [s]	MAGNITUDES [a.u]
1.6643e-02	2.1231e+02	1.7576e-02	2.3308e+02	1.7927e-02	2.2698e+02	1.8035e-02	2.4418e+02
1.6107e-02	2.3710e+02	1.7010e-02	2.2416e+02	1.7350e-02	2.5766e+02	1.7455e-02	2.5695e+02
1.5572e-02	2.3080e+02	1.6445e-02	2.4382e+02	1.6773e-02	2.2134e+02	1.6874e-02	2.2203e+02
1.5036e-02	2.4012e+02	1.5879e-02	2.4059e+02	1.6196e-02	2.4551e+02	1.6294e-02	2.4769e+02
1.4501e-02	2.5130e+02	1.5313e-02	2.2544e+02	1.5620e-02	2.5092e+02	1.5714e-02	2.5936e+02
1.3965e-02	2.3834e+02	1.4748e-02	2.7357e+02	1.5043e-02	2.6104e+02	1.5133e-02	2.4847e+02
1.3430e-02	2.1815e+02	1.4182e-02	2.8077e+02	1.4466e-02	2.5985e+02	1.4553e-02	2.6142e+02
1.2894e-02	2.7073e+02	1.3617e-02	2.8561e+02	1.3889e-02	2.5868e+02	1.3973e-02	2.6758e+02
1.2359e-02	2.4117e+02	1.3051e-02	2.7307e+02	1.3312e-02	3.2333e+02	1.3392e-02	3.0835e+02
1.1823e-02	3.0675e+02	1.2486e-02	2.9467e+02	1.2735e-02	3.0625e+02	1.2812e-02	3.2847e+02
1.1287e-02	3.1929e+02	1.1920e-02	2.8415e+02	1.2159e-02	3.0732e+02	1.2232e-02	3.2213e+02
1.0752e-02	3.1916e+02	1.1355e-02	3.2672e+02	1.1582e-02	3.1295e+02	1.1652e-02	3.1919e+02
1.0216e-02	3.3196e+02	1.0789e-02	3.2846e+02	1.1005e-02	3.2757e+02	1.1071e-02	3.4980e+02
9.6809e-03	3.3345e+02	1.0224e-02	3.6815e+02	1.0428e-02	3.9065e+02	1.0491e-02	3.9006e+02
9.1454e-03	3.1968e+02	9.6581e-03	3.8098e+02	9.8512e-03	3.8919e+02	9.9106e-03	4.1068e+02
8.6099e-03	3.5534e+02	9.0925e-03	3.8384e+02	9.2743e-03	3.7977e+02	9.3302e-03	4.0959e+02
8.0744e-03	4.2355e+02	8.5270e-03	3.9189e+02	8.6975e-03	4.5005e+02	8.7499e-03	4.5930e+02
7.5389e-03	4.5663e+02	7.9615e-03	4.4127e+02	8.1206e-03	4.7154e+02	8.1696e-03	5.0308e+02
7.0033e-03	4.6798e+02	7.3959e-03	4.8323e+02	7.5438e-03	5.0402e+02	7.5893e-03	4.7941e+02
6.4678e-03	5.5363e+02	6.8304e-03	5.2028e+02	6.9670e-03	5.5278e+02	7.0090e-03	5.7827e+02
5.9323e-03	5.8081e+02	6.2648e-03	5.5203e+02	6.3901e-03	6.0424e+02	6.4286e-03	6.2077e+02
5.3968e-03	6.1936e+02	5.6993e-03	6.7430e+02	5.8133e-03	6.5689e+02	5.8483e-03	6.7913e+02
4.8613e-03	6.8947e+02	5.1338e-03	6.9930e+02	5.2364e-03	7.0674e+02	5.2680e-03	7.4185e+02
4.3258e-03	7.8110e+02	4.5682e-03	7.9180e+02	4.6596e-03	7.8457e+02	4.6877e-03	8.4231e+02
3.7902e-03	8.6105e+02	4.0027e-03	9.0533e+02	4.0827e-03	9.2216e+02	4.1073e-03	8.7001e+02
3.2547e-03	9.4472e+02	3.4372e-03	9.3750e+02	3.5059e-03	1.0086e+03	3.5270e-03	1.0395e+03
2.7192e-03	1.0537e+03	2.8716e-03	1.0998e+03	2.9290e-03	1.0947e+03	2.9467e-03	1.1741e+03
2.1837e-03	1.2020e+03	2.3061e-03	1.2082e+03	2.3522e-03	1.2241e+03	2.3664e-03	1.2518e+03
1.6482e-03	1.3406e+03	1.7406e-03	1.3794e+03	1.7754e-03	1.3958e+03	1.7861e-03	1.4111e+03
1.1126e-03	1.4891e+03	1.1750e-03	1.5725e+03	1.1985e-03	1.5741e+03	1.2057e-03	1.6221e+03
5.7713e-04	1.6493e+03	6.0948e-04	1.7773e+03	6.2166e-04	1.7086e+03	6.2541e-04	1.7905e+03
4.1607e-05	1.8506e+03	4.3939e-05	1.9810e+03	4.4817e-05	1.9478e+03	4.5088e-05	1.9787e+03

FREQUENCY [Hz]: 331850		351440		371580		393290	
TAU [s]	MAGNITUDES [a.u]	TAU [s]	MAGNITUDES [a.u]	TAU [s]	MAGNITUDES [a.u]	TAU [s]	MAGNITUDES [a.u]
1.9243e-02	2.2720e+02	1.9562e-02	2.2330e+02	2.1337e-02	2.4653e+02	2.1282e-02	2.2615e+02
1.8624e-02	2.4936e+02	1.8933e-02	2.3612e+02	2.0650e-02	2.6282e+02	2.0597e-02	2.5106e+02
1.8005e-02	2.5653e+02	1.8303e-02	2.3818e+02	1.9964e-02	2.4051e+02	1.9912e-02	2.5704e+02
1.7386e-02	2.4591e+02	1.7674e-02	2.4031e+02	1.9277e-02	3.0688e+02	1.9227e-02	2.5859e+02
1.6766e-02	2.4503e+02	1.7044e-02	2.5407e+02	1.8591e-02	2.4355e+02	1.8542e-02	2.3901e+02
1.6147e-02	2.5651e+02	1.6415e-02	2.7042e+02	1.7904e-02	2.5929e+02	1.7858e-02	2.5161e+02
1.5528e-02	2.8404e+02	1.5785e-02	2.6171e+02	1.7217e-02	2.5784e+02	1.7173e-02	2.7045e+02
1.4909e-02	2.6974e+02	1.5156e-02	2.7921e+02	1.6531e-02	2.7833e+02	1.6488e-02	2.9455e+02
1.4290e-02	3.3217e+02	1.4526e-02	3.0197e+02	1.5844e-02	3.0361e+02	1.5803e-02	2.8996e+02
1.3670e-02	2.9712e+02	1.3897e-02	2.7862e+02	1.5158e-02	3.1148e+02	1.5118e-02	3.1998e+02
1.3051e-02	3.3883e+02	1.3267e-02	3.0352e+02	1.4471e-02	3.2261e+02	1.4434e-02	3.6462e+02
1.2432e-02	3.4785e+02	1.2638e-02	3.1764e+02	1.3785e-02	2.9129e+02	1.3749e-02	3.4490e+02
1.1813e-02	3.5704e+02	1.2009e-02	3.4153e+02	1.3098e-02	3.3630e+02	1.3064e-02	3.7269e+02
1.1194e-02	3.7669e+02	1.1379e-02	3.9389e+02	1.2411e-02	3.5727e+02	1.2379e-02	3.8387e+02
1.0574e-02	3.7918e+02	1.0750e-02	3.8908e+02	1.1725e-02	3.7939e+02	1.1695e-02	4.3687e+02
9.9552e-03	4.2505e+02	1.0120e-02	4.0935e+02	1.1038e-02	4.0285e+02	1.1010e-02	4.2055e+02
9.3360e-03	4.2268e+02	9.4907e-03	4.1738e+02	1.0352e-02	3.9551e+02	1.0325e-02	4.6065e+02
8.7168e-03	4.7481e+02	8.8613e-03	4.7851e+02	9.6652e-03	4.7863e+02	9.6402e-03	4.9367e+02
8.0976e-03	5.0539e+02	8.2318e-03	5.3716e+02	8.9787e-03	5.1161e+02	8.9554e-03	5.1576e+02
7.4784e-03	5.3136e+02	7.6024e-03	5.2417e+02	8.2921e-03	5.4458e+02	8.2706e-03	6.1038e+02
6.8592e-03	6.0930e+02	6.9729e-03	6.1602e+02	7.6055e-03	6.1251e+02	7.5858e-03	6.5789e+02
6.2401e-03	6.7167e+02	6.3435e-03	6.9344e+02	6.9190e-03	6.4010e+02	6.9011e-03	6.9426e+02
5.6209e-03	7.1835e+02	5.7140e-03	7.3677e+02	6.2324e-03	6.9890e+02	6.2163e-03	7.4353e+02
5.0017e-03	7.9907e+02	5.0845e-03	8.0574e+02	5.5459e-03	7.7069e+02	5.5315e-03	8.4425e+02
4.3825e-03	9.0232e+02	4.4551e-03	8.7707e+02	4.8593e-03	8.5671e+02	4.8467e-03	9.6789e+02
3.7633e-03	1.0278e+03	3.8256e-03	1.0232e+03	4.1727e-03	9.6098e+02	4.1619e-03	1.0854e+03
3.1441e-03	1.1222e+03	3.1962e-03	1.1168e+03	3.4862e-03	1.1095e+03	3.4771e-03	1.1820e+03
2.5249e-03	1.2639e+03	2.5667e-03	1.2598e+03	2.7996e-03	1.2030e+03	2.7923e-03	1.3169e+03
1.9057e-03	1.4145e+03	1.9373e-03	1.3867e+03	2.1130e-03	1.3905e+03	2.1076e-03	1.4590e+03
1.2865e-03	1.5714e+03	1.3078e-03	1.5631e+03	1.4265e-03	1.5652e+03	1.4228e-03	1.6328e+03
6.6730e-04	1.7681e+03	6.7836e-04	1.7499e+03	7.3991e-04	1.7730e+03	7.3799e-04	1.8223e+03
4.8108e-05	2.0520e+03	4.8905e-05	1.9620e+03	5.3342e-05	2.0209e+03	5.3204e-05	2.0570e+03

FREQUENCY [Hz]: 415610		439610		464290		491870	
TAU [s]	MAGNITUDES [a.u]	TAU [s]	MAGNITUDES [a.u]	TAU [s]	MAGNITUDES [a.u]	TAU [s]	MAGNITUDES [a.u]
2.2612e-02	2.6670e+02	2.2593e-02	2.2974e+02	2.3514e-02	2.3345e+02	2.3512e-02	2.2150e+02
2.1884e-02	2.5085e+02	2.1866e-02	2.3668e+02	2.2757e-02	2.5099e+02	2.2755e-02	2.6583e+02
2.1157e-02	2.4434e+02	2.1139e-02	2.4538e+02	2.2001e-02	2.8481e+02	2.1999e-02	2.6168e+02
2.0429e-02	2.7005e+02	2.0412e-02	2.4957e+02	2.1244e-02	2.8633e+02	2.1242e-02	2.6112e+02
1.9702e-02	2.7953e+02	1.9685e-02	2.4829e+02	2.0488e-02	2.8030e+02	2.0486e-02	2.9750e+02
1.8974e-02	2.4972e+02	1.8958e-02	2.5765e+02	1.9731e-02	2.6024e+02	1.9729e-02	2.5609e+02
1.8246e-02	2.6121e+02	1.8231e-02	2.9450e+02	1.8974e-02	2.6828e+02	1.8972e-02	2.9914e+02
1.7519e-02	3.0293e+02	1.7504e-02	2.9146e+02	1.8218e-02	2.7798e+02	1.8216e-02	3.0980e+02
1.6791e-02	2.9420e+02	1.6777e-02	2.9506e+02	1.7461e-02	2.9703e+02	1.7459e-02	3.2142e+02
1.6064e-02	3.0343e+02	1.6050e-02	3.3160e+02	1.6704e-02	3.1362e+02	1.6703e-02	3.3389e+02
1.5336e-02	3.2455e+02	1.5323e-02	3.4968e+02	1.5948e-02	3.1520e+02	1.5946e-02	3.4036e+02
1.4608e-02	3.4152e+02	1.4596e-02	3.5292e+02	1.5191e-02	3.5598e+02	1.5190e-02	3.6629e+02
1.3881e-02	3.3279e+02	1.3869e-02	3.6907e+02	1.4435e-02	3.4622e+02	1.4433e-02	3.7866e+02
1.3153e-02	3.8178e+02	1.3142e-02	3.7158e+02	1.3678e-02	3.7792e+02	1.3677e-02	4.2835e+02
1.2426e-02	3.9228e+02	1.2415e-02	4.1583e+02	1.2921e-02	4.0074e+02	1.2920e-02	4.1060e+02
1.1698e-02	3.9731e+02	1.1688e-02	4.6449e+02	1.2165e-02	4.1946e+02	1.2164e-02	4.7414e+02
1.0971e-02	4.4858e+02	1.0961e-02	4.8148e+02	1.1408e-02	4.6009e+02	1.1407e-02	5.1312e+02
1.0243e-02	4.7230e+02	1.0234e-02	5.0936e+02	1.0651e-02	4.6226e+02	1.0650e-02	5.2129e+02
9.5153e-03	5.5498e+02	9.5075e-03	5.3408e+02	9.8949e-03	5.2072e+02	9.8939e-03	5.5311e+02
8.7877e-03	5.9403e+02	8.7805e-03	5.8647e+02	9.1382e-03	5.8995e+02	9.1374e-03	5.8260e+02
8.0601e-03	6.4824e+02	8.0535e-03	6.7701e+02	8.3816e-03	6.7636e+02	8.3808e-03	6.3011e+02
7.3325e-03	6.8538e+02	7.3265e-03	6.9171e+02	7.6250e-03	6.6158e+02	7.6243e-03	6.9457e+02
6.6049e-03	7.5920e+02	6.5995e-03	7.4995e+02	6.8684e-03	7.8959e+02	6.8677e-03	7.9516e+02
5.8773e-03	8.4141e+02	5.8725e-03	8.4310e+02	6.1118e-03	8.4593e+02	6.1112e-03	8.8084e+02
5.1497e-03	9.3351e+02	5.1455e-03	9.4437e+02	5.3551e-03	9.2554e+02	5.3546e-03	9.6348e+02
4.4221e-03	1.0620e+03	4.4185e-03	1.0699e+03	4.5985e-03	1.0305e+03	4.5981e-03	1.0137e+03
3.6945e-03	1.1319e+03	3.6915e-03	1.1789e+03	3.8419e-03	1.1638e+03	3.8415e-03	1.1905e+03
2.9669e-03	1.2827e+03	2.9645e-03	1.3193e+03	3.0853e-03	1.3158e+03	3.0850e-03	1.3021e+03
2.2393e-03	1.4455e+03	2.2375e-03	1.4467e+03	2.3286e-03	1.5047e+03	2.3284e-03	1.4966e+03
1.5117e-03	1.6538e+03	1.5105e-03	1.6244e+03	1.5720e-03	1.6644e+03	1.5719e-03	1.6694e+03
7.8413e-04	1.8660e+03	7.8348e-04	1.8695e+03	8.1541e-04	1.8619e+03	8.1533e-04	1.9040e+03
5.6530e-05	2.1102e+03	5.6484e-05	2.0774e+03	5.8785e-05	2.1066e+03	5.8779e-05	2.0690e+03

FREQUENCY [Hz]: 520240		549410		581810		615140	
TAU [s]	MAGNITUDES [a.u]	TAU [s]	MAGNITUDES [a.u]	TAU [s]	MAGNITUDES [a.u]	TAU [s]	MAGNITUDES [a.u]
2.2615e-02	2.5098e+02	2.2934e-02	2.9881e+02	2.2830e-02	2.7484e+02	2.3576e-02	2.8102e+02
2.1888e-02	2.9179e+02	2.2196e-02	2.5615e+02	2.2095e-02	2.6536e+02	2.2818e-02	2.6241e+02
2.1160e-02	2.8771e+02	2.1458e-02	2.5409e+02	2.1361e-02	2.8382e+02	2.2059e-02	2.7583e+02
2.0432e-02	2.6030e+02	2.0720e-02	2.4846e+02	2.0626e-02	2.9070e+02	2.1300e-02	2.7269e+02
1.9705e-02	2.9546e+02	1.9982e-02	3.0558e+02	1.9891e-02	2.8817e+02	2.0542e-02	2.9017e+02
1.8977e-02	2.6186e+02	1.9244e-02	3.0810e+02	1.9157e-02	2.6989e+02	1.9783e-02	3.1034e+02
1.8249e-02	3.0023e+02	1.8506e-02	3.1351e+02	1.8422e-02	2.8254e+02	1.9024e-02	2.8990e+02
1.7521e-02	2.8988e+02	1.7768e-02	3.0281e+02	1.7688e-02	3.0771e+02	1.8266e-02	3.0332e+02
1.6794e-02	3.0283e+02	1.7030e-02	3.5246e+02	1.6953e-02	3.2034e+02	1.7507e-02	3.4325e+02
1.6066e-02	3.1871e+02	1.6292e-02	3.5587e+02	1.6218e-02	3.1138e+02	1.6749e-02	3.2513e+02
1.5338e-02	3.0185e+02	1.5554e-02	3.6549e+02	1.5484e-02	3.3124e+02	1.5990e-02	3.7113e+02
1.4611e-02	3.5666e+02	1.4816e-02	3.5767e+02	1.4749e-02	3.5236e+02	1.5231e-02	3.4763e+02
1.3883e-02	3.6889e+02	1.4078e-02	3.8590e+02	1.4015e-02	3.7799e+02	1.4473e-02	3.7684e+02
1.3155e-02	3.6771e+02	1.3340e-02	3.8310e+02	1.3280e-02	3.9854e+02	1.3714e-02	3.7623e+02
1.2428e-02	4.0721e+02	1.2603e-02	4.5729e+02	1.2545e-02	3.9758e+02	1.2955e-02	4.1224e+02
1.1700e-02	4.4974e+02	1.1865e-02	4.8968e+02	1.1811e-02	4.5344e+02	1.2197e-02	4.5541e+02
1.0972e-02	5.0297e+02	1.1127e-02	4.9879e+02	1.1076e-02	4.7211e+02	1.1438e-02	4.5586e+02
1.0244e-02	5.1120e+02	1.0389e-02	5.3774e+02	1.0342e-02	5.4334e+02	1.0680e-02	5.2668e+02
9.5167e-03	5.6187e+02	9.6507e-03	5.4194e+02	9.6069e-03	5.8701e+02	9.9210e-03	5.7999e+02
8.7890e-03	6.3998e+02	8.9128e-03	6.0407e+02	8.8723e-03	5.8072e+02	9.1624e-03	6.3621e+02
8.0613e-03	6.6523e+02	8.1748e-03	6.7409e+02	8.1377e-03	6.6060e+02	8.4038e-03	6.1850e+02
7.3336e-03	7.1159e+02	7.4369e-03	7.1706e+02	7.4031e-03	7.2050e+02	7.6451e-03	7.1550e+02
6.6059e-03	8.1883e+02	6.6989e-03	7.8142e+02	6.6685e-03	8.3169e+02	6.8865e-03	7.8398e+02
5.8782e-03	8.6107e+02	5.9610e-03	8.8586e+02	5.9339e-03	8.8204e+02	6.1279e-03	8.2473e+02
5.1505e-03	9.7389e+02	5.2230e-03	9.9814e+02	5.1993e-03	9.7429e+02	5.3693e-03	9.4444e+02
4.4228e-03	1.0881e+03	4.4850e-03	1.0852e+03	4.4647e-03	1.0895e+03	4.6107e-03	1.0497e+03
3.6951e-03	1.2051e+03	3.7471e-03	1.2095e+03	3.7301e-03	1.1839e+03	3.8520e-03	1.1797e+03
2.9674e-03	1.3317e+03	3.0091e-03	1.3232e+03	2.9955e-03	1.3442e+03	3.0934e-03	1.3451e+03
2.2397e-03	1.4672e+03	2.2712e-03	1.4829e+03	2.2609e-03	1.5223e+03	2.3348e-03	1.4578e+03
1.5119e-03	1.6871e+03	1.5332e-03	1.6497e+03	1.5263e-03	1.6375e+03	1.5762e-03	1.6393e+03
7.8424e-04	1.8592e+03	7.9529e-04	1.9225e+03	7.9168e-04	1.9049e+03	8.1756e-04	1.7765e+03
5.6538e-05	2.0317e+03	5.7335e-05	2.1300e+03	5.7075e-05	2.0818e+03	5.8940e-05	2.0565e+03

FREQUENCY [Hz]: 650690		687230		727490		768890	
TAU [s]	MAGNITUDES [a.u]	TAU [s]	MAGNITUDES [a.u]	TAU [s]	MAGNITUDES [a.u]	TAU [s]	MAGNITUDES [a.u]
2.4605e-02	2.2657e+02	2.7228e-02	2.3429e+02	2.8310e-02	3.0077e+02	3.0976e-02	2.8096e+02
2.3813e-02	2.6316e+02	2.6352e-02	2.4255e+02	2.7399e-02	2.8151e+02	2.9979e-02	2.7442e+02
2.3022e-02	2.8601e+02	2.5476e-02	2.4483e+02	2.6488e-02	2.6967e+02	2.8983e-02	2.7489e+02
2.2230e-02	2.7203e+02	2.4600e-02	2.8369e+02	2.5577e-02	2.7415e+02	2.7986e-02	2.8011e+02
2.1438e-02	2.9402e+02	2.3724e-02	2.8253e+02	2.4666e-02	2.8059e+02	2.6989e-02	2.8880e+02
2.0646e-02	3.1759e+02	2.2847e-02	2.9135e+02	2.3755e-02	3.1630e+02	2.5992e-02	3.1267e+02
1.9855e-02	2.8537e+02	2.1971e-02	3.0007e+02	2.2844e-02	2.9048e+02	2.4996e-02	2.9895e+02
1.9063e-02	3.3037e+02	2.1095e-02	3.1739e+02	2.1933e-02	3.2887e+02	2.3999e-02	3.1950e+02
1.8271e-02	3.2450e+02	2.0219e-02	3.1304e+02	2.1022e-02	3.3879e+02	2.3002e-02	3.5571e+02
1.7480e-02	3.3330e+02	1.9343e-02	3.3821e+02	2.0111e-02	3.3166e+02	2.2006e-02	3.0398e+02
1.6688e-02	3.6513e+02	1.8467e-02	3.3511e+02	1.9200e-02	3.7245e+02	2.1009e-02	3.3281e+02
1.5896e-02	3.5775e+02	1.7591e-02	3.7093e+02	1.8290e-02	3.4497e+02	2.0012e-02	3.4355e+02
1.5104e-02	3.7121e+02	1.6715e-02	3.5668e+02	1.7379e-02	3.9294e+02	1.9015e-02	3.6436e+02
1.4313e-02	4.2847e+02	1.5838e-02	4.0155e+02	1.6468e-02	3.9643e+02	1.8019e-02	3.6179e+02
1.3521e-02	4.2037e+02	1.4962e-02	4.1315e+02	1.5557e-02	4.4209e+02	1.7022e-02	4.2239e+02
1.2729e-02	4.4292e+02	1.4086e-02	4.2903e+02	1.4646e-02	4.3465e+02	1.6025e-02	4.2790e+02
1.1937e-02	4.9023e+02	1.3210e-02	4.5486e+02	1.3735e-02	4.5254e+02	1.5028e-02	5.1013e+02
1.1146e-02	5.3042e+02	1.2334e-02	4.9331e+02	1.2824e-02	5.5654e+02	1.4032e-02	5.1304e+02
1.0354e-02	6.0109e+02	1.1458e-02	5.3453e+02	1.1913e-02	5.7409e+02	1.3035e-02	5.6409e+02
9.5623e-03	6.0113e+02	1.0582e-02	5.8460e+02	1.1002e-02	6.2575e+02	1.2038e-02	6.2873e+02
8.7705e-03	6.5513e+02	9.7055e-03	6.4641e+02	1.0091e-02	6.2737e+02	1.1041e-02	6.8291e+02
7.9788e-03	6.9003e+02	8.8294e-03	6.7571e+02	9.1801e-03	7.4830e+02	1.0045e-02	7.3445e+02
7.1871e-03	7.8044e+02	7.9532e-03	7.8559e+02	8.2692e-03	8.0914e+02	9.0480e-03	7.8872e+02
6.3954e-03	8.7366e+02	7.0771e-03	8.5360e+02	7.3583e-03	9.2955e+02	8.0513e-03	8.8141e+02
5.6036e-03	9.3685e+02	6.2010e-03	9.2941e+02	6.4473e-03	9.6587e+02	7.0546e-03	9.5166e+02
4.8119e-03	1.0398e+03	5.3248e-03	1.0518e+03	5.5364e-03	1.0750e+03	6.0578e-03	1.0935e+03
4.0202e-03	1.1970e+03	4.4487e-03	1.1678e+03	4.6255e-03	1.2433e+03	5.0611e-03	1.2380e+03
3.2284e-03	1.3455e+03	3.5726e-03	1.3488e+03	3.7145e-03	1.4215e+03	4.0644e-03	1.3837e+03
2.4367e-03	1.5039e+03	2.6965e-03	1.4978e+03	2.8036e-03	1.5871e+03	3.0676e-03	1.5536e+03
1.6450e-03	1.6879e+03	1.8203e-03	1.7307e+03	1.8926e-03	1.7295e+03	2.0709e-03	1.7359e+03
8.5324e-04	1.8564e+03	9.4420e-04	1.9472e+03	9.8171e-04	1.9697e+03	1.0742e-03	1.9956e+03
6.1513e-05	2.1508e+03	6.8070e-05	2.1404e+03	7.0775e-05	2.1939e+03	7.7440e-05	2.2217e+03

FREQUENCY [Hz]: 812880		859570		909090		961550	
TAU [s]	MAGNITUDES [a.u]	TAU [s]	MAGNITUDES [a.u]	TAU [s]	MAGNITUDES [a.u]	TAU [s]	MAGNITUDES [a.u]
3.3660e-02	2.6090e+02	3.7464e-02	2.9634e+02	4.1746e-02	2.6438e+02	4.2502e-02	2.7640e+02
3.2576e-02	2.7517e+02	3.6258e-02	2.9655e+02	4.0403e-02	2.7462e+02	4.1134e-02	3.0484e+02
3.1493e-02	2.6448e+02	3.5053e-02	2.4800e+02	3.9060e-02	2.7246e+02	3.9767e-02	2.8471e+02
3.0410e-02	2.8846e+02	3.3847e-02	3.2181e+02	3.7716e-02	2.5823e+02	3.8399e-02	3.1690e+02
2.9327e-02	2.6562e+02	3.2642e-02	2.7765e+02	3.6373e-02	3.0144e+02	3.7031e-02	2.8871e+02
2.8244e-02	2.8084e+02	3.1436e-02	2.9793e+02	3.5030e-02	2.6531e+02	3.5664e-02	2.9126e+02
2.7161e-02	3.0325e+02	3.0231e-02	3.1367e+02	3.3686e-02	2.9024e+02	3.4296e-02	3.0866e+02
2.6078e-02	3.0614e+02	2.9025e-02	3.1169e+02	3.2343e-02	3.1015e+02	3.2929e-02	3.0739e+02
2.4995e-02	3.1273e+02	2.7820e-02	3.1985e+02	3.1000e-02	3.1329e+02	3.1561e-02	3.2671e+02
2.3912e-02	3.2302e+02	2.6614e-02	3.1385e+02	2.9657e-02	2.9786e+02	3.0193e-02	3.5412e+02
2.2829e-02	3.1468e+02	2.5409e-02	3.1681e+02	2.8313e-02	3.5153e+02	2.8826e-02	3.4834e+02
2.1746e-02	3.4078e+02	2.4203e-02	3.3631e+02	2.6970e-02	3.4200e+02	2.7458e-02	3.7754e+02
2.0663e-02	4.1317e+02	2.2998e-02	3.6482e+02	2.5627e-02	3.7558e+02	2.6091e-02	4.0646e+02
1.9580e-02	3.9274e+02	2.1792e-02	4.1923e+02	2.4284e-02	3.9677e+02	2.4723e-02	4.2889e+02
1.8496e-02	4.3008e+02	2.0587e-02	4.1484e+02	2.2940e-02	4.1266e+02	2.3355e-02	4.6374e+02
1.7413e-02	4.3932e+02	1.9381e-02	4.4253e+02	2.1597e-02	4.2415e+02	2.1988e-02	4.6018e+02
1.6330e-02	4.8239e+02	1.8176e-02	4.9012e+02	2.0254e-02	4.5267e+02	2.0620e-02	5.2039e+02
1.5247e-02	5.0895e+02	1.6970e-02	5.4233e+02	1.8910e-02	4.9971e+02	1.9253e-02	5.3928e+02
1.4164e-02	5.7313e+02	1.5765e-02	5.5949e+02	1.7567e-02	5.1787e+02	1.7885e-02	6.1022e+02
1.3081e-02	6.1929e+02	1.4559e-02	5.9330e+02	1.6224e-02	5.9481e+02	1.6517e-02	6.3588e+02
1.1998e-02	6.5106e+02	1.3354e-02	6.8839e+02	1.4881e-02	6.3553e+02	1.5150e-02	7.1393e+02
1.0915e-02	7.5203e+02	1.2149e-02	7.2775e+02	1.3537e-02	7.2105e+02	1.3782e-02	8.0336e+02
9.8318e-03	8.2469e+02	1.0943e-02	7.9562e+02	1.2194e-02	8.1031e+02	1.2415e-02	8.2665e+02
8.7488e-03	8.6120e+02	9.7376e-03	8.5382e+02	1.0851e-02	8.8203e+02	1.1047e-02	9.2436e+02
7.6657e-03	9.7002e+02	8.5321e-03	9.8362e+02	9.5074e-03	9.6055e+02	9.6795e-03	9.9851e+02
6.5826e-03	1.0841e+03	7.3266e-03	1.0645e+03	8.1641e-03	1.0797e+03	8.3119e-03	1.1557e+03
5.4995e-03	1.2393e+03	6.1211e-03	1.1906e+03	6.8208e-03	1.2658e+03	6.9443e-03	1.3049e+03
4.4165e-03	1.3685e+03	4.9156e-03	1.3378e+03	5.4775e-03	1.3768e+03	5.5767e-03	1.3895e+03
3.3334e-03	1.5454e+03	3.7101e-03	1.5535e+03	4.1342e-03	1.5574e+03	4.2091e-03	1.5503e+03
2.2503e-03	1.7953e+03	2.5046e-03	1.7514e+03	2.7909e-03	1.7363e+03	2.8415e-03	1.7819e+03
1.1672e-03	2.0009e+03	1.2991e-03	2.0146e+03	1.4477e-03	1.9781e+03	1.4739e-03	2.0018e+03
8.4149e-05	2.2545e+03	9.3659e-05	2.2565e+03	1.0437e-04	2.2574e+03	1.0625e-04	2.2442e+03

FREQUENCY [Hz]: 1.0171E6		1.0759E6		1.1414E6		1.2088E6	
TAU [s]	MAGNITUDES [a.u]	TAU [s]	MAGNITUDES [a.u]	TAU [s]	MAGNITUDES [a.u]	TAU [s]	MAGNITUDES [a.u]
4.4090e-02	2.5204e+02	4.7120e-02	2.8505e+02	5.0036e-02	3.0655e+02	5.1352e-02	2.8098e+02
4.2672e-02	3.0787e+02	4.5604e-02	2.8754e+02	4.8426e-02	3.1460e+02	4.9700e-02	3.0193e+02
4.1253e-02	3.2549e+02	4.4088e-02	3.2691e+02	4.6816e-02	3.2155e+02	4.8047e-02	3.1939e+02
3.9834e-02	3.2654e+02	4.2572e-02	3.3089e+02	4.5206e-02	3.4058e+02	4.6395e-02	3.2354e+02
3.8415e-02	2.9812e+02	4.1055e-02	2.9219e+02	4.3596e-02	3.3493e+02	4.4743e-02	3.3137e+02
3.6997e-02	3.1573e+02	3.9539e-02	3.0361e+02	4.1986e-02	2.9004e+02	4.3090e-02	3.3642e+02
3.5578e-02	2.9261e+02	3.8023e-02	3.3412e+02	4.0376e-02	3.1292e+02	4.1438e-02	3.7750e+02
3.4159e-02	3.1056e+02	3.6507e-02	3.4059e+02	3.8766e-02	3.4130e+02	3.9785e-02	3.1651e+02
3.2741e-02	3.4169e+02	3.4991e-02	3.6029e+02	3.7156e-02	3.7134e+02	3.8133e-02	3.6929e+02
3.1322e-02	3.6923e+02	3.3474e-02	3.7146e+02	3.5546e-02	3.8812e+02	3.6481e-02	3.9371e+02
2.9903e-02	4.0531e+02	3.1958e-02	3.7827e+02	3.3936e-02	3.7287e+02	3.4828e-02	4.2366e+02
2.8484e-02	4.1226e+02	3.0442e-02	4.2138e+02	3.2326e-02	4.1994e+02	3.3176e-02	4.0228e+02
2.7066e-02	3.9632e+02	2.8926e-02	4.0744e+02	3.0716e-02	4.1492e+02	3.1524e-02	4.2738e+02
2.5647e-02	4.1370e+02	2.7409e-02	4.4725e+02	2.9106e-02	4.6553e+02	2.9871e-02	4.4147e+02
2.4228e-02	4.3075e+02	2.5893e-02	4.4578e+02	2.7496e-02	4.5897e+02	2.8219e-02	5.1085e+02
2.2810e-02	4.9338e+02	2.4377e-02	4.9186e+02	2.5886e-02	4.6509e+02	2.6566e-02	5.2805e+02
2.1391e-02	5.5365e+02	2.2861e-02	5.2422e+02	2.4276e-02	5.2331e+02	2.4914e-02	5.5911e+02
1.9972e-02	5.8051e+02	2.1345e-02	5.5662e+02	2.2666e-02	5.7708e+02	2.3262e-02	5.8472e+02
1.8553e-02	6.2172e+02	1.9828e-02	6.0450e+02	2.1056e-02	6.0391e+02	2.1609e-02	6.4721e+02
1.7135e-02	6.2679e+02	1.8312e-02	6.8040e+02	1.9446e-02	6.8344e+02	1.9957e-02	6.7080e+02
1.5716e-02	7.2834e+02	1.6796e-02	7.2763e+02	1.7836e-02	7.1403e+02	1.8305e-02	7.4065e+02
1.4297e-02	7.2331e+02	1.5280e-02	7.8349e+02	1.6226e-02	7.7644e+02	1.6652e-02	8.2520e+02
1.2879e-02	8.4092e+02	1.3764e-02	8.5539e+02	1.4615e-02	8.4720e+02	1.5000e-02	8.8478e+02
1.1460e-02	9.4323e+02	1.2247e-02	9.6259e+02	1.3005e-02	9.5402e+02	1.3347e-02	9.3780e+02
1.0041e-02	1.0333e+03	1.0731e-02	1.0670e+03	1.1395e-02	1.0725e+03	1.1695e-02	1.0560e+03
8.6225e-03	1.1991e+03	9.2150e-03	1.1531e+03	9.7854e-03	1.2069e+03	1.0043e-02	1.1843e+03
7.2038e-03	1.3100e+03	7.6988e-03	1.2994e+03	8.1753e-03	1.2898e+03	8.3903e-03	1.3423e+03
5.7851e-03	1.4531e+03	6.1826e-03	1.4461e+03	6.5653e-03	1.4525e+03	6.7379e-03	1.4834e+03
4.3664e-03	1.6183e+03	4.6664e-03	1.6554e+03	4.9552e-03	1.6460e+03	5.0855e-03	1.6496e+03
2.9477e-03	1.7918e+03	3.1502e-03	1.8407e+03	3.3452e-03	1.8538e+03	3.4331e-03	1.8585e+03
1.5289e-03	2.0540e+03	1.6340e-03	2.1064e+03	1.7351e-03	2.0792e+03	1.7808e-03	2.0375e+03
1.1023e-04	2.2673e+03	1.1780e-04	2.3335e+03	1.2509e-04	2.3899e+03	1.2838e-04	2.3312e+03

FREQUENCY [Hz]: 1.2818E6		1.3588E6		1.44E6		1.5275E6	
TAU [s]	MAGNITUDES [a.u]	TAU [s]	MAGNITUDES [a.u]	TAU [s]	MAGNITUDES [a.u]	TAU [s]	MAGNITUDES [a.u]
5.4778e-02	2.9417e+02	5.6356e-02	3.1925e+02	5.7986e-02	3.3537e+02	5.7476e-02	3.5346e+02
5.3015e-02	3.1596e+02	5.4543e-02	3.3242e+02	5.6121e-02	3.3336e+02	5.5627e-02	3.3563e+02
5.1253e-02	3.2289e+02	5.2729e-02	3.5382e+02	5.4255e-02	3.6521e+02	5.3777e-02	3.6592e+02
4.9490e-02	3.4087e+02	5.0916e-02	3.2786e+02	5.2389e-02	3.2851e+02	5.1928e-02	3.3187e+02
4.7727e-02	3.4652e+02	4.9103e-02	3.2251e+02	5.0523e-02	3.4836e+02	5.0078e-02	3.3935e+02
4.5965e-02	3.4715e+02	4.7289e-02	3.2349e+02	4.8657e-02	3.5190e+02	4.8229e-02	3.5184e+02
4.4202e-02	3.5755e+02	4.5476e-02	3.4565e+02	4.6791e-02	3.4888e+02	4.6380e-02	4.0591e+02
4.2440e-02	3.4961e+02	4.3662e-02	3.4865e+02	4.4925e-02	4.0378e+02	4.4530e-02	4.2030e+02
4.0677e-02	3.5721e+02	4.1849e-02	3.9112e+02	4.3060e-02	4.0588e+02	4.2681e-02	4.1427e+02
3.8914e-02	3.6352e+02	4.0036e-02	3.7629e+02	4.1194e-02	3.8689e+02	4.0831e-02	4.0922e+02
3.7152e-02	3.7488e+02	3.8222e-02	4.2274e+02	3.9328e-02	4.2714e+02	3.8982e-02	4.1417e+02
3.5389e-02	3.9597e+02	3.6409e-02	4.2099e+02	3.7462e-02	4.1971e+02	3.7132e-02	4.6730e+02
3.3627e-02	4.1133e+02	3.4595e-02	4.4675e+02	3.5596e-02	4.7678e+02	3.5283e-02	4.5691e+02
3.1864e-02	4.3693e+02	3.2782e-02	4.4727e+02	3.3730e-02	4.4739e+02	3.3433e-02	5.1992e+02
3.0101e-02	4.6442e+02	3.0969e-02	4.9577e+02	3.1865e-02	5.1981e+02	3.1584e-02	5.2033e+02
2.8339e-02	4.7260e+02	2.9155e-02	4.8870e+02	2.9999e-02	5.5488e+02	2.9735e-02	5.6572e+02
2.6576e-02	5.8602e+02	2.7342e-02	5.6881e+02	2.8133e-02	5.7750e+02	2.7885e-02	5.7429e+02
2.4813e-02	5.4902e+02	2.5528e-02	5.9996e+02	2.6267e-02	6.1897e+02	2.6036e-02	6.2969e+02
2.3051e-02	6.1279e+02	2.3715e-02	6.2900e+02	2.4401e-02	6.6241e+02	2.4186e-02	7.0109e+02
2.1288e-02	6.8278e+02	2.1902e-02	6.5562e+02	2.2535e-02	6.7034e+02	2.2337e-02	6.9790e+02
1.9526e-02	7.2718e+02	2.0088e-02	7.8710e+02	2.0669e-02	7.3739e+02	2.0487e-02	8.0010e+02
1.7763e-02	7.5832e+02	1.8275e-02	8.3516e+02	1.8804e-02	8.2418e+02	1.8638e-02	8.6213e+02
1.6000e-02	8.6212e+02	1.6461e-02	8.7070e+02	1.6938e-02	9.4582e+02	1.6789e-02	9.4213e+02
1.4238e-02	9.5396e+02	1.4648e-02	9.6096e+02	1.5072e-02	9.8332e+02	1.4939e-02	1.0206e+03
1.2475e-02	1.0769e+03	1.2835e-02	1.0385e+03	1.3206e-02	1.0884e+03	1.3090e-02	1.1173e+03
1.0713e-02	1.1445e+03	1.1021e-02	1.1818e+03	1.1340e-02	1.2175e+03	1.1240e-02	1.2391e+03
8.9500e-03	1.2830e+03	9.2079e-03	1.3038e+03	9.4742e-03	1.3422e+03	9.3909e-03	1.4049e+03
7.1874e-03	1.4769e+03	7.3945e-03	1.4657e+03	7.6084e-03	1.5353e+03	7.5414e-03	1.5060e+03
5.4248e-03	1.6479e+03	5.5811e-03	1.6372e+03	5.7425e-03	1.6784e+03	5.6920e-03	1.6837e+03
3.6622e-03	1.8321e+03	3.7677e-03	1.8541e+03	3.8767e-03	1.8696e+03	3.8426e-03	1.8885e+03
1.8996e-03	2.0410e+03	1.9543e-03	2.0733e+03	2.0108e-03	2.0828e+03	1.9931e-03	2.1404e+03
1.3694e-04	2.3461e+03	1.4089e-04	2.3178e+03	1.4497e-04	2.3300e+03	1.4369e-04	2.3549e+03

FREQUENCY [Hz]: 1.6195E6		1.7164E6		1.8204E6		1.9296E6	
TAU [s]	MAGNITUDES [a.u]	TAU [s]	MAGNITUDES [a.u]	TAU [s]	MAGNITUDES [a.u]	TAU [s]	MAGNITUDES [a.u]
5.7448e-02	3.4951e+02	5.7652e-02	3.9574e+02	5.3006e-02	3.6947e+02	4.7106e-02	4.0595e+02
5.5600e-02	3.5182e+02	5.5797e-02	3.7664e+02	5.1300e-02	3.8644e+02	4.5591e-02	4.2181e+02
5.3751e-02	3.8478e+02	5.3941e-02	3.6367e+02	4.9594e-02	3.8740e+02	4.4075e-02	3.9831e+02
5.1902e-02	3.5712e+02	5.2086e-02	3.9818e+02	4.7889e-02	3.9339e+02	4.2559e-02	3.9749e+02
5.0054e-02	3.8168e+02	5.0231e-02	4.1140e+02	4.6183e-02	4.3413e+02	4.1043e-02	4.2381e+02
4.8205e-02	4.2326e+02	4.8376e-02	3.7812e+02	4.4478e-02	4.1069e+02	3.9528e-02	4.5769e+02
4.6357e-02	3.7430e+02	4.6521e-02	3.9278e+02	4.2772e-02	4.4297e+02	3.8012e-02	4.3534e+02
4.4508e-02	4.1274e+02	4.4666e-02	4.3679e+02	4.1066e-02	4.3109e+02	3.6496e-02	4.7262e+02
4.2660e-02	4.6580e+02	4.2811e-02	4.3031e+02	3.9361e-02	4.4448e+02	3.4980e-02	4.6662e+02
4.0811e-02	4.4433e+02	4.0956e-02	4.3929e+02	3.7655e-02	5.0619e+02	3.3464e-02	4.9801e+02
3.8963e-02	4.6934e+02	3.9101e-02	4.4786e+02	3.5950e-02	4.8390e+02	3.1949e-02	5.1266e+02
3.7114e-02	4.6441e+02	3.7246e-02	4.7931e+02	3.4244e-02	5.0968e+02	3.0433e-02	5.5757e+02
3.5266e-02	4.6147e+02	3.5391e-02	4.7352e+02	3.2539e-02	5.1191e+02	2.8917e-02	5.4406e+02
3.3417e-02	5.1390e+02	3.3536e-02	5.1724e+02	3.0833e-02	5.5711e+02	2.7401e-02	6.3202e+02
3.1569e-02	5.3530e+02	3.1681e-02	5.9204e+02	2.9127e-02	5.6715e+02	2.5886e-02	6.6166e+02
2.9720e-02	5.7291e+02	2.9825e-02	5.7719e+02	2.7422e-02	6.3792e+02	2.4370e-02	6.6153e+02
2.7872e-02	6.1325e+02	2.7970e-02	5.9831e+02	2.5716e-02	6.6193e+02	2.2854e-02	6.7900e+02
2.6023e-02	6.0201e+02	2.6115e-02	6.1775e+02	2.4011e-02	7.1535e+02	2.1338e-02	7.5214e+02
2.4175e-02	6.7402e+02	2.4260e-02	6.9916e+02	2.2305e-02	7.6069e+02	1.9823e-02	8.0899e+02
2.2326e-02	7.1908e+02	2.2405e-02	7.6233e+02	2.0599e-02	8.3495e+02	1.8307e-02	8.4815e+02
2.0477e-02	8.0039e+02	2.0550e-02	8.3015e+02	1.8894e-02	8.7255e+02	1.6791e-02	9.3527e+02
1.8629e-02	8.4193e+02	1.8695e-02	8.9717e+02	1.7188e-02	9.4578e+02	1.5275e-02	9.8762e+02
1.6780e-02	9.7004e+02	1.6840e-02	9.6988e+02	1.5483e-02	1.0208e+03	1.3760e-02	1.0485e+03
1.4932e-02	1.0740e+03	1.4985e-02	1.0487e+03	1.3777e-02	1.1169e+03	1.2244e-02	1.1650e+03
1.3083e-02	1.1759e+03	1.3130e-02	1.1788e+03	1.2072e-02	1.1916e+03	1.0728e-02	1.2486e+03
1.1235e-02	1.2167e+03	1.1275e-02	1.2738e+03	1.0366e-02	1.2937e+03	9.2123e-03	1.3674e+03
9.3863e-03	1.4022e+03	9.4195e-03	1.4035e+03	8.6604e-03	1.4138e+03	7.6966e-03	1.4460e+03
7.5377e-03	1.5224e+03	7.5645e-03	1.5246e+03	6.9548e-03	1.5711e+03	6.1808e-03	1.6013e+03
5.6892e-03	1.7009e+03	5.7094e-03	1.7248e+03	5.2493e-03	1.7850e+03	4.6650e-03	1.7586e+03
3.8407e-03	1.8877e+03	3.8543e-03	1.9091e+03	3.5437e-03	1.9246e+03	3.1493e-03	1.9224e+03
1.9921e-03	2.1117e+03	1.9992e-03	2.1738e+03	1.8381e-03	2.1106e+03	1.6335e-03	2.1699e+03
1.4362e-04	2.3314e+03	1.4413e-04	2.3564e+03	1.3251e-04	2.3707e+03	1.1777e-04	2.3763e+03

FREQUENCY [Hz]: 1.9989E6		2.0443E6		2.0534E6		2.1087E6	
TAU [s]	MAGNITUDES [a.u]	TAU [s]	MAGNITUDES [a.u]	TAU [s]	MAGNITUDES [a.u]	TAU [s]	MAGNITUDES [a.u]
4.7881e-02	3.7680e+02	3.9180e-02	4.2854e+02	4.3778e-02	3.9721e+02	3.8437e-02	3.8659e+02
4.4697e-02	3.8112e+02	3.7920e-02	4.3307e+02	4.0867e-02	3.9724e+02	3.5881e-02	3.7711e+02
4.1513e-02	4.3941e+02	3.6659e-02	4.2454e+02	3.7956e-02	4.2057e+02	3.3324e-02	4.2522e+02
3.8328e-02	3.9089e+02	3.5398e-02	4.6991e+02	3.5045e-02	4.3963e+02	3.0768e-02	4.1609e+02
3.5144e-02	4.4892e+02	3.4138e-02	4.3465e+02	3.2133e-02	4.7088e+02	2.8212e-02	4.6069e+02
3.1960e-02	4.8136e+02	3.2877e-02	4.4663e+02	2.9222e-02	4.7602e+02	2.5656e-02	5.2254e+02
2.8776e-02	5.2010e+02	3.1616e-02	4.9500e+02	2.6311e-02	5.2593e+02	2.3100e-02	5.2760e+02
2.5592e-02	6.0334e+02	3.0355e-02	4.9925e+02	2.3400e-02	6.2385e+02	2.0544e-02	5.9861e+02
2.2408e-02	6.8124e+02	2.9095e-02	5.2189e+02	2.0488e-02	6.9132e+02	1.7988e-02	6.8721e+02
1.9224e-02	7.5032e+02	2.7834e-02	5.4275e+02	1.7577e-02	7.4385e+02	1.5432e-02	7.7172e+02
1.6040e-02	8.3870e+02	2.6573e-02	5.9163e+02	1.4666e-02	8.8541e+02	1.2876e-02	9.0858e+02
1.2856e-02	1.0019e+03	2.5312e-02	5.6961e+02	1.1755e-02	1.0522e+03	1.0320e-02	1.0216e+03
9.6719e-03	1.2118e+03	2.4052e-02	6.3578e+02	8.8432e-03	1.1679e+03	7.7642e-03	1.1973e+03
6.4878e-03	1.4312e+03	2.2791e-02	6.0075e+02	5.9320e-03	1.4446e+03	5.2082e-03	1.4556e+03
3.3038e-03	1.7867e+03	2.1530e-02	6.7954e+02	3.0207e-03	1.7600e+03	2.6521e-03	1.7391e+03
1.1970e-04	2.2309e+03	2.0270e-02	7.1177e+02	1.0945e-04	2.1854e+03	9.6091e-05	2.1461e+03
		1.9009e-02	7.5247e+02				
		1.7748e-02	7.8230e+02				
		1.6487e-02	8.2175e+02				
		1.5227e-02	8.8773e+02				
		1.3966e-02	9.5318e+02				
		1.2705e-02	1.0352e+03				
		1.1444e-02	1.0445e+03				
		1.0184e-02	1.1745e+03				
		8.9230e-03	1.2825e+03				
		7.6623e-03	1.3414e+03				
		6.4016e-03	1.4622e+03				
		5.1409e-03	1.6018e+03				
		3.8801e-03	1.8281e+03				
		2.6194e-03	1.9029e+03				
		1.3587e-03	2.1169e+03				
		9.7951e-05	2.2843e+03				

FREQUENCY [Hz]: 2.1647E6		2.167E6		2.2214E6		2.2812E6	
TAU [s]	MAGNITUDES [a.u]	TAU [s]	MAGNITUDES [a.u]	TAU [s]	MAGNITUDES [a.u]	TAU [s]	MAGNITUDES [a.u]
3.7654e-02	3.9879e+02	3.7359e-02	4.3594e+02	3.8391e-02	4.1730e+02	4.2364e-02	3.7389e+02
3.5150e-02	4.3042e+02	3.6157e-02	3.8364e+02	3.5838e-02	4.2333e+02	3.9547e-02	3.9827e+02
3.2646e-02	4.6632e+02	3.4955e-02	4.3536e+02	3.3285e-02	4.4541e+02	3.6730e-02	3.9030e+02
3.0142e-02	4.8936e+02	3.3753e-02	4.2194e+02	3.0732e-02	4.2919e+02	3.3913e-02	4.3657e+02
2.7638e-02	4.8957e+02	3.2551e-02	4.1403e+02	2.8179e-02	4.2365e+02	3.1095e-02	4.6477e+02
2.5134e-02	5.1455e+02	3.1349e-02	4.3400e+02	2.5626e-02	4.9990e+02	2.8278e-02	4.3025e+02
2.2630e-02	5.9709e+02	3.0147e-02	4.6230e+02	2.3073e-02	5.4972e+02	2.5461e-02	5.0864e+02
2.0126e-02	5.9793e+02	2.8944e-02	4.6615e+02	2.0520e-02	5.6092e+02	2.2644e-02	5.4937e+02
1.7622e-02	6.5632e+02	2.7742e-02	4.9362e+02	1.7967e-02	6.5891e+02	1.9826e-02	6.3965e+02
1.5118e-02	7.6656e+02	2.6540e-02	4.8132e+02	1.5414e-02	7.3215e+02	1.7009e-02	6.4983e+02
1.2614e-02	8.7461e+02	2.5338e-02	4.8368e+02	1.2861e-02	8.2272e+02	1.4192e-02	7.9957e+02
1.0110e-02	9.9811e+02	2.4136e-02	5.1855e+02	1.0308e-02	9.7231e+02	1.1375e-02	9.3552e+02
7.6060e-03	1.2446e+03	2.2934e-02	5.4847e+02	7.7551e-03	1.2140e+03	8.5576e-03	1.1244e+03
5.1021e-03	1.5048e+03	2.1732e-02	5.8344e+02	5.2020e-03	1.4321e+03	5.7403e-03	1.4112e+03
2.5981e-03	1.7875e+03	2.0530e-02	6.2245e+02	2.6490e-03	1.7714e+03	2.9231e-03	1.8131e+03
9.4134e-05	2.2157e+03	1.9327e-02	6.4026e+02	9.5979e-05	2.2341e+03	1.0591e-04	2.2263e+03
		1.8125e-02	6.3776e+02				
		1.6923e-02	7.1689e+02				
		1.5721e-02	7.5608e+02				
		1.4519e-02	8.2780e+02				
		1.3317e-02	8.4890e+02				
		1.2115e-02	8.9157e+02				
		1.0913e-02	1.0062e+03				
		9.7104e-03	1.0766e+03				
		8.5083e-03	1.1914e+03				
		7.3061e-03	1.2697e+03				
		6.1040e-03	1.3572e+03				
		4.9019e-03	1.5315e+03				
		3.6998e-03	1.6839e+03				
		2.4976e-03	1.8525e+03				
		1.2955e-03	2.0752e+03				
		9.3398e-05	2.2989e+03				

FREQUENCY [Hz]: 2.2981E6		2.3419E6		2.4058E6		2.4356E6	
TAU [s]	MAGNITUDES [a.u]	TAU [s]	MAGNITUDES [a.u]	TAU [s]	MAGNITUDES [a.u]	TAU [s]	MAGNITUDES [a.u]
5.3932e-02	4.2144e+02	4.8816e-02	3.8508e+02	6.0101e-02	4.0549e+02	7.4263e-02	4.0526e+02
5.2196e-02	4.1506e+02	4.5570e-02	3.8720e+02	5.6105e-02	3.8782e+02	7.1874e-02	3.9171e+02
5.0461e-02	4.0310e+02	4.2324e-02	4.0383e+02	5.2108e-02	3.9472e+02	6.9484e-02	4.1058e+02
4.8725e-02	3.9887e+02	3.9077e-02	4.1776e+02	4.8111e-02	3.8983e+02	6.7094e-02	3.8264e+02
4.6990e-02	4.1373e+02	3.5831e-02	4.2962e+02	4.4114e-02	4.1674e+02	6.4705e-02	3.9987e+02
4.5255e-02	4.2274e+02	3.2585e-02	4.5563e+02	4.0118e-02	4.1289e+02	6.2315e-02	3.8614e+02
4.3519e-02	4.1095e+02	2.9338e-02	4.7779e+02	3.6121e-02	4.8809e+02	5.9926e-02	3.9903e+02
4.1784e-02	4.2089e+02	2.6092e-02	5.1852e+02	3.2124e-02	4.9842e+02	5.7536e-02	3.8955e+02
4.0049e-02	4.4440e+02	2.2846e-02	6.0660e+02	2.8127e-02	5.6086e+02	5.5146e-02	3.8887e+02
3.8313e-02	4.0199e+02	1.9600e-02	6.9231e+02	2.4131e-02	6.6142e+02	5.2757e-02	4.1230e+02
3.6578e-02	4.0436e+02	1.6353e-02	7.6013e+02	2.0134e-02	7.0155e+02	5.0367e-02	3.8844e+02
3.4842e-02	4.1942e+02	1.3107e-02	9.1368e+02	1.6137e-02	8.5165e+02	4.7978e-02	4.1284e+02
3.3107e-02	4.6306e+02	9.8608e-03	1.1051e+03	1.2140e-02	1.0639e+03	4.5588e-02	4.3705e+02
3.1372e-02	4.5329e+02	6.6146e-03	1.3667e+03	8.1437e-03	1.3071e+03	4.3198e-02	4.8326e+02
2.9636e-02	4.3922e+02	3.3683e-03	1.7610e+03	4.1470e-03	1.7229e+03	4.0809e-02	4.8160e+02
2.7901e-02	4.4599e+02	1.2204e-04	2.2004e+03	1.5025e-04	2.2494e+03	3.8419e-02	5.1726e+02
2.6166e-02	4.9049e+02					3.6030e-02	5.0469e+02
2.4430e-02	5.1239e+02					3.3640e-02	5.4339e+02
2.2695e-02	5.7700e+02					3.1250e-02	5.9119e+02
2.0959e-02	6.0463e+02					2.8861e-02	6.1296e+02
1.9224e-02	6.2097e+02					2.6471e-02	6.2548e+02
1.7489e-02	6.4059e+02					2.4082e-02	6.8646e+02
1.5753e-02	7.4687e+02					2.1692e-02	7.5944e+02
1.4018e-02	8.2568e+02					1.9302e-02	8.3954e+02
1.2282e-02	9.2451e+02					1.6913e-02	9.3830e+02
1.0547e-02	1.0217e+03					1.4523e-02	1.0648e+03
8.8117e-03	1.1354e+03					1.2134e-02	1.1948e+03
7.0763e-03	1.3410e+03					9.7440e-03	1.3827e+03
5.3410e-03	1.5076e+03					7.3544e-03	1.5522e+03
3.6056e-03	1.7788e+03					4.9648e-03	1.8128e+03
1.8702e-03	1.9874e+03					2.5753e-03	2.1140e+03
1.3483e-04	2.3306e+03					1.8566e-04	2.3543e+03

FREQUENCY [Hz]: 2.4706E6		2.5362E6		2.5822E6		2.6027E6	
TAU [s]	MAGNITUDES [a.u]	TAU [s]	MAGNITUDES [a.u]	TAU [s]	MAGNITUDES [a.u]	TAU [s]	MAGNITUDES [a.u]
7.0225e-02	3.7999e+02	7.4452e-02	4.0742e+02	6.2022e-02	4.6915e+02	7.3176e-02	4.6267e+02
6.5555e-02	4.0424e+02	6.9501e-02	4.2216e+02	6.0027e-02	4.6808e+02	6.8310e-02	4.3761e+02
6.0885e-02	3.8154e+02	6.4550e-02	4.2127e+02	5.8031e-02	4.8757e+02	6.3444e-02	4.7173e+02
5.6215e-02	4.3522e+02	5.9599e-02	4.4752e+02	5.6035e-02	4.7468e+02	5.8578e-02	4.5764e+02
5.1545e-02	4.5254e+02	5.4648e-02	4.8129e+02	5.4039e-02	4.9786e+02	5.3711e-02	4.9239e+02
4.6875e-02	4.7261e+02	4.9697e-02	4.6029e+02	5.2044e-02	5.2272e+02	4.8845e-02	5.3710e+02
4.2205e-02	4.5586e+02	4.4746e-02	5.2211e+02	5.0048e-02	5.4091e+02	4.3979e-02	6.0607e+02
3.7535e-02	5.2685e+02	3.9795e-02	5.8122e+02	4.8052e-02	5.4404e+02	3.9113e-02	5.8585e+02
3.2865e-02	6.0008e+02	3.4844e-02	6.5007e+02	4.6057e-02	5.8251e+02	3.4246e-02	7.3077e+02
2.8195e-02	6.7360e+02	2.9892e-02	7.2743e+02	4.4061e-02	5.9374e+02	2.9380e-02	7.6177e+02
2.3525e-02	7.8824e+02	2.4941e-02	8.2100e+02	4.2065e-02	5.9102e+02	2.4514e-02	9.1458e+02
1.8856e-02	9.2584e+02	1.9990e-02	9.8048e+02	4.0069e-02	6.2388e+02	1.9648e-02	1.0382e+03
1.4186e-02	1.1373e+03	1.5039e-02	1.1628e+03	3.8074e-02	6.2690e+02	1.4782e-02	1.2781e+03
9.5155e-03	1.4053e+03	1.0088e-02	1.4182e+03	3.6078e-02	6.7677e+02	9.9154e-03	1.5185e+03
4.8455e-03	1.7434e+03	5.1372e-03	1.8301e+03	3.4082e-02	7.1537e+02	5.0492e-03	1.9039e+03
1.7556e-04	2.2874e+03	1.8613e-04	2.2344e+03	3.2087e-02	7.6616e+02	1.8294e-04	2.3284e+03
				3.0091e-02	7.5719e+02		
				2.8095e-02	8.7323e+02		
				2.6099e-02	9.0392e+02		
				2.4104e-02	9.1047e+02		
				2.2108e-02	9.7464e+02		
				2.0112e-02	1.0827e+03		
				1.8117e-02	1.1844e+03		
				1.6121e-02	1.2671e+03		
				1.4125e-02	1.3669e+03		
				1.2129e-02	1.4577e+03		
				1.0134e-02	1.5677e+03		
				8.1379e-03	1.7187e+03		
				6.1422e-03	1.8318e+03		
				4.1465e-03	2.0484e+03		
				2.1508e-03	2.2753e+03		
				1.5506e-04	2.4552e+03		

FREQUENCY [Hz]: 2.6727E6		2.7383E6		2.7436E6		2.8181E6	
TAU [s]	MAGNITUDES [a.u]	TAU [s]	MAGNITUDES [a.u]	TAU [s]	MAGNITUDES [a.u]	TAU [s]	MAGNITUDES [a.u]
5.9229e-02	4.3077e+02	3.6214e-02	6.0648e+02	4.5677e-02	5.1166e+02	3.6650e-02	4.2440e+02
5.5290e-02	4.5949e+02	3.5049e-02	6.1850e+02	4.2639e-02	5.1642e+02	3.4213e-02	4.8536e+02
5.1352e-02	4.7632e+02	3.3884e-02	6.2970e+02	3.9602e-02	5.2239e+02	3.1776e-02	4.8822e+02
4.7413e-02	5.0710e+02	3.2718e-02	6.3051e+02	3.6564e-02	5.6050e+02	2.9339e-02	5.3425e+02
4.3474e-02	5.2343e+02	3.1553e-02	6.5448e+02	3.3527e-02	5.5627e+02	2.6901e-02	5.7601e+02
3.9535e-02	5.5820e+02	3.0388e-02	6.8093e+02	3.0489e-02	6.1401e+02	2.4464e-02	6.0520e+02
3.5597e-02	6.5141e+02	2.9222e-02	6.6950e+02	2.7452e-02	6.6444e+02	2.2027e-02	6.5653e+02
3.1658e-02	7.0058e+02	2.8057e-02	7.5252e+02	2.4414e-02	7.0825e+02	1.9590e-02	6.9459e+02
2.7719e-02	7.8062e+02	2.6892e-02	7.3797e+02	2.1377e-02	8.3754e+02	1.7152e-02	7.8320e+02
2.3780e-02	9.0351e+02	2.5727e-02	8.0329e+02	1.8339e-02	9.0621e+02	1.4715e-02	9.0253e+02
1.9842e-02	9.7356e+02	2.4561e-02	8.1631e+02	1.5302e-02	1.0577e+03	1.2278e-02	9.5125e+02
1.5903e-02	1.1405e+03	2.3396e-02	8.5117e+02	1.2264e-02	1.1895e+03	9.8406e-03	1.1388e+03
1.1964e-02	1.3523e+03	2.2231e-02	8.5646e+02	9.2267e-03	1.4059e+03	7.4034e-03	1.3143e+03
8.0255e-03	1.5874e+03	2.1065e-02	9.1539e+02	6.1892e-03	1.5839e+03	4.9661e-03	1.5154e+03
4.0868e-03	1.8294e+03	1.9900e-02	9.6302e+02	3.1517e-03	1.8633e+03	2.5289e-03	1.7947e+03
1.4807e-04	2.2360e+03	1.8735e-02	9.9382e+02	1.1419e-04	2.2533e+03	9.1626e-05	2.1235e+03
		1.7570e-02	1.0022e+03				
		1.6404e-02	1.0703e+03				
		1.5239e-02	1.1182e+03				
		1.4074e-02	1.2270e+03				
		1.2909e-02	1.2928e+03				
		1.1743e-02	1.3177e+03				
		1.0578e-02	1.3308e+03				
		9.4127e-03	1.4500e+03				
		8.2475e-03	1.5988e+03				
		7.0822e-03	1.6745e+03				
		5.9169e-03	1.7214e+03				
		4.7516e-03	1.8504e+03				
		3.5864e-03	2.0089e+03				
		2.4211e-03	2.1533e+03				
		1.2558e-03	2.2486e+03				
		9.0535e-05	2.3487e+03				

FREQUENCY [Hz]: 2.8936E6		2.9018E6		2.9701E6		3.0504E6	
TAU [s]	MAGNITUDES [a.u]	TAU [s]	MAGNITUDES [a.u]	TAU [s]	MAGNITUDES [a.u]	TAU [s]	MAGNITUDES [a.u]
3.6578e-02	4.1923e+02	5.9457e-02	4.8035e+02	5.2550e-02	4.4484e+02	7.6175e-02	4.5999e+02
3.4145e-02	4.5940e+02	5.7544e-02	4.2894e+02	4.9056e-02	4.3496e+02	7.1109e-02	4.3241e+02
3.1713e-02	4.3821e+02	5.5631e-02	4.5003e+02	4.5561e-02	4.2366e+02	6.6043e-02	4.2326e+02
2.9280e-02	5.0235e+02	5.3717e-02	4.3596e+02	4.2067e-02	4.6361e+02	6.0978e-02	4.4965e+02
2.6848e-02	5.2405e+02	5.1804e-02	4.2887e+02	3.8572e-02	4.7380e+02	5.5912e-02	4.5192e+02
2.4416e-02	5.2186e+02	4.9891e-02	4.0748e+02	3.5077e-02	4.8273e+02	5.0847e-02	4.4178e+02
2.1983e-02	5.7992e+02	4.7978e-02	4.3021e+02	3.1583e-02	5.3640e+02	4.5781e-02	4.6792e+02
1.9551e-02	6.4886e+02	4.6065e-02	4.7481e+02	2.8088e-02	5.2415e+02	4.0715e-02	5.0873e+02
1.7118e-02	6.8321e+02	4.4152e-02	4.6085e+02	2.4594e-02	5.5691e+02	3.5650e-02	5.1080e+02
1.4686e-02	7.9094e+02	4.2238e-02	4.5246e+02	2.1099e-02	6.3785e+02	3.0584e-02	5.8241e+02
1.2254e-02	8.6239e+02	4.0325e-02	4.5157e+02	1.7604e-02	7.0531e+02	2.5519e-02	6.6023e+02
9.8211e-03	1.0017e+03	3.8412e-02	4.7389e+02	1.4110e-02	8.4220e+02	2.0453e-02	7.9870e+02
7.3887e-03	1.2012e+03	3.6499e-02	4.7634e+02	1.0615e-02	1.0006e+03	1.5387e-02	1.0234e+03
4.9563e-03	1.4302e+03	3.4586e-02	4.9955e+02	7.1206e-03	1.3265e+03	1.0322e-02	1.2322e+03
2.5239e-03	1.7204e+03	3.2673e-02	4.4905e+02	3.6260e-03	1.7646e+03	5.2561e-03	1.6759e+03
9.1444e-05	2.2069e+03	3.0759e-02	4.7694e+02	1.3138e-04	2.3463e+03	1.9044e-04	2.2543e+03
		2.8846e-02	4.9855e+02				
		2.6933e-02	5.4913e+02				
		2.5020e-02	5.4905e+02				
		2.3107e-02	5.7435e+02				
		2.1194e-02	6.2800e+02				
		1.9280e-02	6.6903e+02				
		1.7367e-02	7.1421e+02				
		1.5454e-02	7.8570e+02				
		1.3541e-02	8.9213e+02				
		1.1628e-02	9.5313e+02				
		9.7145e-03	1.0509e+03				
		7.8013e-03	1.2089e+03				
		5.8882e-03	1.4295e+03				
		3.9750e-03	1.6420e+03				
		2.0618e-03	1.9819e+03				
		1.4864e-04	2.3122e+03				

FREQUENCY [Hz]: 3.0784E6		3.1318E6		3.2142E6		3.263E6	
TAU [s]	MAGNITUDES [a.u]	TAU [s]	MAGNITUDES [a.u]	TAU [s]	MAGNITUDES [a.u]	TAU [s]	MAGNITUDES [a.u]
9.9193e-02	4.3615e+02	9.0731e-02	4.6715e+02	1.0641e-01	4.5955e+02	1.1702e-01	4.5935e+02
9.6001e-02	4.7468e+02	8.4697e-02	4.1150e+02	9.9338e-02	4.6761e+02	1.1326e-01	4.8365e+02
9.2809e-02	4.6377e+02	7.8664e-02	4.3860e+02	9.2261e-02	4.7121e+02	1.0949e-01	4.6538e+02
8.9617e-02	4.4393e+02	7.2630e-02	4.6734e+02	8.5185e-02	4.6757e+02	1.0572e-01	4.9461e+02
8.6425e-02	4.4652e+02	6.6597e-02	4.9615e+02	7.8108e-02	4.9584e+02	1.0196e-01	4.7298e+02
8.3234e-02	4.3456e+02	6.0563e-02	5.2066e+02	7.1031e-02	5.0647e+02	9.8194e-02	4.6253e+02
8.0042e-02	4.9830e+02	5.4529e-02	5.3674e+02	6.3955e-02	5.1218e+02	9.4429e-02	4.7540e+02
7.6850e-02	4.8263e+02	4.8496e-02	5.7157e+02	5.6878e-02	5.4707e+02	9.0663e-02	5.0317e+02
7.3658e-02	4.4517e+02	4.2462e-02	6.1214e+02	4.9802e-02	6.3316e+02	8.6898e-02	5.0576e+02
7.0467e-02	4.7936e+02	3.6429e-02	6.9461e+02	4.2725e-02	6.8305e+02	8.3132e-02	5.3789e+02
6.7275e-02	4.9144e+02	3.0395e-02	8.1828e+02	3.5649e-02	8.1922e+02	7.9367e-02	5.2033e+02
6.4083e-02	4.7965e+02	2.4361e-02	9.5922e+02	2.8572e-02	9.8866e+02	7.5601e-02	5.3130e+02
6.0891e-02	4.6931e+02	1.8328e-02	1.1858e+03	2.1496e-02	1.2071e+03	7.1836e-02	5.5607e+02
5.7700e-02	4.6507e+02	1.2294e-02	1.4423e+03	1.4419e-02	1.4674e+03	6.8071e-02	5.5821e+02
5.4508e-02	5.2424e+02	6.2604e-03	1.8388e+03	7.3426e-03	1.8436e+03	6.4305e-02	6.0207e+02
5.1316e-02	5.0741e+02	2.2683e-04	2.3596e+03	2.6604e-04	2.3864e+03	6.0540e-02	6.4480e+02
4.8124e-02	5.0517e+02					5.6774e-02	6.4123e+02
4.4933e-02	5.9168e+02					5.3009e-02	6.9301e+02
4.1741e-02	5.9819e+02					4.9243e-02	7.2616e+02
3.8549e-02	6.1014e+02					4.5478e-02	7.6416e+02
3.5357e-02	6.1348e+02					4.1712e-02	8.5330e+02
3.2166e-02	6.9248e+02					3.7947e-02	8.8176e+02
2.8974e-02	7.4902e+02					3.4182e-02	9.5105e+02
2.5782e-02	8.2171e+02					3.0416e-02	1.0418e+03
2.2590e-02	8.9395e+02					2.6651e-02	1.1424e+03
1.9399e-02	1.0327e+03					2.2885e-02	1.2607e+03
1.6207e-02	1.1245e+03					1.9120e-02	1.4203e+03
1.3015e-02	1.3117e+03					1.5354e-02	1.5687e+03
9.8233e-03	1.5009e+03					1.1589e-02	1.7647e+03
6.6315e-03	1.7521e+03					7.8234e-03	1.9387e+03
3.4397e-03	2.0646e+03					4.0580e-03	2.2560e+03
2.4798e-04	2.4597e+03					2.9255e-04	2.5075e+03

FREQUENCY [Hz]: 3.3006E6		3.459E6		3.6668E6		3.887E6	
TAU [s]	MAGNITUDES [a.u]	TAU [s]	MAGNITUDES [a.u]	TAU [s]	MAGNITUDES [a.u]	TAU [s]	MAGNITUDES [a.u]
1.0462e-01	4.8873e+02	1.2400e-01	5.2589e+02	1.3260e-01	4.9608e+02	1.3357e-01	5.1924e+02
9.7661e-02	4.7835e+02	1.2001e-01	5.2859e+02	1.2833e-01	5.4772e+02	1.2927e-01	5.3837e+02
9.0704e-02	4.9717e+02	1.1602e-01	4.9012e+02	1.2406e-01	4.9564e+02	1.2497e-01	5.2820e+02
8.3747e-02	4.9266e+02	1.1203e-01	5.4675e+02	1.1980e-01	5.2643e+02	1.2067e-01	5.6597e+02
7.6790e-02	5.2641e+02	1.0804e-01	5.4337e+02	1.1553e-01	5.3769e+02	1.1637e-01	5.4869e+02
6.9833e-02	5.7779e+02	1.0405e-01	5.4563e+02	1.1126e-01	5.4380e+02	1.1208e-01	5.6656e+02
6.2876e-02	6.0073e+02	1.0006e-01	5.5157e+02	1.0700e-01	5.1292e+02	1.0778e-01	5.6640e+02
5.5919e-02	6.6582e+02	9.6067e-02	5.6381e+02	1.0273e-01	5.5729e+02	1.0348e-01	6.0336e+02
4.8961e-02	7.1932e+02	9.2077e-02	5.7168e+02	9.8463e-02	5.6934e+02	9.9183e-02	5.9413e+02
4.2004e-02	8.2466e+02	8.8087e-02	5.5456e+02	9.4197e-02	5.8762e+02	9.4885e-02	6.2669e+02
3.5047e-02	9.2557e+02	8.4098e-02	5.8376e+02	8.9930e-02	6.4256e+02	9.0587e-02	6.5805e+02
2.8090e-02	1.1263e+03	8.0108e-02	6.6945e+02	8.5663e-02	6.1666e+02	8.6290e-02	6.4074e+02
2.1133e-02	1.2974e+03	7.6118e-02	6.1138e+02	8.1397e-02	6.5962e+02	8.1992e-02	6.6276e+02
1.4176e-02	1.5936e+03	7.2128e-02	6.6858e+02	7.7130e-02	6.7341e+02	7.7694e-02	7.1782e+02
7.2187e-03	1.9892e+03	6.8138e-02	6.9389e+02	7.2864e-02	6.8067e+02	7.3396e-02	7.2316e+02
2.6155e-04	2.3912e+03	6.4148e-02	7.3923e+02	6.8597e-02	7.2313e+02	6.9098e-02	7.3349e+02
		6.0158e-02	7.2721e+02	6.4330e-02	7.6837e+02	6.4801e-02	7.8534e+02
		5.6168e-02	7.8930e+02	6.0064e-02	7.9211e+02	6.0503e-02	8.5490e+02
		5.2178e-02	8.0945e+02	5.5797e-02	8.4724e+02	5.6205e-02	8.5372e+02
		4.8189e-02	8.7327e+02	5.1531e-02	8.7901e+02	5.1907e-02	9.0801e+02
		4.4199e-02	9.6293e+02	4.7264e-02	9.5096e+02	4.7610e-02	1.0054e+03
		4.0209e-02	1.0375e+03	4.2997e-02	9.8880e+02	4.3312e-02	1.0484e+03
		3.6219e-02	1.0909e+03	3.8731e-02	1.0932e+03	3.9014e-02	1.1131e+03
		3.2229e-02	1.1736e+03	3.4464e-02	1.1951e+03	3.4716e-02	1.2033e+03
		2.8239e-02	1.2690e+03	3.0198e-02	1.2224e+03	3.0418e-02	1.3409e+03
		2.4249e-02	1.3808e+03	2.5931e-02	1.4191e+03	2.6121e-02	1.4088e+03
		2.0259e-02	1.5587e+03	2.1664e-02	1.5488e+03	2.1823e-02	1.5373e+03
		1.6270e-02	1.6820e+03	1.7398e-02	1.7138e+03	1.7525e-02	1.6875e+03
		1.2280e-02	1.8768e+03	1.3131e-02	1.7816e+03	1.3227e-02	1.9027e+03
		8.2898e-03	2.1068e+03	8.8647e-03	2.0642e+03	8.9295e-03	2.0767e+03
		4.2999e-03	2.3274e+03	4.5981e-03	2.3052e+03	4.6317e-03	2.3269e+03
		3.0999e-04	2.6077e+03	3.3149e-04	2.5454e+03	3.3391e-04	2.5363e+03

FREQUENCY [Hz]: 4.1201E6		4.3699E6		4.6305E6		4.9093E6	
TAU [s]	MAGNITUDES [a.u]	TAU [s]	MAGNITUDES [a.u]	TAU [s]	MAGNITUDES [a.u]	TAU [s]	MAGNITUDES [a.u]
1.5031e-01	5.8784e+02	1.5388e-01	6.0628e+02	1.6541e-01	5.8708e+02	1.7309e-01	6.2147e+02
1.4548e-01	5.5992e+02	1.4893e-01	6.0622e+02	1.6008e-01	6.3294e+02	1.6752e-01	6.1506e+02
1.4064e-01	5.4551e+02	1.4398e-01	5.7634e+02	1.5476e-01	6.1198e+02	1.6195e-01	6.4856e+02
1.3580e-01	5.9526e+02	1.3903e-01	5.9858e+02	1.4944e-01	5.8135e+02	1.5638e-01	5.9446e+02
1.3097e-01	5.9033e+02	1.3408e-01	6.0651e+02	1.4412e-01	5.9140e+02	1.5081e-01	6.6604e+02
1.2613e-01	5.6570e+02	1.2913e-01	6.0818e+02	1.3879e-01	6.3265e+02	1.4524e-01	6.2367e+02
1.2129e-01	5.9230e+02	1.2417e-01	5.9527e+02	1.3347e-01	6.2924e+02	1.3967e-01	6.8235e+02
1.1646e-01	6.2646e+02	1.1922e-01	6.5967e+02	1.2815e-01	6.3987e+02	1.3410e-01	6.8311e+02
1.1162e-01	6.0229e+02	1.1427e-01	6.1934e+02	1.2283e-01	6.5065e+02	1.2853e-01	6.9382e+02
1.0678e-01	6.7043e+02	1.0932e-01	6.8045e+02	1.1750e-01	6.6001e+02	1.2296e-01	6.8223e+02
1.0195e-01	6.4758e+02	1.0437e-01	6.7570e+02	1.1218e-01	7.1316e+02	1.1739e-01	6.7981e+02
9.7110e-02	6.4424e+02	9.9417e-02	6.8509e+02	1.0686e-01	7.0693e+02	1.1182e-01	7.0666e+02
9.2273e-02	6.8263e+02	9.4465e-02	7.4887e+02	1.0154e-01	7.2849e+02	1.0625e-01	7.5181e+02
8.7437e-02	7.2065e+02	8.9514e-02	7.3595e+02	9.6216e-02	7.5126e+02	1.0068e-01	7.4505e+02
8.2600e-02	7.2243e+02	8.4562e-02	8.0388e+02	9.0893e-02	7.6000e+02	9.5113e-02	7.7342e+02
7.7763e-02	7.3817e+02	7.9610e-02	7.9083e+02	8.5571e-02	7.9098e+02	8.9544e-02	8.1702e+02
7.2926e-02	7.8145e+02	7.4659e-02	7.9726e+02	8.0249e-02	8.1770e+02	8.3974e-02	8.8447e+02
6.8090e-02	8.2556e+02	6.9707e-02	8.7274e+02	7.4926e-02	9.0395e+02	7.8405e-02	9.2001e+02
6.3253e-02	8.9809e+02	6.4756e-02	9.1806e+02	6.9604e-02	8.8763e+02	7.2835e-02	9.1176e+02
5.8416e-02	9.3193e+02	5.9804e-02	9.7320e+02	6.4282e-02	9.3182e+02	6.7266e-02	9.9111e+02
5.3580e-02	9.2364e+02	5.4852e-02	1.0470e+03	5.8959e-02	1.0267e+03	6.1697e-02	1.0315e+03
4.8743e-02	1.0176e+03	4.9901e-02	1.0757e+03	5.3637e-02	1.0921e+03	5.6127e-02	1.1133e+03
4.3906e-02	1.0916e+03	4.4949e-02	1.1357e+03	4.8315e-02	1.1311e+03	5.0558e-02	1.1670e+03
3.9069e-02	1.1520e+03	3.9998e-02	1.2477e+03	4.2992e-02	1.2340e+03	4.4988e-02	1.2603e+03
3.4233e-02	1.2715e+03	3.5046e-02	1.3707e+03	3.7670e-02	1.3552e+03	3.9419e-02	1.3384e+03
2.9396e-02	1.4084e+03	3.0094e-02	1.4482e+03	3.2348e-02	1.4712e+03	3.3849e-02	1.4808e+03
2.4559e-02	1.5726e+03	2.5143e-02	1.5638e+03	2.7025e-02	1.5721e+03	2.8280e-02	1.5719e+03
1.9723e-02	1.6815e+03	2.0191e-02	1.7109e+03	2.1703e-02	1.7535e+03	2.2710e-02	1.7703e+03
1.4886e-02	1.8290e+03	1.5240e-02	1.9145e+03	1.6381e-02	1.9278e+03	1.7141e-02	1.9026e+03
1.0049e-02	2.0753e+03	1.0288e-02	2.1052e+03	1.1058e-02	2.1033e+03	1.1572e-02	2.1346e+03
5.2125e-03	2.3184e+03	5.3363e-03	2.2808e+03	5.7358e-03	2.3709e+03	6.0022e-03	2.3626e+03
3.7578e-04	2.6311e+03	3.8471e-04	2.5666e+03	4.1351e-04	2.6514e+03	4.3271e-04	2.5474e+03

FREQUENCY [Hz]: 5.2035E6		5.5175E6		5.8485E6		6.2009E6	
TAU [s]	MAGNITUDES [a.u]	TAU [s]	MAGNITUDES [a.u]	TAU [s]	MAGNITUDES [a.u]	TAU [s]	MAGNITUDES [a.u]
1.8211e-01	6.4547e+02	1.8528e-01	6.7375e+02	1.9687e-01	7.1032e+02	2.0974e-01	7.4859e+02
1.7625e-01	6.5321e+02	1.7932e-01	6.8688e+02	1.9054e-01	7.1539e+02	2.0299e-01	7.7205e+02
1.7039e-01	6.5298e+02	1.7336e-01	6.5961e+02	1.8420e-01	7.2184e+02	1.9624e-01	7.9800e+02
1.6453e-01	6.3537e+02	1.6739e-01	6.9807e+02	1.7787e-01	7.3552e+02	1.8949e-01	7.7792e+02
1.5867e-01	6.9761e+02	1.6143e-01	7.1270e+02	1.7153e-01	7.4512e+02	1.8274e-01	7.8212e+02
1.5281e-01	6.6271e+02	1.5547e-01	7.2120e+02	1.6520e-01	7.6924e+02	1.7600e-01	7.6550e+02
1.4695e-01	6.7182e+02	1.4951e-01	6.8178e+02	1.5886e-01	7.3829e+02	1.6925e-01	7.7049e+02
1.4109e-01	6.8779e+02	1.4355e-01	7.1874e+02	1.5253e-01	7.7764e+02	1.6250e-01	8.3903e+02
1.3523e-01	7.1307e+02	1.3758e-01	7.1682e+02	1.4619e-01	7.8263e+02	1.5575e-01	8.5060e+02
1.2937e-01	7.3000e+02	1.3162e-01	7.4656e+02	1.3986e-01	7.8178e+02	1.4900e-01	8.5754e+02
1.2351e-01	7.2993e+02	1.2566e-01	7.4156e+02	1.3352e-01	8.1007e+02	1.4225e-01	8.5172e+02
1.1765e-01	7.0181e+02	1.1970e-01	7.6414e+02	1.2719e-01	7.8710e+02	1.3550e-01	8.8807e+02
1.1179e-01	7.5732e+02	1.1374e-01	8.1856e+02	1.2085e-01	8.4498e+02	1.2875e-01	8.9757e+02
1.0593e-01	8.0563e+02	1.0778e-01	8.1699e+02	1.1452e-01	8.2667e+02	1.2200e-01	9.2753e+02
1.0007e-01	8.0771e+02	1.0181e-01	8.5076e+02	1.0818e-01	8.7120e+02	1.1526e-01	9.0384e+02
9.4213e-02	8.5743e+02	9.5852e-02	8.5618e+02	1.0185e-01	8.8717e+02	1.0851e-01	9.5251e+02
8.8353e-02	8.4285e+02	8.9890e-02	9.0866e+02	9.5514e-02	9.5797e+02	1.0176e-01	9.9256e+02
8.2493e-02	8.8676e+02	8.3929e-02	9.4858e+02	8.9179e-02	9.8550e+02	9.5009e-02	1.0134e+03
7.6633e-02	9.3258e+02	7.7967e-02	9.8502e+02	8.2845e-02	1.0027e+03	8.8260e-02	1.0245e+03
7.0774e-02	9.8698e+02	7.2005e-02	1.0568e+03	7.6510e-02	1.0169e+03	8.1511e-02	1.1366e+03
6.4914e-02	1.0317e+03	6.6043e-02	1.0701e+03	7.0175e-02	1.1317e+03	7.4762e-02	1.1739e+03
5.9054e-02	1.0930e+03	6.0081e-02	1.1567e+03	6.3840e-02	1.1851e+03	6.8013e-02	1.2278e+03
5.3194e-02	1.1827e+03	5.4119e-02	1.2125e+03	5.7505e-02	1.2706e+03	6.1265e-02	1.2728e+03
4.7334e-02	1.2421e+03	4.8158e-02	1.2853e+03	5.1171e-02	1.3158e+03	5.4516e-02	1.3645e+03
4.1474e-02	1.3675e+03	4.2196e-02	1.4229e+03	4.4836e-02	1.4340e+03	4.7767e-02	1.4513e+03
3.5614e-02	1.4241e+03	3.6234e-02	1.5076e+03	3.8501e-02	1.5637e+03	4.1018e-02	1.5870e+03
2.9755e-02	1.5893e+03	3.0272e-02	1.6485e+03	3.2166e-02	1.6777e+03	3.4269e-02	1.6693e+03
2.3895e-02	1.7511e+03	2.4310e-02	1.8189e+03	2.5831e-02	1.8475e+03	2.7520e-02	1.8243e+03
1.8035e-02	1.9210e+03	1.8349e-02	1.9074e+03	1.9497e-02	1.9940e+03	2.0771e-02	1.9868e+03
1.2175e-02	2.0787e+03	1.2387e-02	2.1602e+03	1.3162e-02	2.1968e+03	1.4022e-02	2.1775e+03
6.3151e-03	2.2748e+03	6.4250e-03	2.3799e+03	6.8270e-03	2.3465e+03	7.2733e-03	2.4202e+03
4.5528e-04	2.5923e+03	4.6320e-04	2.5708e+03	4.9218e-04	2.6564e+03	5.2435e-04	2.6680e+03

FREQUENCY [Hz]: 6.576E6		6.9705E6		7.3895E6		7.8341E6	
TAU [s]	MAGNITUDES [a.u]	TAU [s]	MAGNITUDES [a.u]	TAU [s]	MAGNITUDES [a.u]	TAU [s]	MAGNITUDES [a.u]
2.2148e-01	7.7294e+02	2.3392e-01	7.4836e+02	2.3628e-01	8.6728e+02	2.4947e-01	9.1080e+02
2.1436e-01	7.6895e+02	2.2640e-01	7.8366e+02	2.2868e-01	8.5532e+02	2.4144e-01	9.0943e+02
2.0723e-01	7.8483e+02	2.1887e-01	8.0244e+02	2.2107e-01	8.7236e+02	2.3341e-01	9.4250e+02
2.0010e-01	7.9376e+02	2.1134e-01	8.0202e+02	2.1347e-01	8.3753e+02	2.2538e-01	9.7174e+02
1.9298e-01	7.9757e+02	2.0382e-01	7.9464e+02	2.0587e-01	8.7077e+02	2.1736e-01	9.5472e+02
1.8585e-01	7.9719e+02	1.9629e-01	8.1945e+02	1.9826e-01	8.5949e+02	2.0933e-01	9.6114e+02
1.7872e-01	7.8202e+02	1.8876e-01	8.3522e+02	1.9066e-01	9.0065e+02	2.0130e-01	9.2400e+02
1.7159e-01	8.3094e+02	1.8123e-01	8.2237e+02	1.8306e-01	9.0364e+02	1.9328e-01	9.8596e+02
1.6447e-01	7.9191e+02	1.7371e-01	8.7057e+02	1.7546e-01	9.1683e+02	1.8525e-01	9.5665e+02
1.5734e-01	8.3196e+02	1.6618e-01	8.5826e+02	1.6785e-01	8.8936e+02	1.7722e-01	9.8587e+02
1.5021e-01	8.5492e+02	1.5865e-01	8.7239e+02	1.6025e-01	9.4094e+02	1.6919e-01	1.0255e+03
1.4309e-01	8.6093e+02	1.5113e-01	8.9772e+02	1.5265e-01	9.5132e+02	1.6117e-01	1.0188e+03
1.3596e-01	8.9008e+02	1.4360e-01	9.0564e+02	1.4504e-01	9.4147e+02	1.5314e-01	1.0319e+03
1.2883e-01	9.4405e+02	1.3607e-01	9.3441e+02	1.3744e-01	1.0039e+03	1.4511e-01	1.0617e+03
1.2171e-01	9.3712e+02	1.2854e-01	9.4620e+02	1.2984e-01	1.0132e+03	1.3709e-01	1.0752e+03
1.1458e-01	9.5098e+02	1.2102e-01	9.4646e+02	1.2224e-01	1.0354e+03	1.2906e-01	1.1244e+03
1.0745e-01	9.8029e+02	1.1349e-01	1.0032e+03	1.1463e-01	1.1190e+03	1.2103e-01	1.1802e+03
1.0033e-01	1.0354e+03	1.0596e-01	1.0683e+03	1.0703e-01	1.0960e+03	1.1300e-01	1.1713e+03
9.3201e-02	1.0668e+03	9.8437e-02	1.0885e+03	9.9427e-02	1.1251e+03	1.0498e-01	1.1598e+03
8.6074e-02	1.1189e+03	9.0910e-02	1.1003e+03	9.1824e-02	1.2036e+03	9.6950e-02	1.2403e+03
7.8948e-02	1.1809e+03	8.3383e-02	1.1994e+03	8.4222e-02	1.2276e+03	8.8923e-02	1.2743e+03
7.1821e-02	1.2198e+03	7.5855e-02	1.2044e+03	7.6619e-02	1.3134e+03	8.0896e-02	1.3239e+03
6.4694e-02	1.2884e+03	6.8328e-02	1.3462e+03	6.9016e-02	1.3378e+03	7.2868e-02	1.4076e+03
5.7567e-02	1.3722e+03	6.0801e-02	1.3624e+03	6.1413e-02	1.4406e+03	6.4841e-02	1.5099e+03
5.0441e-02	1.4080e+03	5.3274e-02	1.4401e+03	5.3810e-02	1.5662e+03	5.6814e-02	1.5768e+03
4.3314e-02	1.5958e+03	4.5747e-02	1.5179e+03	4.6208e-02	1.6298e+03	4.8787e-02	1.6876e+03
3.6187e-02	1.7471e+03	3.8220e-02	1.7272e+03	3.8605e-02	1.7694e+03	4.0760e-02	1.8385e+03
2.9061e-02	1.8457e+03	3.0693e-02	1.8211e+03	3.1002e-02	1.8858e+03	3.2732e-02	1.9136e+03
2.1934e-02	1.9911e+03	2.3166e-02	1.9838e+03	2.3399e-02	2.0500e+03	2.4705e-02	2.0867e+03
1.4807e-02	2.1624e+03	1.5639e-02	2.2081e+03	1.5796e-02	2.1891e+03	1.6678e-02	2.2260e+03
7.6804e-03	2.4125e+03	8.1119e-03	2.3560e+03	8.1935e-03	2.3947e+03	8.6509e-03	2.4421e+03
5.5370e-04	2.6583e+03	5.8481e-04	2.5950e+03	5.9069e-04	2.6543e+03	6.2367e-04	2.7061e+03

FREQUENCY [Hz]: 8.3055E6		8.8048E6		9.3334E6		9.8924E6	
TAU [s]	MAGNITUDES [a.u]	TAU [s]	MAGNITUDES [a.u]	TAU [s]	MAGNITUDES [a.u]	TAU [s]	MAGNITUDES [a.u]
2.6846e-01	9.6160e+02	2.7605e-01	1.0280e+03	2.9014e-01	1.1070e+03	3.0976e-01	1.1902e+03
2.5983e-01	9.3440e+02	2.6717e-01	9.8089e+02	2.8080e-01	1.0846e+03	2.9979e-01	1.2177e+03
2.5119e-01	9.5530e+02	2.5829e-01	1.0137e+03	2.7147e-01	1.0995e+03	2.8983e-01	1.2085e+03
2.4255e-01	9.3829e+02	2.4940e-01	1.0165e+03	2.6213e-01	1.0952e+03	2.7986e-01	1.2058e+03
2.3391e-01	9.8235e+02	2.4052e-01	1.0712e+03	2.5279e-01	1.0909e+03	2.6989e-01	1.2271e+03
2.2527e-01	9.8329e+02	2.3164e-01	1.0400e+03	2.4346e-01	1.1029e+03	2.5993e-01	1.2594e+03
2.1663e-01	9.8207e+02	2.2276e-01	1.0513e+03	2.3412e-01	1.1065e+03	2.4996e-01	1.2723e+03
2.0799e-01	1.0162e+03	2.1387e-01	1.0254e+03	2.2479e-01	1.0905e+03	2.3999e-01	1.2715e+03
1.9936e-01	1.0065e+03	2.0499e-01	1.0756e+03	2.1545e-01	1.1418e+03	2.3002e-01	1.2430e+03
1.9072e-01	1.0179e+03	1.9611e-01	1.0414e+03	2.0611e-01	1.1295e+03	2.2006e-01	1.2736e+03
1.8208e-01	1.0199e+03	1.8723e-01	1.1266e+03	1.9678e-01	1.1502e+03	2.1009e-01	1.3347e+03
1.7344e-01	1.0532e+03	1.7834e-01	1.0992e+03	1.8744e-01	1.1886e+03	2.0012e-01	1.3118e+03
1.6480e-01	1.0732e+03	1.6946e-01	1.1234e+03	1.7811e-01	1.1940e+03	1.9015e-01	1.3139e+03
1.5616e-01	1.1196e+03	1.6058e-01	1.1552e+03	1.6877e-01	1.2665e+03	1.8019e-01	1.3043e+03
1.4753e-01	1.0970e+03	1.5169e-01	1.1586e+03	1.5943e-01	1.2426e+03	1.7022e-01	1.3830e+03
1.3889e-01	1.1259e+03	1.4281e-01	1.2069e+03	1.5010e-01	1.2631e+03	1.6025e-01	1.3755e+03
1.3025e-01	1.1518e+03	1.3393e-01	1.2331e+03	1.4076e-01	1.2921e+03	1.5028e-01	1.3793e+03
1.2161e-01	1.2042e+03	1.2505e-01	1.2390e+03	1.3143e-01	1.2785e+03	1.4032e-01	1.4471e+03
1.1297e-01	1.2509e+03	1.1616e-01	1.2744e+03	1.2209e-01	1.3570e+03	1.3035e-01	1.4705e+03
1.0433e-01	1.2940e+03	1.0728e-01	1.2965e+03	1.1276e-01	1.4177e+03	1.2038e-01	1.5295e+03
9.5695e-02	1.3016e+03	9.8399e-02	1.3412e+03	1.0342e-01	1.4660e+03	1.1042e-01	1.5363e+03
8.7056e-02	1.4038e+03	8.9516e-02	1.4281e+03	9.4084e-02	1.4814e+03	1.0045e-01	1.5704e+03
7.8418e-02	1.4477e+03	8.0634e-02	1.4982e+03	8.4748e-02	1.5504e+03	9.0480e-02	1.6625e+03
6.9779e-02	1.4995e+03	7.1751e-02	1.5566e+03	7.5412e-02	1.5897e+03	8.0513e-02	1.6973e+03
6.1141e-02	1.6044e+03	6.2868e-02	1.6636e+03	6.6076e-02	1.6891e+03	7.0546e-02	1.8035e+03
5.2502e-02	1.7238e+03	5.3986e-02	1.7318e+03	5.6740e-02	1.7839e+03	6.0578e-02	1.8820e+03
4.3864e-02	1.7957e+03	4.5103e-02	1.9021e+03	4.7405e-02	1.8578e+03	5.0611e-02	1.9614e+03
3.5225e-02	1.9153e+03	3.6221e-02	2.0025e+03	3.8069e-02	2.0568e+03	4.0644e-02	2.0564e+03
2.6587e-02	2.1107e+03	2.7338e-02	2.1195e+03	2.8733e-02	2.1829e+03	3.0676e-02	2.2093e+03
1.7948e-02	2.2313e+03	1.8455e-02	2.2977e+03	1.9397e-02	2.3881e+03	2.0709e-02	2.3547e+03
9.3096e-03	2.4627e+03	9.5727e-03	2.4583e+03	1.0061e-02	2.5396e+03	1.0742e-02	2.5404e+03
6.7116e-04	2.6794e+03	6.9013e-04	2.7030e+03	7.2534e-04	2.7225e+03	7.7440e-04	2.7509e+03

FREQUENCY [Hz]: 1.0488E7		1.1118E7		1.1787E7		1.2493E7	
TAU [s]	MAGNITUDES [a.u]	TAU [s]	MAGNITUDES [a.u]	TAU [s]	MAGNITUDES [a.u]	TAU [s]	MAGNITUDES [a.u]
3.1786e-01	1.1560e+03	3.4629e-01	1.1448e+03	3.4381e-01	1.2386e+03	3.5678e-01	1.3398e+03
2.6200e-01	1.1088e+03	2.8543e-01	1.0945e+03	2.8339e-01	1.1801e+03	2.9408e-01	1.2964e+03
2.1595e-01	1.0865e+03	2.3527e-01	1.1059e+03	2.3358e-01	1.1650e+03	2.4239e-01	1.2339e+03
1.7800e-01	1.0640e+03	1.9392e-01	1.0153e+03	1.9253e-01	1.1457e+03	1.9980e-01	1.2198e+03
1.4672e-01	9.8495e+02	1.5984e-01	9.8788e+02	1.5870e-01	1.0796e+03	1.6468e-01	1.1310e+03
1.2093e-01	9.6560e+02	1.3175e-01	9.3180e+02	1.3081e-01	9.8133e+02	1.3574e-01	1.0795e+03
9.9681e-02	8.7501e+02	1.0860e-01	8.6288e+02	1.0782e-01	9.2076e+02	1.1189e-01	1.0341e+03
8.2162e-02	8.2078e+02	8.9511e-02	8.4324e+02	8.8871e-02	8.7195e+02	9.2222e-02	9.0092e+02
6.7723e-02	7.5066e+02	7.3780e-02	7.8707e+02	7.3252e-02	8.0451e+02	7.6015e-02	8.7557e+02
5.5821e-02	6.5511e+02	6.0814e-02	6.9873e+02	6.0379e-02	7.1383e+02	6.2656e-02	7.7758e+02
4.6011e-02	6.3558e+02	5.0126e-02	6.4067e+02	4.9767e-02	6.3393e+02	5.1644e-02	6.8864e+02
3.7925e-02	5.6924e+02	4.1317e-02	5.1480e+02	4.1021e-02	5.6200e+02	4.2568e-02	5.8749e+02
3.1260e-02	5.0439e+02	3.4056e-02	4.8869e+02	3.3812e-02	5.2759e+02	3.5087e-02	5.7592e+02
2.5766e-02	4.5645e+02	2.8071e-02	4.9344e+02	2.7870e-02	4.7335e+02	2.8921e-02	5.2339e+02
2.1238e-02	4.1136e+02	2.3137e-02	4.3049e+02	2.2972e-02	3.8310e+02	2.3838e-02	4.1199e+02
1.7505e-02	3.9235e+02	1.9071e-02	3.9078e+02	1.8935e-02	3.7296e+02	1.9649e-02	4.2164e+02
1.4429e-02	3.4543e+02	1.5720e-02	3.5261e+02	1.5607e-02	3.6078e+02	1.6196e-02	3.7102e+02
1.1893e-02	3.4384e+02	1.2957e-02	2.9938e+02	1.2864e-02	3.1918e+02	1.3349e-02	3.4543e+02
9.8031e-03	3.1492e+02	1.0680e-02	3.3481e+02	1.0603e-02	3.1575e+02	1.1003e-02	3.1880e+02
8.0802e-03	2.9311e+02	8.8030e-03	2.9198e+02	8.7399e-03	3.1352e+02	9.0696e-03	2.7756e+02
6.6602e-03	2.6744e+02	7.2559e-03	2.7837e+02	7.2040e-03	2.7786e+02	7.4756e-03	2.7956e+02
5.4897e-03	2.5167e+02	5.9807e-03	2.5118e+02	5.9379e-03	2.3171e+02	6.1619e-03	2.5581e+02
4.5249e-03	2.5519e+02	4.9297e-03	2.6117e+02	4.8944e-03	2.2639e+02	5.0790e-03	2.5933e+02
3.7297e-03	2.6540e+02	4.0633e-03	2.4483e+02	4.0342e-03	2.2849e+02	4.1864e-03	2.6726e+02
3.0742e-03	2.2590e+02	3.3492e-03	2.3665e+02	3.3252e-03	2.3152e+02	3.4506e-03	2.3458e+02
2.5340e-03	2.4920e+02	2.7606e-03	2.3385e+02	2.7408e-03	2.1996e+02	2.8442e-03	2.1956e+02
2.0886e-03	2.1522e+02	2.2754e-03	2.3404e+02	2.2592e-03	2.4117e+02	2.3444e-03	2.2035e+02
1.7216e-03	2.4023e+02	1.8756e-03	2.0535e+02	1.8621e-03	2.0969e+02	1.9324e-03	2.0697e+02
1.4190e-03	2.3337e+02	1.5459e-03	2.1803e+02	1.5349e-03	2.2975e+02	1.5928e-03	2.0974e+02
1.1696e-03	1.9207e+02	1.2742e-03	2.1799e+02	1.2651e-03	2.0893e+02	1.3128e-03	2.1352e+02
9.6408e-04	2.1541e+02	1.0503e-03	2.1882e+02	1.0428e-03	2.0198e+02	1.0821e-03	2.3198e+02
7.9465e-04	2.1305e+02	8.6572e-04	2.0195e+02	8.5953e-04	1.9917e+02	8.9194e-04	2.2075e+02

FREQUENCY [Hz]: 1.3249E7		1.4045E7		1.4888E7		1.5782E7	
TAU [s]	MAGNITUDES [a.u]	TAU [s]	MAGNITUDES [a.u]	TAU [s]	MAGNITUDES [a.u]	TAU [s]	MAGNITUDES [a.u]
4.0175e-01	1.3386e+03	4.1277e-01	1.4644e+03	4.2039e-01	1.5714e+03	4.4007e-01	1.6220e+03
3.3114e-01	1.3533e+03	3.4023e-01	1.4690e+03	3.4651e-01	1.5326e+03	3.6273e-01	1.5658e+03
2.7295e-01	1.2661e+03	2.8043e-01	1.3753e+03	2.8561e-01	1.4920e+03	2.9898e-01	1.5228e+03
2.2498e-01	1.2579e+03	2.3115e-01	1.3577e+03	2.3542e-01	1.4028e+03	2.4644e-01	1.5191e+03
1.8544e-01	1.1768e+03	1.9053e-01	1.2747e+03	1.9404e-01	1.3652e+03	2.0313e-01	1.4143e+03
1.5285e-01	1.1838e+03	1.5704e-01	1.2193e+03	1.5994e-01	1.2468e+03	1.6743e-01	1.3146e+03
1.2599e-01	1.0718e+03	1.2944e-01	1.1859e+03	1.3183e-01	1.1798e+03	1.3801e-01	1.2468e+03
1.0385e-01	9.7042e+02	1.0669e-01	1.0382e+03	1.0866e-01	1.1009e+03	1.1375e-01	1.1139e+03
8.5596e-02	8.9435e+02	8.7944e-02	9.4249e+02	8.9567e-02	9.8612e+02	9.3761e-02	1.0228e+03
7.0553e-02	8.3109e+02	7.2488e-02	8.6073e+02	7.3826e-02	8.8959e+02	7.7283e-02	8.9092e+02
5.8154e-02	7.3825e+02	5.9749e-02	7.5662e+02	6.0852e-02	7.7167e+02	6.3701e-02	8.6021e+02
4.7934e-02	6.5755e+02	4.9248e-02	7.1372e+02	5.0158e-02	7.1858e+02	5.2506e-02	7.1710e+02
3.9510e-02	5.9438e+02	4.0593e-02	6.1949e+02	4.1343e-02	5.9928e+02	4.3279e-02	6.6449e+02
3.2566e-02	5.3222e+02	3.3459e-02	5.2841e+02	3.4077e-02	5.6028e+02	3.5673e-02	5.7208e+02
2.6843e-02	4.5658e+02	2.7579e-02	4.7975e+02	2.8088e-02	4.9958e+02	2.9403e-02	5.2464e+02
2.2125e-02	4.3936e+02	2.2732e-02	4.4333e+02	2.3152e-02	4.7854e+02	2.4236e-02	4.4188e+02
1.8237e-02	3.8150e+02	1.8737e-02	4.1518e+02	1.9083e-02	3.9812e+02	1.9977e-02	4.0958e+02
1.5032e-02	3.3396e+02	1.5444e-02	3.6763e+02	1.5729e-02	3.6143e+02	1.6466e-02	3.9790e+02
1.2390e-02	3.2649e+02	1.2730e-02	3.2670e+02	1.2965e-02	3.3726e+02	1.3572e-02	3.5065e+02
1.0213e-02	3.3337e+02	1.0493e-02	3.2172e+02	1.0687e-02	3.2238e+02	1.1187e-02	3.5064e+02
8.4179e-03	2.9686e+02	8.6488e-03	2.7238e+02	8.8085e-03	2.7494e+02	9.2209e-03	3.0930e+02
6.9385e-03	2.4514e+02	7.1288e-03	2.8621e+02	7.2604e-03	2.7375e+02	7.6004e-03	2.7401e+02
5.7191e-03	2.7000e+02	5.8760e-03	2.6009e+02	5.9845e-03	2.5558e+02	6.2647e-03	2.7107e+02
4.7140e-03	2.5707e+02	4.8433e-03	2.4430e+02	4.9327e-03	2.5090e+02	5.1637e-03	2.5194e+02
3.8856e-03	2.4117e+02	3.9921e-03	2.3755e+02	4.0658e-03	2.4875e+02	4.2562e-03	2.6083e+02
3.2027e-03	2.1447e+02	3.2906e-03	2.2901e+02	3.3513e-03	2.2028e+02	3.5082e-03	2.8176e+02
2.6399e-03	2.3502e+02	2.7123e-03	2.5492e+02	2.7623e-03	2.2800e+02	2.8917e-03	2.4501e+02
2.1759e-03	2.5776e+02	2.2356e-03	2.1098e+02	2.2769e-03	2.1473e+02	2.3835e-03	2.0363e+02
1.7935e-03	1.9129e+02	1.8427e-03	2.4352e+02	1.8767e-03	2.6159e+02	1.9646e-03	2.2196e+02
1.4783e-03	1.7911e+02	1.5189e-03	2.2141e+02	1.5469e-03	2.3007e+02	1.6193e-03	2.3679e+02
1.2185e-03	2.0058e+02	1.2519e-03	2.1972e+02	1.2750e-03	2.0708e+02	1.3348e-03	2.0701e+02
1.0044e-03	1.8712e+02	1.0319e-03	2.3400e+02	1.0510e-03	2.1740e+02	1.1002e-03	2.2893e+02

FREQUENCY [Hz]: 1.6734E7		1.7741E7		1.8805E7		1.9935E7	
TAU [s]	MAGNITUDES [a.u]	TAU [s]	MAGNITUDES [a.u]	TAU [s]	MAGNITUDES [a.u]	TAU [s]	MAGNITUDES [a.u]
4.4922e-01	1.7528e+03	4.7818e-01	1.7908e+03	5.0889e-01	1.9258e+03	5.6348e-01	2.1008e+03
3.7028e-01	1.6873e+03	3.9415e-01	1.7509e+03	4.1946e-01	1.8342e+03	4.6445e-01	1.9873e+03
3.0520e-01	1.6646e+03	3.2488e-01	1.6920e+03	3.4574e-01	1.8228e+03	3.8283e-01	2.0172e+03
2.5157e-01	1.5900e+03	2.6778e-01	1.6252e+03	2.8498e-01	1.7410e+03	3.1555e-01	1.9248e+03
2.0735e-01	1.4907e+03	2.2072e-01	1.5576e+03	2.3490e-01	1.6410e+03	2.6009e-01	1.8150e+03
1.7091e-01	1.4317e+03	1.8193e-01	1.4589e+03	1.9361e-01	1.5878e+03	2.1438e-01	1.7064e+03
1.4088e-01	1.3323e+03	1.4996e-01	1.3842e+03	1.5959e-01	1.4393e+03	1.7671e-01	1.5903e+03
1.1612e-01	1.2149e+03	1.2360e-01	1.3026e+03	1.3154e-01	1.3660e+03	1.4565e-01	1.4872e+03
9.5711e-02	1.0948e+03	1.0188e-01	1.1542e+03	1.0842e-01	1.2181e+03	1.2005e-01	1.3473e+03
7.8891e-02	1.0010e+03	8.3977e-02	1.0115e+03	8.9369e-02	1.0963e+03	9.8956e-02	1.2262e+03
6.5026e-02	8.7161e+02	6.9218e-02	9.2481e+02	7.3663e-02	9.8003e+02	8.1565e-02	1.0858e+03
5.3598e-02	8.1186e+02	5.7054e-02	8.3444e+02	6.0717e-02	8.2015e+02	6.7230e-02	9.4116e+02
4.4179e-02	7.0002e+02	4.7027e-02	7.2507e+02	5.0047e-02	7.6278e+02	5.5415e-02	8.2383e+02
3.6415e-02	5.9901e+02	3.8762e-02	6.3497e+02	4.1251e-02	6.5381e+02	4.5676e-02	7.8142e+02
3.0015e-02	5.4215e+02	3.1950e-02	5.7814e+02	3.4002e-02	6.0922e+02	3.7649e-02	6.8987e+02
2.4740e-02	4.8742e+02	2.6335e-02	4.8545e+02	2.8026e-02	5.1608e+02	3.1032e-02	5.8950e+02
2.0392e-02	4.4989e+02	2.1707e-02	4.3555e+02	2.3101e-02	4.8068e+02	2.5579e-02	5.2027e+02
1.6808e-02	3.8850e+02	1.7892e-02	4.3552e+02	1.9041e-02	4.1103e+02	2.1083e-02	4.6254e+02
1.3854e-02	3.8247e+02	1.4748e-02	3.9941e+02	1.5695e-02	4.2807e+02	1.7378e-02	4.2485e+02
1.1420e-02	3.3209e+02	1.2156e-02	3.3566e+02	1.2936e-02	3.9489e+02	1.4324e-02	3.6949e+02
9.4127e-03	3.0747e+02	1.0020e-02	2.9056e+02	1.0663e-02	3.3719e+02	1.1807e-02	3.2463e+02
7.7585e-03	3.0391e+02	8.2587e-03	2.9986e+02	8.7890e-03	3.2276e+02	9.7318e-03	3.2867e+02
6.3950e-03	2.5826e+02	6.8073e-03	2.8697e+02	7.2444e-03	2.9266e+02	8.0215e-03	3.0275e+02
5.2711e-03	2.5830e+02	5.6109e-03	2.6673e+02	5.9712e-03	2.6393e+02	6.6118e-03	2.9416e+02
4.3448e-03	2.2666e+02	4.6248e-03	2.5286e+02	4.9218e-03	2.5237e+02	5.4498e-03	2.6797e+02
3.5812e-03	2.4731e+02	3.8121e-03	2.1297e+02	4.0568e-03	2.3848e+02	4.4920e-03	2.5318e+02
2.9518e-03	2.3600e+02	3.1421e-03	2.5449e+02	3.3439e-03	2.4479e+02	3.7026e-03	2.3375e+02
2.4331e-03	2.2265e+02	2.5899e-03	2.1542e+02	2.7562e-03	2.1495e+02	3.0519e-03	2.4584e+02
2.0055e-03	2.3976e+02	2.1348e-03	2.3384e+02	2.2718e-03	2.5296e+02	2.5155e-03	2.3715e+02
1.6530e-03	2.3321e+02	1.7596e-03	2.3968e+02	1.8726e-03	2.0564e+02	2.0734e-03	2.3670e+02
1.3625e-03	2.0868e+02	1.4503e-03	2.0912e+02	1.5435e-03	2.3567e+02	1.7091e-03	2.4399e+02
1.1231e-03	2.3451e+02	1.1955e-03	2.2691e+02	1.2722e-03	2.2238e+02	1.4087e-03	2.0895e+02

FREQUENCY [Hz]: 2.1136E7		2.2401E7		2.375E7		2.5179E7	
TAU [s]	MAGNITUDES [a.u]	TAU [s]	MAGNITUDES [a.u]	TAU [s]	MAGNITUDES [a.u]	TAU [s]	MAGNITUDES [a.u]
5.9562e-01	2.2185e+03	6.3047e-01	2.3032e+03	6.8186e-01	2.5352e+03	7.3546e-01	2.6869e+03
4.9094e-01	2.1576e+03	5.1967e-01	2.2587e+03	5.6203e-01	2.4620e+03	6.0621e-01	2.6005e+03
4.0466e-01	2.0903e+03	4.2834e-01	2.2183e+03	4.6325e-01	2.3313e+03	4.9967e-01	2.4898e+03
3.3354e-01	2.0384e+03	3.5306e-01	2.0806e+03	3.8184e-01	2.2498e+03	4.1186e-01	2.4262e+03
2.7493e-01	1.9307e+03	2.9101e-01	1.9907e+03	3.1473e-01	2.1717e+03	3.3948e-01	2.2944e+03
2.2661e-01	1.7606e+03	2.3987e-01	1.8664e+03	2.5942e-01	2.0426e+03	2.7982e-01	2.1511e+03
1.8679e-01	1.6712e+03	1.9771e-01	1.7831e+03	2.1383e-01	1.8890e+03	2.3064e-01	2.0320e+03
1.5396e-01	1.5209e+03	1.6297e-01	1.6032e+03	1.7625e-01	1.6826e+03	1.9011e-01	1.8330e+03
1.2690e-01	1.3714e+03	1.3433e-01	1.4175e+03	1.4528e-01	1.5582e+03	1.5670e-01	1.6675e+03
1.0460e-01	1.2358e+03	1.1072e-01	1.3359e+03	1.1975e-01	1.3850e+03	1.2916e-01	1.5151e+03
8.6217e-02	1.1478e+03	9.1262e-02	1.1912e+03	9.8701e-02	1.2287e+03	1.0646e-01	1.3159e+03
7.1065e-02	9.9858e+02	7.5223e-02	1.0828e+03	8.1355e-02	1.1188e+03	8.7750e-02	1.1657e+03
5.8576e-02	9.0065e+02	6.2003e-02	9.1556e+02	6.7057e-02	1.0106e+03	7.2329e-02	1.0191e+03
4.8281e-02	7.6270e+02	5.1107e-02	8.2990e+02	5.5272e-02	8.7058e+02	5.9618e-02	9.5880e+02
3.9796e-02	6.9138e+02	4.2125e-02	6.9884e+02	4.5559e-02	7.4720e+02	4.9140e-02	7.9630e+02
3.2802e-02	6.0636e+02	3.4722e-02	6.4783e+02	3.7552e-02	6.7203e+02	4.0504e-02	7.0336e+02
2.7038e-02	5.3940e+02	2.8620e-02	5.6391e+02	3.0952e-02	5.6509e+02	3.3386e-02	6.2240e+02
2.2286e-02	5.1841e+02	2.3590e-02	4.9132e+02	2.5513e-02	5.2768e+02	2.7518e-02	5.4805e+02
1.8369e-02	4.5344e+02	1.9444e-02	4.7797e+02	2.1029e-02	4.8461e+02	2.2682e-02	4.7031e+02
1.5141e-02	3.7758e+02	1.6027e-02	3.8647e+02	1.7333e-02	4.5797e+02	1.8696e-02	4.4452e+02
1.2480e-02	3.3478e+02	1.3210e-02	3.2828e+02	1.4287e-02	3.8737e+02	1.5410e-02	4.2158e+02
1.0287e-02	3.2441e+02	1.0889e-02	3.5297e+02	1.1776e-02	3.1687e+02	1.2702e-02	3.2092e+02
8.4790e-03	3.1240e+02	8.9751e-03	2.9261e+02	9.7067e-03	3.2141e+02	1.0470e-02	3.4102e+02
6.9889e-03	2.7464e+02	7.3978e-03	3.0422e+02	8.0008e-03	3.2120e+02	8.6298e-03	3.1080e+02
5.7606e-03	2.5771e+02	6.0977e-03	2.8408e+02	6.5947e-03	2.8459e+02	7.1132e-03	3.0473e+02
4.7482e-03	2.6669e+02	5.0261e-03	2.6712e+02	5.4357e-03	2.7124e+02	5.8631e-03	2.7302e+02
3.9138e-03	2.5018e+02	4.1428e-03	2.2851e+02	4.4804e-03	2.5010e+02	4.8327e-03	2.7954e+02
3.2259e-03	2.5312e+02	3.4147e-03	2.7599e+02	3.6930e-03	2.3711e+02	3.9834e-03	2.2713e+02
2.6590e-03	2.0848e+02	2.8146e-03	2.5765e+02	3.0440e-03	2.4308e+02	3.2833e-03	2.4190e+02
2.1917e-03	2.1813e+02	2.3199e-03	2.2950e+02	2.5090e-03	2.4212e+02	2.7063e-03	2.3237e+02
1.8065e-03	2.1998e+02	1.9122e-03	2.5074e+02	2.0681e-03	2.3727e+02	2.2307e-03	2.5330e+02
1.4890e-03	2.2726e+02	1.5762e-03	2.5526e+02	1.7046e-03	2.2316e+02	1.8387e-03	2.1127e+02

FREQUENCY [Hz]: 2.669E7		2.8296E7		3.0002E7	
TAU [s]	MAGNITUDES [a.u]	TAU [s]	MAGNITUDES [a.u]	TAU [s]	MAGNITUDES [a.u]
8.0544e-01	2.9722e+03	8.9171e-01	3.1318e+03	8.0000e-01	3.2565e+03
6.6389e-01	2.8361e+03	7.3500e-01	3.1030e+03	6.5941e-01	3.1835e+03
5.4722e-01	2.7971e+03	6.0583e-01	2.9984e+03	5.4352e-01	3.0266e+03
4.5105e-01	2.7231e+03	4.9936e-01	2.8447e+03	4.4800e-01	2.9241e+03
3.7178e-01	2.5080e+03	4.1160e-01	2.7284e+03	3.6927e-01	2.7269e+03
3.0644e-01	2.3961e+03	3.3926e-01	2.5676e+03	3.0437e-01	2.5168e+03
2.5259e-01	2.1772e+03	2.7964e-01	2.4155e+03	2.5088e-01	2.2928e+03
2.0820e-01	2.0150e+03	2.3050e-01	2.1357e+03	2.0679e-01	2.0747e+03
1.7161e-01	1.7911e+03	1.8999e-01	1.9595e+03	1.7045e-01	1.8144e+03
1.4145e-01	1.6793e+03	1.5660e-01	1.7831e+03	1.4049e-01	1.6104e+03
1.1659e-01	1.4512e+03	1.2908e-01	1.5970e+03	1.1580e-01	1.4112e+03
9.6100e-02	1.2754e+03	1.0639e-01	1.3756e+03	9.5451e-02	1.2687e+03
7.9211e-02	1.1761e+03	8.7695e-02	1.2214e+03	7.8676e-02	1.1081e+03
6.5290e-02	9.7874e+02	7.2283e-02	1.0542e+03	6.4849e-02	9.7925e+02
5.3816e-02	8.3835e+02	5.9580e-02	9.5333e+02	5.3452e-02	8.3714e+02
4.4358e-02	7.7856e+02	4.9109e-02	8.1734e+02	4.4058e-02	7.1772e+02
3.6563e-02	7.0766e+02	4.0479e-02	6.9950e+02	3.6315e-02	6.2947e+02
3.0137e-02	6.1902e+02	3.3365e-02	6.5229e+02	2.9933e-02	5.4618e+02
2.4841e-02	5.0717e+02	2.7501e-02	5.9229e+02	2.4673e-02	5.1307e+02
2.0475e-02	4.7627e+02	2.2668e-02	4.9071e+02	2.0337e-02	4.7898e+02
1.6877e-02	4.1121e+02	1.8684e-02	4.5321e+02	1.6763e-02	4.0464e+02
1.3911e-02	3.9419e+02	1.5401e-02	4.2216e+02	1.3817e-02	3.9771e+02
1.1466e-02	3.6563e+02	1.2694e-02	3.8071e+02	1.1389e-02	3.3217e+02
9.4509e-03	3.1041e+02	1.0463e-02	3.1286e+02	9.3871e-03	2.9937e+02
7.7900e-03	2.7640e+02	8.6244e-03	2.9717e+02	7.7373e-03	3.1352e+02
6.4210e-03	2.8575e+02	7.1087e-03	2.6686e+02	6.3776e-03	2.5980e+02
5.2925e-03	2.9226e+02	5.8594e-03	2.7863e+02	5.2567e-03	2.5901e+02
4.3624e-03	2.7380e+02	4.8296e-03	2.7407e+02	4.3329e-03	2.5980e+02
3.5957e-03	2.2099e+02	3.9809e-03	2.6539e+02	3.5714e-03	2.4715e+02
2.9638e-03	2.1001e+02	3.2813e-03	2.5432e+02	2.9438e-03	2.0898e+02
2.4429e-03	2.0509e+02	2.7046e-03	2.4647e+02	2.4264e-03	1.9617e+02
2.0136e-03	2.2770e+02	2.2293e-03	2.3052e+02	2.0000e-03	2.5108e+02

4. [1]H magnetization of lysozyme:

FREQUENCY [Hz]: 14342		17353		19296		21114	
TAU [s]	MAGNITUDES [a.u]	TAU [s]	MAGNITUDES [a.u]	TAU [s]	MAGNITUDES [a.u]	TAU [s]	MAGNITUDES [a.u]
3.4943e-03	4.4240e+02	2.9626e-03	3.7692e+02	2.8996e-03	3.9937e+02	3.6627e-03	4.7397e+02
3.2619e-03	4.0135e+02	2.7656e-03	4.3856e+02	2.7067e-03	4.1104e+02	3.4191e-03	4.7082e+02
3.0295e-03	3.5992e+02	2.5686e-03	4.4089e+02	2.5139e-03	4.1548e+02	3.1755e-03	4.5299e+02
2.7972e-03	4.7567e+02	2.3716e-03	3.8250e+02	2.3211e-03	4.2856e+02	2.9320e-03	3.9730e+02
2.5648e-03	5.0458e+02	2.1745e-03	4.3965e+02	2.1283e-03	3.7496e+02	2.6884e-03	3.5424e+02
2.3324e-03	3.3170e+02	1.9775e-03	3.8597e+02	1.9355e-03	4.7190e+02	2.4448e-03	4.5634e+02
2.1001e-03	4.2956e+02	1.7805e-03	5.9581e+02	1.7426e-03	4.5760e+02	2.2013e-03	5.0067e+02
1.8677e-03	3.8261e+02	1.5835e-03	5.0226e+02	1.5498e-03	4.6026e+02	1.9577e-03	4.1792e+02
1.6353e-03	4.7006e+02	1.3865e-03	4.3345e+02	1.3570e-03	5.2709e+02	1.7141e-03	4.9155e+02
1.4030e-03	4.5418e+02	1.1895e-03	5.8127e+02	1.1642e-03	5.4058e+02	1.4706e-03	4.9345e+02
1.1706e-03	5.3117e+02	9.9247e-04	5.1952e+02	9.7135e-04	5.7439e+02	1.2270e-03	6.5116e+02
9.3821e-04	6.0228e+02	7.9546e-04	6.1408e+02	7.7853e-04	6.3844e+02	9.8343e-04	5.8646e+02
7.0584e-04	5.2088e+02	5.9845e-04	6.9521e+02	5.8571e-04	7.8853e+02	7.3986e-04	6.8014e+02
4.7347e-04	6.8585e+02	4.0143e-04	7.9291e+02	3.9289e-04	8.0077e+02	4.9629e-04	8.5684e+02
2.4111e-04	8.4405e+02	2.0442e-04	9.4351e+02	2.0007e-04	1.0348e+03	2.5272e-04	9.6124e+02
8.7357e-06	1.0560e+03	7.4065e-06	1.1678e+03	7.2489e-06	1.1385e+03	9.1567e-06	1.1749e+03

FREQUENCY [Hz]: 21578		22768		23253		25751	
TAU [s]	MAGNITUDES [a.u]	TAU [s]	MAGNITUDES [a.u]	TAU [s]	MAGNITUDES [a.u]	TAU [s]	MAGNITUDES [a.u]
2.6277e-03	5.2305e+02	4.1393e-03	7.0774e+02	3.2909e-03	4.5315e+02	3.7010e-03	4.1429e+02
2.4530e-03	5.2569e+02	3.8640e-03	5.9243e+02	3.0721e-03	4.2345e+02	3.4548e-03	4.6546e+02
2.2782e-03	5.6773e+02	3.5888e-03	6.5363e+02	2.8532e-03	4.3469e+02	3.2087e-03	4.8533e+02
2.1035e-03	4.8167e+02	3.3135e-03	6.7942e+02	2.6344e-03	4.6180e+02	2.9626e-03	4.0946e+02
1.9288e-03	5.3904e+02	3.0382e-03	6.0415e+02	2.4155e-03	4.8394e+02	2.7165e-03	4.5203e+02
1.7540e-03	5.5048e+02	2.7630e-03	5.1981e+02	2.1967e-03	4.4696e+02	2.4704e-03	4.6525e+02
1.5793e-03	5.4813e+02	2.4877e-03	6.6326e+02	1.9778e-03	4.9499e+02	2.2243e-03	4.5137e+02
1.4045e-03	5.6966e+02	2.2124e-03	6.2299e+02	1.7590e-03	5.2727e+02	1.9782e-03	4.6109e+02
1.2298e-03	6.6024e+02	1.9372e-03	6.8193e+02	1.5401e-03	5.4290e+02	1.7320e-03	5.7260e+02
1.0550e-03	5.5099e+02	1.6619e-03	5.8433e+02	1.3213e-03	5.9800e+02	1.4859e-03	5.0731e+02
8.8029e-04	6.3809e+02	1.3867e-03	6.5401e+02	1.1025e-03	5.6865e+02	1.2398e-03	5.6396e+02
7.0555e-04	6.3484e+02	1.1114e-03	7.1496e+02	8.8361e-04	6.8270e+02	9.9371e-04	6.3011e+02
5.3080e-04	6.3218e+02	8.3614e-04	8.0027e+02	6.6476e-04	7.5389e+02	7.4759e-04	7.5783e+02
3.5606e-04	6.9130e+02	5.6087e-04	7.8358e+02	4.4592e-04	9.2437e+02	5.0148e-04	8.8822e+02
1.8131e-04	7.5554e+02	2.8561e-04	8.8316e+02	2.2707e-04	1.1140e+03	2.5537e-04	9.6975e+02
6.5693e-06	8.3737e+02	1.0348e-05	9.5410e+02	8.2273e-06	1.1129e+03	9.2524e-06	1.1577e+03

FREQUENCY [Hz]: 28378		31131		31697		34308	
TAU [s]	MAGNITUDES [a.u]	TAU [s]	MAGNITUDES [a.u]	TAU [s]	MAGNITUDES [a.u]	TAU [s]	MAGNITUDES [a.u]
4.4515e-03	4.5262e+02	4.6353e-03	3.8329e+02	2.3956e-03	6.3688e+02	4.0194e-03	4.3563e+02
4.1554e-03	4.8596e+02	4.3271e-03	3.6482e+02	2.2363e-03	5.7293e+02	3.7521e-03	4.2496e+02
3.8594e-03	4.3725e+02	4.0188e-03	4.6952e+02	2.0770e-03	6.7253e+02	3.4848e-03	4.7595e+02
3.5634e-03	4.2378e+02	3.7106e-03	5.1798e+02	1.9177e-03	5.9496e+02	3.2175e-03	3.8119e+02
3.2674e-03	4.1631e+02	3.4023e-03	5.0819e+02	1.7584e-03	6.2804e+02	2.9502e-03	4.7928e+02
2.9714e-03	4.3622e+02	3.0941e-03	4.7366e+02	1.5990e-03	7.0637e+02	2.6830e-03	5.1501e+02
2.6753e-03	3.6568e+02	2.7858e-03	5.2655e+02	1.4397e-03	5.7506e+02	2.4157e-03	5.9705e+02
2.3793e-03	5.1865e+02	2.4776e-03	4.6056e+02	1.2804e-03	6.3713e+02	2.1484e-03	4.9592e+02
2.0833e-03	4.4307e+02	2.1693e-03	4.6364e+02	1.1211e-03	7.1370e+02	1.8811e-03	4.8705e+02
1.7873e-03	5.2404e+02	1.8611e-03	4.9456e+02	9.6182e-04	7.2904e+02	1.6138e-03	5.4164e+02
1.4912e-03	6.3029e+02	1.5528e-03	5.8881e+02	8.0252e-04	7.0044e+02	1.3465e-03	6.8666e+02
1.1952e-03	7.2863e+02	1.2446e-03	6.7340e+02	6.4321e-04	7.2557e+02	1.0792e-03	7.9090e+02
8.9920e-04	7.3797e+02	9.3634e-04	8.5684e+02	4.8391e-04	8.5062e+02	8.1192e-04	9.0628e+02
6.0317e-04	8.3901e+02	6.2809e-04	9.5037e+02	3.2460e-04	9.3789e+02	5.4463e-04	9.1251e+02
3.0715e-04	9.5018e+02	3.1984e-04	9.8231e+02	1.6529e-04	9.2523e+02	2.7734e-04	1.1892e+03
1.1129e-05	1.2617e+03	1.1588e-05	1.2301e+03	5.9889e-06	9.0897e+02	1.0049e-05	1.2474e+03

FREQUENCY [Hz]: 35802		37946		41771		44088	
TAU [s]	MAGNITUDES [a.u]	TAU [s]	MAGNITUDES [a.u]	TAU [s]	MAGNITUDES [a.u]	TAU [s]	MAGNITUDES [a.u]
4.6823e-03	5.0758e+02	5.2472e-03	3.6756e+02	4.7459e-03	4.2906e+02	4.0940e-03	5.8586e+02
4.3709e-03	4.3517e+02	4.8982e-03	4.6218e+02	4.4303e-03	4.0291e+02	3.8218e-03	6.7718e+02
4.0596e-03	4.5739e+02	4.5493e-03	4.4999e+02	4.1147e-03	4.3450e+02	3.5495e-03	6.2465e+02
3.7482e-03	6.0362e+02	4.2003e-03	4.4816e+02	3.7991e-03	4.8487e+02	3.2773e-03	7.1827e+02
3.4368e-03	5.3438e+02	3.8514e-03	4.9027e+02	3.4835e-03	4.8580e+02	3.0050e-03	6.6343e+02
3.1254e-03	4.9104e+02	3.5025e-03	4.5457e+02	3.1679e-03	5.2160e+02	2.7328e-03	6.2507e+02
2.8141e-03	5.4212e+02	3.1535e-03	4.5573e+02	2.8523e-03	5.1548e+02	2.4605e-03	7.6545e+02
2.5027e-03	6.6311e+02	2.8046e-03	4.7127e+02	2.5367e-03	4.8771e+02	2.1883e-03	6.7968e+02
2.1913e-03	4.9395e+02	2.4557e-03	6.0207e+02	2.2211e-03	5.6138e+02	1.9160e-03	7.5701e+02
1.8799e-03	6.1787e+02	2.1067e-03	5.2637e+02	1.9055e-03	6.1504e+02	1.6438e-03	6.9863e+02
1.5686e-03	5.5609e+02	1.7578e-03	6.5837e+02	1.5899e-03	6.2810e+02	1.3715e-03	8.0591e+02
1.2572e-03	7.0228e+02	1.4089e-03	6.2683e+02	1.2743e-03	7.7139e+02	1.0992e-03	6.9751e+02
9.4583e-04	6.5356e+02	1.0599e-03	7.1044e+02	9.5868e-04	8.5189e+02	8.2699e-04	8.2910e+02
6.3445e-04	6.8201e+02	7.1099e-04	9.3187e+02	6.4308e-04	1.0390e+03	5.5474e-04	9.3432e+02
3.2308e-04	7.4368e+02	3.6205e-04	1.1001e+03	3.2747e-04	1.0926e+03	2.8249e-04	9.8379e+02
1.1706e-05	9.4504e+02	1.3118e-05	1.3778e+03	1.1865e-05	1.3130e+03	1.0235e-05	9.9042e+02

FREQUENCY [Hz]: 46123		50689		55847		60075	
TAU [s]	MAGNITUDES [a.u]	TAU [s]	MAGNITUDES [a.u]	TAU [s]	MAGNITUDES [a.u]	TAU [s]	MAGNITUDES [a.u]
5.4324e-03	5.0857e+02	5.6408e-03	4.8040e+02	5.2317e-03	4.4671e+02	5.7939e-03	4.2319e+02
5.0711e-03	4.9622e+02	5.2657e-03	5.6122e+02	4.8838e-03	4.7611e+02	5.4086e-03	4.0061e+02
4.7099e-03	3.9260e+02	4.8906e-03	4.3476e+02	4.5358e-03	4.4926e+02	5.0233e-03	5.0997e+02
4.3486e-03	4.5978e+02	4.5154e-03	4.5806e+02	4.1879e-03	4.4034e+02	4.6380e-03	5.1747e+02
3.9874e-03	4.9489e+02	4.1403e-03	4.6503e+02	3.8400e-03	4.1409e+02	4.2527e-03	6.0391e+02
3.6261e-03	4.9438e+02	3.7652e-03	4.5707e+02	3.4921e-03	4.9556e+02	3.8674e-03	4.8542e+02
3.2649e-03	4.9474e+02	3.3901e-03	5.7211e+02	3.1442e-03	4.1374e+02	3.4821e-03	5.7103e+02
2.9036e-03	5.4505e+02	3.0150e-03	5.2151e+02	2.7963e-03	5.4870e+02	3.0968e-03	4.6318e+02
2.5424e-03	5.3352e+02	2.6399e-03	6.0756e+02	2.4484e-03	6.3584e+02	2.7115e-03	4.4687e+02
2.1811e-03	5.8451e+02	2.2648e-03	6.2247e+02	2.1005e-03	6.3748e+02	2.3263e-03	5.8060e+02
1.8199e-03	6.7481e+02	1.8897e-03	6.6465e+02	1.7526e-03	6.7012e+02	1.9410e-03	5.8626e+02
1.4586e-03	6.2999e+02	1.5145e-03	8.3461e+02	1.4047e-03	8.0645e+02	1.5557e-03	6.9112e+02
1.0973e-03	8.3214e+02	1.1394e-03	8.3280e+02	1.0568e-03	9.3258e+02	1.1704e-03	7.3962e+02
7.3609e-04	1.0297e+03	7.6433e-04	1.0557e+03	7.0889e-04	1.0234e+03	7.8507e-04	7.8539e+02
3.7484e-04	1.2056e+03	3.8921e-04	1.1112e+03	3.6098e-04	1.3137e+03	3.9978e-04	8.1997e+02
1.3581e-05	1.3434e+03	1.4102e-05	1.4174e+03	1.3079e-05	1.4282e+03	1.4485e-05	1.0047e+03

FREQUENCY [Hz]: 67741		74554		76306		82149	
TAU [s]	MAGNITUDES [a.u]	TAU [s]	MAGNITUDES [a.u]	TAU [s]	MAGNITUDES [a.u]	TAU [s]	MAGNITUDES [a.u]
6.0070e-03	4.7861e+02	6.9812e-03	4.1923e+02	5.8815e-03	6.1606e+02	6.3472e-03	4.0703e+02
5.6075e-03	4.6164e+02	6.5170e-03	3.4257e+02	5.4904e-03	6.4483e+02	5.9251e-03	5.6944e+02
5.2080e-03	4.8937e+02	6.0527e-03	4.0850e+02	5.0993e-03	6.9287e+02	5.5030e-03	4.8687e+02
4.8086e-03	5.1142e+02	5.5885e-03	4.5335e+02	4.7082e-03	6.5335e+02	5.0809e-03	5.2324e+02
4.4091e-03	4.1885e+02	5.1242e-03	4.6451e+02	4.3170e-03	6.5053e+02	4.6588e-03	4.5810e+02
4.0096e-03	5.0603e+02	4.6600e-03	4.6755e+02	3.9259e-03	7.2040e+02	4.2368e-03	4.9996e+02
3.6102e-03	6.1791e+02	4.1957e-03	4.8278e+02	3.5348e-03	6.6184e+02	3.8147e-03	5.3155e+02
3.2107e-03	5.9192e+02	3.7315e-03	4.5620e+02	3.1437e-03	7.8948e+02	3.3926e-03	5.5919e+02
2.8113e-03	5.5815e+02	3.2672e-03	5.1859e+02	2.7525e-03	7.6019e+02	2.9705e-03	6.1595e+02
2.4118e-03	6.8047e+02	2.8030e-03	5.6880e+02	2.3614e-03	7.9238e+02	2.5484e-03	6.8994e+02
2.0123e-03	7.6455e+02	2.3387e-03	5.6895e+02	1.9703e-03	9.1213e+02	2.1263e-03	7.4625e+02
1.6129e-03	8.3611e+02	1.8745e-03	7.0525e+02	1.5792e-03	8.2449e+02	1.7042e-03	9.0034e+02
1.2134e-03	9.3100e+02	1.4102e-03	9.7441e+02	1.1881e-03	8.7818e+02	1.2821e-03	9.8301e+02
8.1394e-04	1.0158e+03	9.4596e-04	1.0687e+03	7.9695e-04	9.8534e+02	8.6004e-04	1.1719e+03
4.1448e-04	1.2571e+03	4.8171e-04	1.2330e+03	4.0582e-04	1.0159e+03	4.3796e-04	1.3946e+03
1.5017e-05	1.5215e+03	1.7453e-05	1.5725e+03	1.4704e-05	1.2471e+03	1.5868e-05	1.5247e+03

FREQUENCY [Hz]: 84918		90594		99956		105070	
TAU [s]	MAGNITUDES [a.u]	TAU [s]	MAGNITUDES [a.u]	TAU [s]	MAGNITUDES [a.u]	TAU [s]	MAGNITUDES [a.u]
5.5199e-03	5.7955e+02	7.4436e-03	5.0508e+02	1.0000e-02	4.0513e+02	7.3703e-03	5.1776e+02
5.1528e-03	6.7334e+02	6.9486e-03	5.1849e+02	9.3350e-03	3.7236e+02	6.8802e-03	6.1161e+02
4.7857e-03	6.0066e+02	6.4536e-03	4.2140e+02	8.6700e-03	4.3875e+02	6.3900e-03	5.5292e+02
4.4186e-03	7.1764e+02	5.9586e-03	4.7704e+02	8.0050e-03	5.0182e+02	5.8999e-03	5.5662e+02
4.0516e-03	6.6042e+02	5.4636e-03	5.9847e+02	7.3400e-03	4.7270e+02	5.4098e-03	5.6450e+02
3.6845e-03	5.8549e+02	4.9686e-03	4.5735e+02	6.6750e-03	4.4585e+02	4.9197e-03	6.2005e+02
3.3174e-03	7.6100e+02	4.4736e-03	5.4533e+02	6.0100e-03	5.7148e+02	4.4295e-03	5.5509e+02
2.9504e-03	7.1148e+02	3.9786e-03	5.0793e+02	5.3450e-03	5.5423e+02	3.9394e-03	5.8213e+02
2.5833e-03	7.2429e+02	3.4836e-03	5.0879e+02	4.6800e-03	5.5894e+02	3.4493e-03	5.3286e+02
2.2162e-03	7.7919e+02	2.9886e-03	6.5063e+02	4.0150e-03	5.5884e+02	2.9592e-03	6.6765e+02
1.8492e-03	8.0760e+02	2.4936e-03	7.7306e+02	3.3500e-03	6.2158e+02	2.4690e-03	7.9176e+02
1.4821e-03	9.7416e+02	1.9986e-03	8.0925e+02	2.6850e-03	6.9624e+02	1.9789e-03	7.7703e+02
1.1150e-03	9.1359e+02	1.5036e-03	9.2671e+02	2.0200e-03	8.7444e+02	1.4888e-03	8.6938e+02
7.4794e-04	1.0053e+03	1.0086e-03	1.1306e+03	1.3550e-03	1.0031e+03	9.9867e-04	9.4102e+02
3.8087e-04	1.1481e+03	5.1361e-04	1.3722e+03	6.9000e-04	1.2485e+03	5.0855e-04	9.4016e+02
1.3800e-05	1.1722e+03	1.8609e-05	1.6476e+03	2.5000e-05	1.6195e+03	1.8426e-05	1.0918e+03

FREQUENCY [Hz]: 107150		115130		120610		126220	
TAU [s]	MAGNITUDES [a.u]	TAU [s]	MAGNITUDES [a.u]	TAU [s]	MAGNITUDES [a.u]	TAU [s]	MAGNITUDES [a.u]
9.3738e-03	5.9939e+02	9.0546e-03	4.7400e+02	9.6925e-03	4.6929e+02	8.4915e-03	4.9762e+02
8.7504e-03	6.6697e+02	8.4525e-03	4.3200e+02	9.0479e-03	5.2579e+02	7.9268e-03	5.2830e+02
8.1271e-03	6.5423e+02	7.8504e-03	4.6881e+02	8.4034e-03	5.1874e+02	7.3621e-03	5.6216e+02
7.5037e-03	6.7039e+02	7.2482e-03	5.3798e+02	7.7588e-03	5.2858e+02	6.7974e-03	5.3192e+02
6.8804e-03	6.8420e+02	6.6461e-03	5.6228e+02	7.1143e-03	4.5299e+02	6.2328e-03	4.9127e+02
6.2570e-03	7.3786e+02	6.0440e-03	5.4233e+02	6.4697e-03	4.5707e+02	5.6681e-03	5.5915e+02
5.6336e-03	7.0843e+02	5.4418e-03	5.0236e+02	5.8252e-03	4.4186e+02	5.1034e-03	6.5044e+02
5.0103e-03	6.6387e+02	4.8397e-03	6.1904e+02	5.1806e-03	5.8535e+02	4.5387e-03	6.3454e+02
4.3869e-03	7.4400e+02	4.2376e-03	4.7328e+02	4.5361e-03	5.1131e+02	3.9740e-03	5.5181e+02
3.7636e-03	8.2822e+02	3.6354e-03	6.5363e+02	3.8915e-03	5.4643e+02	3.4093e-03	5.8558e+02
3.1402e-03	8.0150e+02	3.0333e-03	5.7941e+02	3.2470e-03	6.2956e+02	2.8447e-03	6.5121e+02
2.5169e-03	7.6873e+02	2.4312e-03	6.5418e+02	2.6024e-03	7.1368e+02	2.2800e-03	7.4098e+02
1.8935e-03	8.1608e+02	1.8290e-03	8.0967e+02	1.9579e-03	7.4957e+02	1.7153e-03	7.9230e+02
1.2701e-03	9.7481e+02	1.2269e-03	8.1142e+02	1.3133e-03	7.4842e+02	1.1506e-03	8.5259e+02
6.4679e-04	1.1120e+03	6.2477e-04	8.6247e+02	6.6878e-04	9.2311e+02	5.8591e-04	9.9632e+02
2.3434e-05	1.1980e+03	2.2637e-05	1.0599e+03	2.4231e-05	1.0523e+03	2.1229e-05	1.0314e+03

FREQUENCY [Hz]: 126790		132540		139010		143820	
TAU [s]	MAGNITUDES [a.u]	TAU [s]	MAGNITUDES [a.u]	TAU [s]	MAGNITUDES [a.u]	TAU [s]	MAGNITUDES [a.u]
8.9755e-03	6.4217e+02	9.2269e-03	4.9420e+02	1.0389e-02	4.8618e+02	9.2359e-03	6.1093e+02
8.3787e-03	6.1849e+02	8.6133e-03	5.0625e+02	9.6978e-03	4.2302e+02	8.6217e-03	7.3374e+02
7.7818e-03	6.0520e+02	7.9997e-03	5.0456e+02	9.0070e-03	4.3910e+02	8.0075e-03	7.1134e+02
7.1849e-03	6.9612e+02	7.3861e-03	4.4446e+02	8.3161e-03	4.3746e+02	7.3933e-03	7.9276e+02
6.5880e-03	6.9004e+02	6.7725e-03	4.1545e+02	7.6253e-03	4.6503e+02	6.7791e-03	6.4266e+02
5.9912e-03	6.6184e+02	6.1589e-03	4.5957e+02	6.9344e-03	4.8136e+02	6.1650e-03	6.4137e+02
5.3943e-03	6.6818e+02	5.5453e-03	5.0006e+02	6.2436e-03	5.2409e+02	5.5508e-03	6.9418e+02
4.7974e-03	7.3064e+02	4.9318e-03	5.2642e+02	5.5527e-03	5.3549e+02	4.9366e-03	6.9819e+02
4.2005e-03	8.0845e+02	4.3182e-03	6.1433e+02	4.8619e-03	5.5746e+02	4.3224e-03	7.5425e+02
3.6037e-03	7.8346e+02	3.7046e-03	5.6721e+02	4.1711e-03	5.7561e+02	3.7082e-03	7.4743e+02
3.0068e-03	7.9775e+02	3.0910e-03	6.0027e+02	3.4802e-03	5.6158e+02	3.0940e-03	9.0443e+02
2.4099e-03	8.1900e+02	2.4774e-03	6.2808e+02	2.7894e-03	6.3245e+02	2.4798e-03	7.6929e+02
1.8131e-03	7.5768e+02	1.8638e-03	7.5747e+02	2.0985e-03	7.1896e+02	1.8656e-03	1.0601e+03
1.2162e-03	9.4813e+02	1.2502e-03	7.6741e+02	1.4077e-03	8.1137e+02	1.2515e-03	1.0450e+03
6.1931e-04	1.1658e+03	6.3665e-04	8.8113e+02	7.1682e-04	9.3215e+02	6.3728e-04	1.0482e+03
2.2439e-05	1.1560e+03	2.3067e-05	1.0743e+03	2.5972e-05	1.1217e+03	2.3090e-05	1.1901e+03

FREQUENCY [Hz]: 145640		149930		152420		162550	
TAU [s]	MAGNITUDES [a.u]	TAU [s]	MAGNITUDES [a.u]	TAU [s]	MAGNITUDES [a.u]	TAU [s]	MAGNITUDES [a.u]
1.0270e-02	4.6877e+02	7.8745e-03	5.9786e+02	1.5049e-02	4.3271e+02	8.3297e-03	6.7869e+02
9.5870e-03	4.5428e+02	7.3509e-03	7.2008e+02	1.4048e-02	4.2650e+02	7.7757e-03	7.6091e+02
8.9041e-03	4.4959e+02	6.8272e-03	6.2213e+02	1.3048e-02	5.2017e+02	7.2218e-03	6.6313e+02
8.2211e-03	4.8864e+02	6.3035e-03	6.9157e+02	1.2047e-02	4.9742e+02	6.6679e-03	8.1731e+02
7.5382e-03	4.2974e+02	5.7799e-03	6.9032e+02	1.1046e-02	4.1874e+02	6.1140e-03	6.7323e+02
6.8552e-03	5.3458e+02	5.2562e-03	6.9653e+02	1.0045e-02	4.3790e+02	5.5601e-03	8.1298e+02
6.1722e-03	4.7762e+02	4.7326e-03	7.0165e+02	9.0445e-03	4.2779e+02	5.0061e-03	7.9983e+02
5.4893e-03	4.8703e+02	4.2089e-03	6.8303e+02	8.0437e-03	4.4221e+02	4.4522e-03	7.3811e+02
4.8063e-03	5.6358e+02	3.6853e-03	6.6993e+02	7.0430e-03	4.6078e+02	3.8983e-03	7.5159e+02
4.1234e-03	6.2826e+02	3.1616e-03	7.5029e+02	6.0422e-03	5.0492e+02	3.3444e-03	8.8095e+02
3.4404e-03	6.2711e+02	2.6380e-03	8.2765e+02	5.0414e-03	6.2409e+02	2.7904e-03	8.8029e+02
2.7575e-03	6.5086e+02	2.1143e-03	9.1013e+02	4.0407e-03	5.9665e+02	2.2365e-03	8.4941e+02
2.0745e-03	7.8197e+02	1.5907e-03	9.3580e+02	3.0399e-03	6.4982e+02	1.6826e-03	9.5312e+02
1.3916e-03	8.2227e+02	1.0670e-03	9.8769e+02	2.0392e-03	7.3781e+02	1.1287e-03	1.0427e+03
7.0863e-04	9.4012e+02	5.4334e-04	1.0839e+03	1.0384e-03	8.9761e+02	5.7475e-04	1.2443e+03
2.5675e-05	1.1381e+03	1.9686e-05	1.2122e+03	3.7623e-05	1.1384e+03	2.0824e-05	1.2034e+03

FREQUENCY [Hz]: 167750		175690		184490		192820	
TAU [s]	MAGNITUDES [a.u]	TAU [s]	MAGNITUDES [a.u]	TAU [s]	MAGNITUDES [a.u]	TAU [s]	MAGNITUDES [a.u]
1.0497e-02	4.4244e+02	1.0463e-02	5.2101e+02	1.1907e-02	5.0615e+02	1.0215e-02	4.0043e+02
9.7988e-03	4.5295e+02	9.7668e-03	5.1379e+02	1.1115e-02	5.1514e+02	9.5359e-03	4.6315e+02
9.1008e-03	5.1734e+02	9.0711e-03	4.8984e+02	1.0324e-02	4.6824e+02	8.8566e-03	5.0769e+02
8.4027e-03	4.6512e+02	8.3753e-03	4.8150e+02	9.5318e-03	5.1451e+02	8.1773e-03	5.0582e+02
7.7047e-03	5.2235e+02	7.6796e-03	4.8092e+02	8.7400e-03	4.6612e+02	7.4980e-03	5.1429e+02
7.0066e-03	5.1156e+02	6.9838e-03	5.6661e+02	7.9481e-03	5.6355e+02	6.8187e-03	5.2537e+02
6.3086e-03	5.0623e+02	6.2880e-03	5.5886e+02	7.1563e-03	4.3539e+02	6.1394e-03	4.8927e+02
5.6106e-03	5.5576e+02	5.5923e-03	5.6707e+02	6.3645e-03	5.1590e+02	5.4600e-03	5.3561e+02
4.9125e-03	6.1336e+02	4.8965e-03	6.7698e+02	5.5726e-03	5.4678e+02	4.7807e-03	5.2104e+02
4.2145e-03	5.8994e+02	4.2007e-03	6.2900e+02	4.7808e-03	5.9765e+02	4.1014e-03	6.4212e+02
3.5164e-03	6.5309e+02	3.5050e-03	5.8878e+02	3.9889e-03	6.3075e+02	3.4221e-03	7.2406e+02
2.8184e-03	6.9327e+02	2.8092e-03	6.9445e+02	3.1971e-03	6.9705e+02	2.7428e-03	7.3774e+02
2.1204e-03	7.5117e+02	2.1134e-03	7.9211e+02	2.4053e-03	8.3789e+02	2.0635e-03	7.7765e+02
1.4223e-03	9.4132e+02	1.4177e-03	7.9089e+02	1.6134e-03	7.9672e+02	1.3842e-03	8.2417e+02
7.2428e-04	9.5315e+02	7.2192e-04	9.5244e+02	8.2160e-04	9.8462e+02	7.0485e-04	1.0283e+03
2.6242e-05	1.1622e+03	2.6157e-05	1.0997e+03	2.9768e-05	1.1837e+03	2.5538e-05	1.1549e+03

FREQUENCY [Hz]: 202040		212210		222630		244230	
TAU [s]	MAGNITUDES [a.u]	TAU [s]	MAGNITUDES [a.u]	TAU [s]	MAGNITUDES [a.u]	TAU [s]	MAGNITUDES [a.u]
1.1384e-02	4.9068e+02	1.1126e-02	4.9963e+02	1.4182e-02	4.3450e+02	1.1619e-02	5.4776e+02
1.0627e-02	4.7630e+02	1.0386e-02	4.4410e+02	1.3239e-02	4.6617e+02	1.0846e-02	4.9198e+02
9.8703e-03	5.1651e+02	9.6461e-03	4.2455e+02	1.2296e-02	4.3987e+02	1.0073e-02	5.8570e+02
9.1132e-03	4.9501e+02	8.9062e-03	4.5783e+02	1.1353e-02	4.7328e+02	9.3007e-03	5.6662e+02
8.3561e-03	4.9494e+02	8.1663e-03	5.1699e+02	1.0410e-02	4.6154e+02	8.5280e-03	5.2789e+02
7.5991e-03	5.1111e+02	7.4265e-03	4.7375e+02	9.4666e-03	5.5348e+02	7.7554e-03	5.8115e+02
6.8420e-03	4.9667e+02	6.6866e-03	4.3668e+02	8.5234e-03	4.4046e+02	6.9828e-03	5.3029e+02
6.0850e-03	5.4809e+02	5.9467e-03	5.6345e+02	7.5803e-03	4.6583e+02	6.2101e-03	5.3823e+02
5.3279e-03	4.9507e+02	5.2069e-03	5.8107e+02	6.6372e-03	5.3778e+02	5.4375e-03	5.8059e+02
4.5708e-03	5.7051e+02	4.4670e-03	5.9569e+02	5.6941e-03	5.4488e+02	4.6649e-03	6.9516e+02
3.8138e-03	6.5907e+02	3.7271e-03	6.7571e+02	4.7510e-03	6.4185e+02	3.8922e-03	7.0459e+02
3.0567e-03	6.4716e+02	2.9873e-03	6.5257e+02	3.8079e-03	6.5025e+02	3.1196e-03	7.6487e+02
2.2996e-03	8.4074e+02	2.2474e-03	7.6276e+02	2.8648e-03	7.1938e+02	2.3470e-03	7.2374e+02
1.5426e-03	8.7903e+02	1.5075e-03	8.7850e+02	1.9217e-03	8.6188e+02	1.5743e-03	9.5669e+02
7.8552e-04	1.0405e+03	7.6768e-04	1.0397e+03	9.7856e-04	9.7785e+02	8.0168e-04	1.0735e+03
2.8461e-05	1.1859e+03	2.7815e-05	1.1635e+03	3.5455e-05	1.2546e+03	2.9046e-05	1.1492e+03

FREQUENCY [Hz]: 248990		256210		263540		281030	
TAU [s]	MAGNITUDES [a.u]	TAU [s]	MAGNITUDES [a.u]	TAU [s]	MAGNITUDES [a.u]	TAU [s]	MAGNITUDES [a.u]
1.3888e-02	6.4666e+02	1.7279e-02	5.1798e+02	2.2263e-02	7.4453e+02	1.2364e-02	6.0982e+02
1.2964e-02	6.7744e+02	1.6130e-02	5.0987e+02	2.0783e-02	7.6932e+02	1.1542e-02	5.5332e+02
1.2041e-02	7.2158e+02	1.4981e-02	6.0779e+02	1.9302e-02	7.7698e+02	1.0720e-02	5.3157e+02
1.1117e-02	6.1518e+02	1.3831e-02	5.4942e+02	1.7822e-02	6.9994e+02	9.8974e-03	5.5035e+02
1.0194e-02	7.0580e+02	1.2682e-02	5.1518e+02	1.6341e-02	6.9469e+02	9.0752e-03	5.6024e+02
9.2700e-03	7.8373e+02	1.1533e-02	5.8081e+02	1.4861e-02	6.9080e+02	8.2530e-03	5.3762e+02
8.3464e-03	7.4102e+02	1.0384e-02	5.8605e+02	1.3380e-02	7.8405e+02	7.4308e-03	5.9518e+02
7.4229e-03	7.4833e+02	9.2354e-03	5.6935e+02	1.1900e-02	7.2365e+02	6.6086e-03	6.4509e+02
6.4994e-03	7.5483e+02	8.0864e-03	6.0258e+02	1.0419e-02	7.6189e+02	5.7864e-03	5.8953e+02
5.5759e-03	7.7124e+02	6.9373e-03	5.3897e+02	8.9386e-03	6.9493e+02	4.9642e-03	6.8022e+02
4.6523e-03	8.4931e+02	5.7883e-03	6.0404e+02	7.4581e-03	7.5624e+02	4.1420e-03	7.8240e+02
3.7288e-03	8.5906e+02	4.6393e-03	6.6162e+02	5.9776e-03	8.3076e+02	3.3197e-03	8.0070e+02
2.8053e-03	9.0188e+02	3.4903e-03	8.3500e+02	4.4971e-03	1.0046e+03	2.4975e-03	8.8741e+02
1.8818e-03	9.8775e+02	2.3412e-03	7.6611e+02	3.0166e-03	9.0332e+02	1.6753e-03	1.0566e+03
9.5824e-04	1.1526e+03	1.1922e-03	1.1077e+03	1.5362e-03	1.1483e+03	8.5312e-04	1.0971e+03
3.4719e-05	1.2593e+03	4.3196e-05	1.2108e+03	5.5658e-05	1.4462e+03	3.0910e-05	1.2794e+03

FREQUENCY [Hz]: 294740		295610		335960		339670	
TAU [s]	MAGNITUDES [a.u]	TAU [s]	MAGNITUDES [a.u]	TAU [s]	MAGNITUDES [a.u]	TAU [s]	MAGNITUDES [a.u]
1.6839e-02	4.8501e+02	1.7501e-02	6.6390e+02	3.9107e-03	1.3155e+03	1.4181e-02	5.2509e+02
1.5719e-02	5.6883e+02	1.6337e-02	7.3756e+02	3.6506e-03	1.4613e+03	1.3238e-02	4.8571e+02
1.4599e-02	5.9751e+02	1.5173e-02	6.3144e+02	3.3906e-03	1.4050e+03	1.2295e-02	5.3077e+02
1.3480e-02	4.8953e+02	1.4009e-02	6.7513e+02	3.1305e-03	1.2800e+03	1.1352e-02	5.0352e+02
1.2360e-02	5.3563e+02	1.2845e-02	6.8232e+02	2.8705e-03	1.3009e+03	1.0409e-02	5.5956e+02
1.1240e-02	5.2531e+02	1.1682e-02	6.1607e+02	2.6104e-03	1.4550e+03	9.4661e-03	5.8311e+02
1.0120e-02	5.6425e+02	1.0518e-02	6.4069e+02	2.3503e-03	1.8584e+03	8.5230e-03	6.7445e+02
9.0004e-03	5.5714e+02	9.3541e-03	6.5723e+02	2.0903e-03	1.5261e+03	7.5800e-03	5.9285e+02
7.8806e-03	5.1251e+02	8.1903e-03	7.0548e+02	1.8302e-03	1.4149e+03	6.6369e-03	6.5861e+02
6.7608e-03	5.7157e+02	7.0265e-03	7.8848e+02	1.5702e-03	1.5241e+03	5.6938e-03	6.4154e+02
5.6410e-03	6.2070e+02	5.8627e-03	8.2811e+02	1.3101e-03	1.5243e+03	4.7508e-03	6.8311e+02
4.5213e-03	6.9180e+02	4.6989e-03	8.8229e+02	1.0500e-03	1.4155e+03	3.8077e-03	7.2108e+02
3.4015e-03	7.7116e+02	3.5351e-03	8.7342e+02	7.8996e-04	1.4484e+03	2.8646e-03	8.2054e+02
2.2817e-03	8.9412e+02	2.3713e-03	1.0004e+03	5.2990e-04	1.5696e+03	1.9216e-03	9.3140e+02
1.1619e-03	1.0642e+03	1.2075e-03	1.0345e+03	2.6984e-04	1.6179e+03	9.7852e-04	1.0897e+03
4.2097e-05	1.2223e+03	4.3752e-05	1.3561e+03	9.7768e-06	1.8513e+03	3.5454e-05	1.2339e+03

FREQUENCY [Hz]: 355680		373030		390790		429640	
TAU [s]	MAGNITUDES [a.u]	TAU [s]	MAGNITUDES [a.u]	TAU [s]	MAGNITUDES [a.u]	TAU [s]	MAGNITUDES [a.u]
1.5052e-02	5.1045e+02	1.6031e-02	5.0426e+02	1.3206e-02	4.4237e+02	1.7299e-02	6.0326e+02
1.4051e-02	4.7837e+02	1.4965e-02	5.3867e+02	1.2328e-02	5.5366e+02	1.6149e-02	4.7203e+02
1.3050e-02	4.6947e+02	1.3899e-02	4.5953e+02	1.1449e-02	5.7140e+02	1.4998e-02	5.1867e+02
1.2049e-02	4.9494e+02	1.2833e-02	5.3562e+02	1.0571e-02	5.3044e+02	1.3848e-02	5.3786e+02
1.1048e-02	5.3896e+02	1.1767e-02	4.4545e+02	9.6930e-03	5.3830e+02	1.2698e-02	5.4754e+02
1.0047e-02	5.3450e+02	1.0701e-02	4.8010e+02	8.8149e-03	6.1729e+02	1.1547e-02	5.0159e+02
9.0463e-03	5.5380e+02	9.6349e-03	6.0171e+02	7.9367e-03	5.3845e+02	1.0397e-02	5.3269e+02
8.0453e-03	5.5045e+02	8.5688e-03	5.3837e+02	7.0585e-03	6.9846e+02	9.2464e-03	5.5568e+02
7.0443e-03	5.5755e+02	7.5027e-03	6.1348e+02	6.1803e-03	6.5444e+02	8.0960e-03	6.6197e+02
6.0434e-03	5.3183e+02	6.4366e-03	6.4006e+02	5.3021e-03	6.4871e+02	6.9456e-03	6.2647e+02
5.0424e-03	7.2668e+02	5.3705e-03	6.2034e+02	4.4239e-03	7.5691e+02	5.7952e-03	8.0581e+02
4.0415e-03	7.0037e+02	4.3044e-03	7.6765e+02	3.5457e-03	7.3498e+02	4.6448e-03	7.6932e+02
3.0405e-03	8.7328e+02	3.2384e-03	8.3662e+02	2.6676e-03	9.4357e+02	3.4944e-03	7.9293e+02
2.0395e-03	9.0534e+02	2.1723e-03	8.6839e+02	1.7894e-03	9.9462e+02	2.3440e-03	9.5391e+02
1.0386e-03	1.0817e+03	1.1062e-03	1.0693e+03	9.1120e-04	1.1304e+03	1.1936e-03	1.1401e+03
3.7630e-05	1.2566e+03	4.0079e-05	1.2593e+03	3.3014e-05	1.2589e+03	4.3248e-05	1.2480e+03

FREQUENCY [Hz]: 449760		471430		490250		517450	
TAU [s]	MAGNITUDES [a.u]	TAU [s]	MAGNITUDES [a.u]	TAU [s]	MAGNITUDES [a.u]	TAU [s]	MAGNITUDES [a.u]
1.6371e-02	4.9058e+02	2.0314e-02	4.9261e+02	1.5680e-02	6.7315e+02	1.7388e-02	5.6918e+02
1.5282e-02	5.4417e+02	1.8963e-02	4.7158e+02	1.4637e-02	7.9615e+02	1.6232e-02	6.0599e+02
1.4193e-02	5.1597e+02	1.7612e-02	6.0862e+02	1.3595e-02	6.9084e+02	1.5075e-02	5.8699e+02
1.3105e-02	4.8072e+02	1.6261e-02	6.0808e+02	1.2552e-02	6.8579e+02	1.3919e-02	5.5316e+02
1.2016e-02	5.5234e+02	1.4910e-02	6.1862e+02	1.1509e-02	7.0186e+02	1.2763e-02	5.7473e+02
1.0927e-02	5.3941e+02	1.3560e-02	5.4715e+02	1.0467e-02	6.4582e+02	1.1607e-02	5.3982e+02
9.8388e-03	6.5217e+02	1.2209e-02	5.5624e+02	9.4238e-03	7.3988e+02	1.0450e-02	5.6670e+02
8.7501e-03	6.6305e+02	1.0858e-02	5.9744e+02	8.3811e-03	8.1651e+02	9.2939e-03	5.8401e+02
7.6615e-03	6.9258e+02	9.5069e-03	6.6479e+02	7.3383e-03	7.9519e+02	8.1376e-03	6.3985e+02
6.5728e-03	6.6893e+02	8.1561e-03	6.7752e+02	6.2956e-03	7.1358e+02	6.9813e-03	6.8722e+02
5.4842e-03	7.0049e+02	6.8052e-03	6.3867e+02	5.2529e-03	7.8585e+02	5.8250e-03	8.1723e+02
4.3955e-03	7.9246e+02	5.4543e-03	6.9962e+02	4.2101e-03	9.3129e+02	4.6687e-03	8.0787e+02
3.3069e-03	9.1146e+02	4.1034e-03	9.1547e+02	3.1674e-03	1.0178e+03	3.5124e-03	8.5068e+02
2.2182e-03	9.4183e+02	2.7525e-03	9.0128e+02	2.1247e-03	1.1148e+03	2.3561e-03	1.0270e+03
1.1296e-03	1.1034e+03	1.4017e-03	1.1011e+03	1.0819e-03	1.1582e+03	1.1998e-03	1.1570e+03
4.0927e-05	1.3584e+03	5.0785e-05	1.3285e+03	3.9200e-05	1.3274e+03	4.3470e-05	1.3827e+03

FREQUENCY [Hz]: 543030		569220		581330		596030	
TAU [s]	MAGNITUDES [a.u]	TAU [s]	MAGNITUDES [a.u]	TAU [s]	MAGNITUDES [a.u]	TAU [s]	MAGNITUDES [a.u]
1.6496e-02	5.8414e+02	1.7070e-02	5.2912e+02	2.3989e-02	6.3121e+02	1.9266e-02	5.2744e+02
1.5399e-02	5.2395e+02	1.5935e-02	5.4371e+02	2.2394e-02	7.4400e+02	1.7985e-02	5.8592e+02
1.4302e-02	5.3669e+02	1.4800e-02	5.4254e+02	2.0799e-02	6.7362e+02	1.6704e-02	5.0623e+02
1.3205e-02	5.5643e+02	1.3665e-02	6.3860e+02	1.9204e-02	6.5134e+02	1.5423e-02	5.1909e+02
1.2108e-02	5.7379e+02	1.2529e-02	4.7914e+02	1.7608e-02	6.8678e+02	1.4141e-02	4.8121e+02
1.1011e-02	6.4145e+02	1.1394e-02	5.1165e+02	1.6013e-02	6.7311e+02	1.2860e-02	5.3512e+02
9.9141e-03	5.9422e+02	1.0259e-02	5.7718e+02	1.4418e-02	6.2497e+02	1.1579e-02	6.4714e+02
8.8171e-03	6.3404e+02	9.1239e-03	5.8727e+02	1.2822e-02	6.8651e+02	1.0298e-02	5.3244e+02
7.7201e-03	6.0406e+02	7.9888e-03	6.7998e+02	1.1227e-02	7.4190e+02	9.0166e-03	5.3790e+02
6.6231e-03	6.5127e+02	6.8536e-03	6.9620e+02	9.6318e-03	6.8697e+02	7.7354e-03	6.7341e+02
5.5262e-03	7.5737e+02	5.7185e-03	7.6941e+02	8.0365e-03	6.4314e+02	6.4542e-03	6.6682e+02
4.4292e-03	9.1186e+02	4.5833e-03	8.0865e+02	6.4412e-03	8.8204e+02	5.1730e-03	7.0008e+02
3.3322e-03	9.2353e+02	3.4481e-03	8.4381e+02	4.8459e-03	9.0950e+02	3.8918e-03	8.0080e+02
2.2352e-03	9.2146e+02	2.3130e-03	9.5241e+02	3.2506e-03	9.9243e+02	2.6106e-03	9.0977e+02
1.1382e-03	1.2141e+03	1.1778e-03	1.1624e+03	1.6553e-03	1.2271e+03	1.3294e-03	1.0696e+03
4.1240e-05	1.3139e+03	4.2675e-05	1.3484e+03	5.9974e-05	1.4212e+03	4.8166e-05	1.1954e+03

FREQUENCY [Hz]: 615920		624730		655380		686770	
TAU [s]	MAGNITUDES [a.u]	TAU [s]	MAGNITUDES [a.u]	TAU [s]	MAGNITUDES [a.u]	TAU [s]	MAGNITUDES [a.u]
8.1814e-03	1.3703e+03	1.9017e-02	5.2854e+02	1.3992e-02	5.1794e+02	1.5433e-02	5.7447e+02
7.6373e-03	1.2932e+03	1.7752e-02	5.3928e+02	1.3062e-02	5.9809e+02	1.4407e-02	5.4870e+02
7.0932e-03	1.3711e+03	1.6488e-02	6.7961e+02	1.2131e-02	5.5238e+02	1.3381e-02	5.7744e+02
6.5492e-03	1.2843e+03	1.5223e-02	5.6341e+02	1.1201e-02	6.1343e+02	1.2354e-02	5.8064e+02
6.0051e-03	1.3679e+03	1.3959e-02	4.7438e+02	1.0270e-02	6.1891e+02	1.1328e-02	5.5094e+02
5.4611e-03	1.4546e+03	1.2694e-02	4.5936e+02	9.3397e-03	6.5645e+02	1.0302e-02	5.9906e+02
4.9170e-03	1.3807e+03	1.1429e-02	6.1878e+02	8.4093e-03	6.2199e+02	9.2753e-03	5.6660e+02
4.3729e-03	1.2214e+03	1.0165e-02	5.6625e+02	7.4788e-03	6.2370e+02	8.2490e-03	6.1442e+02
3.8289e-03	1.3566e+03	8.9000e-03	6.1193e+02	6.5483e-03	7.1250e+02	7.2227e-03	6.7981e+02
3.2848e-03	1.5401e+03	7.6354e-03	5.9851e+02	5.6178e-03	7.0602e+02	6.1964e-03	7.3962e+02
2.7408e-03	1.6042e+03	6.3707e-03	8.1250e+02	4.6874e-03	8.3985e+02	5.1701e-03	7.9258e+02
2.1967e-03	1.6280e+03	5.1061e-03	8.2682e+02	3.7569e-03	8.4913e+02	4.1438e-03	7.4017e+02
1.6526e-03	1.5038e+03	3.8415e-03	8.9042e+02	2.8264e-03	1.0234e+03	3.1175e-03	8.7472e+02
1.1086e-03	1.6218e+03	2.5768e-03	1.0130e+03	1.8959e-03	1.0590e+03	2.0912e-03	1.0060e+03
5.6451e-04	1.7327e+03	1.3122e-03	1.1058e+03	9.6545e-04	1.0434e+03	1.0649e-03	1.1676e+03
2.0453e-05	1.6698e+03	4.7543e-05	1.3116e+03	3.4980e-05	1.3290e+03	3.8583e-05	1.3766e+03

FREQUENCY [Hz]: 720250		754530		785350		791020	
TAU [s]	MAGNITUDES [a.u]	TAU [s]	MAGNITUDES [a.u]	TAU [s]	MAGNITUDES [a.u]	TAU [s]	MAGNITUDES [a.u]
1.7334e-02	5.4532e+02	2.3790e-02	7.0057e+02	2.6701e-02	1.2951e+03	2.6942e-02	5.5008e+02
1.6181e-02	6.0476e+02	2.2208e-02	6.1814e+02	2.4926e-02	1.2987e+03	2.5150e-02	5.2731e+02
1.5028e-02	6.2852e+02	2.0626e-02	6.1357e+02	2.3150e-02	1.5528e+03	2.3358e-02	5.6791e+02
1.3876e-02	6.0601e+02	1.9044e-02	6.6036e+02	2.1374e-02	1.2669e+03	2.1567e-02	5.3967e+02
1.2723e-02	6.6733e+02	1.7462e-02	6.3682e+02	1.9599e-02	1.1295e+03	1.9775e-02	5.9399e+02
1.1570e-02	5.8930e+02	1.5880e-02	6.1151e+02	1.7823e-02	1.3187e+03	1.7984e-02	5.4490e+02
1.0418e-02	7.1494e+02	1.4298e-02	6.3577e+02	1.6048e-02	1.2589e+03	1.6192e-02	5.8250e+02
9.2649e-03	6.6005e+02	1.2716e-02	6.0553e+02	1.4272e-02	1.2145e+03	1.4400e-02	6.6855e+02
8.1122e-03	6.0818e+02	1.1134e-02	6.3010e+02	1.2496e-02	1.3538e+03	1.2609e-02	6.6696e+02
6.9595e-03	6.7082e+02	9.5519e-03	6.9887e+02	1.0721e-02	1.2229e+03	1.0817e-02	5.4623e+02
5.8068e-03	7.8348e+02	7.9698e-03	7.2465e+02	8.9450e-03	1.3748e+03	9.0254e-03	7.7869e+02
4.6541e-03	9.6340e+02	6.3877e-03	8.7431e+02	7.1693e-03	1.4010e+03	7.2338e-03	8.7583e+02
3.5014e-03	9.2602e+02	4.8057e-03	9.0543e+02	5.3937e-03	1.3194e+03	5.4422e-03	8.8926e+02
2.3487e-03	1.0502e+03	3.2236e-03	1.0244e+03	3.6180e-03	1.3277e+03	3.6506e-03	1.0164e+03
1.1960e-03	1.1742e+03	1.6415e-03	1.1748e+03	1.8424e-03	1.5160e+03	1.8590e-03	1.2912e+03
4.3335e-05	1.4741e+03	5.9476e-05	1.4322e+03	6.6753e-05	1.7922e+03	6.7354e-05	1.4587e+03

FREQUENCY [Hz]: 815350		828380		868080		886030	
TAU [s]	MAGNITUDES [a.u]	TAU [s]	MAGNITUDES [a.u]	TAU [s]	MAGNITUDES [a.u]	TAU [s]	MAGNITUDES [a.u]
3.1177e-02	5.7286e+02	2.8483e-02	6.0885e+02	2.9481e-02	5.0820e+02	3.2919e-02	1.4328e+03
2.9103e-02	7.4862e+02	2.6589e-02	6.3237e+02	2.7521e-02	5.7593e+02	3.0730e-02	1.3983e+03
2.7030e-02	7.2456e+02	2.4695e-02	5.2797e+02	2.5560e-02	5.1475e+02	2.8540e-02	1.3431e+03
2.4957e-02	6.7199e+02	2.2801e-02	5.9263e+02	2.3600e-02	5.6814e+02	2.6351e-02	1.3411e+03
2.2884e-02	7.6204e+02	2.0907e-02	5.7313e+02	2.1639e-02	6.2837e+02	2.4162e-02	1.3573e+03
2.0810e-02	7.7597e+02	1.9013e-02	5.8462e+02	1.9679e-02	6.2784e+02	2.1973e-02	1.2621e+03
1.8737e-02	6.5896e+02	1.7118e-02	5.5795e+02	1.7718e-02	5.4180e+02	1.9784e-02	1.4928e+03
1.6664e-02	6.4323e+02	1.5224e-02	6.2640e+02	1.5758e-02	6.8467e+02	1.7595e-02	1.3458e+03
1.4591e-02	6.7783e+02	1.3330e-02	6.6564e+02	1.3797e-02	6.1381e+02	1.5406e-02	1.1694e+03
1.2517e-02	7.9190e+02	1.1436e-02	6.4979e+02	1.1837e-02	7.1820e+02	1.3217e-02	1.3292e+03
1.0444e-02	8.5233e+02	9.5419e-03	7.4141e+02	9.8763e-03	7.1225e+02	1.1028e-02	1.5989e+03
8.3709e-03	8.1676e+02	7.6478e-03	8.8327e+02	7.9157e-03	8.7397e+02	8.8387e-03	1.3639e+03
6.2977e-03	9.8421e+02	5.7536e-03	9.1974e+02	5.9552e-03	9.8758e+02	6.6496e-03	1.6326e+03
4.2244e-03	1.1075e+03	3.8595e-03	1.0771e+03	3.9947e-03	1.0084e+03	4.4605e-03	1.7224e+03
2.1512e-03	1.2619e+03	1.9653e-03	1.2535e+03	2.0342e-03	1.1873e+03	2.2714e-03	1.8262e+03
7.7941e-05	1.5568e+03	7.1208e-05	1.3778e+03	7.3703e-05	1.4638e+03	8.2297e-05	1.8645e+03

FREQUENCY [Hz]: 910240		953390		965910		1.0007E6	
TAU [s]	MAGNITUDES [a.u]	TAU [s]	MAGNITUDES [a.u]	TAU [s]	MAGNITUDES [a.u]	TAU [s]	MAGNITUDES [a.u]
3.2603e-02	5.9877e+02	3.6610e-02	6.6271e+02	4.2148e-02	7.0387e+02	4.0000e-02	1.0636e+03
3.0435e-02	6.1433e+02	3.4175e-02	6.1500e+02	3.9345e-02	6.9641e+02	3.7340e-02	1.3418e+03
2.8267e-02	6.0770e+02	3.1741e-02	5.8475e+02	3.6543e-02	6.8054e+02	3.4680e-02	1.3758e+03
2.6099e-02	7.0065e+02	2.9306e-02	5.8966e+02	3.3740e-02	6.7139e+02	3.2020e-02	1.3868e+03
2.3931e-02	5.8326e+02	2.6872e-02	6.6617e+02	3.0937e-02	6.9239e+02	2.9360e-02	1.3882e+03
2.1763e-02	5.8077e+02	2.4437e-02	6.0900e+02	2.8134e-02	7.7148e+02	2.6700e-02	1.4764e+03
1.9595e-02	6.1231e+02	2.2003e-02	6.6473e+02	2.5331e-02	7.1471e+02	2.4040e-02	1.2446e+03
1.7426e-02	7.0119e+02	1.9568e-02	6.9663e+02	2.2528e-02	6.7990e+02	2.1380e-02	1.2813e+03
1.5258e-02	7.4455e+02	1.7133e-02	7.0039e+02	1.9725e-02	8.2133e+02	1.8720e-02	1.2672e+03
1.3090e-02	7.7252e+02	1.4699e-02	6.4591e+02	1.6923e-02	7.2898e+02	1.6060e-02	1.2629e+03
1.0922e-02	7.8063e+02	1.2264e-02	7.2071e+02	1.4120e-02	8.6733e+02	1.3400e-02	1.4480e+03
8.7540e-03	8.3026e+02	9.8298e-03	8.9548e+02	1.1317e-02	7.8690e+02	1.0740e-02	1.4411e+03
6.5859e-03	9.8304e+02	7.3952e-03	1.0370e+03	8.5140e-03	1.0537e+03	8.0800e-03	1.6260e+03
4.4178e-03	1.1238e+03	4.9606e-03	1.1445e+03	5.7111e-03	1.0482e+03	5.4200e-03	1.6776e+03
2.2496e-03	1.2881e+03	2.5261e-03	1.2542e+03	2.9082e-03	1.2719e+03	2.7600e-03	1.7870e+03
8.1508e-05	1.5005e+03	9.1525e-05	1.4861e+03	1.0537e-04	1.4993e+03	1.0000e-04	1.9444e+03

FREQUENCY [Hz]: 1.1445E6		1.355E6		1.5373E6		1.5772E6	
TAU [s]	MAGNITUDES [a.u]	TAU [s]	MAGNITUDES [a.u]	TAU [s]	MAGNITUDES [a.u]	TAU [s]	MAGNITUDES [a.u]
4.8364e-02	6.4630e+02	5.2066e-02	6.4137e+02	4.1947e-02	6.6101e+02	3.9662e-02	5.6333e+02
4.5147e-02	6.1759e+02	4.8604e-02	6.0501e+02	3.9158e-02	5.9415e+02	3.7024e-02	6.5424e+02
4.1931e-02	6.9493e+02	4.5142e-02	6.7759e+02	3.6368e-02	6.2828e+02	3.4387e-02	6.3917e+02
3.8715e-02	6.3356e+02	4.1679e-02	7.2485e+02	3.3579e-02	6.2966e+02	3.1749e-02	6.3266e+02
3.5499e-02	7.2502e+02	3.8217e-02	6.4870e+02	3.0789e-02	6.6053e+02	2.9112e-02	5.6392e+02
3.2283e-02	7.5363e+02	3.4754e-02	7.4926e+02	2.8000e-02	5.8439e+02	2.6474e-02	6.3036e+02
2.9067e-02	6.6088e+02	3.1292e-02	7.4496e+02	2.5210e-02	7.1764e+02	2.3837e-02	7.2267e+02
2.5850e-02	7.5340e+02	2.7830e-02	7.0180e+02	2.2421e-02	7.3909e+02	2.1199e-02	6.1160e+02
2.2634e-02	7.8845e+02	2.4367e-02	8.8839e+02	1.9631e-02	7.5634e+02	1.8562e-02	6.8046e+02
1.9418e-02	7.4066e+02	2.0905e-02	7.3813e+02	1.6842e-02	7.3498e+02	1.5924e-02	7.9977e+02
1.6202e-02	8.8299e+02	1.7442e-02	8.8951e+02	1.4052e-02	8.0560e+02	1.3287e-02	8.4744e+02
1.2986e-02	9.1886e+02	1.3980e-02	9.9858e+02	1.1263e-02	9.2235e+02	1.0649e-02	9.5897e+02
9.7694e-03	9.8527e+02	1.0517e-02	1.0076e+03	8.4733e-03	9.9515e+02	8.0117e-03	1.0333e+03
6.5533e-03	1.1388e+03	7.0550e-03	1.1879e+03	5.6838e-03	1.1482e+03	5.3742e-03	1.0620e+03
3.3371e-03	1.2382e+03	3.5926e-03	1.3210e+03	2.8944e-03	1.2833e+03	2.7367e-03	1.2231e+03
1.2091e-04	1.5552e+03	1.3017e-04	1.6327e+03	1.0487e-04	1.4383e+03	9.9154e-05	1.4976e+03

FREQUENCY [Hz]: 1.6175E6		1.6399E6		1.7019E6		1.7883E6	
TAU [s]	MAGNITUDES [a.u]	TAU [s]	MAGNITUDES [a.u]	TAU [s]	MAGNITUDES [a.u]	TAU [s]	MAGNITUDES [a.u]
4.8876e-02	5.9500e+02	3.9942e-02	7.3459e+02	3.0855e-02	6.0219e+02	3.7808e-02	5.7413e+02
4.5625e-02	5.9263e+02	3.7285e-02	7.3375e+02	2.8803e-02	5.9200e+02	3.5294e-02	5.3923e+02
4.2375e-02	5.8286e+02	3.4629e-02	8.6350e+02	2.6751e-02	6.1489e+02	3.2779e-02	5.9510e+02
3.9125e-02	5.7468e+02	3.1973e-02	7.9223e+02	2.4700e-02	5.9482e+02	3.0265e-02	5.7958e+02
3.5875e-02	6.1306e+02	2.9317e-02	7.7153e+02	2.2648e-02	6.8581e+02	2.7751e-02	6.9774e+02
3.2624e-02	6.5877e+02	2.6661e-02	7.7629e+02	2.0596e-02	6.0773e+02	2.5237e-02	6.6795e+02
2.9374e-02	6.3788e+02	2.4005e-02	7.8317e+02	1.8544e-02	7.4707e+02	2.2722e-02	6.6835e+02
2.6124e-02	6.6903e+02	2.1349e-02	7.9604e+02	1.6492e-02	7.2623e+02	2.0208e-02	6.6387e+02
2.2874e-02	6.6738e+02	1.8693e-02	8.9273e+02	1.4440e-02	7.4742e+02	1.7694e-02	7.4282e+02
1.9624e-02	7.9575e+02	1.6037e-02	8.7748e+02	1.2388e-02	8.3581e+02	1.5180e-02	7.4309e+02
1.6373e-02	7.7326e+02	1.3380e-02	9.5465e+02	1.0336e-02	8.8726e+02	1.2666e-02	7.6969e+02
1.3123e-02	7.8939e+02	1.0724e-02	1.0776e+03	8.2846e-03	1.0188e+03	1.0151e-02	9.2662e+02
9.8729e-03	9.5613e+02	8.0682e-03	1.1557e+03	6.2327e-03	1.0594e+03	7.6372e-03	9.0052e+02
6.6226e-03	1.0631e+03	5.4121e-03	1.2865e+03	4.1809e-03	1.2031e+03	5.1230e-03	1.0964e+03
3.3724e-03	1.3084e+03	2.7560e-03	1.4296e+03	2.1290e-03	1.3327e+03	2.6087e-03	1.2650e+03
1.2219e-04	1.5466e+03	9.9854e-05	1.6115e+03	7.7138e-05	1.4859e+03	9.4519e-05	1.4403e+03

FREQUENCY [Hz]: 1.8119E6		1.8335E6		1.8813E6		1.9011E6	
TAU [s]	MAGNITUDES [a.u]	TAU [s]	MAGNITUDES [a.u]	TAU [s]	MAGNITUDES [a.u]	TAU [s]	MAGNITUDES [a.u]
2.4822e-02	6.9421e+02	3.6210e-02	5.5358e+02	3.4509e-02	6.3236e+02	2.0259e-02	9.3762e+02
2.3171e-02	9.3661e+02	3.3802e-02	5.0385e+02	3.2215e-02	5.0608e+02	1.8912e-02	7.9707e+02
2.1521e-02	1.0345e+03	3.1394e-02	5.0533e+02	2.9920e-02	5.5559e+02	1.7565e-02	7.9021e+02
1.9870e-02	8.7288e+02	2.8986e-02	6.1500e+02	2.7625e-02	5.9603e+02	1.6218e-02	8.4432e+02
1.8219e-02	8.9347e+02	2.6578e-02	6.5628e+02	2.5330e-02	6.3882e+02	1.4870e-02	8.3341e+02
1.6569e-02	8.8960e+02	2.4170e-02	5.7531e+02	2.3035e-02	6.6330e+02	1.3523e-02	8.6805e+02
1.4918e-02	1.0272e+03	2.1762e-02	6.4893e+02	2.0740e-02	6.8170e+02	1.2176e-02	9.3749e+02
1.3267e-02	9.8945e+02	1.9354e-02	6.8236e+02	1.8445e-02	5.7958e+02	1.0829e-02	9.6315e+02
1.1617e-02	1.0216e+03	1.6946e-02	6.5663e+02	1.6150e-02	7.3402e+02	9.4814e-03	9.8567e+02
9.9660e-03	1.1353e+03	1.4538e-02	7.2466e+02	1.3856e-02	7.4221e+02	8.1342e-03	1.0642e+03
8.3153e-03	1.0621e+03	1.2130e-02	7.6665e+02	1.1561e-02	8.2892e+02	6.7869e-03	1.1310e+03
6.6647e-03	1.2187e+03	9.7223e-03	9.0592e+02	9.2658e-03	9.6889e+02	5.4397e-03	1.1227e+03
5.0140e-03	1.3094e+03	7.3143e-03	9.0710e+02	6.9709e-03	1.0088e+03	4.0924e-03	1.1324e+03
3.3634e-03	1.4379e+03	4.9064e-03	1.0802e+03	4.6760e-03	1.2186e+03	2.7452e-03	1.2379e+03
1.7127e-03	1.5316e+03	2.4985e-03	1.1966e+03	2.3812e-03	1.3152e+03	1.3979e-03	1.4257e+03
6.2055e-05	1.6063e+03	9.0524e-05	1.4582e+03	8.6274e-05	1.5887e+03	5.0649e-05	1.4727e+03

FREQUENCY [Hz]: 1.9298E6		1.9789E6		1.9992E6		2.0287E6	
TAU [s]	MAGNITUDES [a.u]	TAU [s]	MAGNITUDES [a.u]	TAU [s]	MAGNITUDES [a.u]	TAU [s]	MAGNITUDES [a.u]
2.7688e-02	6.5919e+02	2.8430e-02	5.8741e+02	1.9196e-02	8.6809e+02	2.0861e-02	6.0673e+02
2.5846e-02	6.2643e+02	2.6539e-02	6.3947e+02	1.7919e-02	8.9416e+02	1.9473e-02	5.4909e+02
2.4005e-02	6.8523e+02	2.4649e-02	6.2931e+02	1.6643e-02	9.2546e+02	1.8086e-02	6.6724e+02
2.2164e-02	7.4241e+02	2.2758e-02	6.2169e+02	1.5366e-02	7.7821e+02	1.6699e-02	6.8965e+02
2.0323e-02	6.6467e+02	2.0867e-02	6.1303e+02	1.4090e-02	8.9374e+02	1.5312e-02	6.5798e+02
1.8482e-02	6.1157e+02	1.8977e-02	6.0790e+02	1.2813e-02	9.4886e+02	1.3924e-02	6.4839e+02
1.6640e-02	6.9485e+02	1.7086e-02	5.9395e+02	1.1537e-02	1.0322e+03	1.2537e-02	8.1660e+02
1.4799e-02	7.2097e+02	1.5196e-02	7.5446e+02	1.0260e-02	8.3723e+02	1.1150e-02	7.7296e+02
1.2958e-02	7.5346e+02	1.3305e-02	7.8760e+02	8.9835e-03	9.6775e+02	9.7628e-03	8.1004e+02
1.1117e-02	8.3154e+02	1.1415e-02	7.0841e+02	7.7070e-03	9.5253e+02	8.3755e-03	8.8393e+02
9.2754e-03	8.4414e+02	9.5240e-03	8.2062e+02	6.4305e-03	1.1515e+03	6.9883e-03	1.0256e+03
7.4342e-03	9.7813e+02	7.6334e-03	8.8494e+02	5.1540e-03	1.2141e+03	5.6011e-03	9.5291e+02
5.5929e-03	1.0855e+03	5.7428e-03	9.2423e+02	3.8775e-03	1.2333e+03	4.2138e-03	1.0373e+03
3.7517e-03	1.1698e+03	3.8522e-03	1.1445e+03	2.6010e-03	1.3847e+03	2.8266e-03	1.2169e+03
1.9105e-03	1.3212e+03	1.9617e-03	1.2907e+03	1.3245e-03	1.4842e+03	1.4394e-03	1.2864e+03
6.9219e-05	1.3757e+03	7.1075e-05	1.4622e+03	4.7989e-05	1.5588e+03	5.2152e-05	1.6014e+03

FREQUENCY [Hz]: 2.079E6		2.1183E6		2.2076E6		2.2505E6	
TAU [s]	MAGNITUDES [a.u]	TAU [s]	MAGNITUDES [a.u]	TAU [s]	MAGNITUDES [a.u]	TAU [s]	MAGNITUDES [a.u]
2.5450e-02	6.2652e+02	2.4270e-02	1.1878e+03	2.8525e-02	7.9880e+02	4.4087e-02	6.7191e+02
2.3757e-02	6.7510e+02	2.2656e-02	1.1411e+03	2.6628e-02	8.5840e+02	4.1155e-02	6.2702e+02
2.2065e-02	7.3648e+02	2.1042e-02	1.4409e+03	2.4731e-02	7.3240e+02	3.8223e-02	6.9038e+02
2.0373e-02	5.7010e+02	1.9428e-02	1.2968e+03	2.2834e-02	8.5778e+02	3.5292e-02	7.6142e+02
1.8680e-02	6.9873e+02	1.7814e-02	1.4916e+03	2.0937e-02	8.9334e+02	3.2360e-02	7.1028e+02
1.6988e-02	6.3659e+02	1.6200e-02	1.1775e+03	1.9040e-02	8.6610e+02	2.9428e-02	7.4274e+02
1.5295e-02	6.6081e+02	1.4586e-02	1.2646e+03	1.7144e-02	8.7658e+02	2.6496e-02	7.3230e+02
1.3603e-02	7.1908e+02	1.2972e-02	1.2323e+03	1.5247e-02	8.3149e+02	2.3565e-02	7.2645e+02
1.1911e-02	7.3628e+02	1.1358e-02	1.2543e+03	1.3350e-02	9.0318e+02	2.0633e-02	8.0779e+02
1.0218e-02	8.1293e+02	9.7443e-03	1.1282e+03	1.1453e-02	1.0084e+03	1.7701e-02	6.6387e+02
8.5257e-03	8.3182e+02	8.1304e-03	1.4432e+03	9.5559e-03	9.0680e+02	1.4769e-02	6.5432e+02
6.8333e-03	8.7716e+02	6.5165e-03	1.5560e+03	7.6590e-03	1.0238e+03	1.1837e-02	7.5643e+02
5.1409e-03	9.4658e+02	4.9025e-03	1.3992e+03	5.7621e-03	1.0391e+03	8.9056e-03	8.7231e+02
3.4484e-03	1.0506e+03	3.2886e-03	1.7142e+03	3.8651e-03	1.1440e+03	5.9738e-03	9.3042e+02
1.7560e-03	1.2692e+03	1.6746e-03	1.5848e+03	1.9682e-03	1.2798e+03	3.0420e-03	1.1389e+03
6.3625e-05	1.4955e+03	6.0675e-05	1.8838e+03	7.1313e-05	1.5970e+03	1.1022e-04	1.4383e+03

FREQUENCY [Hz]: 2.301E6		2.3108E6		2.3595E6		2.4813E6	
TAU [s]	MAGNITUDES [a.u]	TAU [s]	MAGNITUDES [a.u]	TAU [s]	MAGNITUDES [a.u]	TAU [s]	MAGNITUDES [a.u]
2.7463e-02	5.4496e+02	1.8136e-02	1.4149e+03	4.3317e-02	6.2712e+02	4.8748e-02	5.2492e+02
2.5637e-02	6.1870e+02	1.6930e-02	1.2378e+03	4.0436e-02	5.0142e+02	4.5506e-02	6.5187e+02
2.3811e-02	6.3904e+02	1.5724e-02	1.0674e+03	3.7556e-02	5.8828e+02	4.2265e-02	5.8907e+02
2.1984e-02	5.8927e+02	1.4518e-02	1.2222e+03	3.4675e-02	6.0758e+02	3.9023e-02	7.0405e+02
2.0158e-02	6.1917e+02	1.3312e-02	1.1616e+03	3.1794e-02	7.0648e+02	3.5781e-02	6.9793e+02
1.8332e-02	5.5913e+02	1.2106e-02	1.4324e+03	2.8914e-02	5.5068e+02	3.2539e-02	6.2183e+02
1.6505e-02	7.1171e+02	1.0900e-02	1.4725e+03	2.6033e-02	5.3262e+02	2.9298e-02	6.8245e+02
1.4679e-02	6.7914e+02	9.6936e-03	1.2748e+03	2.3153e-02	5.6230e+02	2.6056e-02	6.6297e+02
1.2853e-02	6.9455e+02	8.4876e-03	1.5411e+03	2.0272e-02	6.6037e+02	2.2814e-02	7.3631e+02
1.1026e-02	7.5926e+02	7.2815e-03	1.3423e+03	1.7392e-02	7.3457e+02	1.9572e-02	7.5901e+02
9.2002e-03	9.2426e+02	6.0755e-03	1.5397e+03	1.4511e-02	7.3851e+02	1.6331e-02	8.0401e+02
7.3739e-03	8.4717e+02	4.8695e-03	1.4393e+03	1.1631e-02	7.2649e+02	1.3089e-02	8.5953e+02
5.5476e-03	1.0211e+03	3.6634e-03	1.4163e+03	8.7500e-03	8.8063e+02	9.8471e-03	1.0300e+03
3.7213e-03	1.0761e+03	2.4574e-03	1.5075e+03	5.8694e-03	8.9477e+02	6.6054e-03	1.0671e+03
1.8950e-03	1.1604e+03	1.2514e-03	1.7912e+03	2.9888e-03	1.1601e+03	3.3636e-03	1.3228e+03
6.8658e-05	1.5126e+03	4.5340e-05	1.7365e+03	1.0829e-04	1.4824e+03	1.2187e-04	1.5700e+03

FREQUENCY [Hz]: 2.5957E6		2.6761E6		2.7418E6		2.8137E6	
TAU [s]	MAGNITUDES [a.u]	TAU [s]	MAGNITUDES [a.u]	TAU [s]	MAGNITUDES [a.u]	TAU [s]	MAGNITUDES [a.u]
4.7070e-02	9.3882e+02	3.6509e-02	7.1867e+02	2.9399e-02	6.9364e+02	3.5686e-02	6.5858e+02
4.3939e-02	8.6319e+02	3.4081e-02	7.4089e+02	2.7444e-02	6.9855e+02	3.3312e-02	5.3037e+02
4.0809e-02	1.0155e+03	3.1653e-02	7.2075e+02	2.5489e-02	7.6224e+02	3.0939e-02	7.2017e+02
3.7679e-02	8.4374e+02	2.9225e-02	7.2198e+02	2.3534e-02	7.3833e+02	2.8566e-02	6.8791e+02
3.4549e-02	1.0054e+03	2.6797e-02	7.3321e+02	2.1579e-02	7.7988e+02	2.6193e-02	7.4744e+02
3.1419e-02	9.6629e+02	2.4369e-02	6.7508e+02	1.9624e-02	8.4399e+02	2.3820e-02	6.4391e+02
2.8289e-02	9.2444e+02	2.1942e-02	6.6761e+02	1.7669e-02	8.3465e+02	2.1447e-02	7.8852e+02
2.5159e-02	1.0036e+03	1.9514e-02	7.5033e+02	1.5714e-02	8.9350e+02	1.9074e-02	7.8998e+02
2.2029e-02	1.1161e+03	1.7086e-02	8.2718e+02	1.3759e-02	8.5002e+02	1.6701e-02	7.8374e+02
1.8898e-02	1.0488e+03	1.4658e-02	9.1952e+02	1.1804e-02	8.6886e+02	1.4328e-02	7.7846e+02
1.5768e-02	1.0931e+03	1.2230e-02	9.1915e+02	9.8486e-03	9.5695e+02	1.1955e-02	8.0664e+02
1.2638e-02	1.1566e+03	9.8026e-03	9.5539e+02	7.8936e-03	1.1139e+03	9.5816e-03	8.8333e+02
9.5081e-03	1.1746e+03	7.3747e-03	1.1007e+03	5.9386e-03	1.1609e+03	7.2085e-03	1.0220e+03
6.3779e-03	1.4266e+03	4.9469e-03	1.3536e+03	3.9835e-03	1.2131e+03	4.8354e-03	1.0958e+03
3.2478e-03	1.5793e+03	2.5191e-03	1.4073e+03	2.0285e-03	1.4232e+03	2.4623e-03	1.4069e+03
1.1767e-04	1.7510e+03	9.1271e-05	1.5375e+03	7.3497e-05	1.5667e+03	8.9214e-05	1.5717e+03

FREQUENCY [Hz]: 2.9136E6		2.9574E6		2.9711E6		2.9987E6	
TAU [s]	MAGNITUDES [a.u]	TAU [s]	MAGNITUDES [a.u]	TAU [s]	MAGNITUDES [a.u]	TAU [s]	MAGNITUDES [a.u]
3.8751e-02	8.0376e+02	3.2437e-02	6.1653e+02	7.4887e-02	8.9811e+02	1.0000e-01	7.5952e+02
3.6174e-02	8.1273e+02	3.0280e-02	6.5258e+02	6.9907e-02	7.5062e+02	9.3350e-02	7.9649e+02
3.3597e-02	8.6219e+02	2.8123e-02	6.4426e+02	6.4927e-02	8.6632e+02	8.6700e-02	9.2678e+02
3.1020e-02	1.0773e+03	2.5966e-02	7.0313e+02	5.9947e-02	7.5340e+02	8.0050e-02	7.5067e+02
2.8443e-02	9.5685e+02	2.3809e-02	7.3719e+02	5.4967e-02	7.9611e+02	7.3400e-02	8.8836e+02
2.5866e-02	9.6749e+02	2.1652e-02	6.3841e+02	4.9987e-02	7.5198e+02	6.6750e-02	7.9322e+02
2.3289e-02	9.1262e+02	1.9495e-02	6.6870e+02	4.5007e-02	7.1922e+02	6.0100e-02	7.5760e+02
2.0712e-02	9.1172e+02	1.7338e-02	8.1371e+02	4.0027e-02	7.6091e+02	5.3450e-02	7.7646e+02
1.8135e-02	1.1042e+03	1.5181e-02	6.7608e+02	3.5047e-02	8.4817e+02	4.6800e-02	7.4017e+02
1.5558e-02	1.1566e+03	1.3024e-02	7.6733e+02	3.0067e-02	8.0100e+02	4.0150e-02	7.6653e+02
1.2981e-02	9.6315e+02	1.0867e-02	7.1407e+02	2.5087e-02	8.2466e+02	3.3500e-02	7.7445e+02
1.0405e-02	1.1218e+03	8.7094e-03	9.0220e+02	2.0107e-02	7.4063e+02	2.6850e-02	9.0521e+02
7.8276e-03	1.0022e+03	6.5524e-03	1.0113e+03	1.5127e-02	9.9973e+02	2.0200e-02	9.3375e+02
5.2507e-03	1.2119e+03	4.3953e-03	1.1356e+03	1.0147e-02	9.8584e+02	1.3550e-02	1.0571e+03
2.6738e-03	1.4260e+03	2.2382e-03	1.3402e+03	5.1672e-03	1.1447e+03	6.9000e-03	1.1365e+03
9.6877e-05	1.6154e+03	8.1094e-05	1.5629e+03	1.8722e-04	1.6892e+03	2.5000e-04	1.6353e+03

FREQUENCY [Hz]: 3.032E6		3.1104E6		3.1584E6		3.1897E6	
TAU [s]	MAGNITUDES [a.u]	TAU [s]	MAGNITUDES [a.u]	TAU [s]	MAGNITUDES [a.u]	TAU [s]	MAGNITUDES [a.u]
5.1464e-02	5.6342e+02	5.5674e-02	5.7396e+02	9.7612e-02	6.0971e+02	7.3167e-02	6.1336e+02
4.8041e-02	6.3161e+02	5.1972e-02	6.4431e+02	9.1120e-02	8.1988e+02	6.8302e-02	6.4385e+02
4.4619e-02	5.9567e+02	4.8269e-02	6.7743e+02	8.4629e-02	7.6348e+02	6.3436e-02	6.3302e+02
4.1197e-02	6.4004e+02	4.4567e-02	5.6389e+02	7.8138e-02	6.2091e+02	5.8570e-02	5.5748e+02
3.7774e-02	7.0863e+02	4.0865e-02	5.9614e+02	7.1647e-02	7.3610e+02	5.3705e-02	5.8887e+02
3.4352e-02	6.2857e+02	3.7162e-02	6.6210e+02	6.5156e-02	7.9165e+02	4.8839e-02	6.0425e+02
3.0930e-02	7.3191e+02	3.3460e-02	7.0125e+02	5.8665e-02	7.5895e+02	4.3974e-02	6.9194e+02
2.7507e-02	6.4756e+02	2.9758e-02	6.6655e+02	5.2173e-02	7.3308e+02	3.9108e-02	7.1725e+02
2.4085e-02	7.2454e+02	2.6055e-02	8.3013e+02	4.5682e-02	6.8535e+02	3.4242e-02	7.5368e+02
2.0663e-02	6.6796e+02	2.2353e-02	7.3948e+02	3.9191e-02	7.0191e+02	2.9377e-02	7.5031e+02
1.7240e-02	8.4746e+02	1.8651e-02	8.0056e+02	3.2700e-02	9.0029e+02	2.4511e-02	7.6756e+02
1.3818e-02	8.9499e+02	1.4948e-02	8.4885e+02	2.6209e-02	9.2909e+02	1.9645e-02	7.8316e+02
1.0396e-02	9.1632e+02	1.1246e-02	1.0137e+03	1.9718e-02	8.3539e+02	1.4780e-02	9.7776e+02
6.9733e-03	9.9305e+02	7.5438e-03	1.1549e+03	1.3226e-02	1.0642e+03	9.9142e-03	1.1213e+03
3.5510e-03	1.2311e+03	3.8415e-03	1.3333e+03	6.7352e-03	1.2642e+03	5.0485e-03	1.3444e+03
1.2866e-04	1.6749e+03	1.3919e-04	1.5494e+03	2.4403e-04	1.7146e+03	1.8292e-04	1.6213e+03

FREQUENCY [Hz]: 3.2701E6		3.2816E6		3.5261E6		3.7391E6	
TAU [s]	MAGNITUDES [a.u]	TAU [s]	MAGNITUDES [a.u]	TAU [s]	MAGNITUDES [a.u]	TAU [s]	MAGNITUDES [a.u]
1.2352e-01	6.0234e+02	1.1329e-01	7.4290e+02	1.1635e-01	6.5463e+02	1.4860e-01	8.0141e+02
1.1531e-01	6.3301e+02	1.0576e-01	7.9226e+02	1.0861e-01	7.2097e+02	1.3872e-01	7.5102e+02
1.0709e-01	6.5054e+02	9.8222e-02	7.7579e+02	1.0088e-01	7.0578e+02	1.2884e-01	7.9798e+02
9.8879e-02	5.7267e+02	9.0688e-02	7.3210e+02	9.3138e-02	6.7772e+02	1.1896e-01	8.3155e+02
9.0664e-02	6.1767e+02	8.3155e-02	8.3131e+02	8.5401e-02	7.7105e+02	1.0907e-01	8.2381e+02
8.2450e-02	6.2894e+02	7.5621e-02	8.9959e+02	7.7663e-02	6.6331e+02	9.9192e-02	8.1532e+02
7.4236e-02	6.1142e+02	6.8087e-02	8.3493e+02	6.9926e-02	7.2821e+02	8.9310e-02	9.0778e+02
6.6022e-02	5.9774e+02	6.0553e-02	8.8477e+02	6.2189e-02	7.8252e+02	7.9428e-02	7.3302e+02
5.7808e-02	5.9406e+02	5.3020e-02	8.9199e+02	5.4452e-02	8.5760e+02	6.9546e-02	8.1376e+02
4.9594e-02	6.9754e+02	4.5486e-02	8.9808e+02	4.6714e-02	8.1749e+02	5.9664e-02	8.7102e+02
4.1380e-02	6.1832e+02	3.7952e-02	8.5391e+02	3.8977e-02	9.0013e+02	4.9782e-02	1.0284e+03
3.3165e-02	7.7917e+02	3.0418e-02	1.0003e+03	3.1240e-02	9.6814e+02	3.9900e-02	9.2002e+02
2.4951e-02	8.9205e+02	2.2884e-02	1.0198e+03	2.3503e-02	9.5837e+02	3.0018e-02	1.0512e+03
1.6737e-02	1.0704e+03	1.5351e-02	1.1661e+03	1.5765e-02	1.1963e+03	2.0136e-02	1.2880e+03
8.5230e-03	1.2477e+03	7.8170e-03	1.4197e+03	8.0281e-03	1.4938e+03	1.0254e-02	1.4594e+03
3.0880e-04	1.6315e+03	2.8322e-04	1.6719e+03	2.9087e-04	1.6372e+03	3.7151e-04	1.7817e+03

FREQUENCY [Hz]: 3.8042E6		3.9014E6		3.9998E6		4.429E6	
TAU [s]	MAGNITUDES [a.u]	TAU [s]	MAGNITUDES [a.u]	TAU [s]	MAGNITUDES [a.u]	TAU [s]	MAGNITUDES [a.u]
1.1503e-01	6.3994e+02	1.0836e-01	6.5364e+02	1.4000e-01	8.7657e+02	1.5570e-01	7.3376e+02
1.0738e-01	5.9323e+02	1.0115e-01	6.5729e+02	1.3069e-01	8.6602e+02	1.4535e-01	8.2097e+02
9.9728e-02	5.5235e+02	9.3944e-02	6.4025e+02	1.2138e-01	8.5995e+02	1.3499e-01	9.1262e+02
9.2079e-02	5.8586e+02	8.6739e-02	6.6579e+02	1.1207e-01	7.9288e+02	1.2464e-01	8.0752e+02
8.4430e-02	6.9085e+02	7.9533e-02	6.3066e+02	1.0276e-01	8.1116e+02	1.1429e-01	9.1183e+02
7.6781e-02	6.4349e+02	7.2327e-02	6.1236e+02	9.3450e-02	7.9378e+02	1.0393e-01	8.9601e+02
6.9131e-02	6.5107e+02	6.5122e-02	6.0941e+02	8.4140e-02	9.0994e+02	9.3577e-02	1.0905e+03
6.1482e-02	6.9277e+02	5.7916e-02	7.3214e+02	7.4830e-02	9.3007e+02	8.3223e-02	9.0881e+02
5.3833e-02	7.3221e+02	5.0710e-02	6.9353e+02	6.5520e-02	9.3629e+02	7.2868e-02	8.6076e+02
4.6183e-02	6.9497e+02	4.3505e-02	8.0392e+02	5.6210e-02	9.0140e+02	6.2514e-02	1.1049e+03
3.8534e-02	8.2048e+02	3.6299e-02	8.7162e+02	4.6900e-02	1.0118e+03	5.2160e-02	1.1231e+03
3.0885e-02	9.9675e+02	2.9093e-02	1.0406e+03	3.7590e-02	1.1214e+03	4.1806e-02	1.0872e+03
2.3235e-02	1.0637e+03	2.1888e-02	1.0438e+03	2.8280e-02	1.3217e+03	3.1452e-02	1.2241e+03
1.5586e-02	1.2133e+03	1.4682e-02	1.3208e+03	1.8970e-02	1.3780e+03	2.1098e-02	1.3713e+03
7.9369e-03	1.4074e+03	7.4765e-03	1.5934e+03	9.6600e-03	1.5716e+03	1.0743e-02	1.5586e+03
2.8757e-04	1.6786e+03	2.7089e-04	1.5749e+03	3.5000e-04	1.8091e+03	3.8925e-04	1.8404e+03

FREQUENCY [Hz]: 5.2466E6		6.2167E6		7.3598E6		8.7167E6	
TAU [s]	MAGNITUDES [a.u]	TAU [s]	MAGNITUDES [a.u]	TAU [s]	MAGNITUDES [a.u]	TAU [s]	MAGNITUDES [a.u]
1.9874e-01	8.9499e+02	2.4079e-01	9.0307e+02	2.5678e-01	8.8475e+02	3.2434e-01	1.1176e+03
1.8552e-01	9.1164e+02	2.2478e-01	9.1893e+02	2.3970e-01	9.0864e+02	3.0277e-01	9.3787e+02
1.7231e-01	8.0994e+02	2.0876e-01	7.4913e+02	2.2263e-01	8.3350e+02	2.8120e-01	9.7501e+02
1.5909e-01	9.6876e+02	1.9275e-01	8.5321e+02	2.0555e-01	7.8894e+02	2.5963e-01	9.3407e+02
1.4587e-01	8.4141e+02	1.7674e-01	8.9287e+02	1.8847e-01	9.4688e+02	2.3806e-01	9.6357e+02
1.3266e-01	8.9287e+02	1.6073e-01	9.3265e+02	1.7140e-01	9.5618e+02	2.1649e-01	1.0079e+03
1.1944e-01	9.6694e+02	1.4471e-01	8.9002e+02	1.5432e-01	1.0429e+03	1.9493e-01	1.1184e+03
1.0623e-01	1.0079e+03	1.2870e-01	8.8090e+02	1.3725e-01	9.9160e+02	1.7336e-01	9.8900e+02
9.3009e-02	9.1093e+02	1.1269e-01	9.6691e+02	1.2017e-01	9.5027e+02	1.5179e-01	1.0022e+03
7.9793e-02	9.8573e+02	9.6677e-02	8.6729e+02	1.0310e-01	9.9176e+02	1.3022e-01	1.1056e+03
6.6577e-02	9.8118e+02	8.0665e-02	1.0315e+03	8.6020e-02	1.0213e+03	1.0865e-01	1.1465e+03
5.3361e-02	1.0389e+03	6.4652e-02	1.0950e+03	6.8945e-02	1.1903e+03	8.7084e-02	1.2056e+03
4.0145e-02	1.1794e+03	4.8640e-02	1.1640e+03	5.1869e-02	1.3030e+03	6.5516e-02	1.1476e+03
2.6929e-02	1.4389e+03	3.2627e-02	1.3753e+03	3.4793e-02	1.2997e+03	4.3947e-02	1.4051e+03
1.3713e-02	1.3597e+03	1.6615e-02	1.5442e+03	1.7718e-02	1.4364e+03	2.2379e-02	1.6783e+03
4.9684e-04	1.7130e+03	6.0197e-04	1.7045e+03	6.4194e-04	1.7596e+03	8.1084e-04	1.7095e+03

FREQUENCY [Hz]: 1.0326E7		1.4485E7		1.7163E7		2.0326E7	
TAU [s]	MAGNITUDES [a.u]	TAU [s]	MAGNITUDES [a.u]	TAU [s]	MAGNITUDES [a.u]	TAU [s]	MAGNITUDES [a.u]
3.0733e-01	1.0443e+03	6.0794e-01	1.1277e+03	6.8835e-01	1.3321e+03	8.1351e-01	1.6323e+03
2.8689e-01	1.1202e+03	5.6751e-01	1.2039e+03	6.4257e-01	1.2549e+03	7.5941e-01	1.5582e+03
2.6645e-01	9.4573e+02	5.2708e-01	1.3005e+03	5.9680e-01	1.2619e+03	7.0531e-01	1.4388e+03
2.4602e-01	1.1466e+03	4.8666e-01	1.1946e+03	5.5102e-01	1.3233e+03	6.5121e-01	1.5088e+03
2.2558e-01	9.9473e+02	4.4623e-01	1.2201e+03	5.0525e-01	1.3666e+03	5.9712e-01	1.5071e+03
2.0514e-01	9.7824e+02	4.0580e-01	1.2588e+03	4.5947e-01	1.2990e+03	5.4302e-01	1.5071e+03
1.8470e-01	9.6689e+02	3.6537e-01	1.2727e+03	4.1370e-01	1.2972e+03	4.8892e-01	1.5935e+03
1.6427e-01	1.0455e+03	3.2494e-01	1.2145e+03	3.6792e-01	1.2706e+03	4.3482e-01	1.5582e+03
1.4383e-01	1.0943e+03	2.8452e-01	1.2500e+03	3.2215e-01	1.3091e+03	3.8072e-01	1.4738e+03
1.2339e-01	9.8562e+02	2.4409e-01	1.0994e+03	2.7637e-01	1.2636e+03	3.2662e-01	1.4624e+03
1.0296e-01	1.0002e+03	2.0366e-01	1.0537e+03	2.3060e-01	1.1454e+03	2.7253e-01	1.4553e+03
8.2518e-02	9.4614e+02	1.6323e-01	1.0690e+03	1.8482e-01	1.1101e+03	2.1843e-01	1.2200e+03
6.2080e-02	8.0857e+02	1.2280e-01	1.0965e+03	1.3905e-01	1.0960e+03	1.6433e-01	1.0781e+03
4.1643e-02	8.1429e+02	8.2376e-02	9.2988e+02	9.3271e-02	9.1936e+02	1.1023e-01	1.0876e+03
2.1206e-02	7.5992e+02	4.1948e-02	7.9410e+02	4.7496e-02	8.3546e+02	5.6132e-02	8.7525e+02
7.6832e-04	7.0978e+02	1.5198e-03	7.0978e+02	1.7209e-03	6.9801e+02	2.0338e-03	5.9958e+02

FREQUENCY [Hz]: 2.4069E7		2.8511E7		3.3772E7		4.0003E7	
TAU [s]	MAGNITUDES [a.u]	TAU [s]	MAGNITUDES [a.u]	TAU [s]	MAGNITUDES [a.u]	TAU [s]	MAGNITUDES [a.u]
1.0402e+00	1.7616e+03	1.1568e+00	2.1358e+03	1.2069e+00	2.4377e+03	1.2000e+00	2.7629e+03
9.7105e-01	1.7451e+03	1.0798e+00	2.0486e+03	1.1266e+00	2.3278e+03	1.1202e+00	2.7673e+03
9.0187e-01	1.7371e+03	1.0029e+00	2.0489e+03	1.0464e+00	2.2787e+03	1.0404e+00	2.7251e+03
8.3270e-01	1.7442e+03	9.2598e-01	2.0189e+03	9.6613e-01	2.3187e+03	9.6060e-01	2.7132e+03
7.6352e-01	1.6872e+03	8.4905e-01	1.9179e+03	8.8587e-01	2.3267e+03	8.8080e-01	2.6137e+03
6.9435e-01	1.8365e+03	7.7213e-01	2.0097e+03	8.0561e-01	2.4556e+03	8.0100e-01	2.7331e+03
6.2517e-01	1.8098e+03	6.9521e-01	1.9390e+03	7.2535e-01	2.3770e+03	7.2120e-01	2.5683e+03
5.5600e-01	1.7367e+03	6.1828e-01	1.8791e+03	6.4509e-01	2.2361e+03	6.4140e-01	2.5978e+03
4.8682e-01	1.7646e+03	5.4136e-01	1.8856e+03	5.6483e-01	2.1699e+03	5.6160e-01	2.4859e+03
4.1765e-01	1.6947e+03	4.6444e-01	1.8976e+03	4.8457e-01	2.0352e+03	4.8180e-01	2.4587e+03
3.4847e-01	1.6139e+03	3.8751e-01	1.7142e+03	4.0431e-01	2.0067e+03	4.0200e-01	2.2252e+03
2.7930e-01	1.5029e+03	3.1059e-01	1.7215e+03	3.2405e-01	1.8379e+03	3.2220e-01	2.0808e+03
2.1013e-01	1.3147e+03	2.3366e-01	1.4141e+03	2.4380e-01	1.7579e+03	2.4240e-01	1.7501e+03
1.4095e-01	1.1987e+03	1.5674e-01	1.2897e+03	1.6354e-01	1.4255e+03	1.6260e-01	1.6059e+03
7.1775e-02	8.9333e+02	7.9816e-02	1.0135e+03	8.3277e-02	1.0327e+03	8.2800e-02	1.0555e+03
2.6006e-03	7.0273e+02	2.8919e-03	6.4234e+02	3.0173e-03	7.7467e+02	3.0000e-03	7.1764e+02
